# Proteomics and Bioinformatics Investigations Link Overexpression of FGF8 and Associated Hub Genes to the Progression of Ovarian Cancer and Poor Prognosis

**DOI:** 10.1155/2024/4288753

**Published:** 2024-09-13

**Authors:** Vikrant Kumar, Anil Kumar Tomar, Ayushi Thapliyal, Savita Yadav

**Affiliations:** Department of Biophysics All India Institute of Medical Sciences, New Delhi 11029, India

## Abstract

Ovarian cancer's asymptomatic nature, high recurrence rate, and resistance to platinum-based chemotherapy highlight the need to find and characterize new diagnostic and therapeutic targets. While prior studies have linked aberrant expression of fibroblast growth factor 8 (FGF8) to various cancer types, its precise role has remained elusive. Recently, we observed that FGF8 silencing reduces the cancer-promoting properties of ovarian cancer cells, and thus, this study aimed to understand how FGF8 regulates the development of ovarian cancer. LC-MS/MS-based quantitative proteomics analysis identified 418 DEPs, and most of them were downregulated in FGF8-silenced ovarian cancer cells. Many of these DEPs are associated with cancer progression and unfavorable prognosis. To decipher the biological significance of DEPs, bioinformatics analyses encompassing gene ontology, pathway analysis, protein-protein interaction networks, and expression analysis of hub genes were carried out. Hub genes identified in the FGF8 protein network were upregulated in ovarian cancer compared to controls and were linked to poor prognosis. Subsequently, the expression of hub genes was correlated with patient survival and regulation of the tumor microenvironment. Conclusively, FGF8 and associated hub genes help in the progression of ovarian cancer, and their overexpression may lead to higher immune infiltration, poor prognosis, and poor survival.

## 1. Introduction

The seventh most frequent cancer globally in 2020, ovarian cancer, is a complex illness that typically arises from the ovary and nearby structures, including fallopian tubes and the peritoneum. The most prevalent form of ovarian cancer, epithelial ovarian cancer (EOC), which makes up more than 90% of all cases, has a high mortality rate due to its asymptomatic nature, resistance to platinum-based therapy, and high recurrence rate [1]. Healthcare professionals claim that early detection of ovarian cancer can unquestionably raise patient survival rates, which emphasizes the need for new and improved biomarkers. The most reliable biomarker for the early detection of ovarian cancer is serum cancer antigen 125 (CA-125), but it lacks specificity and has been reported to be increased in other benign and nongynecological disorders [2]. To search for and establish a sensitive and specific ovarian cancer screening biomarker, advanced research investigations are required at the molecular level. The mechanisms underlying tumor progression and metastasis in ovarian cancer are not fully understood [3]. These mechanisms are intricate and involve various genetic and epigenetic alterations. The tumor microenvironment (TME), which consists of extracellular matrix elements (chemokines, cytokines, and integrins), cancer stem cells, cancer-associated fibroblasts, endothelial cells, and immune cells, plays a crucial role in ovarian cancer progression [4]. Numerous studies have identified key risk factors and gene mutations associated with ovarian cancer. A family history of early-stage ovarian or breast cancer and mutations in tumor suppressor genes such as TP53, BRCA1, and BRCA2 are significant risk factors for ovarian cancer [5–7]. The MAPK pathway is also involved in the pathogenesis of ovarian cancer. This pathway is often dysregulated through mutations in KRAS or BRAF and activation of the PI3K/AKT/mTOR pathway in ovarian cancer cells [8, 9]. The long-term use of hormone replacement therapy, particularly estrogen-only therapy, also increases the risk of ovarian cancer development and progression in postmenopausal women [10–12].

Fibroblast growth factor 8 (FGF8) protein is a 26 kDa growth factor with mitogenic and cell survival properties. It is associated with a variety of biological processes, including tumor growth and invasion, tissue healing, cell proliferation, morphogenesis, and embryonic development [13]. FGF8 is reported to express extensively throughout embryonic development and in many cancers, including breast, ovarian, and prostate cancer, but its expression is much more constrained in normal adult tissues. The regulation of growth and progression of hormonal cancer is regulated by FGF8, and any modification in the growth factor signaling cascade is linked to cancer progression [14–18]. In addition to controlling events such as cell differentiation, proliferation, and angiogenesis, FGF8 interacts with FGF receptors (FGFRs) and regulates fundamental developmental pathways during embryonic development [19–21]. Heparan sulphate proteoglycans regulate FGF8 signaling and its interaction with FGFR, which activates several cell signaling cascades, including the MAPK, phospholipase C gamma, and phosphatidylinositol-3 kinase pathways [22, 23]. FGF8 has recently been linked to tumor progression in clinical samples from patients with prostate and breast cancer [24].

Targeting FGF8 may be an effective treatment strategy for some cancer types, including ovarian cancer, as various studies have linked abnormal FGF8 expression to the onset and progression of different cancers. We have recently reported progressive overexpression of FGF8 in tissue samples, from control samples to low-grade and high-grade EOC samples, observed at both the protein and mRNA levels [25]. Also, silencing of the FGF8 gene in SKOV3 cells caused detrimental effects on several cellular properties crucial for cancer cell survival and metastasis, implying a potential prometastatic role of FGF8 in EOC [25]. However, to understand the definite role of FGF8, more research is necessary. Therefore, to gain functional insights into FGF8 through molecular interplays and associated pathways in ovarian cancer progression, differential proteomics analysis utilizing LC-MS/MS-based label-free quantitation was applied to identify alterations in proteins of FGF8-silenced ovarian cancer cells. Additionally, protein-protein interaction networks were built using bioinformatics tools, and hub genes were identified. Studying hub genes is crucial for gaining a comprehensive understanding of a process or disease because these genes interact with numerous other genes and typically play a pivotal role in biological processes and gene regulation. The identified hub genes were further investigated for their expression levels, prognostic significance, mutational burden, and role in immune cell infiltration in the TME. We believe that exploratory studies in this direction might help in the development of a potential therapeutic approach.

## 2. Material and Methods

### 2.1. Cell Culture, RNA Interference, and FGF8 Silencing

Ovarian cancer cell lines (SKOV3) were obtained from the National Centre for Cell Science (NCCS), Pune, cultured in McCoy's 5-a modified medium containing 10% deactivated fetal bovine serum (Fisher Scientific), and maintained in 5% CO_2_ at 37°C. The cells were transfected with FGF8 siRNA (siRNA ID: HSS142008). As described previously, 40 pmol of FGF8-specific siRNA was used to achieve optimum inhibition of FGF8 expression in SKOV3 cells [25]. For this, 3 × 10^5^ cells were seeded in six-well plates for 24 h until cells reached at least 70% confluency. After that, the complete media was replaced with Opti-MEM. Transfection media was prepared by adding siRNAs and lipofectamine to Opti-MEM, followed by a 15-minute incubation. It was then added to all the experiment wells, and cells were incubated for 48 h.

### 2.2. Western Blotting

Post-transfection, cells were harvested and lysed in RIPA buffer for 45 min, followed by 5 cycles of sonication (30% amplitude, 5 sec ON, 55 sec OFF). After sonication, the cell lysate was centrifuged. The supernatant containing proteins was collected, and cell debris (pellet) was discarded. The total protein content in all the samples was quantified by the Bradford assay, and 30 *μ*g of protein was resolved on 12.5% SDS-PAGE gel. After separation, proteins were transferred onto a 0.2-um PVDF membrane and blocked with 5% nonfat milk (w/v) for 2 h. The membrane was then incubated in anti-FGF8 primary antibodies at 4°C for overnight and in HRP-conjugated secondary antibodies for 2 h. After thorough washing, FGF8 bands were visualized by ECL (Bio-Rad Labs, USA) on the Syngene G-box instrument (Syngene, USA). *β*-tubulin was used as an internal control.

### 2.3. Quantitative Proteomic Analysis

LC-MS/MS-based differential proteomics analysis was carried out as described previously [26, 27]. Briefly, protein samples (50 *μ*l and 1 *μ*g/*μ*l) from siRNA-transfected and the control groups were first reduced with 5 mM tris (2-carboxyethyl) phosphine, followed by alkylation with 50 mM iodoacetamide. Proteins were then digested by incubating samples in 1 *μ*g of mass spectrometry grade trypsin (Promega, USA) for 16 h at 37°C. The digested peptide samples were cleaned using a C18 silica cartridge, vacuum dried, and resuspended in Buffer A (5% acetonitrile and 0.1% formic acid). The separation and mass spectrometric analyses of peptides were performed on an EASY-nLC-1200 system attached to an Orbitrap Exploris Mass Spectrometer (Thermo Fisher Scientific). Peptides were separated onto a 15-cm, 3.0-*μ*m EASY-Spray C18 column and eluted with a gradient of buffer B (containing 95% acetonitrile and 0.1% formic acid; gradient: 0–40%, time: 100 min, and flow rate: 300 nL/min). MS1 (resolution 70,000) and MS2 (resolution 17,500) spectra were acquired, and raw files were processed and searched against UniProt's human proteome database using the Proteome Discoverer™ software v2.2 (Thermo Fisher Scientific). The peptide-spectrum match (PSM) and protein false discovery rate (FDR) were set to 0.01. Other important search parameters were the maximum missed enzymatic cleavages allowed: 02; the precursor mass tolerance: 10 ppm; fragment mass tolerances: 0.5 Da; fixed modification: carbamidomethyl on cysteine; and variable modifications: oxidation of methionine and N-terminal acetylation. The Proteome Discoverer framework using the Minora feature detection approach was applied to perform label-free quantitation of identified proteins.

### 2.4. Differential Statistical Analysis

MetaboAnalyst 5.0 was utilized to perform statistical analysis to identify differentially expressed proteins (DEPs) in siRNA-transfected cells [28]. The following analyses were performed: volcano plot analysis, principal component analysis (PCA), partial least squares-discriminant analysis (PLS-DA), and hierarchical clustering. Before analysis, missing values, if any, were added by the kNN imputation method, and Pareto scaling was applied for data normalization. Proteins with a fold change of ≥2 and a *p* value ≤0.05 were considered differentially expressed. DEPs with higher and lower abundance in a particular group were categorised as upregulated and downregulated, respectively.

### 2.5. Bioinformatics Analysis

The Database for Annotation, Visualization, and Integrated Discovery (DAVID) Gene Enrichment tool v6.8 was used for functional annotations and pathway analysis of downregulated proteins in FGF8-silenced cells [29]. The STRING database v11 was explored for protein-protein interaction (PPI) network analysis [30]. The PPI network generated by STRING was transferred to Cytoscape 3.10.1 to identify hub genes using the cytoHubba plugin [31]. To generate the PPI network of DEPs, the following parameters were set in settings: window-network type: full STRING network; network edges: confidence; interaction sources: all; minimum interaction score: 0.4; and network display: hide disconnected nodes in the network. After generation, the network was divided into 3 clusters using the k-means clustering method.

### 2.6. Expression of Hub Genes in Ovarian Cancer

The relative expression of hub genes (at protein level) in normal and ovarian cancer samples and their association with individual cancer stages were analyzed by the UALCAN database (https://ualcan.path.uab.edu/analysis-prot.html) using data from the “Clinical Proteomic Tumor Analysis Consortium” (CPTAC) [32]. The UALCAN database is an interactive web resource that offers analysis of cancer omics data for 14 different types of cancers, including breast cancer, clear cell renal cell carcinoma, colorectal cancer, gastric cancer, glioblastoma, ovarian cancer, head and neck squamous cell carcinoma, liver cancer, lung adenocarcinoma, lung squamous cell carcinoma, pancreatic cancer, pediatric brain tumors, prostate cancer, and uterine corpus endometrial carcinoma. Further, tissue expression data (immunohistochemistry) of hub genes in ovarian cancer and normal ovary were retrieved from the human protein Atlas database (https://www.proteinatlas.org) [33]. The human protein Atlas database comprises proteomic data based on 26,941 antibodies that target 17,165 unique proteins. To understand if the expression of these hub genes influences the life expectancy of ovarian cancer patients, survival analysis was performed by the Kaplan–Meier plotter [34]. This analysis also included the computation of the hazard ratio (HR) with 95% confidence intervals and log-rank *p* values. The Kaplan–Meier plotter can assess the correlation between the expression of all genes (presently recognizing 70,632 gene symbols) and survival in more than 30 thousand samples from 21 tumor types, including ovarian cancer.

### 2.7. Genomic Alterations in Hub Genes in Ovarian Cancer

CBioPortal integrates genomic data from different databases, such as the International Cancer Genome Consortium (ICGC) and the Cancer Genome Atlas (TCGA), and offers an in-depth analysis [35]. Genomic alterations in hub genes, including copy-number deep deletion, amplification, and missense mutation, in ovarian cancer samples were acquired from the cBioPortal database.

### 2.8. Hub Genes and Immune Cell Infiltration

The Tumor Immune Estimate Resource (TIMER), an integrated online server, was used to evaluate the prevalence of tumor-infiltrating immune cells (TIICs) [36]. The TIMER uses a deconvolution statistical technique on gene expression profiles to estimate the abundance of TIICs.

## 3. Results

### 3.1. Differentially Expressed Proteins

**Figure 1:**
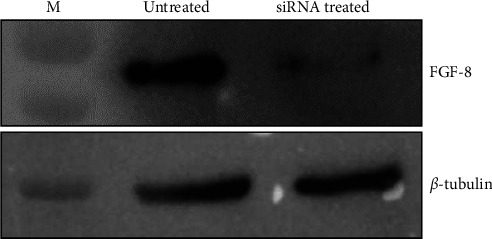
Western blot analysis to monitor FGF8 expression in ovarian cancer cells (SKOV3) after FGF8 knockdown. To knockdown FGF8 in cells, 40 pmol of siRNA was used. Lane M: molecular weight markers.

**Figure 2:**
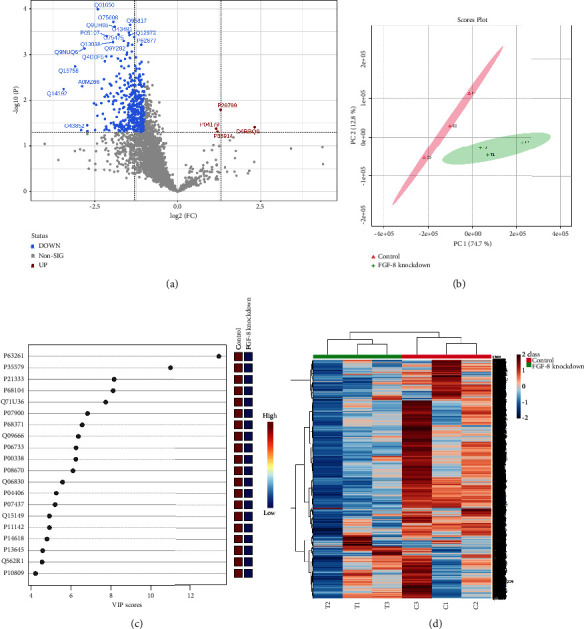
Differentially expressed proteins (DEPs) in FGF8-silenced cells. (a) Volcano plot analysis; (b) principal component analysis (PCA) score plot; (c) top 20 DEPs identified by PLS-DA; colored boxes on the right indicate the relative abundance of the corresponding proteins; and (d) hierarchical clustering analysis.

FGF8 was knocked down using anti-human FGF8 siRNA (40 pmol) and confirmed by western blot analysis (Figure 1). Proteins were extracted from FGF8-expressing (control) and FGF8-silenced ovarian cancer cells and processed for differential proteomics analysis by LC-MS/MS. In total, 21,403 peptide groups corresponding to 3,018 proteins were identified in the processed samples (Supplementary File 1). Out of 3018 proteins, 2,751 were identified in both groups, while 90 and 177 proteins were exclusively identified in FGF8-silenced and control cells, respectively (Supplementary Figure 1). For label-free quantitation, all the proteins identified with one or more unique peptides were processed. Protein abundance data were processed for the identification of DEPs as described in the methodology section. A volcano plot analysis with a fold change threshold of 2 and a *p* value ≤0.05 identified 418 DEPs, most of which were found downregulated in FGF8-silenced cells (Figure 2(a), Supplementary File 2). Precisely, 414 and 4 proteins were found to be downregulated and upregulated, respectively, in FGF8-silenced cells. Ubiquitin carboxyl-terminal hydrolase 17-like protein 17 (USP17L17), superoxide dismutase [Mn], mitochondrial (SOD2), progranulin (GRN), hydroxymethylglutaryl-CoA lyase, and mitochondrial (HMGCL) were found upregulated, whilst the list of downregulated proteins included large neutral amino acids transporter small subunit 1 (SLC7A5), four and a half LIM domains protein 2 (FHL2), neutral amino acid transporter B, calumenin, SPATS2-like protein (SPATS2L), dolichyl-diphosphooligosaccharide-protein glycosyltransferase subunit DAD1 (DAD1), plasma membrane calcium-transporting ATPase 4 (ATP2B4), tumor protein D52 (TPD52), RNA polymerase II-associated protein 3 (RPAP3), DNA replication licensing factor MCM2 (MCM2), ataxin-2 (ATXN2), integrin beta-2 (ITGB2), adapter molecule crk (CRK), cytochrome c oxidase subunit 5A, mitochondrial (COX5A), calcium homeostasis endoplasmic reticulum protein (CHERP), nicotinate phosphoribosyltransferase (NAPRT), exportin-7 (XPO7), lipoma-preferred partner (LPP), phosphoserine aminotransferase (PSAT1), C-terminal-binding protein 2 (CTBP2), plakophilin-3 (PKP3), thioredoxin-related transmembrane protein 1 (TMX1), protein S100-A6 (S100A6), splicing factor 3B subunit 4 (SF3B4), lymphokine-activated killer T-cell-originated protein kinase (PBK), and beta-1,4-galactosyltransferase 2 (B4GALT2). The top DEPs are listed in Table 1.

### 3.2. Statistical Significance of DEPs

The abundance variation of proteins in different samples was displayed using a two-dimensional PCA, which reduces the level of complexity in high-dimensional data. Between FGF8-silenced and control cells, there was a clear separation visible on the PCA score plot (Figure 2(b)). Similarly, the PLS-DA score plots also easily distinguished FGF8-silenced and control groups. PLS-DA is a supervised multivariate regression method that recognizes features with the ability to set the groups apart and produce the VIP scores. For variability, features with VIP scores ≥1 are considered significant classifiers. A total of 404 proteins were identified with VIP scores ≥1, including actin, cytoplasmic 2 (ACTG1), myosin-9 (MYH9), filamin-A (FLNA), elongation factor 1-alpha 1 (EEF1A1), heat shock protein HSP 90-alpha (HSP90AA1), neuroblast differentiation-associated protein AHNAK (AHNAK), alpha-enolase (ENO1), L-lactate dehydrogenase A chain (LDHA), vimentin (VIM), peroxiredoxin-1 (PRDX1), and plectin (PLEC) (Figure 2(c)). The samples of the FGF8-silenced and control cells were accurately sorted in discrete clusters by HCA, which groups data points with similar properties into clusters (Figure 2(d)). Together, these results demonstrate the statistical capability of the identified DEPs to categorize these groups.

### 3.3. Bioinformatics Analysis

**Figure 3:**
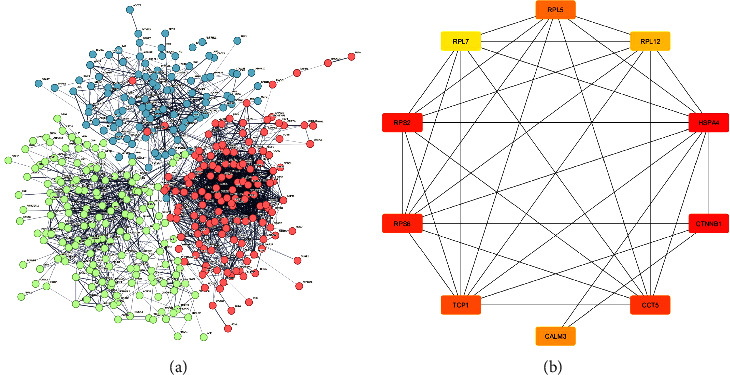
Protein-protein interaction (PPI) network and hub genes. (a) PPI network of DEPs in FGF8-silenced ovarian cancer cells and (b) subnetwork of the top 10 hub genes.

Among the proteins with lower abundance in FGF8-silenced ovarian cancer cells, a total of 67 GO terms were enriched (8 biological processes including translation, proteasomal protein catabolic process, protein stabilization, protein transport, and protein UFMylation; 45 cellular components including adherens junction, mitochondrial inner membrane, nuclear envelope, Golgi apparatus, actin filament, endoplasmic reticulum, proteasome core complex, endoplasmic reticulum membrane, telomeric region, chaperone complex, nuclear membrane, and centrosome; and 14 molecular functions including a structural constituent of ribosome, actin binding, endopeptidase activity, phosphatidylinositol binding, ATP binding, protein binding, threonine-type endopeptidase activity, and calmodulin binding). Also, 11 KEGG pathways were under-represented in FGF-8-silenced cells (Supplementary file 3). PPI network analysis generated a dense network, showing that the majority of altered proteins were interconnected (Figure 3(a)). This network was then transferred to Cytoscape to identify hub genes. The top 10 hub genes based on closeness were identified, which included TCP1, RPS6, RPL12, CTNNB1, RPL5, RPL7, HSPA4, CCT5, RPS2, and CALM3 (Figure 3(b)).

### 3.4. Expression of Hub Genes

**Figure 4:**
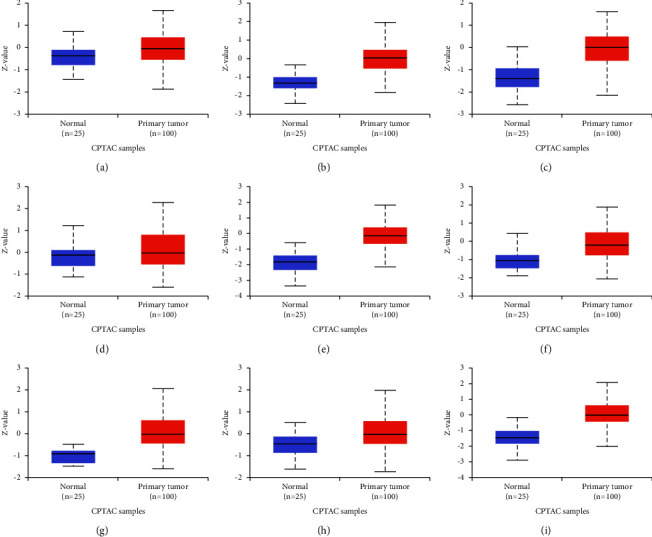
Differential expression of hub genes at the protein level in ovarian cancer. The UALCAN database was used to identify differential expression of hub genes. (a) TCP1; (b) RPS6; (c) RPL12; (d) CTNNB1; (e) RPL5; (f) RPL7; (g) HSPA4; (h) CCT5; and (i) RPS2 (*p* ≤ 0.05; normal = 25; primary tumor = 100). CALM3 expression data were not available.

**Figure 5:**
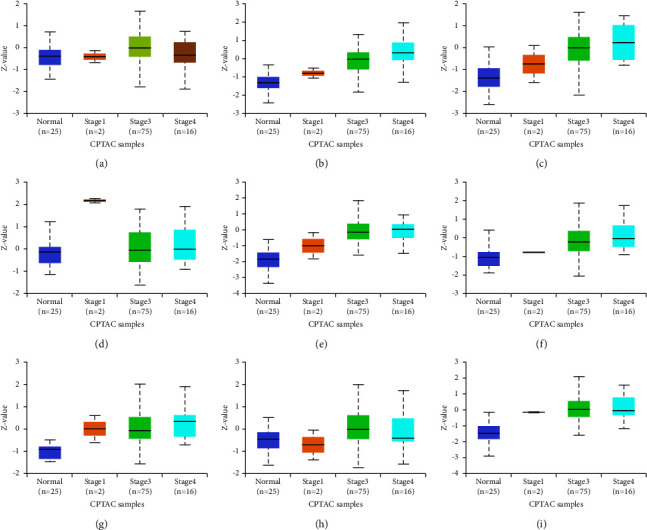
Expression of hub genes in different stages of ovarian cancer. (a) TCP1; (b) RPS6; (c) RPL12; (d) CTNNB1; (e) RPL5; (f) RPL7; (g) HSPA4; (h) CCT5; and (i) RPS2 (*p* value ≤ 0.05; normal = 25; stage 1 = 2; stage 3 = 75; stage 4 = 16). CALM3 expression data were not available.

**Figure 6:**
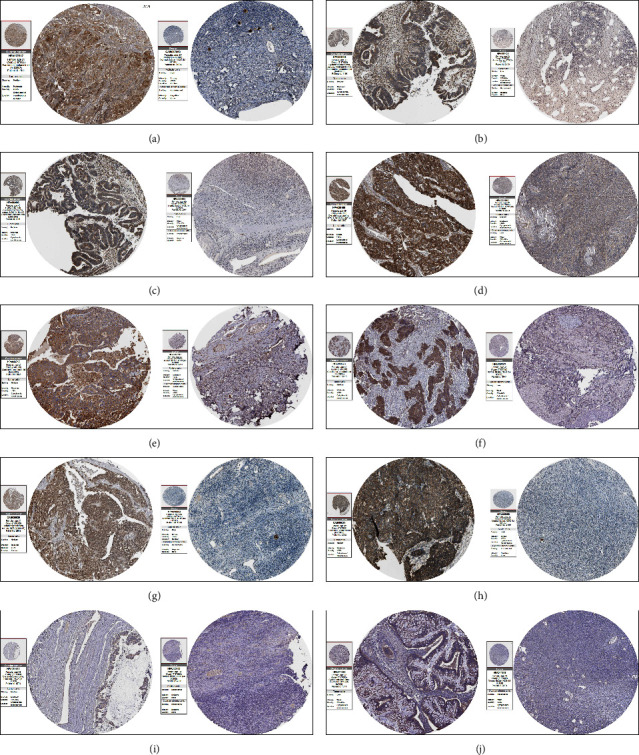
Expression of hub genes at the protein level in ovarian tissue. (a) TCP1; (b) RPS6; (c) RPL12; (d) CTNNB1; (e) RPL5; (f) RPL7; (g) HSPA4; (h) CCT5; (i) RPS2; and (j) CALM3.

The UALCAN database was used to determine the expression variations in hub genes in ovarian cancer. The expression of TCP1 (*p* value = 4.00*E* − 2), RPS6 (*p* value = 5.90*E* − 12), RPL12 (*p* value = 6.14*E* − 12), RPL5 (*p* value = 1.13*E* − 13), RPL7 (*p* value = 3.73*E* − 05), HSPA4 (*p* value = 1.05*E* − 17), CCT5 (*p* value = 3.02*E* − 04), and RPS2 (*p* value = 5.00*E* − 13) was found to be higher in OV as compared to normal tissues, whereas no difference was observed in CTNNB1 (Figure 4). In different stages of ovarian cancer, higher expression of TCP1 (normal vs. stage 3, *p* value = 3.00*E* − 2), RPS6 (normal vs. stage 3, *p* value = 6.81*E* − 08), RPL12 (normal vs. stage 3, *p* value = 3.33*E* − 07), RPL5 (normal vs. stage 3, *p* value = 3.35*E* − 08), RPL7 (normal vs. stage 3, *p* value = 1.81*E* − 04 and normal vs. stage 4, *p* value = 4.77*E* − 08), HSPA4 (normal vs. stage 3, *p* value = 1.17*E* − 05), CCT5 (normal vs. stage 3, *p* value = 5.00*e* − 2), and RPS2 (normal vs. stage 1, *p* value = 3.01*E* − 10; normal vs. stage 3, *p* value = 1.52*E* − 07; and normal vs. stage 4, *p* value = 4.89*E* − 02) was observed (Figure 5). There was no difference in the expression of CTNNB1 in normal and different cancer stages. The immunohistochemistry data show that expression of TCP1, RPS6, RPL12, CTNNB1, RPL5, RPL7, HSPA4, CCT5, RPS2, and CALM3 was significantly higher in ovarian cancer tissue samples compared to normals, suggesting a substantial role of these proteins in tumorigenesis (Figure 6).

### 3.5. Prognostic Value of Hub Genes in Ovarian Cancer

**Figure 7:**
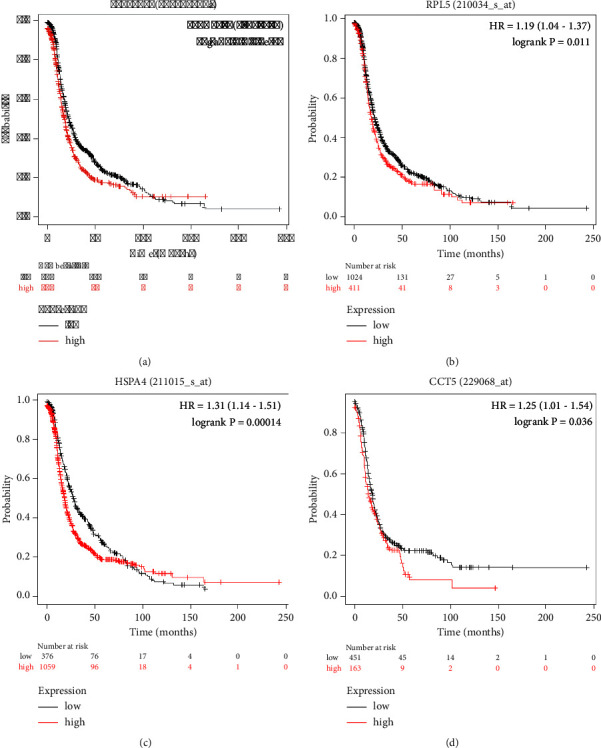
Prognostic value of hub genes in ovarian cancer. The Kaplan–Meier plotter database was used to identify the statistically significant prognostic value of hub genes in ovarian cancer. (a) TCP1; (b) RPL5; (c) HSPA4; and (d) CCT5. (*p* value ≤ 0.05).

The Kaplan–Meier plot analysis revealed that higher expressions of TCP1 (HR = 1.3 (1.14–1.48), *p* value = 5.60*E* − 05), RPL5 (HR = 1.19 (1.04–1.37), *p* value = 1.00*E* − 2), HSPA4 (HR = 1.31 (1.14–1.51), *p* value = 1.40*E* − 4), and CCT5 (HR = 1.25 (1.01–1.54), *p* value = 3.00*E* − 2) were linked to poor outcome in ovarian cancer patients (Figure 7). Notably, expression levels of RPS6, RPL12, CTNNB1, RPL7, RPS2, and CALM3 did not have any impact on the survival of ovarian cancer patients.

### 3.6. Genetic Alterations of Hub Genes

**Figure 8:**
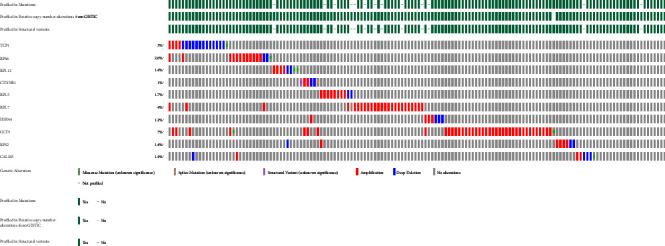
The genetic alterations and their frequency in hub genes in ovarian cancer. The mutational burden was determined using the cBioPortal database.

Comparative analysis of hub genes to identify genetic mutations in ovarian cancer revealed that the TCP1 gene has a mutational burden in 3% of cases, RPS6 in 2.6%, RPL2 in 1.2%, CTNNB1 in 1%, RPL5 in 1.5%, RPL7 in 4%, HSPA4 in 1.2%, CCT5 in 7%, RPS2 in 1.4%, and CALM3 in 1.4% of cases of ovarian cancer. Deep deletion mainly occurred in the TCP1 gene, while amplification was the most significant mutation in the rest of the genes (Figure 8).

### 3.7. Correlation between the Expressions of Hub Genes and Immune Infiltration in Ovarian Cancer

**Figure 9:**
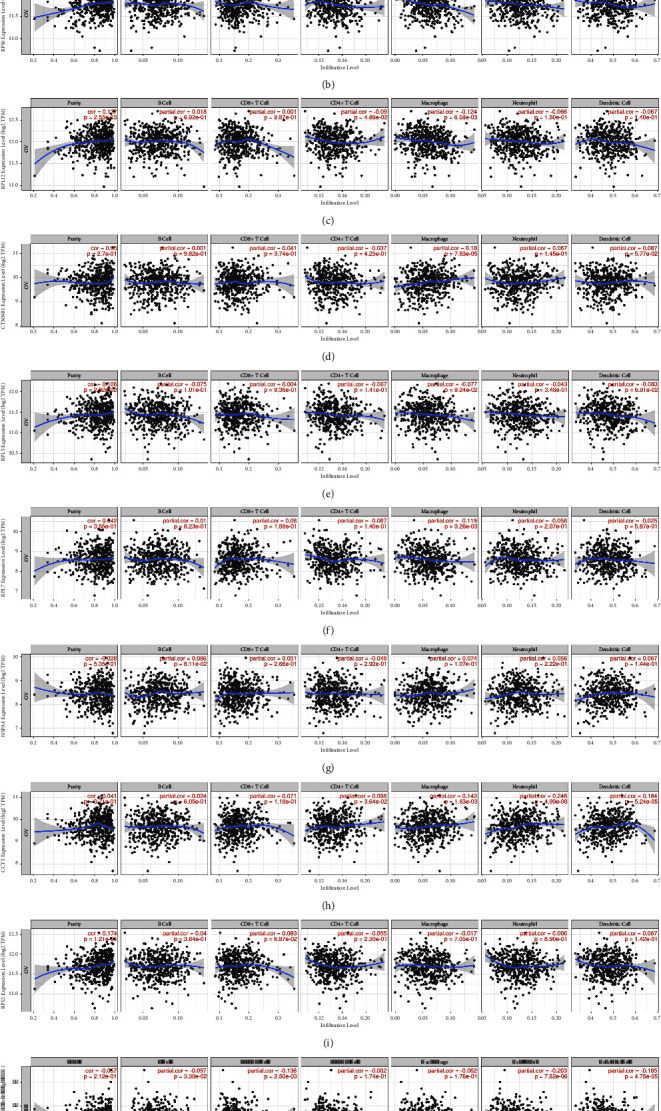
The relationship between the expression of hub genes and immune infiltrating cells in ovarian cancer. The correlation between the expression of the hub genes and immune infiltration level in ovarian cancer was deduced by the TIMER database. (a) TCP1; (b) RPS6; (c) RPL12; (d) CTNNB1; (e) RPL5; (f) RPL7; (g) HSPA4; (h) CCT5; (i) RPS2; and (j) CALM3.

TIICs form a significant part of the complex microenvironment, which regulates the development and progression of different cancers [37]. Their quantity and activity are considered significant prognostic criteria for survival in ovarian cancer [3]. Thus, we investigated the relationship between the expression of hub genes and immune infiltration in ovarian cancer. After the correlation adjustment by purity, the results indicated that TCP1 expression has significant positive correlations with macrophage (*r* = 0.224, *p* value = 7.36*E* − 07), neutrophil (*r* = 0.177, *p* value = 9.12*E* − 05), and dendritic cells (*r* = 0.151, *p* value = 8.8*E* − 4). RPS6 expression has negative correlation with macrophage (*r* = −0.161, *p* value = 3.85*E* − 04). RPL12 expression has negative correlation with CD4^+^ T cells (*r* = 0.09, *p* value = 4.86*E* − 02) and macrophages (*r* = 0.124, *p* value = 6.58*E* − 03). CTNNB1 expression has positive correlation with macrophages (*r* = 0.18, *p* value = 7.63*E* − 05). RPL7 expression has negative correlation with macrophages (*r* = 0.116, *p* value = 9.2*E* − 03). CCT5 expression has a positive correlation with CD4^+^ T cells (*r* = 0.09, *p* value = 3.64*E* − 02), macrophage (*r* = 0.143, *p* value = 1.63*E* − 03), neutrophil (*r* = 0.246, *p* value = 4.99*E* − 08), and dendritic cells (*r* = 0.184, *p* value = 5.24*E* − 05). CALM3 expression has a negative correlation with CD8^+^ T cells (*r* = −0.138, *p* value = 2.50*E* − 03), neutrophil (*r* = −0.203, *p* value = 7.62*E* − 06), and dendritic cells (*r* = −0185, *p* value = 4.75*E* − 05). RPL5, HSPA4, and RPS2 expressions did not show any significant correlation with immune infiltration (Figure 9).

## 4. Discussion

The prognosis for recurrent patients and those with advanced forms of ovarian cancer remains grim despite substantial advancements in diagnostic, surgical, and therapeutic procedures. Thus, it is essential to understand the complex molecular interplays and mechanisms underlying the initiation and progression of ovarian cancer in order to improve the survival rates of such patients. This will allow for the development of more specific diagnostic biomarkers, which identify patients with a high possibility of rapid tumor growth and progression. The human fibroblast growth factor (FGF) family consists of 22 proteins, which regulate numerous physiological processes in both developing and adult organisms [38]. These FGF proteins are secreted glycoproteins, often bound to the cell surface and the extracellular matrix by heparan sulphate proteoglycans (HPSGs). Fundamental developmental pathways, including mesoderm patterning in the early embryo and the development of numerous organ systems, are regulated by FGF proteins, which signal through FGFRs [39]. FGF8, an important member of the FGF family, was originally purified from androgen-stimulated SC-3 cells and identified as the first hormone-induced autocrine growth factor [40]. It has a very limited expression in normal adult tissues, being expressed in only specific cells associated with oogenesis and spermatogenesis; however, it is broadly expressed in developing tissues in a controlled way [41]. Some studies have shown the role of FGF8 in the initiation, progression, and development of malignancies and associated its abnormal expression with hormone-responsive cancers, including breast and prostate cancer [16, 42]. In addition, several types of cancer cells acquire an aggressively altered phenotype due to the overexpression of FGF8. A study showed that overexpressed FGF8 in mouse mammary cancer cells led to EMT, anchorage-independent growth, and faster tumor growth in vivo [43]. In one of our recent studies, we observed that FGF8 silencing reduced various tumor-promoting properties of ovarian cancer cells (SKOV3), including cell survival, cell adhesion to the extracellular matrix, migration, and adhesion, signifying that FGF8 plays a vital role in the progression of ovarian cancer [25]. Therefore, this study was designed to gain more functional insights into FGF8 expression and understand the FGF8-linked pathways and mechanisms associated with the pathogenesis of ovarian cancer.

In this study, high-throughput quantitative and differential proteomics analysis was carried out in FGF8-silenced cells to delve deeper into the functions of FGF8 and related molecular interactions and pathways, contributing to the progression of ovarian cancer. Out of 418 DEPs identified in the FGF8-silenced ovarian cancer cells, 414 proteins were downregulated, and only four proteins were upregulated. Many of the downregulated proteins were previously reported to play an important role in cancer progression, including SLC7A5, FHL2, SPATS2L, DAD1, TPD52, MCM2, PKP3, CRK, LPP, PSAT1, PKP3, TMX1, and SF3B4. For example, SLC7A5 expression increases as cancer progresses, resulting in higher levels of expression in high-grade tumors and metastasis. Furthermore, SLC7A5 supplies essential amino acids to cancer cells and, thus, plays an important role in cancer-associated reprogrammed metabolic networks [44]. Similarly, FHL2 is also involved in ovarian cancer growth. The knockdown of FHL2 resulted in the reduction of cell growth and cell viability, obstruction of cell cycle progression, inhibition of cell migration through downregulation of AKT expression, and upregulation of apoptosis-related proteins in EOC [45]. An upregulated expression of SPATS2L, a cytoplasmic RNA-binding protein linked to tumorigenicity in several cancers, has been associated with poor prognosis in esophageal squamous cell carcinoma and liver cancer [46]. Another downregulated protein DAD1 is a negative regulator of apoptosis, and tumor cells may benefit from its ability to prevent apoptosis from continuing to grow indefinitely. The expression of DAD1 in ovarian cancer was reported in OVCAR3 cells following the treatment of cisplatin, indicating that over-expression of DAD1 contributed to the development of cisplatin resistance in ovarian cancer [47]. Several studies have reported upregulation of TPD52 in all four major histological types of EOC, concluding that it is a potential early tumor marker for detection as well as a plausible therapeutic target [48]. MCM2 knockdown significantly reduced cell proliferation in ovarian cancer cells and reported a higher expression of p53 and *γ*-H2A histone family member *X* in carboplatin-treated cells, suggesting a role of MCM2 in carboplatin resistance [49]. Recent studies have identified the role of CRK in proliferation, invasion, dissemination, and an altered pattern of mucus production and secretion in ovarian cancer cells, suggesting its role in focal adhesion and invasion in ovarian cancer [50]. In ovarian cancer, a function of LPP and PSAT1 was discovered in endothelial cell motility through focal adhesion and stress fibre production, as well as in cell proliferation and metastasis [51, 52]. Immunohistochemistry and gene knockdown studies showed OV expression of PKP3 and repression of apoptosis through silencing of death receptors (DRs) 4/5 by C-terminal-binding protein 2 in ovarian cancer cell lines, suggesting a role of these two proteins in the disease's progression [53, 54]. Recent research has revealed the predictive significance of TMX1 and SF3B4 in ovarian cancer, as well as their roles in proliferation and motility, respectively [55, 56]. In FGF8-silenced ovarian cancer cells, the downregulation of several proteins directly linked to the growth of cancer implies that FGF8 plays a significant role in the development of ovarian cancer.

Proteins play a vital role in various biological processes, and their interactions are crucial for the correct functioning of the cell. Thus, PPI network analysis provides key insights into the organization and regulation of cellular pathways and can help identify key proteins and pathways involved in disease development. The STRING database, comprising both known and predicted PPIs, is a widely used platform for constructing PPI networks [57, 58]. STRING analysis of DEPs in FGF8-silenced cells generated a dense PPI network, showing that the proteins in the network are interconnected with each other. It suggests that a deep and sophisticated web of interactions exists between these proteins. Further, gene enrichment and pathway analysis revealed that several GO terms and KEGG pathways that are known to aid in cancer progression were associated with downregulated proteins in FGF8-silenced ovarian cancer cells. The specific biological processes identified were translation, protein stabilization, protein transport, proteasomal protein catabolic process, and protein UFMylation. Cancer cells utilize various mechanisms of protein stabilization to prevent the degradation of oncogenic proteins, which contribute to tumor development and progression [59]. The protein stabilization mechanisms might include inhibition of autophagy and proteasomal degradation, post-translational modification, mutations, and chaperone-mediated protein modification. Interestingly, the downregulation of 17 proteins linked to protein stabilization, including Ras-related protein Rab-21 (RAB21), flotillin-1 (FLOT1), interferon-inducible double-stranded RNA-dependent protein kinase activator A (PRKRA), RPAP3, desmoglein-1 (DSG1), BAG family molecular chaperone regulator 3 (BAG3), 60S ribosomal protein L5 (RPL5), profilin-2 (PFN2), and FLNA in FGF8-silenced cells, accentuates the role of FGF8 in ovarian cancer progression (Supplementary file 3). RAB21 helps tumor-associated fibroblasts to invade squamous carcinoma cells. Ge et al. have reported a significant overexpression of RAB21 in glioma cell lines [60]. They further showed that the knockdown of Rab21 using specific siRNA drastically inhibited cell growth and induced cell apoptosis, demonstrating that Rab21 might act as an oncogene [60]. Another protein, FLOT1, has also been linked to many cancer types and is known to promote tumorigenesis and cancer progression, which leads to poor prognosis [61]. Similarly, other DEPs associated with the protein stabilization process are also involved in cancer progression [62–66]. A reduced expression of such proteins in FGF8 knockdown cells strengthens the hypothesis that FGF8 is involved in the growth of ovarian cancer and that all these proteins function as an interconnected network.

Next, the PPI network of DEPs was transferred to Cytoscape to identify hub genes. Eight out of the top 10 hub genes encode two types of proteins: ribosomal proteins (RPS6, RPL12, RPL5, RPL7, and RPS2) and chaperones (TCP1, HSPA4, and CCT5). The other two genes are CTNNB1 and CALM3, which encode beta-catenin and calmodulin 3 proteins, respectively. Beta-catenin is one of the constituents of a protein complex that makes up adherens junctions. It aids in the production and maintenance of epithelial cells through regulation of cell growth and adhesion between cells. Also, mutations in the CTNNB1 gene have been reported in some cancers, including ovarian cancer [67–70]. On the other hand, calmodulin 3, a calcium-binding protein, functions as an enzymatic cofactor and regulates the cell cycle and cytokinesis. It plays an important role in tumor cell migration, invasiveness, and metastasis [71]. Notably, these hub genes are linked to poor prognosis in cancer patients.

The expression levels of the hub genes and the systematic prognostic landscapes in ovarian cancer were explored using the UALCAN and the Human Protein Atlas database. Ovarian cancer tissue samples exhibit higher expression of TCP1, RPS6, RPL12, RPL5, RPL7, HSPA4, CCT5, and RPS2 in comparison to normal tissues. Interestingly, the expression of all these genes increases with an increasing ovarian cancer stage. In “The Protein Atlas database” data, we observed that hub genes were highly expressed in ovarian cancer as compared to healthy controls. Furthermore, analysis of data from the Kaplan–Meier plot showed a correlation between higher expression of TCP1, RPL5, HSPA4, and CCT5 and poor prognosis in ovarian cancer.

According to recent developments in the sequencing and genotyping of cancer tissues at the genome level, tens of thousands of somatic mutations are found in each malignancy. These comprise a broad spectrum of genetic changes, including deletions, insertions, copy-number alterations, rearrangements, whole-chromosome duplications/deletions, and loss of heterozygosity (LOH) events. They also include epigenetic changes, inheritable changes in the cell state, and single-nucleotide substitutions [72–74]. The effectiveness of chemotherapy, the course of the disease, and the overall survival rate are all impacted by total mutational burden. Therefore, a comparative analysis was carried out to identify the genetic mutations in hub genes in cases of ovarian cancer. Analysis of genetic alterations showed that “amplification” was the most significant genetic alteration in RPS6, RPL12, CTNNB1, RPL5, RPL7, HSPA4, CCT5, RPS2, and CALM3, while it was “deep deletion” in the TCP1 gene.

The TME is essential for the development of tumors, therapeutic response, and patient outcomes. The TME is thought to be an active driver of cancer progression rather than only a passive observer. Although the TME's makeup varies depending on the kind of tumor, blood vessels, stromal cells, immune cells, and extracellular matrix are common constituents [37, 75, 76]. Numerous studies have discovered immune cell signatures in the TME of ovarian cancer, highlighting the significance of immune cells in the TME for prognosis and diagnosis [77–80]. The association between the expression of hub genes and different levels of immune infiltration in ovarian cancer is another significant finding of this study. A significant positive correlation was observed between the expression of hub genes and T4^+^ T cells, macrophage, neutrophil, and dendritic cell infiltration levels in ovarian cancer. Depending on the makeup of immune cells and their phenotypic states, the infiltrated immune cells may inhibit or accelerate the development of cancer. For instance, M2 macrophages function as tumor-associated macrophages (TAMs) to disrupt inflammatory and adaptive immune circuits and promote tumor growth [81]. In advanced-stage ovarian cancer, TAMs are the immune cells that are most prevalent and promote the growth, invasion, angiogenesis, metastasis, and drug resistance of tumors [82]. Therefore, the relationship between the expression of hub genes of the FGF8 protein network and immune cell marker genes suggests that FGF8 is implicated in modulating tumor immunology in ovarian cancer.

Upon correlating our study's findings with the existing literature, we observed that silencing FGF8 in ovarian cancer cells resulted in the downregulation of several proteins associated with cancer progression, including SLC7A5, FHL2, SPATS2L, and DAD1. Additionally, protein-protein interaction (PPI) analysis unveiled a complex network of proteins, with hub genes in this network displaying higher expression in ovarian cancer tissues, correlating with a poorer prognosis. Thus, the molecular interplay revealed by FGF8 silencing suggests a significant role for FGF8 in the development and progression of ovarian cancer.

The limitation of this study is that the experiments were conducted using only a single cell line. While all experiments were performed in triplicate, further validation in additional cell lines and animal models would provide more comprehensive and informative results.

## 5. Conclusion

In the FGF8-silenced ovarian cancer cells, differential proteomics found downregulated expression of many proteins connected to the cancer growth, indicating the significant involvement of FGF8 in the development and spread of ovarian cancer. Additionally, elevated expression of related hub genes correlates with poor prognosis and higher immune infiltration in ovarian cancer patients. Thus, FGF8 might serve as a possible therapeutic target for ovarian cancer, according to the findings of this study. We suggest further exploratory studies pertaining to validation of identified hub genes, which can aid in identifying novel therapeutic targets.

## Figures and Tables

**Table 1:** List of differentially expressed proteins in FGF8-silenced SKOV3 cells (FC ≥ 2, *p* value ≤ 0.05).

Accession	Protein name	Fold change	Log2 (FC)	*T*-test (*p* value)
*Top 20 downregulated proteins in FGF8-silenced cells*				
Q14192	Four and a half LIM domains protein 2	0.092226	−3.4387	0.0057078
Q15758	Neutral amino acid transporter B(0)	0.11727	−3.0921	0.0018211
O43852	Calumenin	0.13294	−2.9112	0.045015
A0MZ66	Shootin-1	0.13587	−2.8797	0.0049367
Q9NUQ6	SPATS2-like protein	0.1427	−2.809	0.00074072
P61803	Dolichyl-diphosphooligosaccharide—protein glycosyltransferase subunit DAD1	0.15084	−2.7289	0.035274
Q12965	Unconventional myosin-Ie	0.15167	−2.721	0.047315
P23634	Plasma membrane calcium-transporting ATPase 4	0.17416	−2.5215	0.01175
P55327	Tumor protein D52	0.17816	−2.4887	0.044933
Q15021	Condensin complex subunit 1	0.18254	−2.4537	0.012592
Q9H6T3	RNA polymerase II-associated protein 3	0.1872	−2.4173	0.021988
Q01650	Large neutral amino acids transporter small subunit 1	0.1886	−2.4066	0.0001025
Q9BWD1	Acetyl-CoA acetyltransferase, cytosolic	0.203	−2.3004	0.011556
O14672	Disintegrin and metalloproteinase domain-containing protein 10	0.20479	−2.2878	0.005573
O96005	Cleft lip and palate transmembrane protein 1	0.20951	−2.2549	0.019922
P49736	DNA replication licensing factor MCM2	0.21432	−2.2222	0.0034988
Q9H0U3	Magnesium transporter protein 1	0.21638	−2.2083	0.045159
P19387	DNA-directed RNA polymerase II subunit RPB3	0.21773	−2.1994	0.0084209
A0FGR8	Extended synaptotagmin-2	0.21851	−2.1942	0.0014046
Q96KG9	N-terminal kinase-like protein	0.21884	−2.1921	0.025682

*Upregulated proteins in FGF8-silenced cells*				
D6RBQ6	Ubiquitin carboxyl-terminal hydrolase 17-like protein 17	4.9764	2.3151	0.038838
P28799	Progranulin	2.4522	1.2941	0.016237
P35914	Hydroxymethylglutaryl-CoA lyase, mitochondrial	2.2947	1.1983	0.048561
P04179	Superoxide dismutase [Mn], mitochondrial	2.2345	1.1599	0.041962

## Data Availability

The data supporting the findings of this study are available within the article and its supplementary materials.

## Supplementary Materials

Supplementary Materials Supplementary Figure 1: venn diagram of proteins identified in FGF8-silenced and FGF8-expressing ovarian cancer cells (SKOV3). Supplementary File 1: proteins identified in ovarian cancer cells (SKOV3). Supplementary File 2: differentially expressed proteins identified by volcano plot analysis. Supplementary File 3: enrichment of GO terms and pathways among downregulated proteins in FGF8-silenced ovarian cancer cells (SKOV3).
