# Supplementary material for: Proteomics and Bioinformatics Investigations Link Overexpression of FGF8 and Associated Hub Genes to the Progression of Ovarian Cancer and Poor Prognosis
Source: Biochem Res Int. 2024 Sep 13;2024:4288753. doi: 10.1155/2024/4288753 (PMC11415250; doi:10.1155/2024/4288753)
Supplement: Supplementary Materials — Supplementary Figure 1: venn diagram of proteins identified in FGF8-silenced and FGF8-expressing ovarian cancer cells (SKOV3). Supplementary File 1: proteins identified in ovarian cancer cells (SKOV3). Supplementary File 2: differentially expressed proteins identified by volcano plot analysis. Supplementary File 3: enrichment of GO terms and pathways among downregulated proteins in FGF8-silenced ovarian cancer cells (SKOV3). [file 4288753.f1.zip › Supplementary figure 1.pdf]

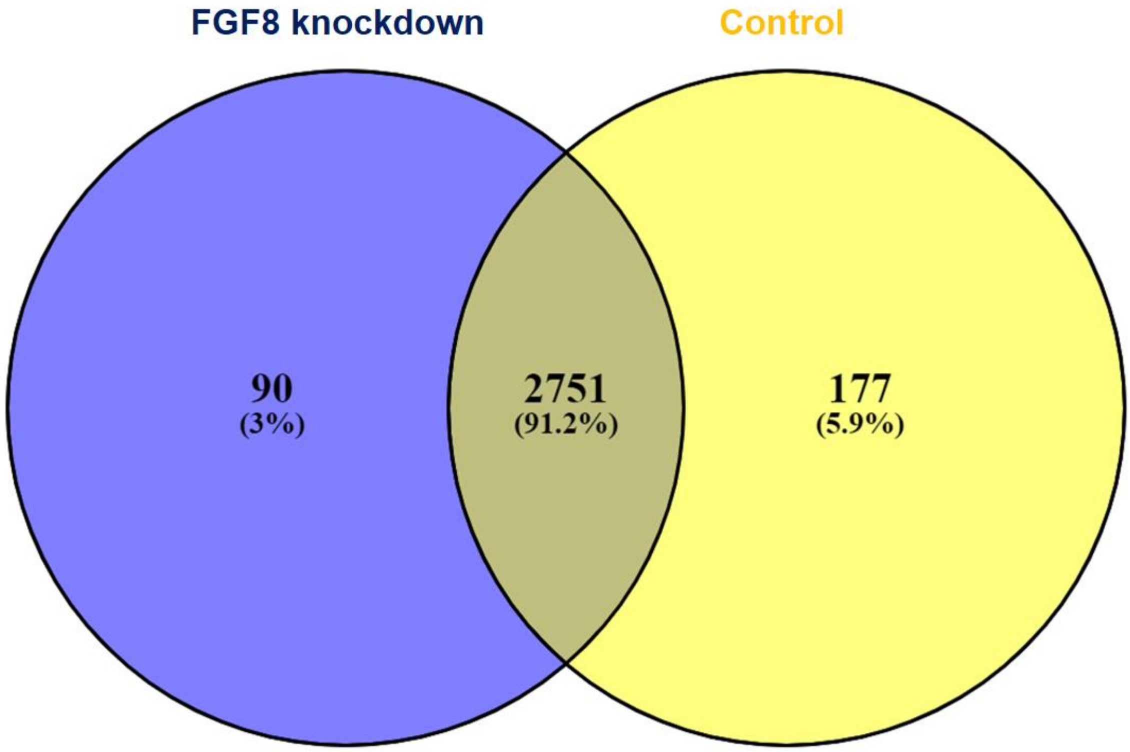

Supplementary figure 1: Venn diagram of proteins identified in FGF8-silenced and FGF8-expressing ovarian cancer cells (SKOV3).
