# Supplementary material for: Proteomics and Bioinformatics Investigations Link Overexpression of FGF8 and Associated Hub Genes to the Progression of Ovarian Cancer and Poor Prognosis
Source: Biochem Res Int. 2024 Sep 13;2024:4288753. doi: 10.1155/2024/4288753 (PMC11415250; doi:10.1155/2024/4288753)
Supplement: Supplementary Materials — Supplementary Figure 1: venn diagram of proteins identified in FGF8-silenced and FGF8-expressing ovarian cancer cells (SKOV3). Supplementary File 1: proteins identified in ovarian cancer cells (SKOV3). Supplementary File 2: differentially expressed proteins identified by volcano plot analysis. Supplementary File 3: enrichment of GO terms and pathways among downregulated proteins in FGF8-silenced ovarian cancer cells (SKOV3). [file 4288753.f1.zip › Supplementary file 1.pdf]

Supplementary file 1: Proteins identified in ovarian cancer cells (SKOV3).

Accession	Description	MW [kDa]	calc. pI	Gene Symbol
Q09666	Neuroblast differentiation-associated protein AHNAK OS=Homo sapiens OX=9606 GN=AHNAK PE=1 SV=2	628.7	6.15	AHNAK
Q15149	Plectin OS=Homo sapiens OX=9606 GN=PLEC PE=1 SV=3	531.5	5.96	PLEC
P21333	Filamin-A OS=Homo sapiens OX=9606 GN=FLNA PE=1 SV=4	280.6	6.06	FLNA
P35579	Myosin-9 OS=Homo sapiens OX=9606 GN=MYH9 PE=1 SV=4	226.4	5.6	MYH9
Q14204	Cytoplasmic dynein 1 heavy chain 1 OS=Homo sapiens OX=9606 GN=DYNC1H1 PE=1 SV=5	532.1	6.4	DYNC1H1
Q9Y490	Talin-1 OS=Homo sapiens OX=9606 GN=TLN1 PE=1 SV=3	269.6	6.07	TLN1
O43707	Alpha-actinin-4 OS=Homo sapiens OX=9606 GN=ACTN4 PE=1 SV=2	104.8	5.44	ACTN4
O75369	Filamin-B OS=Homo sapiens OX=9606 GN=FLNB PE=1 SV=2	278	5.73	FLNB
Q8IVF2	Protein AHNAK2 OS=Homo sapiens OX=9606 GN=AHNAK2 PE=1 SV=2	616.2	5.36	AHNAK2
Q00610	Clathrin heavy chain 1 OS=Homo sapiens OX=9606 GN=CLTC PE=1 SV=5	191.5	5.69	CLTC
P14618	Pyruvate kinase PKM OS=Homo sapiens OX=9606 GN=PKM PE=1 SV=4	57.9	7.84	PKM
P08670	Vimentin OS=Homo sapiens OX=9606 GN=VIM PE=1 SV=4	53.6	5.12	VIM
Q13813	Spectrin alpha chain, non-erythrocytic 1 OS=Homo sapiens OX=9606 GN=SPTAN1 PE=1 SV=3	284.4	5.35	SPTAN1
P78527	DNA-dependent protein kinase catalytic subunit OS=Homo sapiens OX=9606 GN=PRKDC PE=1 SV=3	468.8	7.12	PRKDC
P12814	Alpha-actinin-1 OS=Homo sapiens OX=9606 GN=ACTN1 PE=1 SV=2	103	5.41	ACTN1

P63261	Actin, cytoplasmic 2 OS=Homo sapiens OX=9606 GN=ACTG1 PE=1 SV=1	41.8	5.48	ACTG1
P06733	Alpha-enolase OS=Homo sapiens OX=9606 GN=ENO1 PE=1 SV=2	47.1	7.39	ENO1
P04264	Keratin, type II cytoskeletal 1 OS=Homo sapiens OX=9606 GN=KRT1 PE=1 SV=6	66	8.12	KRT1
Q01082	Spectrin beta chain, non-erythrocytic 1 OS=Homo sapiens OX=9606 GN=SPTBN1 PE=1 SV=2	274.4	5.57	SPTBN1
P60709	Actin, cytoplasmic 1 OS=Homo sapiens OX=9606 GN=ACTB PE=1 SV=1	41.7	5.48	ACTB
P55072	Transitional endoplasmic reticulum ATPase OS=Homo sapiens OX=9606 GN=VCP PE=1 SV=4	89.3	5.26	VCP
P68371	Tubulin beta-4B chain OS=Homo sapiens OX=9606 GN=TUBB4B PE=1 SV=1	49.8	4.89	TUBB4B
P10809	60 kDa heat shock protein, mitochondrial OS=Homo sapiens OX=9606 GN=HSPD1 PE=1 SV=2	61	5.87	HSPD1
P07900	Heat shock protein HSP 90-alpha OS=Homo sapiens OX=9606 GN=HSP90AA1 PE=1 SV=5	84.6	5.02	HSP90AA1
P35908	Keratin, type II cytoskeletal 2 epidermal OS=Homo sapiens OX=9606 GN=KRT2 PE=1 SV=2	65.4	8	KRT2
P07437	Tubulin beta chain OS=Homo sapiens OX=9606 GN=TUBB PE=1 SV=2	49.6	4.89	TUBB
P08238	Heat shock protein HSP 90-beta OS=Homo sapiens OX=9606 GN=HSP90AB1 PE=1 SV=4	83.2	5.03	HSP90AB1
P04406	Glyceraldehyde-3-phosphate dehydrogenase OS=Homo sapiens OX=9606 GN=GAPDH PE=1 SV=3	36	8.46	GAPDH
P13645	Keratin, type I cytoskeletal 10 OS=Homo sapiens OX=9606 GN=KRT10 PE=1 SV=6	58.8	5.21	KRT10
P14625	Endoplasmin OS=Homo sapiens OX=9606 GN=HSP90B1 PE=1 SV=1	92.4	4.84	HSP90B1
P11142	Heat shock cognate 71 kDa protein OS=Homo sapiens OX=9606 GN=HSPA8 PE=1 SV=1	70.9	5.52	HSPA8

P49327	Fatty acid synthase OS=Homo sapiens OX=9606 GN=FASN PE=1 SV=3	273.3	6.44	FASN
P13639	Elongation factor 2 OS=Homo sapiens OX=9606 GN=EEF2 PE=1 SV=4	95.3	6.83	EEF2
P29401	Transketolase OS=Homo sapiens OX=9606 GN=TKT PE=1 SV=3	67.8	7.66	TKT
P15311	Ezrin OS=Homo sapiens OX=9606 GN=EZR PE=1 SV=4	69.4	6.27	EZR
P18206	Vinculin OS=Homo sapiens OX=9606 GN=VCL PE=1 SV=4	123.7	5.66	VCL
Q13885	Tubulin beta-2A chain OS=Homo sapiens OX=9606 GN=TUBB2A PE=1 SV=1	49.9	4.89	TUBB2A
P46940	Ras GTPase-activating-like protein IQGAP1 OS=Homo sapiens OX=9606 GN=IQGAP1 PE=1 SV=1	189.1	6.48	IQGAP1
P00338	L-lactate dehydrogenase A chain OS=Homo sapiens OX=9606 GN=LDHA PE=1 SV=2	36.7	8.27	LDHA
P26038	Moesin OS=Homo sapiens OX=9606 GN=MSN PE=1 SV=3	67.8	6.4	MSN
P35527	Keratin, type I cytoskeletal 9 OS=Homo sapiens OX=9606 GN=KRT9 PE=1 SV=3	62	5.24	KRT9
P06576	ATP synthase subunit beta, mitochondrial OS=Homo sapiens OX=9606 GN=ATP5F1B PE=1 SV=3	56.5	5.4	ATP5F1B
P11021	Endoplasmic reticulum chaperone BiP OS=Homo sapiens OX=9606 GN=HSPA5 PE=1 SV=2	72.3	5.16	HSPA5
P07355	Annexin A2 OS=Homo sapiens OX=9606 GN=ANXA2 PE=1 SV=2	38.6	7.75	ANXA2
P04075	Fructose-bisphosphate aldolase A OS=Homo sapiens OX=9606 GN=ALDOA PE=1 SV=2	39.4	8.09	ALDOA
Q14764	Major vault protein OS=Homo sapiens OX=9606 GN=MVP PE=1 SV=4	99.3	5.48	MVP
P02545	Prelamin-A/C OS=Homo sapiens OX=9606 GN=LMNA PE=1 SV=1	74.1	7.02	LMNA
Q14697	Neutral alpha-glucosidase AB OS=Homo sapiens OX=9606 GN=GANAB PE=1 SV=3	106.8	6.14	GANAB

P04350	Tubulin beta-4A chain OS=Homo sapiens OX=9606 GN=TUBB4A PE=1 SV=2	49.6	4.88	TUBB4A
Q71U36	Tubulin alpha-1A chain OS=Homo sapiens OX=9606 GN=TUBA1A PE=1 SV=1	50.1	5.06	TUBA1A
P04083	Annexin A1 OS=Homo sapiens OX=9606 GN=ANXA1 PE=1 SV=2	38.7	7.02	ANXA1
Q9BQE3	Tubulin alpha-1C chain OS=Homo sapiens OX=9606 GN=TUBA1C PE=1 SV=1	49.9	5.1	TUBA1C
P07237	Protein disulfide-isomerase OS=Homo sapiens OX=9606 GN=P4HB PE=1 SV=3	57.1	4.87	P4HB
Q13509	Tubulin beta-3 chain OS=Homo sapiens OX=9606 GN=TUBB3 PE=1 SV=2	50.4	4.93	TUBB3
O00429	Dynamin-1-like protein OS=Homo sapiens OX=9606 GN=DNM1L PE=1 SV=2	81.8	6.81	DNM1L
P22314	Ubiquitin-like modifier-activating enzyme 1 OS=Homo sapiens OX=9606 GN=UBA1 PE=1 SV=3	117.8	5.76	UBA1
P11216	Glycogen phosphorylase, brain form OS=Homo sapiens OX=9606 GN=PYGB PE=1 SV=5	96.6	6.86	PYGB
P04626	Receptor tyrosine-protein kinase erbB-2 OS=Homo sapiens OX=9606 GN=ERBB2 PE=1 SV=1	137.8	5.91	ERBB2
P05787	Keratin, type II cytoskeletal 8 OS=Homo sapiens OX=9606 GN=KRT8 PE=1 SV=7	53.7	5.59	KRT8
P68366	Tubulin alpha-4A chain OS=Homo sapiens OX=9606 GN=TUBA4A PE=1 SV=1	49.9	5.06	TUBA4A
P68104	Elongation factor 1-alpha 1 OS=Homo sapiens OX=9606 GN=EEF1A1 PE=1 SV=1	50.1	9.01	EEF1A1
P0DMV9	Heat shock 70 kDa protein 1B OS=Homo sapiens OX=9606 GN=HSPA1B PE=1 SV=1	70	5.66	HSPA1B
P42704	Leucine-rich PPR motif-containing protein, mitochondrial OS=Homo sapiens OX=9606 GN=LRPPRC PE=1 SV=3	157.8	6.13	LRPPRC
P00558	Phosphoglycerate kinase 1 OS=Homo sapiens OX=9606 GN=PGK1 PE=1 SV=3	44.6	8.1	PGK1

P08758	Annexin A5 OS=Homo sapiens OX=9606 GN=ANXA5 PE=1 SV=2	35.9	5.05	ANXA5
P38646	Stress-70 protein, mitochondrial OS=Homo sapiens OX=9606 GN=HSPA9 PE=1 SV=2	73.6	6.16	HSPA9
P78371	T-complex protein 1 subunit beta OS=Homo sapiens OX=9606 GN=CCT2 PE=1 SV=4	57.5	6.46	CCT2
Q99715	Collagen alpha-1(XII) chain OS=Homo sapiens OX=9606 GN=COL12A1 PE=1 SV=2	332.9	5.53	COL12A1
P12956	X-ray repair cross-complementing protein 6 OS=Homo sapiens OX=9606 GN=XRCC6 PE=1 SV=2	69.8	6.64	XRCC6
P35580	Myosin-10 OS=Homo sapiens OX=9606 GN=MYH10 PE=1 SV=3	228.9	5.54	MYH10
Q01813	ATP-dependent 6-phosphofructokinase, platelet type OS=Homo sapiens OX=9606 GN=PFKP PE=1 SV=2	85.5	7.55	PFKP
P50395	Rab GDP dissociation inhibitor beta OS=Homo sapiens OX=9606 GN=GDI2 PE=1 SV=2	50.6	6.47	GDI2
Q92616	eIF-2-alpha kinase activator GCN1 OS=Homo sapiens OX=9606 GN=GCN1 PE=1 SV=6	292.6	7.47	
Q08211	ATP-dependent RNA helicase A OS=Homo sapiens OX=9606 GN=DHX9 PE=1 SV=4	140.9	6.84	DHX9
P49588	Alanine--tRNA ligase, cytoplasmic OS=Homo sapiens OX=9606 GN=AARS1 PE=1 SV=2	106.7	5.53	AARS1
P53396	ATP-citrate synthase OS=Homo sapiens OX=9606 GN=ACLY PE=1 SV=3	120.8	7.33	ACLY
P68032	Actin, alpha cardiac muscle 1 OS=Homo sapiens OX=9606 GN=ACTC1 PE=1 SV=1	42	5.39	ACTC1
Q9BUF5	Tubulin beta-6 chain OS=Homo sapiens OX=9606 GN=TUBB6 PE=1 SV=1	49.8	4.88	TUBB6
P30101	Protein disulfide-isomerase A3 OS=Homo sapiens OX=9606 GN=PDIA3 PE=1 SV=4	56.7	6.35	PDIA3
P25705	ATP synthase subunit alpha, mitochondrial OS=Homo sapiens OX=9606 GN=ATP5F1A PE=1 SV=1	59.7	9.13	ATP5F1A

P60842	Eukaryotic initiation factor 4A-I OS=Homo sapiens OX=9606 GN=EIF4A1 PE=1 SV=1	46.1	5.48	EIF4A1
P52272	Heterogeneous nuclear ribonucleoprotein M OS=Homo sapiens OX=9606 GN=HNRNPM PE=1 SV=3	77.5	8.7	HNRNPM
Q86VP6	Cullin-associated NEDD8-dissociated protein 1 OS=Homo sapiens OX=9606 GN=CAND1 PE=1 SV=2	136.3	5.78	CAND1
P60174	Triosephosphate isomerase OS=Homo sapiens OX=9606 GN=TPI1 PE=1 SV=4	26.7	6.9	TPI1
P61978	Heterogeneous nuclear ribonucleoprotein K OS=Homo sapiens OX=9606 GN=HNRNPK PE=1 SV=1	50.9	5.54	HNRNPK
P05783	Keratin, type I cytoskeletal 18 OS=Homo sapiens OX=9606 GN=KRT18 PE=1 SV=2	48	5.45	KRT18
P05023	Sodium/potassium-transporting ATPase subunit alpha-1 OS=Homo sapiens OX=9606 GN=ATP1A1 PE=1 SV=1	112.8	5.49	ATP1A1
O43491	Band 4.1-like protein 2 OS=Homo sapiens OX=9606 GN=EPB41L2 PE=1 SV=1	112.5	5.44	EPB41L2
P09651	Heterogeneous nuclear ribonucleoprotein A1 OS=Homo sapiens OX=9606 GN=HNRNPA1 PE=1 SV=5	38.7	9.13	HNRNPA1
P09525	Annexin A4 OS=Homo sapiens OX=9606 GN=ANXA4 PE=1 SV=4	35.9	6.13	ANXA4
Q9P2E9	Ribosome-binding protein 1 OS=Homo sapiens OX=9606 GN=RRBP1 PE=1 SV=5	152.4	8.6	RRBP1
P33176	Kinesin-1 heavy chain OS=Homo sapiens OX=9606 GN=KIF5B PE=1 SV=1	109.6	6.51	KIF5B
Q9Y4L1	Hypoxia up-regulated protein 1 OS=Homo sapiens OX=9606 GN=HYOU1 PE=1 SV=1	111.3	5.22	HYOU1
P13010	X-ray repair cross-complementing protein 5 OS=Homo sapiens OX=9606 GN=XRCC5 PE=1 SV=3	82.7	5.81	XRCC5
P19338	Nucleolin OS=Homo sapiens OX=9606 GN=NCL PE=1 SV=3	76.6	4.7	NCL
P27797	Calreticulin OS=Homo sapiens OX=9606 GN=CALR PE=1 SV=1	48.1	4.44	CALR

P50990	T-complex protein 1 subunit theta OS=Homo sapiens OX=9606 GN=CCT8 PE=1 SV=4	59.6	5.6	CCT8
P08727	Keratin, type I cytoskeletal 19 OS=Homo sapiens OX=9606 GN=KRT19 PE=1 SV=4	44.1	5.14	KRT19
P63104	14-3-3 protein zeta/delta OS=Homo sapiens OX=9606 GN=YWHAZ PE=1 SV=1	27.7	4.79	YWHAZ
P09960	Leukotriene A-4 hydrolase OS=Homo sapiens OX=9606 GN=LTA4H PE=1 SV=2	69.2	6.18	LTA4H
P26599	Polypyrimidine tract-binding protein 1 OS=Homo sapiens OX=9606 GN=PTBP1 PE=1 SV=1	57.2	9.17	PTBP1
O60506	Heterogeneous nuclear ribonucleoprotein Q OS=Homo sapiens OX=9606 GN=SYNCRIP PE=1 SV=2	69.6	8.59	SYNCRIP
P30153	Serine/threonine-protein phosphatase 2A 65 kDa regulatory subunit A alpha isoform OS=Homo sapiens OX=9606 GN=PPP2R1A PE=1 SV=4	65.3	5.11	PPP2R1A
Q00341	Vigilin OS=Homo sapiens OX=9606 GN=HDLBP PE=1 SV=2	141.4	6.87	HDLBP
P50454	Serpin H1 OS=Homo sapiens OX=9606 GN=SERPINH1 PE=1 SV=2	46.4	8.69	SERPINH1
Q06830	Peroxiredoxin-1 OS=Homo sapiens OX=9606 GN=PRDX1 PE=1 SV=1	22.1	8.13	PRDX1
P35241	Radixin OS=Homo sapiens OX=9606 GN=RDX PE=1 SV=1	68.5	6.37	RDX
P08729	Keratin, type II cytoskeletal 7 OS=Homo sapiens OX=9606 GN=KRT7 PE=1 SV=5	51.4	5.48	KRT7
P48643	T-complex protein 1 subunit epsilon OS=Homo sapiens OX=9606 GN=CCT5 PE=1 SV=1	59.6	5.66	CCT5
P07814	Bifunctional glutamate/proline--tRNA ligase OS=Homo sapiens OX=9606 GN=EPRS1 PE=1 SV=5	170.5	7.33	EPRS1
Q9NZM1	Myoferlin OS=Homo sapiens OX=9606 GN=MYOF PE=1 SV=1	234.6	6.18	MYOF
P22626	Heterogeneous nuclear ribonucleoproteins A2/B1 OS=Homo sapiens OX=9606 GN=HNRNPA2B1 PE=1 SV=2	37.4	8.95	HNRNPA2B1

P49368	T-complex protein 1 subunit gamma OS=Homo sapiens OX=9606 GN=CCT3 PE=1 SV=4	60.5	6.49	CCT3
P55786	Puromycin-sensitive aminopeptidase OS=Homo sapiens OX=9606 GN=NPEPPS PE=1 SV=2	103.2	5.72	NPEPPS
P27816	Microtubule-associated protein 4 OS=Homo sapiens OX=9606 GN=MAP4 PE=1 SV=3	120.9	5.43	MAP4
Q86UP2	Kinectin OS=Homo sapiens OX=9606 GN=KTN1 PE=1 SV=1	156.2	5.64	KTN1
P55060	Exportin-2 OS=Homo sapiens OX=9606 GN=CSE1L PE=1 SV=3	110.3	5.77	CSE1L
P07195	L-lactate dehydrogenase B chain OS=Homo sapiens OX=9606 GN=LDHB PE=1 SV=2	36.6	6.05	LDHB
Q14980	Nuclear mitotic apparatus protein 1 OS=Homo sapiens OX=9606 GN=NUMA1 PE=1 SV=2	238.1	5.78	NUMA1
Q01518	Adenylyl cyclase-associated protein 1 OS=Homo sapiens OX=9606 GN=CAP1 PE=1 SV=5	51.9	8.06	CAP1
Q07065	Cytoskeleton-associated protein 4 OS=Homo sapiens OX=9606 GN=CKAP4 PE=1 SV=2	66	5.92	CKAP4
P09429	High mobility group protein B1 OS=Homo sapiens OX=9606 GN=HMGB1 PE=1 SV=3	24.9	5.74	HMGB1
P67936	Tropomyosin alpha-4 chain OS=Homo sapiens OX=9606 GN=TPM4 PE=1 SV=3	28.5	4.69	TPM4
P06744	Glucose-6-phosphate isomerase OS=Homo sapiens OX=9606 GN=GPI PE=1 SV=4	63.1	8.32	GPI
P17987	T-complex protein 1 subunit alpha OS=Homo sapiens OX=9606 GN=TCP1 PE=1 SV=1	60.3	6.11	TCP1
P62258	14-3-3 protein epsilon OS=Homo sapiens OX=9606 GN=YWHAE PE=1 SV=1	29.2	4.74	YWHAE
P31948	Stress-induced-phosphoprotein 1 OS=Homo sapiens OX=9606 GN=STIP1 PE=1 SV=1	62.6	6.8	STIP1
P40939	Trifunctional enzyme subunit alpha, mitochondrial OS=Homo sapiens OX=9606 GN=HADHA PE=1 SV=2	82.9	9.04	HADHA

Q16555	Dihydropyrimidinase-related protein 2 OS=Homo sapiens OX=9606 GN=DPYSL2 PE=1 SV=1	62.3	6.38	DPYSL2
P23528	Cofilin-1 OS=Homo sapiens OX=9606 GN=CFL1 PE=1 SV=3	18.5	8.09	CFL1
Q14974	Importin subunit beta-1 OS=Homo sapiens OX=9606 GN=KPNB1 PE=1 SV=2	97.1	4.78	KPNB1
P46821	Microtubule-associated protein 1B OS=Homo sapiens OX=9606 GN=MAP1B PE=1 SV=2	270.5	4.81	MAP1B
Q00839	Heterogeneous nuclear ribonucleoprotein U OS=Homo sapiens OX=9606 GN=HNRNPU PE=1 SV=6	90.5	6	HNRNPU
P08195	4F2 cell-surface antigen heavy chain OS=Homo sapiens OX=9606 GN=SLC3A2 PE=1 SV=3	68	5.01	SLC3A2
P18669	Phosphoglycerate mutase 1 OS=Homo sapiens OX=9606 GN=PGAM1 PE=1 SV=2	28.8	7.18	PGAM1
P36871	Phosphoglucomutase-1 OS=Homo sapiens OX=9606 GN=PGM1 PE=1 SV=3	61.4	6.76	PGM1
P06748	Nucleophosmin OS=Homo sapiens OX=9606 GN=NPM1 PE=1 SV=2	32.6	4.78	NPM1
O00410	Importin-5 OS=Homo sapiens OX=9606 GN=IPO5 PE=1 SV=4	123.6	4.94	IPO5
Q7Z6Z7	E3 ubiquitin-protein ligase HUWE1 OS=Homo sapiens OX=9606 GN=HUWE1 PE=1 SV=3	481.6	5.22	HUWE1
P34932	Heat shock 70 kDa protein 4 OS=Homo sapiens OX=9606 GN=HSPA4 PE=1 SV=4	94.3	5.19	HSPA4
Q92598	Heat shock protein 105 kDa OS=Homo sapiens OX=9606 GN=HSPH1 PE=1 SV=1	96.8	5.39	HSPH1
P31939	Bifunctional purine biosynthesis protein ATIC OS=Homo sapiens OX=9606 GN=ATIC PE=1 SV=3	64.6	6.71	ATIC
P31946	14-3-3 protein beta/alpha OS=Homo sapiens OX=9606 GN=YWHAB PE=1 SV=3	28.1	4.83	YWHAB
P50991	T-complex protein 1 subunit delta OS=Homo sapiens OX=9606 GN=CCT4 PE=1 SV=4	57.9	7.83	CCT4

P09211	Glutathione S-transferase P OS=Homo sapiens OX=9606 GN=GSTP1 PE=1 SV=2	23.3	5.64	GSTP1
P26641	Elongation factor 1-gamma OS=Homo sapiens OX=9606 GN=EEF1G PE=1 SV=3	50.1	6.67	EEF1G
P15924	Desmoplakin OS=Homo sapiens OX=9606 GN=DSP PE=1 SV=3	331.6	6.81	DSP
P21796	Voltage-dependent anion-selective channel protein 1 OS=Homo sapiens OX=9606 GN=VDAC1 PE=1 SV=2	30.8	8.54	VDAC1
P54652	Heat shock-related 70 kDa protein 2 OS=Homo sapiens OX=9606 GN=HSPA2 PE=1 SV=1	70	5.74	HSPA2
Q13200	26S proteasome non-ATPase regulatory subunit 2 OS=Homo sapiens OX=9606 GN=PSMD2 PE=1 SV=3	100.1	5.2	PSMD2
P36578	60S ribosomal protein L4 OS=Homo sapiens OX=9606 GN=RPL4 PE=1 SV=5	47.7	11.06	RPL4
Q9H6S3	Epidermal growth factor receptor kinase substrate 8-like protein 2 OS=Homo sapiens OX=9606 GN=EPS8L2 PE=1 SV=2	80.6	6.84	EPS8L2
P36776	Lon protease homolog, mitochondrial OS=Homo sapiens OX=9606 GN=LONP1 PE=1 SV=2	106.4	6.39	LONP1
Q9BSJ8	Extended synaptotagmin-1 OS=Homo sapiens OX=9606 GN=ESYT1 PE=1 SV=1	122.8	5.83	ESYT1
Q96QK1	Vacuolar protein sorting-associated protein 35 OS=Homo sapiens OX=9606 GN=VPS35 PE=1 SV=2	91.6	5.49	VPS35
Q96TA1	Protein Niban 2 OS=Homo sapiens OX=9606 GN=NIBAN2 PE=1 SV=3	84.1	6.19	NIBAN2
O94925	Glutaminase kidney isoform, mitochondrial OS=Homo sapiens OX=9606 GN=GLS PE=1 SV=1	73.4	7.77	GLS
O00571	ATP-dependent RNA helicase DDX3X OS=Homo sapiens OX=9606 GN=DDX3X PE=1 SV=3	73.2	7.18	DDX3X
P63010	AP-2 complex subunit beta OS=Homo sapiens OX=9606 GN=AP2B1 PE=1 SV=1	104.5	5.38	AP2B1
P13797	Plastin-3 OS=Homo sapiens OX=9606 GN=PLS3 PE=1 SV=4	70.8	5.6	PLS3

P49411	Elongation factor Tu, mitochondrial OS=Homo sapiens OX=9606 GN=TUFM PE=1 SV=2	49.5	7.61	TUFM
P51991	Heterogeneous nuclear ribonucleoprotein A3 OS=Homo sapiens OX=9606 GN=HNRNPA3 PE=1 SV=2	39.6	9.01	HNRNPA3
P53621	Coatomer subunit alpha OS=Homo sapiens OX=9606 GN=COPA PE=1 SV=2	138.3	7.66	COPA
P16152	Carbonyl reductase [NADPH] 1 OS=Homo sapiens OX=9606 GN=CBR1 PE=1 SV=3	30.4	8.32	CBR1
P43243	Matrin-3 OS=Homo sapiens OX=9606 GN=MATR3 PE=1 SV=2	94.6	6.25	MATR3
P31150	Rab GDP dissociation inhibitor alpha OS=Homo sapiens OX=9606 GN=GDI1 PE=1 SV=2	50.6	5.14	GDI1
Q14240	Eukaryotic initiation factor 4A-II OS=Homo sapiens OX=9606 GN=EIF4A2 PE=1 SV=2	46.4	5.48	EIF4A2
P07339	Cathepsin D OS=Homo sapiens OX=9606 GN=CTSD PE=1 SV=1	44.5	6.54	CTSD
Q8WUM4	Programmed cell death 6-interacting protein OS=Homo sapiens OX=9606 GN=PDCD6IP PE=1 SV=1	96	6.52	PDCD6IP
P13647	Keratin, type II cytoskeletal 5 OS=Homo sapiens OX=9606 GN=KRT5 PE=1 SV=3	62.3	7.74	KRT5
P22392	Nucleoside diphosphate kinase B OS=Homo sapiens OX=9606 GN=NME2 PE=1 SV=1	17.3	8.41	NME2
P62937	Peptidyl-prolyl cis-trans isomerase A OS=Homo sapiens OX=9606 GN=PPIA PE=1 SV=2	18	7.81	PPIA
P35237	Serpin B6 OS=Homo sapiens OX=9606 GN=SERPINB6 PE=1 SV=3	42.6	5.27	SERPINB6
Q07960	Rho GTPase-activating protein 1 OS=Homo sapiens OX=9606 GN=ARHGAP1 PE=1 SV=1	50.4	6.29	ARHGAP1
Q04637	Eukaryotic translation initiation factor 4 gamma 1 OS=Homo sapiens OX=9606 GN=EIF4G1 PE=1 SV=4	175.4	5.33	EIF4G1
Q99460	26S proteasome non-ATPase regulatory subunit 1 OS=Homo sapiens OX=9606 GN=PSMD1 PE=1 SV=2	105.8	5.39	PSMD1

P04792	Heat shock protein beta-1 OS=Homo sapiens OX=9606 GN=HSPB1 PE=1 SV=2	22.8	6.4	HSPB1
P19367	Hexokinase-1 OS=Homo sapiens OX=9606 GN=HK1 PE=1 SV=3	102.4	6.8	HK1
Q13263	Transcription intermediary factor 1-beta OS=Homo sapiens OX=9606 GN=TRIM28 PE=1 SV=5	88.5	5.77	TRIM28
Q99798	Aconitate hydratase, mitochondrial OS=Homo sapiens OX=9606 GN=ACO2 PE=1 SV=2	85.4	7.61	ACO2
P35221	Catenin alpha-1 OS=Homo sapiens OX=9606 GN=CTNNA1 PE=1 SV=1	100	6.29	CTNNA1
P53618	Coatomer subunit beta OS=Homo sapiens OX=9606 GN=COPB1 PE=1 SV=3	107.1	6.05	COPB1
P02533	Keratin, type I cytoskeletal 14 OS=Homo sapiens OX=9606 GN=KRT14 PE=1 SV=4	51.5	5.16	KRT14
P00491	Purine nucleoside phosphorylase OS=Homo sapiens OX=9606 GN=PNP PE=1 SV=2	32.1	6.95	PNP
P49915	GMP synthase [glutamine-hydrolyzing] OS=Homo sapiens OX=9606 GN=GMPS PE=1 SV=1	76.7	6.87	GMPS
P40227	T-complex protein 1 subunit zeta OS=Homo sapiens OX=9606 GN=CCT6A PE=1 SV=3	58	6.68	CCT6A
P11586	C-1-tetrahydrofolate synthase, cytoplasmic OS=Homo sapiens OX=9606 GN=MTHFD1 PE=1 SV=4	101.5	7.3	MTHFD1
P11413	Glucose-6-phosphate 1-dehydrogenase OS=Homo sapiens OX=9606 GN=G6PD PE=1 SV=4	59.2	6.84	G6PD
Q14152	Eukaryotic translation initiation factor 3 subunit A OS=Homo sapiens OX=9606 GN=EIF3A PE=1 SV=1	166.5	6.79	EIF3A
P67809	Y-box-binding protein 1 OS=Homo sapiens OX=9606 GN=YBX1 PE=1 SV=3	35.9	9.88	YBX1
P54886	Delta-1-pyrroline-5-carboxylate synthase OS=Homo sapiens OX=9606 GN=ALDH18A1 PE=1 SV=2	87.2	7.12	ALDH18A1
O14980	Exportin-1 OS=Homo sapiens OX=9606 GN=XPO1 PE=1 SV=1	123.3	6.06	XPO1

P12277	Creatine kinase B-type OS=Homo sapiens OX=9606 GN=CKB PE=1 SV=1	42.6	5.59	CKB
Q99832	T-complex protein 1 subunit eta OS=Homo sapiens OX=9606 GN=CCT7 PE=1 SV=2	59.3	7.65	CCT7
P51659	Peroxisomal multifunctional enzyme type 2 OS=Homo sapiens OX=9606 GN=HSD17B4 PE=1 SV=3	79.6	8.84	HSD17B4
Q92945	Far upstream element-binding protein 2 OS=Homo sapiens OX=9606 GN=KHSRP PE=1 SV=4	73.1	7.3	KHSRP
Q12906	Interleukin enhancer-binding factor 3 OS=Homo sapiens OX=9606 GN=ILF3 PE=1 SV=3	95.3	8.76	ILF3
P49748	Very long-chain specific acyl-CoA dehydrogenase, mitochondrial OS=Homo sapiens OX=9606 GN=ACADVL PE=1 SV=1	70.3	8.75	ACADVL
P04844	Dolichyl-diphosphooligosaccharide--protein glycosyltransferase subunit 2 OS=Homo sapiens OX=9606 GN=RPN2 PE=1 SV=3	69.2	5.69	RPN2
P05556	Integrin beta-1 OS=Homo sapiens OX=9606 GN=ITGB1 PE=1 SV=2	88.4	5.39	ITGB1
Q15233	Non-POU domain-containing octamer-binding protein OS=Homo sapiens OX=9606 GN=NONO PE=1 SV=4	54.2	8.95	NONO
P15121	Aldo-keto reductase family 1 member B1 OS=Homo sapiens OX=9606 GN=AKR1B1 PE=1 SV=3	35.8	6.98	AKR1B1
P30041	Peroxiredoxin-6 OS=Homo sapiens OX=9606 GN=PRDX6 PE=1 SV=3	25	6.38	PRDX6
P23246	Splicing factor, proline- and glutamine-rich OS=Homo sapiens OX=9606 GN=SFPQ PE=1 SV=2	76.1	9.44	SFPQ
Q9UQ80	Proliferation-associated protein 2G4 OS=Homo sapiens OX=9606 GN=PA2G4 PE=1 SV=3	43.8	6.55	PA2G4
P40763	Signal transducer and activator of transcription 3 OS=Homo sapiens OX=9606 GN=STAT3 PE=1 SV=2	88	6.3	STAT3
Q16891	MICOS complex subunit MIC60 OS=Homo sapiens OX=9606 GN=IMMT PE=1 SV=1	83.6	6.48	IMMT

P11940	Polyadenylate-binding protein 1 OS=Homo sapiens OX=9606 GN=PABPC1 PE=1 SV=2	70.6	9.5	PABPC1
P27824	Calnexin OS=Homo sapiens OX=9606 GN=CANX PE=1 SV=2	67.5	4.6	CANX
P17858	ATP-dependent 6-phosphofructokinase, liver type OS=Homo sapiens OX=9606 GN=PFKL PE=1 SV=6	85	7.5	PFKL
O43242	26S proteasome non-ATPase regulatory subunit 3 OS=Homo sapiens OX=9606 GN=PSMD3 PE=1 SV=2	60.9	8.44	PSMD3
P06753	Tropomyosin alpha-3 chain OS=Homo sapiens OX=9606 GN=TPM3 PE=1 SV=2	32.9	4.72	TPM3
P50570	Dynamin-2 OS=Homo sapiens OX=9606 GN=DNM2 PE=1 SV=2	98	7.44	DNM2
O60701	UDP-glucose 6-dehydrogenase OS=Homo sapiens OX=9606 GN=UGDH PE=1 SV=1	55	7.12	UGDH
Q16881	Thioredoxin reductase 1, cytoplasmic OS=Homo sapiens OX=9606 GN=TXNRD1 PE=1 SV=3	70.9	7.39	TXNRD1
P42330	Aldo-keto reductase family 1 member C3 OS=Homo sapiens OX=9606 GN=AKR1C3 PE=1 SV=4	36.8	7.94	AKR1C3
Q15019	Septin-2 OS=Homo sapiens OX=9606 GN=SEPTIN2 PE=1 SV=1	41.5	6.6	SEPTIN2
P23381	Tryptophan--tRNA ligase, cytoplasmic OS=Homo sapiens OX=9606 GN=WARS1 PE=1 SV=2	53.1	6.23	WARS1
P35606	Coatomer subunit beta' OS=Homo sapiens OX=9606 GN=COPB2 PE=1 SV=2	102.4	5.27	COPB2
P63241	Eukaryotic translation initiation factor 5A-1 OS=Homo sapiens OX=9606 GN=EIF5A PE=1 SV=2	16.8	5.24	EIF5A
P26640	Valine--tRNA ligase OS=Homo sapiens OX=9606 GN=VARS1 PE=1 SV=4	140.4	7.59	VARS1
P04843	Dolichyl-diphosphooligosaccharide--protein glycosyltransferase subunit 1 OS=Homo sapiens OX=9606 GN=RPN1 PE=1 SV=1	68.5	6.38	RPN1
P05455	Lupus La protein OS=Homo sapiens OX=9606 GN=SSB PE=1 SV=2	46.8	7.12	SSB

O43776	Asparagine--tRNA ligase, cytoplasmic OS=Homo sapiens OX=9606 GN=NARS1 PE=1 SV=1	62.9	6.25	NARS1
P22234	Multifunctional protein ADE2 OS=Homo sapiens OX=9606 GN=PAICS PE=1 SV=3	47	7.23	PAICS
P20700	Lamin-B1 OS=Homo sapiens OX=9606 GN=LMNB1 PE=1 SV=2	66.4	5.16	LMNB1
Q15084	Protein disulfide-isomerase A6 OS=Homo sapiens OX=9606 GN=PDIA6 PE=1 SV=1	48.1	5.08	PDIA6
P14868	Aspartate--tRNA ligase, cytoplasmic OS=Homo sapiens OX=9606 GN=DARS1 PE=1 SV=2	57.1	6.55	DARS1
O75083	WD repeat-containing protein 1 OS=Homo sapiens OX=9606 GN=WDR1 PE=1 SV=4	66.2	6.65	WDR1
P05388	60S acidic ribosomal protein P0 OS=Homo sapiens OX=9606 GN=RPLP0 PE=1 SV=1	34.3	5.97	RPLP0
P06396	Gelsolin OS=Homo sapiens OX=9606 GN=GSN PE=1 SV=1	85.6	6.28	GSN
Q9UJZ1	Stomatin-like protein 2, mitochondrial OS=Homo sapiens OX=9606 GN=STOML2 PE=1 SV=1	38.5	7.39	STOML2
O00299	Chloride intracellular channel protein 1 OS=Homo sapiens OX=9606 GN=CLIC1 PE=1 SV=4	26.9	5.17	CLIC1
O00764	Pyridoxal kinase OS=Homo sapiens OX=9606 GN=PDXK PE=1 SV=1	35.1	6.13	PDXK
Q14247	Src substrate cortactin OS=Homo sapiens OX=9606 GN=CTTN PE=1 SV=2	61.5	5.4	CTTN
Q9NYU2	UDP-glucose:glycoprotein glucosyltransferase 1 OS=Homo sapiens OX=9606 GN=UGGT1 PE=1 SV=3	177.1	5.63	UGGT1
P17980	26S proteasome regulatory subunit 6A OS=Homo sapiens OX=9606 GN=PSMC3 PE=1 SV=3	49.2	5.24	PSMC3
Q02878	60S ribosomal protein L6 OS=Homo sapiens OX=9606 GN=RPL6 PE=1 SV=3	32.7	10.58	RPL6
Q99497	Parkinson disease protein 7 OS=Homo sapiens OX=9606 GN=PARK7 PE=1 SV=2	19.9	6.79	PARK7

P39023	60S ribosomal protein L3 OS=Homo sapiens OX=9606 GN=RPL3 PE=1 SV=2	46.1	10.18	RPL3
Q16531	DNA damage-binding protein 1 OS=Homo sapiens OX=9606 GN=DDB1 PE=1 SV=1	126.9	5.26	DDB1
Q15046	Lysine--tRNA ligase OS=Homo sapiens OX=9606 GN=KARS1 PE=1 SV=3	68	6.35	KARS1
P08865	40S ribosomal protein SA OS=Homo sapiens OX=9606 GN=RPSA PE=1 SV=4	32.8	4.87	RPSA
P41252	Isoleucine--tRNA ligase, cytoplasmic OS=Homo sapiens OX=9606 GN=IARS1 PE=1 SV=2	144.4	6.15	IARS1
P27708	CAD protein OS=Homo sapiens OX=9606 GN=CAD PE=1 SV=3	242.8	6.46	CAD
Q15365	Poly(rC)-binding protein 1 OS=Homo sapiens OX=9606 GN=PCBP1 PE=1 SV=2	37.5	7.09	PCBP1
P30086	Phosphatidylethanolamine-binding protein 1 OS=Homo sapiens OX=9606 GN=PEBP1 PE=1 SV=3	21	7.53	PEBP1
O43390	Heterogeneous nuclear ribonucleoprotein R OS=Homo sapiens OX=9606 GN=HNRNPR PE=1 SV=1	70.9	8.13	HNRNPR
P63244	Receptor of activated protein C kinase 1 OS=Homo sapiens OX=9606 GN=RACK1 PE=1 SV=3	35.1	7.69	RACK1
P23526	Adenosylhomocysteinase OS=Homo sapiens OX=9606 GN=AHCY PE=1 SV=4	47.7	6.34	AHCY
O60664	Perilipin-3 OS=Homo sapiens OX=9606 GN=PLIN3 PE=1 SV=3	47	5.44	PLIN3
P08779	Keratin, type I cytoskeletal 16 OS=Homo sapiens OX=9606 GN=KRT16 PE=1 SV=4	51.2	5.05	KRT16
P40926	Malate dehydrogenase, mitochondrial OS=Homo sapiens OX=9606 GN=MDH2 PE=1 SV=3	35.5	8.68	MDH2
P30084	Enoyl-CoA hydratase, mitochondrial OS=Homo sapiens OX=9606 GN=ECHS1 PE=1 SV=4	31.4	8.07	ECHS1
Q92841	Probable ATP-dependent RNA helicase DDX17 OS=Homo sapiens OX=9606 GN=DDX17 PE=1 SV=2	80.2	8.27	DDX17

P37802	Transgelin-2 OS=Homo sapiens OX=9606 GN=TAGLN2 PE=1 SV=3	22.4	8.25	TAGLN2
Q9UHD8	Septin-9 OS=Homo sapiens OX=9606 GN=SEPTIN9 PE=1 SV=2	65.4	8.97	SEPTIN9
P23396	40S ribosomal protein S3 OS=Homo sapiens OX=9606 GN=RPS3 PE=1 SV=2	26.7	9.66	RPS3
O75533	Splicing factor 3B subunit 1 OS=Homo sapiens OX=9606 GN=SF3B1 PE=1 SV=3	145.7	7.09	SF3B1
P06737	Glycogen phosphorylase, liver form OS=Homo sapiens OX=9606 GN=PYGL PE=1 SV=4	97.1	7.17	PYGL
Q03252	Lamin-B2 OS=Homo sapiens OX=9606 GN=LMNB2 PE=1 SV=4	69.9	5.59	LMNB2
P07737	Profilin-1 OS=Homo sapiens OX=9606 GN=PFN1 PE=1 SV=2	15	8.27	PFN1
Q6P2E9	Enhancer of mRNA-decapping protein 4 OS=Homo sapiens OX=9606 GN=EDC4 PE=1 SV=1	151.6	5.86	EDC4
P51858	Hepatoma-derived growth factor OS=Homo sapiens OX=9606 GN=HDGF PE=1 SV=1	26.8	4.73	HDGF
P02538	Keratin, type II cytoskeletal 6A OS=Homo sapiens OX=9606 GN=KRT6A PE=1 SV=3	60	8	KRT6A
P07951	Tropomyosin beta chain OS=Homo sapiens OX=9606 GN=TPM2 PE=1 SV=1	32.8	4.7	TPM2
P31943	Heterogeneous nuclear ribonucleoprotein H OS=Homo sapiens OX=9606 GN=HNRNPH1 PE=1 SV=4	49.2	6.3	HNRNPH1
P41091	Eukaryotic translation initiation factor 2 subunit 3 OS=Homo sapiens OX=9606 GN=EIF2S3 PE=1 SV=3	51.1	8.4	EIF2S3
O00231	26S proteasome non-ATPase regulatory subunit 11 OS=Homo sapiens OX=9606 GN=PSMD11 PE=1 SV=3	47.4	6.48	PSMD11
P00387	NADH-cytochrome b5 reductase 3 OS=Homo sapiens OX=9606 GN=CYB5R3 PE=1 SV=3	34.2	7.59	CYB5R3
P49591	Serine--tRNA ligase, cytoplasmic OS=Homo sapiens OX=9606 GN=SARS1 PE=1 SV=3	58.7	6.43	SARS1

P43686	26S proteasome regulatory subunit 6B OS=Homo sapiens OX=9606 GN=PSMC4 PE=1 SV=2	47.3	5.21	PSMC4
P16615	Sarcoplasmic/endoplasmic reticulum calcium ATPase 2 OS=Homo sapiens OX=9606 GN=ATP2A2 PE=1 SV=1	114.7	5.34	ATP2A2
P55209	Nucleosome assembly protein 1-like 1 OS=Homo sapiens OX=9606 GN=NAP1L1 PE=1 SV=1	45.3	4.46	NAP1L1
Q58FF8	Putative heat shock protein HSP 90-beta 2 OS=Homo sapiens OX=9606 GN=HSP90AB2P PE=1 SV=2	44.3	4.84	HSP90AB2P
P62191	26S proteasome regulatory subunit 4 OS=Homo sapiens OX=9606 GN=PSMC1 PE=1 SV=1	49.2	6.21	PSMC1
Q7L576	Cytoplasmic FMR1-interacting protein 1 OS=Homo sapiens OX=9606 GN=CYFIP1 PE=1 SV=1	145.1	6.9	CYFIP1
P49419	Alpha-aminoadipic semialdehyde dehydrogenase OS=Homo sapiens OX=9606 GN=ALDH7A1 PE=1 SV=5	58.5	7.99	ALDH7A1
P37837	Transaldolase OS=Homo sapiens OX=9606 GN=TALDO1 PE=1 SV=2	37.5	6.81	TALDO1
Q99613	Eukaryotic translation initiation factor 3 subunit C OS=Homo sapiens OX=9606 GN=EIF3C PE=1 SV=1	105.3	5.68	EIF3C
P62805	Histone H4 OS=Homo sapiens OX=9606 GN=H4C1 PE=1 SV=2	11.4	11.36	H4-16; H4C1; H4C11; H4C12; H4C13; H4C14; H4C15; H4C2; H4C3; H4C4; H4C5; H4C6; H4C8; H4C9
O75874	Isocitrate dehydrogenase [NADP] cytoplasmic OS=Homo sapiens OX=9606 GN=IDH1 PE=1 SV=2	46.6	7.01	IDH1
P13674	Prolyl 4-hydroxylase subunit alpha-1 OS=Homo sapiens OX=9606 GN=P4HA1 PE=1 SV=2	61	6.01	P4HA1
Q96KP4	Cytosolic non-specific dipeptidase OS=Homo sapiens OX=9606 GN=CNDP2 PE=1 SV=2	52.8	5.97	CNDP2
P38919	Eukaryotic initiation factor 4A-III OS=Homo sapiens OX=9606 GN=EIF4A3 PE=1 SV=4	46.8	6.73	EIF4A3
P52597	Heterogeneous nuclear ribonucleoprotein F OS=Homo sapiens OX=9606 GN=HNRNPF PE=1 SV=3	45.6	5.58	HNRNPF

P35749	Myosin-11 OS=Homo sapiens OX=9606 GN=MYH11 PE=1 SV=3	227.2	5.5	MYH11
P04259	Keratin, type II cytoskeletal 6B OS=Homo sapiens OX=9606 GN=KRT6B PE=1 SV=5	60	8	KRT6B
P54136	Arginine--tRNA ligase, cytoplasmic OS=Homo sapiens OX=9606 GN=RARS1 PE=1 SV=2	75.3	6.68	RARS1
P34897	Serine hydroxymethyltransferase, mitochondrial OS=Homo sapiens OX=9606 GN=SHMT2 PE=1 SV=3	56	8.53	SHMT2
Q7KZF4	Staphylococcal nuclease domain-containing protein 1 OS=Homo sapiens OX=9606 GN=SND1 PE=1 SV=1	101.9	7.17	SND1
P27348	14-3-3 protein theta OS=Homo sapiens OX=9606 GN=YWHAQ PE=1 SV=1	27.7	4.78	YWHAQ
Q15417	Calponin-3 OS=Homo sapiens OX=9606 GN=CNN3 PE=1 SV=1	36.4	6.05	CNN3
O00232	26S proteasome non-ATPase regulatory subunit 12 OS=Homo sapiens OX=9606 GN=PSMD12 PE=1 SV=3	52.9	7.65	PSMD12
Q9Y262	Eukaryotic translation initiation factor 3 subunit L OS=Homo sapiens OX=9606 GN=EIF3L PE=1 SV=1	66.7	6.34	EIF3L
Q562R1	Beta-actin-like protein 2 OS=Homo sapiens OX=9606 GN=ACTBL2 PE=1 SV=2	42	5.59	ACTBL2
P47897	Glutamine--tRNA ligase OS=Homo sapiens OX=9606 GN=QARS1 PE=1 SV=1	87.7	7.15	QARS1
Q9NY33	Dipeptidyl peptidase 3 OS=Homo sapiens OX=9606 GN=DPP3 PE=1 SV=2	82.5	5.1	DPP3
P28838	Cytosol aminopeptidase OS=Homo sapiens OX=9606 GN=LAP3 PE=1 SV=3	56.1	7.93	LAP3
Q12931	Heat shock protein 75 kDa, mitochondrial OS=Homo sapiens OX=9606 GN=TRAP1 PE=1 SV=3	80.1	8.21	TRAP1
P51149	Ras-related protein Rab-7a OS=Homo sapiens OX=9606 GN=RAB7A PE=1 SV=1	23.5	6.7	RAB7A
Q9Y265	RuvB-like 1 OS=Homo sapiens OX=9606 GN=RUVBL1 PE=1 SV=1	50.2	6.42	RUVBL1

Q15008	26S proteasome non-ATPase regulatory subunit 6 OS=Homo sapiens OX=9606 GN=PSMD6 PE=1 SV=1	45.5	5.62	PSMD6
P42224	Signal transducer and activator of transcription 1-alpha/beta OS=Homo sapiens OX=9606 GN=STAT1 PE=1 SV=2	87.3	6.05	STAT1
P07954	Fumarate hydratase, mitochondrial OS=Homo sapiens OX=9606 GN=FH PE=1 SV=3	54.6	8.76	FH
Q12905	Interleukin enhancer-binding factor 2 OS=Homo sapiens OX=9606 GN=ILF2 PE=1 SV=2	43	5.26	ILF2
Q6P2Q9	Pre-mRNA-processing-splicing factor 8 OS=Homo sapiens OX=9606 GN=PRPF8 PE=1 SV=2	273.4	8.84	PRPF8
Q9HB71	Calcyclin-binding protein OS=Homo sapiens OX=9606 GN=CACYBP PE=1 SV=2	26.2	8.25	CACYBP
P40925	Malate dehydrogenase, cytoplasmic OS=Homo sapiens OX=9606 GN=MDH1 PE=1 SV=4	36.4	7.36	MDH1
P62195	26S proteasome regulatory subunit 8 OS=Homo sapiens OX=9606 GN=PSMC5 PE=1 SV=1	45.6	7.55	PSMC5
Q9Y230	RuvB-like 2 OS=Homo sapiens OX=9606 GN=RUVBL2 PE=1 SV=3	51.1	5.64	RUVBL2
Q13838	Spliceosome RNA helicase DDX39B OS=Homo sapiens OX=9606 GN=DDX39B PE=1 SV=1	49	5.67	DDX39B
Q5T4S7	E3 ubiquitin-protein ligase UBR4 OS=Homo sapiens OX=9606 GN=UBR4 PE=1 SV=1	573.5	6.04	UBR4
Q13085	Acetyl-CoA carboxylase 1 OS=Homo sapiens OX=9606 GN=ACACA PE=1 SV=2	265.4	6.37	ACACA
P52209	6-phosphogluconate dehydrogenase, decarboxylating OS=Homo sapiens OX=9606 GN=PGD PE=1 SV=3	53.1	7.23	PGD
P61981	14-3-3 protein gamma OS=Homo sapiens OX=9606 GN=YWHAG PE=1 SV=2	28.3	4.89	YWHAG
Q04917	14-3-3 protein eta OS=Homo sapiens OX=9606 GN=YWHAH PE=1 SV=4	28.2	4.84	YWHAH
P07910	Heterogeneous nuclear ribonucleoproteins C1/C2 OS=Homo sapiens OX=9606 GN=HNRNPC PE=1 SV=4	33.7	5.08	HNRNPC

P61247	40S ribosomal protein S3a OS=Homo sapiens OX=9606 GN=RPS3A PE=1 SV=2	29.9	9.73	RPS3A
Q06210	Glutamine--fructose-6-phosphate aminotransferase [isomerizing] 1 OS=Homo sapiens OX=9606 GN=GFPT1 PE=1 SV=3	78.8	7.11	GFPT1
P62701	40S ribosomal protein S4, X isoform OS=Homo sapiens OX=9606 GN=RPS4X PE=1 SV=2	29.6	10.15	RPS4X
P26639	Threonine--tRNA ligase 1, cytoplasmic OS=Homo sapiens OX=9606 GN=TARS1 PE=1 SV=3	83.4	6.67	TARS1
O00159	Unconventional myosin-Ic OS=Homo sapiens OX=9606 GN=MYO1C PE=1 SV=4	121.6	9.41	MYO1C
P15531	Nucleoside diphosphate kinase A OS=Homo sapiens OX=9606 GN=NME1 PE=1 SV=1	17.1	6.19	NME1
Q16658	Fascin OS=Homo sapiens OX=9606 GN=FSCN1 PE=1 SV=3	54.5	7.24	FSCN1
Q8NC51	Plasminogen activator inhibitor 1 RNA-binding protein OS=Homo sapiens OX=9606 GN=SERBP1 PE=1 SV=2	44.9	8.65	SERBP1
Q8N163	Cell cycle and apoptosis regulator protein 2 OS=Homo sapiens OX=9606 GN=CCAR2 PE=1 SV=2	102.8	5.22	CCAR2
P31947	14-3-3 protein sigma OS=Homo sapiens OX=9606 GN=SFN PE=1 SV=1	27.8	4.74	SFN
P12004	Proliferating cell nuclear antigen OS=Homo sapiens OX=9606 GN=PCNA PE=1 SV=1	28.8	4.69	PCNA
O43143	Pre-mRNA-splicing factor ATP-dependent RNA helicase DHX15 OS=Homo sapiens OX=9606 GN=DHX15 PE=1 SV=2	90.9	7.46	DHX15
P13667	Protein disulfide-isomerase A4 OS=Homo sapiens OX=9606 GN=PDIA4 PE=1 SV=2	72.9	5.07	PDIA4
P48444	Coatomer subunit delta OS=Homo sapiens OX=9606 GN=ARCN1 PE=1 SV=1	57.2	6.21	ARCN1
Q05639	Elongation factor 1-alpha 2 OS=Homo sapiens OX=9606 GN=EEF1A2 PE=1 SV=1	50.4	9.03	EEF1A2

Q15436	Protein transport protein Sec23A OS=Homo sapiens OX=9606 GN=SEC23A PE=1 SV=2	86.1	7.08	SEC23A
Q14195	Dihydropyrimidinase-related protein 3 OS=Homo sapiens OX=9606 GN=DPYSL3 PE=1 SV=1	61.9	6.49	DPYSL3
O60763	General vesicular transport factor p115 OS=Homo sapiens OX=9606 GN=USO1 PE=1 SV=2	107.8	4.91	USO1
P78347	General transcription factor II-I OS=Homo sapiens OX=9606 GN=GTF2I PE=1 SV=2	112.3	6.39	GTF2I
Q92900	Regulator of nonsense transcripts 1 OS=Homo sapiens OX=9606 GN=UPF1 PE=1 SV=2	124.3	6.61	UPF1
Q99714	3-hydroxyacyl-CoA dehydrogenase type-2 OS=Homo sapiens OX=9606 GN=HSD17B10 PE=1 SV=3	26.9	7.78	HSD17B10
P78344	Eukaryotic translation initiation factor 4 gamma 2 OS=Homo sapiens OX=9606 GN=EIF4G2 PE=1 SV=1	102.3	7.14	EIF4G2
P35998	26S proteasome regulatory subunit 7 OS=Homo sapiens OX=9606 GN=PSMC2 PE=1 SV=3	48.6	5.95	PSMC2
Q9H4A4	Aminopeptidase B OS=Homo sapiens OX=9606 GN=RNPEP PE=1 SV=2	72.5	5.74	RNPEP
Q15075	Early endosome antigen 1 OS=Homo sapiens OX=9606 GN=EEA1 PE=1 SV=2	162.4	5.68	EEA1
Q99733	Nucleosome assembly protein 1-like 4 OS=Homo sapiens OX=9606 GN=NAP1L4 PE=1 SV=1	42.8	4.69	NAP1L4
Q9Y2Z4	Tyrosine--tRNA ligase, mitochondrial OS=Homo sapiens OX=9606 GN=YARS2 PE=1 SV=2	53.2	8.98	YARS2
Q96AE4	Far upstream element-binding protein 1 OS=Homo sapiens OX=9606 GN=FUBP1 PE=1 SV=3	67.5	7.61	FUBP1
P40121	Macrophage-capping protein OS=Homo sapiens OX=9606 GN=CAPG PE=1 SV=2	38.5	6.19	CAPG
P00367	Glutamate dehydrogenase 1, mitochondrial OS=Homo sapiens OX=9606 GN=GLUD1 PE=1 SV=2	61.4	7.8	GLUD1
P09493	Tropomyosin alpha-1 chain OS=Homo sapiens OX=9606 GN=TPM1 PE=1 SV=2	32.7	4.74	TPM1

P51148	Ras-related protein Rab-5C OS=Homo sapiens OX=9606 GN=RAB5C PE=1 SV=2	23.5	8.41	RAB5C
P28331	NADH-ubiquinone oxidoreductase 75 kDa subunit, mitochondrial OS=Homo sapiens OX=9606 GN=NDUFS1 PE=1 SV=3	79.4	6.23	NDUFS1
P15144	Aminopeptidase N OS=Homo sapiens OX=9606 GN=ANPEP PE=1 SV=4	109.5	5.48	ANPEP
P22695	Cytochrome b-c1 complex subunit 2, mitochondrial OS=Homo sapiens OX=9606 GN=UQCRC2 PE=1 SV=3	48.4	8.63	UQCRC2
P18124	60S ribosomal protein L7 OS=Homo sapiens OX=9606 GN=RPL7 PE=1 SV=1	29.2	10.65	RPL7
O75643	U5 small nuclear ribonucleoprotein 200 kDa helicase OS=Homo sapiens OX=9606 GN=SNRNP200 PE=1 SV=2	244.4	6.06	SNRNP200
Q15029	116 kDa U5 small nuclear ribonucleoprotein component OS=Homo sapiens OX=9606 GN=EFTUD2 PE=1 SV=1	109.4	5	EFTUD2
P08133	Annexin A6 OS=Homo sapiens OX=9606 GN=ANXA6 PE=1 SV=3	75.8	5.6	ANXA6
O15144	Actin-related protein 2/3 complex subunit 2 OS=Homo sapiens OX=9606 GN=ARPC2 PE=1 SV=1	34.3	7.36	ARPC2
Q14914	Prostaglandin reductase 1 OS=Homo sapiens OX=9606 GN=PTGR1 PE=1 SV=2	35.8	8.29	PTGR1
P14866	Heterogeneous nuclear ribonucleoprotein L OS=Homo sapiens OX=9606 GN=HNRNPL PE=1 SV=2	64.1	8.22	HNRNPL
P50453	Serpin B9 OS=Homo sapiens OX=9606 GN=SERPINB9 PE=1 SV=1	42.4	5.86	SERPINB9
P23284	Peptidyl-prolyl cis-trans isomerase B OS=Homo sapiens OX=9606 GN=PPIB PE=1 SV=2	23.7	9.41	PPIB
Q13740	CD166 antigen OS=Homo sapiens OX=9606 GN=ALCAM PE=1 SV=2	65.1	6.25	ALCAM
P26006	Integrin alpha-3 OS=Homo sapiens OX=9606 GN=ITGA3 PE=1 SV=5	116.5	6.77	ITGA3
P12236	ADP/ATP translocase 3 OS=Homo sapiens OX=9606 GN=SLC25A6 PE=1 SV=4	32.8	9.74	SLC25A6

Q9UNZ2	NSFL1 cofactor p47 OS=Homo sapiens OX=9606 GN=NSFL1C PE=1 SV=2	40.5	5.1	NSFL1C
P02786	Transferrin receptor protein 1 OS=Homo sapiens OX=9606 GN=TFRC PE=1 SV=2	84.8	6.61	TFRC
Q99623	Prohibitin-2 OS=Homo sapiens OX=9606 GN=PHB2 PE=1 SV=2	33.3	9.83	PHB2
P56192	Methionine--tRNA ligase, cytoplasmic OS=Homo sapiens OX=9606 GN=MARS1 PE=1 SV=2	101.1	6.16	MARS1
Q16181	Septin-7 OS=Homo sapiens OX=9606 GN=SEPTIN7 PE=1 SV=2	50.6	8.63	SEPTIN7
P25787	Proteasome subunit alpha type-2 OS=Homo sapiens OX=9606 GN=PSMA2 PE=1 SV=2	25.9	7.43	PSMA2
Q14103	Heterogeneous nuclear ribonucleoprotein D0 OS=Homo sapiens OX=9606 GN=HNRNPD PE=1 SV=1	38.4	7.81	HNRNPD
P17844	Probable ATP-dependent RNA helicase DDX5 OS=Homo sapiens OX=9606 GN=DDX5 PE=1 SV=1	69.1	8.92	DDX5
Q15274	Nicotinate-nucleotide pyrophosphorylase [carboxylating] OS=Homo sapiens OX=9606 GN=QPRT PE=1 SV=3	30.8	6.21	QPRT
P16403	Histone H1.2 OS=Homo sapiens OX=9606 GN=H1-2 PE=1 SV=2	21.4	10.93	H1-2
P32119	Peroxiredoxin-2 OS=Homo sapiens OX=9606 GN=PRDX2 PE=1 SV=5	21.9	5.97	PRDX2
P05141	ADP/ATP translocase 2 OS=Homo sapiens OX=9606 GN=SLC25A5 PE=1 SV=7	32.8	9.69	SLC25A5
P12429	Annexin A3 OS=Homo sapiens OX=9606 GN=ANXA3 PE=1 SV=3	36.4	5.92	ANXA3
Q9UNM6	26S proteasome non-ATPase regulatory subunit 13 OS=Homo sapiens OX=9606 GN=PSMD13 PE=1 SV=2	42.9	5.81	PSMD13
P54577	Tyrosine--tRNA ligase, cytoplasmic OS=Homo sapiens OX=9606 GN=YARS1 PE=1 SV=4	59.1	7.05	YARS1
P47756	F-actin-capping protein subunit beta OS=Homo sapiens OX=9606 GN=CAPZB PE=1 SV=4	31.3	5.59	CAPZB

P46777	60S ribosomal protein L5 OS=Homo sapiens OX=9606 GN=RPL5 PE=1 SV=3	34.3	9.72	RPL5
P50995	Annexin A11 OS=Homo sapiens OX=9606 GN=ANXA11 PE=1 SV=1	54.4	7.65	ANXA11
Q9ULV4	Coronin-1C OS=Homo sapiens OX=9606 GN=CORO1C PE=1 SV=1	53.2	7.08	CORO1C
Q8N1G4	Leucine-rich repeat-containing protein 47 OS=Homo sapiens OX=9606 GN=LRRC47 PE=1 SV=1	63.4	8.28	LRRC47
Q99536	Synaptic vesicle membrane protein VAT-1 homolog OS=Homo sapiens OX=9606 GN=VAT1 PE=1 SV=2	41.9	6.29	VAT1
P80723	Brain acid soluble protein 1 OS=Homo sapiens OX=9606 GN=BASP1 PE=1 SV=2	22.7	4.63	BASP1
Q9Y277	Voltage-dependent anion-selective channel protein 3 OS=Homo sapiens OX=9606 GN=VDAC3 PE=1 SV=1	30.6	8.66	VDAC3
Q6NZI2	Caveolae-associated protein 1 OS=Homo sapiens OX=9606 GN=CAVIN1 PE=1 SV=1	43.5	5.6	CAVIN1
P30419	Glycylpeptide N-tetradecanoyltransferase 1 OS=Homo sapiens OX=9606 GN=NMT1 PE=1 SV=2	56.8	7.8	NMT1
P51665	26S proteasome non-ATPase regulatory subunit 7 OS=Homo sapiens OX=9606 GN=PSMD7 PE=1 SV=2	37	6.77	PSMD7
O00116	Alkyldihydroxyacetonephosphate synthase, peroxisomal OS=Homo sapiens OX=9606 GN=AGPS PE=1 SV=1	72.9	7.34	AGPS
O14818	Proteasome subunit alpha type-7 OS=Homo sapiens OX=9606 GN=PSMA7 PE=1 SV=1	27.9	8.46	PSMA7
Q13435	Splicing factor 3B subunit 2 OS=Homo sapiens OX=9606 GN=SF3B2 PE=1 SV=2	100.2	5.67	SF3B2
Q9P2J5	Leucine--tRNA ligase, cytoplasmic OS=Homo sapiens OX=9606 GN=LARS1 PE=1 SV=2	134.4	7.3	LARS1
P04439	HLA class I histocompatibility antigen, A alpha chain OS=Homo sapiens OX=9606 GN=HLA-A PE=1 SV=2	40.8	6	HLA-A
P04179	Superoxide dismutase [Mn], mitochondrial OS=Homo sapiens OX=9606 GN=SOD2 PE=1 SV=3	24.7	8.25	SOD2

Q9H0U4	Ras-related protein Rab-1B OS=Homo sapiens OX=9606 GN=RAB1B PE=1 SV=1	22.2	5.73	RAB1B
Q6FI13	Histone H2A type 2-A OS=Homo sapiens OX=9606 GN=H2AC18 PE=1 SV=3	14.1	10.9	H2AC18; H2AC19
P61106	Ras-related protein Rab-14 OS=Homo sapiens OX=9606 GN=RAB14 PE=1 SV=4	23.9	6.21	RAB14
O14950	Myosin regulatory light chain 12B OS=Homo sapiens OX=9606 GN=MYL12B PE=1 SV=2	19.8	4.84	MYL12B
P10412	Histone H1.4 OS=Homo sapiens OX=9606 GN=H1-4 PE=1 SV=2	21.9	11.03	H1-4
P24752	Acetyl-CoA acetyltransferase, mitochondrial OS=Homo sapiens OX=9606 GN=ACAT1 PE=1 SV=1	45.2	8.85	ACAT1
Q10567	AP-1 complex subunit beta-1 OS=Homo sapiens OX=9606 GN=AP1B1 PE=1 SV=2	104.6	5.06	
P12270	Nucleoprotein TPR OS=Homo sapiens OX=9606 GN=TPR PE=1 SV=3	267.1	5.02	TPR
P60228	Eukaryotic translation initiation factor 3 subunit E OS=Homo sapiens OX=9606 GN=EIF3E PE=1 SV=1	52.2	6.04	EIF3E
Q9NZN4	EH domain-containing protein 2 OS=Homo sapiens OX=9606 GN=EHD2 PE=1 SV=2	61.1	6.46	EHD2
P11766	Alcohol dehydrogenase class-3 OS=Homo sapiens OX=9606 GN=ADH5 PE=1 SV=4	39.7	7.49	ADH5
Q15691	Microtubule-associated protein RP/EB family member 1 OS=Homo sapiens OX=9606 GN=MAPRE1 PE=1 SV=3	30	5.14	MAPRE1
Q9BWD1	Acetyl-CoA acetyltransferase, cytosolic OS=Homo sapiens OX=9606 GN=ACAT2 PE=1 SV=2	41.3	6.92	ACAT2
P61586	Transforming protein RhoA OS=Homo sapiens OX=9606 GN=RHOA PE=1 SV=1	21.8	6.1	RHOA
P30085	UMP-CMP kinase OS=Homo sapiens OX=9606 GN=CMPK1 PE=1 SV=3	22.2	5.57	CMPK1
P12235	ADP/ATP translocase 1 OS=Homo sapiens OX=9606 GN=SLC25A4 PE=1 SV=4	33	9.76	SLC25A4

Q13283	Ras GTPase-activating protein-binding protein 1 OS=Homo sapiens OX=9606 GN=G3BP1 PE=1 SV=1	52.1	5.52	G3BP1
P48047	ATP synthase subunit O, mitochondrial OS=Homo sapiens OX=9606 GN=ATP5PO PE=1 SV=1	23.3	9.96	ATP5PO
P45880	Voltage-dependent anion-selective channel protein 2 OS=Homo sapiens OX=9606 GN=VDAC2 PE=1 SV=2	31.5	7.56	VDAC2
Q7L7L0	Histone H2A type 3 OS=Homo sapiens OX=9606 GN=H2AW PE=1 SV=3	14.1	11.05	H2AW
Q13177	Serine/threonine-protein kinase PAK 2 OS=Homo sapiens OX=9606 GN=PAK2 PE=1 SV=3	58	5.96	PAK2
Q14008	Cytoskeleton-associated protein 5 OS=Homo sapiens OX=9606 GN=CKAP5 PE=1 SV=3	225.4	7.8	CKAP5
P05198	Eukaryotic translation initiation factor 2 subunit 1 OS=Homo sapiens OX=9606 GN=EIF2S1 PE=1 SV=3	36.1	5.08	EIF2S1
P28482	Mitogen-activated protein kinase 1 OS=Homo sapiens OX=9606 GN=MAPK1 PE=1 SV=3	41.4	6.98	MAPK1
Q99829	Copine-1 OS=Homo sapiens OX=9606 GN=CPNE1 PE=1 SV=1	59	5.83	CPNE1
P62820	Ras-related protein Rab-1A OS=Homo sapiens OX=9606 GN=RAB1A PE=1 SV=3	22.7	6.21	RAB1A
P07384	Calpain-1 catalytic subunit OS=Homo sapiens OX=9606 GN=CAPN1 PE=1 SV=1	81.8	5.67	CAPN1
Q15181	Inorganic pyrophosphatase OS=Homo sapiens OX=9606 GN=PPA1 PE=1 SV=2	32.6	5.86	PPA1
P62906	60S ribosomal protein L10a OS=Homo sapiens OX=9606 GN=RPL10A PE=1 SV=2	24.8	9.94	RPL10A
Q92499	ATP-dependent RNA helicase DDX1 OS=Homo sapiens OX=9606 GN=DDX1 PE=1 SV=2	82.4	7.23	DDX1
Q9Y696	Chloride intracellular channel protein 4 OS=Homo sapiens OX=9606 GN=CLIC4 PE=1 SV=4	28.8	5.59	CLIC4
Q14847	LIM and SH3 domain protein 1 OS=Homo sapiens OX=9606 GN=LASP1 PE=1 SV=2	29.7	7.05	LASP1

O94874	E3 UFM1-protein ligase 1 OS=Homo sapiens OX=9606 GN=UFL1 PE=1 SV=2	89.5	6.79	UFL1
P40261	Nicotinamide N-methyltransferase OS=Homo sapiens OX=9606 GN=NNMT PE=1 SV=1	29.6	5.74	NNMT
O94979	Protein transport protein Sec31A OS=Homo sapiens OX=9606 GN=SEC31A PE=1 SV=3	132.9	6.89	SEC31A
Q16851	UTP--glucose-1-phosphate uridylyltransferase OS=Homo sapiens OX=9606 GN=UGP2 PE=1 SV=5	56.9	8.15	UGP2
Q9NZB2	Constitutive coactivator of PPAR-gamma-like protein 1 OS=Homo sapiens OX=9606 GN=FAM120A PE=1 SV=2	121.8	8.88	FAM120A
P16402	Histone H1.3 OS=Homo sapiens OX=9606 GN=H1-3 PE=1 SV=2	22.3	11.02	H1-3
P55084	Trifunctional enzyme subunit beta, mitochondrial OS=Homo sapiens OX=9606 GN=HADHB PE=1 SV=3	51.3	9.41	HADHB
P61019	Ras-related protein Rab-2A OS=Homo sapiens OX=9606 GN=RAB2A PE=1 SV=1	23.5	6.54	RAB2A
P62241	40S ribosomal protein S8 OS=Homo sapiens OX=9606 GN=RPS8 PE=1 SV=2	24.2	10.32	RPS8
Q9H4M9	EH domain-containing protein 1 OS=Homo sapiens OX=9606 GN=EHD1 PE=1 SV=2	60.6	6.83	EHD1
O95831	Apoptosis-inducing factor 1, mitochondrial OS=Homo sapiens OX=9606 GN=AIFM1 PE=1 SV=1	66.9	8.95	AIFM1
P18085	ADP-ribosylation factor 4 OS=Homo sapiens OX=9606 GN=ARF4 PE=1 SV=3	20.5	7.14	ARF4
P13804	Electron transfer flavoprotein subunit alpha, mitochondrial OS=Homo sapiens OX=9606 GN=ETFA PE=1 SV=1	35.1	8.38	ETFA
Q14203	Dynactin subunit 1 OS=Homo sapiens OX=9606 GN=DCTN1 PE=1 SV=3	141.6	5.81	DCTN1
Q9Y281	Cofilin-2 OS=Homo sapiens OX=9606 GN=CFL2 PE=1 SV=1	18.7	7.88	CFL2
Q92614	Unconventional myosin-XVIIIa OS=Homo sapiens OX=9606 GN=MYO18A PE=1 SV=3	233	6.3	MYO18A

Q9UHG3	Prenylcysteine oxidase 1 OS=Homo sapiens OX=9606 GN=PCYOX1 PE=1 SV=3	56.6	6.18	PCYOX1
Q9Y678	Coatomer subunit gamma-1 OS=Homo sapiens OX=9606 GN=COPG1 PE=1 SV=1	97.7	5.47	COPG1
Q9NTK5	Obg-like ATPase 1 OS=Homo sapiens OX=9606 GN=OLA1 PE=1 SV=2	44.7	7.81	OLA1
P26583	High mobility group protein B2 OS=Homo sapiens OX=9606 GN=HMGB2 PE=1 SV=2	24	7.81	HMGB2
P04181	Ornithine aminotransferase, mitochondrial OS=Homo sapiens OX=9606 GN=OAT PE=1 SV=1	48.5	7.03	OAT
P15559	NAD(P)H dehydrogenase [quinone] 1 OS=Homo sapiens OX=9606 GN=NQO1 PE=1 SV=1	30.8	8.88	NQO1
P35637	RNA-binding protein FUS OS=Homo sapiens OX=9606 GN=FUS PE=1 SV=1	53.4	9.36	FUS
Q13310	Polyadenylate-binding protein 4 OS=Homo sapiens OX=9606 GN=PABPC4 PE=1 SV=1	70.7	9.26	PABPC4
Q9UM54	Unconventional myosin-VI OS=Homo sapiens OX=9606 GN=MYO6 PE=1 SV=4	149.6	8.53	MYO6
O14617	AP-3 complex subunit delta-1 OS=Homo sapiens OX=9606 GN=AP3D1 PE=1 SV=1	130.1	8.48	AP3D1
P32969	60S ribosomal protein L9 OS=Homo sapiens OX=9606 GN=RPL9 PE=1 SV=1	21.9	9.95	RPL9; RPL9P7; RPL9P8; RPL9P9
O75436	Vacuolar protein sorting-associated protein 26A OS=Homo sapiens OX=9606 GN=VPS26A PE=1 SV=2	38.1	6.57	VPS26A
P61204	ADP-ribosylation factor 3 OS=Homo sapiens OX=9606 GN=ARF3 PE=1 SV=2	20.6	7.43	ARF3
P62424	60S ribosomal protein L7a OS=Homo sapiens OX=9606 GN=RPL7A PE=1 SV=2	30	10.61	RPL7A
P16144	Integrin beta-4 OS=Homo sapiens OX=9606 GN=ITGB4 PE=1 SV=5	202	6.09	ITGB4
Q9H0D6	5'-3' exoribonuclease 2 OS=Homo sapiens OX=9606 GN=XRN2 PE=1 SV=1	108.5	7.47	XRN2

Q9UHX1	Poly(U)-binding-splicing factor PUF60 OS=Homo sapiens OX=9606 GN=PUF60 PE=1 SV=1	59.8	5.29	PUF60
P52565	Rho GDP-dissociation inhibitor 1 OS=Homo sapiens OX=9606 GN=ARHGDIA PE=1 SV=3	23.2	5.11	ARHGDIA
P25786	Proteasome subunit alpha type-1 OS=Homo sapiens OX=9606 GN=PSMA1 PE=1 SV=1	29.5	6.61	PSMA1
O95782	AP-2 complex subunit alpha-1 OS=Homo sapiens OX=9606 GN=AP2A1 PE=1 SV=3	107.5	7.03	AP2A1
Q13561	Dynactin subunit 2 OS=Homo sapiens OX=9606 GN=DCTN2 PE=1 SV=4	44.2	5.21	DCTN2
P12268	Inosine-5'-monophosphate dehydrogenase 2 OS=Homo sapiens OX=9606 GN=IMPDH2 PE=1 SV=2	55.8	6.9	IMPDH2
P15880	40S ribosomal protein S2 OS=Homo sapiens OX=9606 GN=RPS2 PE=1 SV=2	31.3	10.24	RPS2
Q02809	Procollagen-lysine,2-oxoglutarate 5-dioxygenase 1 OS=Homo sapiens OX=9606 GN=PLOD1 PE=1 SV=2	83.5	6.95	PLOD1
Q9BYX7	Putative beta-actin-like protein 3 OS=Homo sapiens OX=9606 GN=POTEKP PE=5 SV=1	42	6.33	POTEKP
P62873	Guanine nucleotide-binding protein G(I)/G(S)/G(T) subunit beta-1 OS=Homo sapiens OX=9606 GN=GNB1 PE=1 SV=3	37.4	6	GNB1
Q16401	26S proteasome non-ATPase regulatory subunit 5 OS=Homo sapiens OX=9606 GN=PSMD5 PE=1 SV=3	56.2	5.48	PSMD5
Q9Y3I0	RNA-splicing ligase RtcB homolog OS=Homo sapiens OX=9606 GN=RTCB PE=1 SV=1	55.2	7.23	RTCB
Q13162	Peroxiredoxin-4 OS=Homo sapiens OX=9606 GN=PRDX4 PE=1 SV=1	30.5	6.29	PRDX4
Q16647	Prostacyclin synthase OS=Homo sapiens OX=9606 GN=PTGIS PE=1 SV=1	57.1	7.31	PTGIS
Q86V81	THO complex subunit 4 OS=Homo sapiens OX=9606 GN=ALYREF PE=1 SV=3	26.9	11.15	ALYREF
P20810	Calpastatin OS=Homo sapiens OX=9606 GN=CAST PE=1 SV=4	76.5	5.07	CAST

Q15393	Splicing factor 3B subunit 3 OS=Homo sapiens OX=9606 GN=SF3B3 PE=1 SV=4	135.5	5.26	SF3B3
P14314	Glucosidase 2 subunit beta OS=Homo sapiens OX=9606 GN=PRKCSH PE=1 SV=2	59.4	4.41	PRKCSH
P46782	40S ribosomal protein S5 OS=Homo sapiens OX=9606 GN=RPS5 PE=1 SV=4	22.9	9.72	RPS5
P52907	F-actin-capping protein subunit alpha-1 OS=Homo sapiens OX=9606 GN=CAPZA1 PE=1 SV=3	32.9	5.69	CAPZA1
Q15366	Poly(rC)-binding protein 2 OS=Homo sapiens OX=9606 GN=PCBP2 PE=1 SV=1	38.6	6.79	PCBP2
Q15459	Splicing factor 3A subunit 1 OS=Homo sapiens OX=9606 GN=SF3A1 PE=1 SV=1	88.8	5.22	SF3A1
P54578	Ubiquitin carboxyl-terminal hydrolase 14 OS=Homo sapiens OX=9606 GN=USP14 PE=1 SV=3	56	5.3	USP14
Q13308	Inactive tyrosine-protein kinase 7 OS=Homo sapiens OX=9606 GN=PTK7 PE=1 SV=2	118.3	7.09	PTK7
O43399	Tumor protein D54 OS=Homo sapiens OX=9606 GN=TPD52L2 PE=1 SV=2	22.2	5.36	TPD52L2
Q9NUQ9	CYFIP-related Rac1 interactor B OS=Homo sapiens OX=9606 GN=CYRIB PE=1 SV=1	36.7	6.06	CYRIB
Q9Y617	Phosphoserine aminotransferase OS=Homo sapiens OX=9606 GN=PSAT1 PE=1 SV=2	40.4	7.66	PSAT1
O00148	ATP-dependent RNA helicase DDX39A OS=Homo sapiens OX=9606 GN=DDX39A PE=1 SV=2	49.1	5.68	DDX39A
Q96I24	Far upstream element-binding protein 3 OS=Homo sapiens OX=9606 GN=FUBP3 PE=1 SV=2	61.6	8.38	FUBP3
P49773	Adenosine 5'-monophosphoramidase HINT1 OS=Homo sapiens OX=9606 GN=HINT1 PE=1 SV=2	13.8	6.95	HINT1
P45974	Ubiquitin carboxyl-terminal hydrolase 5 OS=Homo sapiens OX=9606 GN=USP5 PE=1 SV=2	95.7	5.03	USP5
P09104	Gamma-enolase OS=Homo sapiens OX=9606 GN=ENO2 PE=1 SV=3	47.2	5.03	ENO2

Q14315	Filamin-C OS=Homo sapiens OX=9606 GN=FLNC PE=1 SV=3	290.8	5.97	FLNC
Q5VYK3	Proteasome adapter and scaffold protein ECM29 OS=Homo sapiens OX=9606 GN=ECPAS PE=1 SV=2	204.2	7.12	ECPAS
Q13045	Protein flightless-1 homolog OS=Homo sapiens OX=9606 GN=FLII PE=1 SV=2	144.7	6.05	FLII
Q01105	Protein SET OS=Homo sapiens OX=9606 GN=SET PE=1 SV=3	33.5	4.32	SET
Q00325	Phosphate carrier protein, mitochondrial OS=Homo sapiens OX=9606 GN=SLC25A3 PE=1 SV=2	40.1	9.38	SLC25A3
P62277	40S ribosomal protein S13 OS=Homo sapiens OX=9606 GN=RPS13 PE=1 SV=2	17.2	10.54	RPS13
P61158	Actin-related protein 3 OS=Homo sapiens OX=9606 GN=ACTR3 PE=1 SV=3	47.3	5.88	ACTR3
P42167	Lamina-associated polypeptide 2, isoforms beta/gamma OS=Homo sapiens OX=9606 GN=TMPO PE=1 SV=2	50.6	9.38	TMPO
Q9Y6M1	Insulin-like growth factor 2 mRNA-binding protein 2 OS=Homo sapiens OX=9606 GN=IGF2BP2 PE=1 SV=2	66.1	8.46	IGF2BP2
O60841	Eukaryotic translation initiation factor 5B OS=Homo sapiens OX=9606 GN=EIF5B PE=1 SV=4	138.7	5.49	EIF5B
Q9Y2A7	Nck-associated protein 1 OS=Homo sapiens OX=9606 GN=NCKAP1 PE=1 SV=1	128.7	6.62	NCKAP1
P00505	Aspartate aminotransferase, mitochondrial OS=Homo sapiens OX=9606 GN=GOT2 PE=1 SV=3	47.5	9.01	GOT2
P38159	RNA-binding motif protein, X chromosome OS=Homo sapiens OX=9606 GN=RBMX PE=1 SV=3	42.3	10.05	RBMX
Q15293	Reticulocalbin-1 OS=Homo sapiens OX=9606 GN=RCN1 PE=1 SV=1	38.9	5	RCN1
P53992	Protein transport protein Sec24C OS=Homo sapiens OX=9606 GN=SEC24C PE=1 SV=3	118.2	7.06	SEC24C
P06756	Integrin alpha-V OS=Homo sapiens OX=9606 GN=ITGAV PE=1 SV=2	116	5.68	ITGAV

P62333	26S proteasome regulatory subunit 10B OS=Homo sapiens OX=9606 GN=PSMC6 PE=1 SV=1	44.1	7.49	PSMC6
P30044	Peroxiredoxin-5, mitochondrial OS=Homo sapiens OX=9606 GN=PRDX5 PE=1 SV=4	22.1	8.7	PRDX5
P62136	Serine/threonine-protein phosphatase PP1-alpha catalytic subunit OS=Homo sapiens OX=9606 GN=PPP1CA PE=1 SV=1	37.5	6.33	PPP1CA
P60660	Myosin light polypeptide 6 OS=Homo sapiens OX=9606 GN=MYL6 PE=1 SV=2	16.9	4.65	MYL6
Q8NBS9	Thioredoxin domain-containing protein 5 OS=Homo sapiens OX=9606 GN=TXNDC5 PE=1 SV=2	47.6	5.97	TXNDC5
P27695	DNA-(apurinic or apyrimidinic site) endonuclease OS=Homo sapiens OX=9606 GN=APEX1 PE=1 SV=2	35.5	8.12	APEX1
Q9UHB9	Signal recognition particle subunit SRP68 OS=Homo sapiens OX=9606 GN=SRP68 PE=1 SV=2	70.7	8.56	SRP68
P08134	Rho-related GTP-binding protein RhoC OS=Homo sapiens OX=9606 GN=RHOC PE=1 SV=1	22	6.58	RHOC
P09382	Galectin-1 OS=Homo sapiens OX=9606 GN=LGALS1 PE=1 SV=2	14.7	5.5	LGALS1
Q14376	UDP-glucose 4-epimerase OS=Homo sapiens OX=9606 GN=GALE PE=1 SV=2	38.3	6.73	GALE
Q9UQE7	Structural maintenance of chromosomes protein 3 OS=Homo sapiens OX=9606 GN=SMC3 PE=1 SV=2	141.5	7.18	SMC3
P52943	Cysteine-rich protein 2 OS=Homo sapiens OX=9606 GN=CRIP2 PE=1 SV=1	22.5	8.72	CRIP2
Q14839	Chromodomain-helicase-DNA-binding protein 4 OS=Homo sapiens OX=9606 GN=CHD4 PE=1 SV=2	217.9	5.86	CHD4
P04040	Catalase OS=Homo sapiens OX=9606 GN=CAT PE=1 SV=3	59.7	7.39	CAT
P31930	Cytochrome b-c1 complex subunit 1, mitochondrial OS=Homo sapiens OX=9606 GN=UQCRC1 PE=1 SV=3	52.6	6.37	UQCRC1
Q15907	Ras-related protein Rab-11B OS=Homo sapiens OX=9606 GN=RAB11B PE=1 SV=4	24.5	5.94	RAB11B

P20042	Eukaryotic translation initiation factor 2 subunit 2 OS=Homo sapiens OX=9606 GN=EIF2S2 PE=1 SV=2	38.4	5.8	EIF2S2
P41250	Glycine--tRNA ligase OS=Homo sapiens OX=9606 GN=GARS1 PE=1 SV=3	83.1	7.03	GARS1
Q9Y266	Nuclear migration protein nudC OS=Homo sapiens OX=9606 GN=NUDC PE=1 SV=1	38.2	5.38	NUDC
P48735	Isocitrate dehydrogenase [NADP], mitochondrial OS=Homo sapiens OX=9606 GN=IDH2 PE=1 SV=2	50.9	8.69	IDH2
P60981	Destrin OS=Homo sapiens OX=9606 GN=DSTN PE=1 SV=3	18.5	7.85	DSTN
P62081	40S ribosomal protein S7 OS=Homo sapiens OX=9606 GN=RPS7 PE=1 SV=1	22.1	10.1	RPS7
Q9NR46	Endophilin-B2 OS=Homo sapiens OX=9606 GN=SH3GLB2 PE=1 SV=1	43.9	5.99	SH3GLB2
P22102	Trifunctional purine biosynthetic protein adenosine-3 OS=Homo sapiens OX=9606 GN=GART PE=1 SV=1	107.7	6.7	GART
P31937	3-hydroxyisobutyrate dehydrogenase, mitochondrial OS=Homo sapiens OX=9606 GN=HIBADH PE=1 SV=2	35.3	8.13	HIBADH
Q13409	Cytoplasmic dynein 1 intermediate chain 2 OS=Homo sapiens OX=9606 GN=DYNC1I2 PE=1 SV=3	71.4	5.2	DYNC1I2
P30050	60S ribosomal protein L12 OS=Homo sapiens OX=9606 GN=RPL12 PE=1 SV=1	17.8	9.42	RPL12
P16104	Histone H2AX OS=Homo sapiens OX=9606 GN=H2AX PE=1 SV=2	15.1	10.74	H2AX
Q13148	TAR DNA-binding protein 43 OS=Homo sapiens OX=9606 GN=TARDBP PE=1 SV=1	44.7	6.19	TARDBP
Q8TAT6	Nuclear protein localization protein 4 homolog OS=Homo sapiens OX=9606 GN=NPLOC4 PE=1 SV=3	68.1	6.38	NPLOC4
P16949	Stathmin OS=Homo sapiens OX=9606 GN=STMN1 PE=1 SV=3	17.3	5.97	STMN1
P09972	Fructose-bisphosphate aldolase C OS=Homo sapiens OX=9606 GN=ALDOC PE=1 SV=2	39.4	6.87	ALDOC

O95373	Importin-7 OS=Homo sapiens OX=9606 GN=IPO7 PE=1 SV=1	119.4	4.82	IPO7
O76094	Signal recognition particle subunit SRP72 OS=Homo sapiens OX=9606 GN=SRP72 PE=1 SV=3	74.6	9.26	SRP72
P61313	60S ribosomal protein L15 OS=Homo sapiens OX=9606 GN=RPL15 PE=1 SV=2	24.1	11.62	RPL15
Q14444	Caprin-1 OS=Homo sapiens OX=9606 GN=CAPRIN1 PE=1 SV=2	78.3	5.25	CAPRIN1
P84085	ADP-ribosylation factor 5 OS=Homo sapiens OX=9606 GN=ARF5 PE=1 SV=2	20.5	6.79	ARF5
P68400	Casein kinase II subunit alpha OS=Homo sapiens OX=9606 GN=CSNK2A1 PE=1 SV=1	45.1	7.74	CSNK2A1
Q12792	Twinfilin-1 OS=Homo sapiens OX=9606 GN=TWF1 PE=1 SV=3	40.3	6.96	TWF1
O94973	AP-2 complex subunit alpha-2 OS=Homo sapiens OX=9606 GN=AP2A2 PE=1 SV=2	103.9	6.96	AP2A2
P20073	Annexin A7 OS=Homo sapiens OX=9606 GN=ANXA7 PE=1 SV=3	52.7	5.68	ANXA7
Q9NSE4	Isoleucine--tRNA ligase, mitochondrial OS=Homo sapiens OX=9606 GN=IARS2 PE=1 SV=2	113.7	7.2	IARS2
P30740	Leukocyte elastase inhibitor OS=Homo sapiens OX=9606 GN=SERPINB1 PE=1 SV=1	42.7	6.28	SERPINB1
Q16543	Hsp90 co-chaperone Cdc37 OS=Homo sapiens OX=9606 GN=CDC37 PE=1 SV=1	44.4	5.25	CDC37
P49755	Transmembrane emp24 domain-containing protein 10 OS=Homo sapiens OX=9606 GN=TMED10 PE=1 SV=2	25	7.44	TMED10
P06493	Cyclin-dependent kinase 1 OS=Homo sapiens OX=9606 GN=CDK1 PE=1 SV=3	34.1	8.4	CDK1
Q14157	Ubiquitin-associated protein 2-like OS=Homo sapiens OX=9606 GN=UBAP2L PE=1 SV=2	114.5	7.11	UBAP2L
P51114	Fragile X mental retardation syndrome-related protein 1 OS=Homo sapiens OX=9606 GN=FXR1 PE=1 SV=3	69.7	6.15	FXR1

P04899	Guanine nucleotide-binding protein G(i) subunit alpha-2 OS=Homo sapiens OX=9606 GN=GNAI2 PE=1 SV=3	40.4	5.54	GNAI2
O75390	Citrate synthase, mitochondrial OS=Homo sapiens OX=9606 GN=CS PE=1 SV=2	51.7	8.32	CS
O75131	Copine-3 OS=Homo sapiens OX=9606 GN=CPNE3 PE=1 SV=1	60.1	5.85	CPNE3
Q7Z406	Myosin-14 OS=Homo sapiens OX=9606 GN=MYH14 PE=1 SV=2	227.7	5.6	MYH14
P16989	Y-box-binding protein 3 OS=Homo sapiens OX=9606 GN=YBX3 PE=1 SV=4	40.1	9.77	YBX3
P17655	Calpain-2 catalytic subunit OS=Homo sapiens OX=9606 GN=CAPN2 PE=1 SV=6	79.9	4.98	CAPN2
Q14258	E3 ubiquitin/ISG15 ligase TRIM25 OS=Homo sapiens OX=9606 GN=TRIM25 PE=1 SV=2	70.9	8.09	TRIM25
Q9P0L0	Vesicle-associated membrane protein-associated protein A OS=Homo sapiens OX=9606 GN=VAPA PE=1 SV=3	27.9	8.62	VAPA
Q02790	Peptidyl-prolyl cis-trans isomerase FKBP4 OS=Homo sapiens OX=9606 GN=FKBP4 PE=1 SV=3	51.8	5.43	FKBP4
Q93009	Ubiquitin carboxyl-terminal hydrolase 7 OS=Homo sapiens OX=9606 GN=USP7 PE=1 SV=2	128.2	5.55	USP7
Q5JRX3	Presequence protease, mitochondrial OS=Homo sapiens OX=9606 GN=PITRM1 PE=1 SV=3	117.3	6.92	PITRM1
P49321	Nuclear autoantigenic sperm protein OS=Homo sapiens OX=9606 GN=NASP PE=1 SV=2	85.2	4.3	NASP
P35232	Prohibitin OS=Homo sapiens OX=9606 GN=PHB PE=1 SV=1	29.8	5.76	PHB1
P42166	Lamina-associated polypeptide 2, isoform alpha OS=Homo sapiens OX=9606 GN=TMPO PE=1 SV=2	75.4	7.66	TMPO
Q32MZ4	Leucine-rich repeat flightless-interacting protein 1 OS=Homo sapiens OX=9606 GN=LRRFIP1 PE=1 SV=2	89.2	4.65	LRRFIP1
O43684	Mitotic checkpoint protein BUB3 OS=Homo sapiens OX=9606 GN=BUB3 PE=1 SV=1	37.1	6.84	BUB3

Q9H223	EH domain-containing protein 4 OS=Homo sapiens OX=9606 GN=EHD4 PE=1 SV=1	61.1	6.76	EHD4
P27635	60S ribosomal protein L10 OS=Homo sapiens OX=9606 GN=RPL10 PE=1 SV=4	24.6	10.08	LOC100768740; LOC101344695; LOC105294701; LOC112632580; Rpl10; RPL10
P22061	Protein-L-isoaspartate(D-aspartate) O-methyltransferase OS=Homo sapiens OX=9606 GN=PCMT1 PE=1 SV=4	24.6	7.21	PCMT1
O00154	Cytosolic acyl coenzyme A thioester hydrolase OS=Homo sapiens OX=9606 GN=ACOT7 PE=1 SV=3	41.8	8.54	ACOT7
Q06323	Proteasome activator complex subunit 1 OS=Homo sapiens OX=9606 GN=PSME1 PE=1 SV=1	28.7	6.02	PSME1
O94905	Erlin-2 OS=Homo sapiens OX=9606 GN=ERLIN2 PE=1 SV=1	37.8	5.62	ERLIN2
Q9UL46	Proteasome activator complex subunit 2 OS=Homo sapiens OX=9606 GN=PSME2 PE=1 SV=4	27.4	5.73	PSME2
P08708	40S ribosomal protein S17 OS=Homo sapiens OX=9606 GN=RPS17 PE=1 SV=2	15.5	9.85	RPS17
P28070	Proteasome subunit beta type-4 OS=Homo sapiens OX=9606 GN=PSMB4 PE=1 SV=4	29.2	5.97	PSMB4
Q96FW1	Ubiquitin thioesterase OTUB1 OS=Homo sapiens OX=9606 GN=OTUB1 PE=1 SV=2	31.3	4.94	OTUB1
P62495	Eukaryotic peptide chain release factor subunit 1 OS=Homo sapiens OX=9606 GN=ETF1 PE=1 SV=3	49	5.71	ETF1
P38606	V-type proton ATPase catalytic subunit A OS=Homo sapiens OX=9606 GN=ATP6V1A PE=1 SV=2	68.3	5.52	ATP6V1A
P13929	Beta-enolase OS=Homo sapiens OX=9606 GN=ENO3 PE=1 SV=5	47	7.71	ENO3
P62826	GTP-binding nuclear protein Ran OS=Homo sapiens OX=9606 GN=RAN PE=1 SV=3	24.4	7.49	RAN
P18621	60S ribosomal protein L17 OS=Homo sapiens OX=9606 GN=RPL17 PE=1 SV=3	21.4	10.17	RPL17
P51610	Host cell factor 1 OS=Homo sapiens OX=9606 GN=HCFC1 PE=1 SV=2	208.6	7.46	HCFC1

P62753	40S ribosomal protein S6 OS=Homo sapiens OX=9606 GN=RPS6 PE=1 SV=1	28.7	10.84	RPS6
P39656	Dolichyl-diphosphooligosaccharide--protein glycosyltransferase 48 kDa subunit OS=Homo sapiens OX=9606 GN=DDOST PE=1 SV=4	50.8	6.55	DDOST
P63220	40S ribosomal protein S21 OS=Homo sapiens OX=9606 GN=RPS21 PE=1 SV=1	9.1	8.5	RPS21
Q9Y2T3	Guanine deaminase OS=Homo sapiens OX=9606 GN=GDA PE=1 SV=1	51	5.68	GDA
O00487	26S proteasome non-ATPase regulatory subunit 14 OS=Homo sapiens OX=9606 GN=PSMD14 PE=1 SV=1	34.6	6.52	PSMD14
Q6DD88	Atlastin-3 OS=Homo sapiens OX=9606 GN=ATL3 PE=1 SV=1	60.5	5.66	ATL3
P13489	Ribonuclease inhibitor OS=Homo sapiens OX=9606 GN=RNH1 PE=1 SV=2	49.9	4.82	RNH1
Q96CW1	AP-2 complex subunit mu OS=Homo sapiens OX=9606 GN=AP2M1 PE=1 SV=2	49.6	9.54	AP2M1
P26196	Probable ATP-dependent RNA helicase DDX6 OS=Homo sapiens OX=9606 GN=DDX6 PE=1 SV=2	54.4	8.66	DDX6
P54819	Adenylate kinase 2, mitochondrial OS=Homo sapiens OX=9606 GN=AK2 PE=1 SV=2	26.5	7.81	AK2
Q9H6Z4	Ran-binding protein 3 OS=Homo sapiens OX=9606 GN=RANBP3 PE=1 SV=1	60.2	4.78	RANBP3
O14979	Heterogeneous nuclear ribonucleoprotein D-like OS=Homo sapiens OX=9606 GN=HNRNPDL PE=1 SV=3	46.4	9.57	HNRNPDL
Q9P258	Protein RCC2 OS=Homo sapiens OX=9606 GN=RCC2 PE=1 SV=2	56	8.78	RCC2
P04632	Calpain small subunit 1 OS=Homo sapiens OX=9606 GN=CAPNS1 PE=1 SV=1	28.3	5.2	CAPNS1
P04080	Cystatin-B OS=Homo sapiens OX=9606 GN=CSTB PE=1 SV=2	11.1	7.56	CSTB
P52306	Rap1 GTPase-GDP dissociation stimulator 1 OS=Homo sapiens OX=9606 GN=RAP1GDS1 PE=1 SV=3	66.3	5.31	RAP1GDS1

Q07955	Serine/arginine-rich splicing factor 1 OS=Homo sapiens OX=9606 GN=SRSF1 PE=1 SV=2	27.7	10.36	SRSF1
P55884	Eukaryotic translation initiation factor 3 subunit B OS=Homo sapiens OX=9606 GN=EIF3B PE=1 SV=3	92.4	5	EIF3B
P00390	Glutathione reductase, mitochondrial OS=Homo sapiens OX=9606 GN=GSR PE=1 SV=2	56.2	8.5	GSR
P20618	Proteasome subunit beta type-1 OS=Homo sapiens OX=9606 GN=PSMB1 PE=1 SV=2	26.5	8.13	PSMB1
O43237	Cytoplasmic dynein 1 light intermediate chain 2 OS=Homo sapiens OX=9606 GN=DYNC1LI2 PE=1 SV=1	54.1	6.38	DYNC1LI2
O43795	Unconventional myosin-Ib OS=Homo sapiens OX=9606 GN=MYO1B PE=1 SV=3	131.9	9.38	MYO1B
O43396	Thioredoxin-like protein 1 OS=Homo sapiens OX=9606 GN=TXNL1 PE=1 SV=3	32.2	4.96	TXNL1
P62750	60S ribosomal protein L23a OS=Homo sapiens OX=9606 GN=RPL23A PE=1 SV=1	17.7	10.45	RPL23A
P61221	ATP-binding cassette sub-family E member 1 OS=Homo sapiens OX=9606 GN=ABCE1 PE=1 SV=1	67.3	8.34	ABCE1
Q9BT78	COP9 signalosome complex subunit 4 OS=Homo sapiens OX=9606 GN=COPS4 PE=1 SV=1	46.2	5.83	COPS4
A0AVT1	Ubiquitin-like modifier-activating enzyme 6 OS=Homo sapiens OX=9606 GN=UBA6 PE=1 SV=1	117.9	6.14	UBA6
P26368	Splicing factor U2AF 65 kDa subunit OS=Homo sapiens OX=9606 GN=U2AF2 PE=1 SV=4	53.5	9.09	U2AF2
P00966	Argininosuccinate synthase OS=Homo sapiens OX=9606 GN=ASS1 PE=1 SV=2	46.5	8.02	ASS1
P30040	Endoplasmic reticulum resident protein 29 OS=Homo sapiens OX=9606 GN=ERP29 PE=1 SV=4	29	7.31	ERP29
P62140	Serine/threonine-protein phosphatase PP1-beta catalytic subunit OS=Homo sapiens OX=9606 GN=PPP1CB PE=1 SV=3	37.2	6.19	PPP1CB
Q99873	Protein arginine N-methyltransferase 1 OS=Homo sapiens OX=9606 GN=PRMT1 PE=1 SV=3	42.4	5.35	PRMT1

P31040	Succinate dehydrogenase [ubiquinone] flavoprotein subunit, mitochondrial OS=Homo sapiens OX=9606 GN=SDHA PE=1 SV=2	72.6	7.39	SDHA
P80303	Nucleobindin-2 OS=Homo sapiens OX=9606 GN=NUCB2 PE=1 SV=3	50.2	5.12	NUCB2
O60749	Sorting nexin-2 OS=Homo sapiens OX=9606 GN=SNX2 PE=1 SV=2	58.4	5.12	SNX2
O95347	Structural maintenance of chromosomes protein 2 OS=Homo sapiens OX=9606 GN=SMC2 PE=1 SV=2	135.6	8.43	SMC2
P55795	Heterogeneous nuclear ribonucleoprotein H2 OS=Homo sapiens OX=9606 GN=HNRNPH2 PE=1 SV=1	49.2	6.3	HNRNPH2
Q6PIU2	Neutral cholesterol ester hydrolase 1 OS=Homo sapiens OX=9606 GN=NCEH1 PE=1 SV=3	45.8	7.23	NCEH1
P51572	B-cell receptor-associated protein 31 OS=Homo sapiens OX=9606 GN=BCAP31 PE=1 SV=3	28	8.44	BCAP31
O76003	Glutaredoxin-3 OS=Homo sapiens OX=9606 GN=GLRX3 PE=1 SV=2	37.4	5.39	GLRX3
Q9BTW9	Tubulin-specific chaperone D OS=Homo sapiens OX=9606 GN=TBCD PE=1 SV=2	132.5	6.19	TBCD
P60900	Proteasome subunit alpha type-6 OS=Homo sapiens OX=9606 GN=PSMA6 PE=1 SV=1	27.4	6.76	PSMA6
P23193	Transcription elongation factor A protein 1 OS=Homo sapiens OX=9606 GN=TCEA1 PE=1 SV=2	33.9	8.38	TCEA1
O75947	ATP synthase subunit d, mitochondrial OS=Homo sapiens OX=9606 GN=ATP5PD PE=1 SV=3	18.5	5.3	ATP5PD
Q96AG4	Leucine-rich repeat-containing protein 59 OS=Homo sapiens OX=9606 GN=LRRC59 PE=1 SV=1	34.9	9.57	LRRC59
Q13011	Delta(3,5)-Delta(2,4)-dienoyl-CoA isomerase, mitochondrial OS=Homo sapiens OX=9606 GN=ECH1 PE=1 SV=2	35.8	8	ECH1
P08754	Guanine nucleotide-binding protein G(i) subunit alpha-3 OS=Homo sapiens OX=9606 GN=GNAI3 PE=1 SV=3	40.5	5.69	GNAI3

Q96P70	Importin-9 OS=Homo sapiens OX=9606 GN=IPO9 PE=1 SV=3	115.9	4.81	IPO9
P11177	Pyruvate dehydrogenase E1 component subunit beta, mitochondrial OS=Homo sapiens OX=9606 GN=PDHB PE=1 SV=3	39.2	6.65	PDHB
P11498	Pyruvate carboxylase, mitochondrial OS=Homo sapiens OX=9606 GN=PC PE=1 SV=2	129.6	6.84	PC
Q5QNW6	Histone H2B type 2-F OS=Homo sapiens OX=9606 GN=H2BC18 PE=1 SV=3	13.9	10.32	H2BC18
O95202	Mitochondrial proton/calcium exchanger protein OS=Homo sapiens OX=9606 GN=LETM1 PE=1 SV=1	83.3	6.7	LETM1
Q92597	Protein NDRG1 OS=Homo sapiens OX=9606 GN=NDRG1 PE=1 SV=1	42.8	5.82	NDRG1
P46781	40S ribosomal protein S9 OS=Homo sapiens OX=9606 GN=RPS9 PE=1 SV=3	22.6	10.65	RPS9
P61604	10 kDa heat shock protein, mitochondrial OS=Homo sapiens OX=9606 GN=HSPE1 PE=1 SV=2	10.9	8.92	HSPE1
Q1KMD3	Heterogeneous nuclear ribonucleoprotein U-like protein 2 OS=Homo sapiens OX=9606 GN=HNRNPUL2 PE=1 SV=1	85.1	4.91	HNRNPUL2
P61026	Ras-related protein Rab-10 OS=Homo sapiens OX=9606 GN=RAB10 PE=1 SV=1	22.5	8.38	RAB10
Q2TB90	Hexokinase HKDC1 OS=Homo sapiens OX=9606 GN=HKDC1 PE=1 SV=3	102.5	7.12	HKDC1
Q58FF6	Putative heat shock protein HSP 90-beta 4 OS=Homo sapiens OX=9606 GN=HSP90AB4P PE=5 SV=1	58.2	4.73	HSP90AB4P
Q04446	1,4-alpha-glucan-branching enzyme OS=Homo sapiens OX=9606 GN=GBE1 PE=1 SV=3	80.4	6.32	GBE1
P46459	Vesicle-fusing ATPase OS=Homo sapiens OX=9606 GN=NSF PE=1 SV=3	82.5	6.95	NSF
P68036	Ubiquitin-conjugating enzyme E2 L3 OS=Homo sapiens OX=9606 GN=UBE2L3 PE=1 SV=1	17.9	8.51	UBE2L3
P07858	Cathepsin B OS=Homo sapiens OX=9606 GN=CTSB PE=1 SV=3	37.8	6.3	CTSB

Q86UE4	Protein LYRIC OS=Homo sapiens OX=9606 GN=MTDH PE=1 SV=2	63.8	9.32	MTDH
Q9Y3F4	Serine-threonine kinase receptor-associated protein OS=Homo sapiens OX=9606 GN=STRAP PE=1 SV=1	38.4	5.12	STRAP
Q5SSJ5	Heterochromatin protein 1-binding protein 3 OS=Homo sapiens OX=9606 GN=HP1BP3 PE=1 SV=1	61.2	9.67	HP1BP3
P10768	S-formylglutathione hydrolase OS=Homo sapiens OX=9606 GN=ESD PE=1 SV=2	31.4	7.02	ESD
P17931	Galectin-3 OS=Homo sapiens OX=9606 GN=LGALS3 PE=1 SV=5	26.1	8.56	LGALS3
P61224	Ras-related protein Rap-1b OS=Homo sapiens OX=9606 GN=RAP1B PE=1 SV=1	20.8	5.78	RAP1B
P78417	Glutathione S-transferase omega-1 OS=Homo sapiens OX=9606 GN=GSTO1 PE=1 SV=2	27.5	6.6	GSTO1
P19224	UDP-glucuronosyltransferase 1-6 OS=Homo sapiens OX=9606 GN=UGT1A6 PE=1 SV=2	60.7	8.41	UGT1A6
P27361	Mitogen-activated protein kinase 3 OS=Homo sapiens OX=9606 GN=MAPK3 PE=1 SV=4	43.1	6.74	MAPK3
P21980	Protein-glutamine gamma-glutamyltransferase 2 OS=Homo sapiens OX=9606 GN=TGM2 PE=1 SV=2	77.3	5.22	TGM2
Q96HC4	PDZ and LIM domain protein 5 OS=Homo sapiens OX=9606 GN=PDLIM5 PE=1 SV=5	63.9	8.21	PDLIM5
Q9C0C2	182 kDa tankyrase-1-binding protein OS=Homo sapiens OX=9606 GN=TNKS1BP1 PE=1 SV=4	181.7	4.86	TNKS1BP1
P36542	ATP synthase subunit gamma, mitochondrial OS=Homo sapiens OX=9606 GN=ATP5F1C PE=1 SV=1	33	9.22	ATP5F1C
Q16778	Histone H2B type 2-E OS=Homo sapiens OX=9606 GN=H2BC21 PE=1 SV=3	13.9	10.32	H2BC21
Q15637	Splicing factor 1 OS=Homo sapiens OX=9606 GN=SF1 PE=1 SV=4	68.3	8.98	SF1
P35613	Basigin OS=Homo sapiens OX=9606 GN=BSG PE=1 SV=2	42.2	5.66	BSG

Q9UHD1	Cysteine and histidine-rich domain-containing protein 1 OS=Homo sapiens OX=9606 GN=CHORDC1 PE=1 SV=2	37.5	7.87	CHORDC1
Q04695	Keratin, type I cytoskeletal 17 OS=Homo sapiens OX=9606 GN=KRT17 PE=1 SV=2	48.1	5.02	KRT17
P00441	Superoxide dismutase [Cu-Zn] OS=Homo sapiens OX=9606 GN=SOD1 PE=1 SV=2	15.9	6.13	SOD1
P50502	Hsc70-interacting protein OS=Homo sapiens OX=9606 GN=ST13 PE=1 SV=2	41.3	5.27	ST13
P62263	40S ribosomal protein S14 OS=Homo sapiens OX=9606 GN=RPS14 PE=1 SV=3	16.3	10.05	RPS14
Q15631	Translin OS=Homo sapiens OX=9606 GN=TSN PE=1 SV=1	26.2	6.44	TSN
P56537	Eukaryotic translation initiation factor 6 OS=Homo sapiens OX=9606 GN=EIF6 PE=1 SV=1	26.6	4.68	EIF6
P29966	Myristoylated alanine-rich C-kinase substrate OS=Homo sapiens OX=9606 GN=MARCKS PE=1 SV=4	31.5	4.45	MARCKS
P17812	CTP synthase 1 OS=Homo sapiens OX=9606 GN=CTPS1 PE=1 SV=2	66.6	6.46	CTPS1
Q9Y5S2	Serine/threonine-protein kinase MRCK beta OS=Homo sapiens OX=9606 GN=CDC42BPB PE=1 SV=2	194.2	6.37	CDC42BPB
P23588	Eukaryotic translation initiation factor 4B OS=Homo sapiens OX=9606 GN=EIF4B PE=1 SV=2	69.1	5.73	EIF4B
P24539	ATP synthase F(0) complex subunit B1, mitochondrial OS=Homo sapiens OX=9606 GN=ATP5PB PE=1 SV=2	28.9	9.36	ATP5PB
P62841	40S ribosomal protein S15 OS=Homo sapiens OX=9606 GN=RPS15 PE=1 SV=2	17	10.39	RPS15
Q96AC1	Fermitin family homolog 2 OS=Homo sapiens OX=9606 GN=FERMT2 PE=1 SV=1	77.8	6.7	FERMT2
P33121	Long-chain-fatty-acid--CoA ligase 1 OS=Homo sapiens OX=9606 GN=ACSL1 PE=1 SV=1	77.9	7.15	ACSL1
Q9NVA2	Septin-11 OS=Homo sapiens OX=9606 GN=SEPTIN11 PE=1 SV=3	49.4	6.81	SEPTIN11

Q07666	KH domain-containing, RNA-binding, signal transduction-associated protein 1 OS=Homo sapiens OX=9606 GN=KHDRBS1 PE=1 SV=1	48.2	8.66	KHDRBS1
O95573	Fatty acid CoA ligase Acsl3 OS=Homo sapiens OX=9606 GN=ACSL3 PE=1 SV=3	80.4	8.38	ACSL3
P62280	40S ribosomal protein S11 OS=Homo sapiens OX=9606 GN=RPS11 PE=1 SV=3	18.4	10.3	RPS11
P14923	Junction plakoglobin OS=Homo sapiens OX=9606 GN=JUP PE=1 SV=3	81.7	6.14	JUP
P54727	UV excision repair protein RAD23 homolog B OS=Homo sapiens OX=9606 GN=RAD23B PE=1 SV=1	43.1	4.84	RAD23B
P49720	Proteasome subunit beta type-3 OS=Homo sapiens OX=9606 GN=PSMB3 PE=1 SV=2	22.9	6.55	PSMB3
P36873	Serine/threonine-protein phosphatase PP1-gamma catalytic subunit OS=Homo sapiens OX=9606 GN=PPP1CC PE=1 SV=1	37	6.54	PPP1CC
Q9Y285	Phenylalanine--tRNA ligase alpha subunit OS=Homo sapiens OX=9606 GN=FARSA PE=1 SV=3	57.5	7.8	FARSA
P14550	Aldo-keto reductase family 1 member A1 OS=Homo sapiens OX=9606 GN=AKR1A1 PE=1 SV=3	36.6	6.79	AKR1A1
Q12904	Aminoacyl tRNA synthase complex-interacting multifunctional protein 1 OS=Homo sapiens OX=9606 GN=AIMP1 PE=1 SV=2	34.3	8.43	AIMP1
P21399	Cytoplasmic aconitate hydratase OS=Homo sapiens OX=9606 GN=ACO1 PE=1 SV=3	98.3	6.68	ACO1
Q92896	Golgi apparatus protein 1 OS=Homo sapiens OX=9606 GN=GLG1 PE=1 SV=2	134.5	6.9	GLG1
Q01995	Transgelin OS=Homo sapiens OX=9606 GN=TAGLN PE=1 SV=4	22.6	8.84	TAGLN
P08621	U1 small nuclear ribonucleoprotein 70 kDa OS=Homo sapiens OX=9606 GN=SNRNP70 PE=1 SV=2	51.5	9.94	SNRNP70
P28074	Proteasome subunit beta type-5 OS=Homo sapiens OX=9606 GN=PSMB5 PE=1 SV=3	28.5	6.92	PSMB5

O00303	Eukaryotic translation initiation factor 3 subunit F OS=Homo sapiens OX=9606 GN=EIF3F PE=1 SV=1	37.5	5.45	EIF3F
P62269	40S ribosomal protein S18 OS=Homo sapiens OX=9606 GN=RPS18 PE=1 SV=3	17.7	10.99	RPS18
O75347	Tubulin-specific chaperone A OS=Homo sapiens OX=9606 GN=TBCA PE=1 SV=3	12.8	5.29	TBCA
P46060	Ran GTPase-activating protein 1 OS=Homo sapiens OX=9606 GN=RANGAP1 PE=1 SV=1	63.5	4.68	RANGAP1
P20340	Ras-related protein Rab-6A OS=Homo sapiens OX=9606 GN=RAB6A PE=1 SV=3	23.6	5.54	RAB6A
O60313	Dynamin-like 120 kDa protein, mitochondrial OS=Homo sapiens OX=9606 GN=OPA1 PE=1 SV=3	111.6	7.87	OPA1
P10644	cAMP-dependent protein kinase type I-alpha regulatory subunit OS=Homo sapiens OX=9606 GN=PRKAR1A PE=1 SV=1	43	5.35	PRKAR1A
O14974	Protein phosphatase 1 regulatory subunit 12A OS=Homo sapiens OX=9606 GN=PPP1R12A PE=1 SV=1	115.2	5.4	PPP1R12A
P29692	Elongation factor 1-delta OS=Homo sapiens OX=9606 GN=EEF1D PE=1 SV=5	31.1	5.01	EEF1D
Q9UKK9	ADP-sugar pyrophosphatase OS=Homo sapiens OX=9606 GN=NUDT5 PE=1 SV=1	24.3	4.94	NUDT5
P61011	Signal recognition particle 54 kDa protein OS=Homo sapiens OX=9606 GN=SRP54 PE=1 SV=1	55.7	8.75	SRP54
Q15435	Protein phosphatase 1 regulatory subunit 7 OS=Homo sapiens OX=9606 GN=PPP1R7 PE=1 SV=1	41.5	4.91	PPP1R7
Q92734	Protein TFG OS=Homo sapiens OX=9606 GN=TFG PE=1 SV=2	43.4	5.1	TFG
P16401	Histone H1.5 OS=Homo sapiens OX=9606 GN=H1-5 PE=1 SV=3	22.6	10.92	H1-5
P43490	Nicotinamide phosphoribosyltransferase OS=Homo sapiens OX=9606 GN=NAMPT PE=1 SV=1	55.5	7.15	NAMPT
Q12797	Aspartyl/asparaginyl beta-hydroxylase OS=Homo sapiens OX=9606 GN=ASPH PE=1 SV=3	85.8	5.01	ASPH

Q9NUU7	ATP-dependent RNA helicase DDX19A OS=Homo sapiens OX=9606 GN=DDX19A PE=1 SV=1	53.9	6.58	DDX19A
Q8TCT9	Minor histocompatibility antigen H13 OS=Homo sapiens OX=9606 GN=HM13 PE=1 SV=1	41.5	6.43	HM13
Q15257	Serine/threonine-protein phosphatase 2A activator OS=Homo sapiens OX=9606 GN=PTPA PE=1 SV=3	40.6	5.94	PTPA
Q13765	Nascent polypeptide-associated complex subunit alpha OS=Homo sapiens OX=9606 GN=NACA PE=1 SV=1	23.4	4.56	NACA
Q16698	2,4-dienoyl-CoA reductase [(3E)-enoyl-CoA-producing], mitochondrial OS=Homo sapiens OX=9606 GN=DECR1 PE=1 SV=1	36	9.28	DECR1
P61160	Actin-related protein 2 OS=Homo sapiens OX=9606 GN=ACTR2 PE=1 SV=1	44.7	6.74	ACTR2
P15170	Eukaryotic peptide chain release factor GTP-binding subunit ERF3A OS=Homo sapiens OX=9606 GN=GSPT1 PE=1 SV=1	55.7	5.62	GSPT1
Q15155	Nodal modulator 1 OS=Homo sapiens OX=9606 GN=NOMO1 PE=1 SV=5	134.2	5.81	NOMO1
P26447	Protein S100-A4 OS=Homo sapiens OX=9606 GN=S100A4 PE=1 SV=1	11.7	6.11	S100A4
O96019	Actin-like protein 6A OS=Homo sapiens OX=9606 GN=ACTL6A PE=1 SV=1	47.4	5.6	ACTL6A
Q6NUK1	Calcium-binding mitochondrial carrier protein SCaMC-1 OS=Homo sapiens OX=9606 GN=SLC25A24 PE=1 SV=2	53.3	6.33	SLC25A24
Q96FQ6	Protein S100-A16 OS=Homo sapiens OX=9606 GN=S100A16 PE=1 SV=1	11.8	6.79	S100A16
Q9UMS4	Pre-mRNA-processing factor 19 OS=Homo sapiens OX=9606 GN=PRPF19 PE=1 SV=1	55.1	6.61	PRPF19
P30048	Thioredoxin-dependent peroxide reductase, mitochondrial OS=Homo sapiens OX=9606 GN=PRDX3 PE=1 SV=3	27.7	7.78	PRDX3
O95336	6-phosphogluconolactonase OS=Homo sapiens OX=9606 GN=PGLS PE=1 SV=2	27.5	6.05	PGLS

O00203	AP-3 complex subunit beta-1 OS=Homo sapiens OX=9606 GN=AP3B1 PE=1 SV=3	121.2	6.04	AP3B1
Q9H2G2	STE20-like serine/threonine-protein kinase OS=Homo sapiens OX=9606 GN=SLK PE=1 SV=1	142.6	5.15	SLK
Q7L2H7	Eukaryotic translation initiation factor 3 subunit M OS=Homo sapiens OX=9606 GN=EIF3M PE=1 SV=1	42.5	5.63	EIF3M
Q6YN16	Hydroxysteroid dehydrogenase-like protein 2 OS=Homo sapiens OX=9606 GN=HSDL2 PE=1 SV=1	45.4	7.99	HSDL2
P52895	Aldo-keto reductase family 1 member C2 OS=Homo sapiens OX=9606 GN=AKR1C2 PE=1 SV=3	36.7	7.49	AKR1C2
Q06124	Tyrosine-protein phosphatase non-receptor type 11 OS=Homo sapiens OX=9606 GN=PTPN11 PE=1 SV=3	68	7.3	PTPN11
Q9Y4W6	AFG3-like protein 2 OS=Homo sapiens OX=9606 GN=AFG3L2 PE=1 SV=2	88.5	8.66	AFG3L2
Q92688	Acidic leucine-rich nuclear phosphoprotein 32 family member B OS=Homo sapiens OX=9606 GN=ANP32B PE=1 SV=1	28.8	4.06	ANP32B
P06454	Prothymosin alpha OS=Homo sapiens OX=9606 GN=PTMA PE=1 SV=2	12.2	3.78	PTMA
P33993	DNA replication licensing factor MCM7 OS=Homo sapiens OX=9606 GN=MCM7 PE=1 SV=4	81.3	6.46	MCM7
Q9UJS0	Calcium-binding mitochondrial carrier protein Aralar2 OS=Homo sapiens OX=9606 GN=SLC25A13 PE=1 SV=2	74.1	8.62	SLC25A13
P27694	Replication protein A 70 kDa DNA-binding subunit OS=Homo sapiens OX=9606 GN=RPA1 PE=1 SV=2	68.1	7.21	RPA1
P39687	Acidic leucine-rich nuclear phosphoprotein 32 family member A OS=Homo sapiens OX=9606 GN=ANP32A PE=1 SV=1	28.6	4.09	ANP32A
Q8WVM8	Sec1 family domain-containing protein 1 OS=Homo sapiens OX=9606 GN=SCFD1 PE=1 SV=4	72.3	6.27	SCFD1
P38117	Electron transfer flavoprotein subunit beta OS=Homo sapiens OX=9606 GN=ETFB PE=1 SV=3	27.8	8.1	ETFB

P36551	Oxygen-dependent coproporphyrinogen-III oxidase, mitochondrial OS=Homo sapiens OX=9606 GN=CPOX PE=1 SV=3	50.1	8.25	CPOX
Q9BZQ8	Protein Niban 1 OS=Homo sapiens OX=9606 GN=NIBAN1 PE=1 SV=1	103.1	4.78	NIBAN1
P23368	NAD-dependent malic enzyme, mitochondrial OS=Homo sapiens OX=9606 GN=ME2 PE=1 SV=1	65.4	7.61	ME2
O60716	Catenin delta-1 OS=Homo sapiens OX=9606 GN=CTNND1 PE=1 SV=1	108.1	6.23	CTNND1
O15143	Actin-related protein 2/3 complex subunit 1B OS=Homo sapiens OX=9606 GN=ARPC1B PE=1 SV=3	40.9	8.35	ARPC1B
Q27J81	Inverted formin-2 OS=Homo sapiens OX=9606 GN=INF2 PE=1 SV=2	135.5	5.38	INF2
Q96HE7	ERO1-like protein alpha OS=Homo sapiens OX=9606 GN=ERO1A PE=1 SV=2	54.4	5.68	ERO1A
Q9UBE0	SUMO-activating enzyme subunit 1 OS=Homo sapiens OX=9606 GN=SAE1 PE=1 SV=1	38.4	5.3	SAE1
Q9NSD9	Phenylalanine--tRNA ligase beta subunit OS=Homo sapiens OX=9606 GN=FARSB PE=1 SV=3	66.1	6.84	FARSB
P0DP25	Calmodulin-3 OS=Homo sapiens OX=9606 GN=CALM3 PE=1 SV=1	16.8	4.22	CALM3
P99999	Cytochrome c OS=Homo sapiens OX=9606 GN=CYCS PE=1 SV=2	11.7	9.57	CYCS
P28288	ATP-binding cassette sub-family D member 3 OS=Homo sapiens OX=9606 GN=ABCD3 PE=1 SV=1	75.4	9.36	ABCD3
O43852	Calumenin OS=Homo sapiens OX=9606 GN=CALU PE=1 SV=2	37.1	4.64	CALU
P39748	Flap endonuclease 1 OS=Homo sapiens OX=9606 GN=FEN1 PE=1 SV=1	42.6	8.62	FEN1
Q9ULC4	Malignant T-cell-amplified sequence 1 OS=Homo sapiens OX=9606 GN=MCTS1 PE=1 SV=1	20.5	8.82	MCTS1
Q9UKM9	RNA-binding protein Raly OS=Homo sapiens OX=9606 GN=RALY PE=1 SV=1	32.4	9.17	RALY

Q16643	Drebrin OS=Homo sapiens OX=9606 GN=DBN1 PE=1 SV=4	71.4	4.45	DBN1
P50213	Isocitrate dehydrogenase [NAD] subunit alpha, mitochondrial OS=Homo sapiens OX=9606 GN=IDH3A PE=1 SV=1	39.6	6.92	IDH3A
P62249	40S ribosomal protein S16 OS=Homo sapiens OX=9606 GN=RPS16 PE=1 SV=2	16.4	10.21	RPS16
P13798	Acylamino-acid-releasing enzyme OS=Homo sapiens OX=9606 GN=APEH PE=1 SV=4	81.2	5.48	APEH
O15371	Eukaryotic translation initiation factor 3 subunit D OS=Homo sapiens OX=9606 GN=EIF3D PE=1 SV=1	63.9	6.05	EIF3D
Q9H910	Jupiter microtubule associated homolog 2 OS=Homo sapiens OX=9606 GN=JPT2 PE=1 SV=1	20.1	9.26	JPT2
Q07020	60S ribosomal protein L18 OS=Homo sapiens OX=9606 GN=RPL18 PE=1 SV=2	21.6	11.72	RPL18
P62879	Guanine nucleotide-binding protein G(I)/G(S)/G(T) subunit beta-2 OS=Homo sapiens OX=9606 GN=GNB2 PE=1 SV=3	37.3	6	GNB2
P31153	S-adenosylmethionine synthase isoform type-2 OS=Homo sapiens OX=9606 GN=MAT2A PE=1 SV=1	43.6	6.48	MAT2A
O43865	S-adenosylhomocysteine hydrolase-like protein 1 OS=Homo sapiens OX=9606 GN=AHCYL1 PE=1 SV=2	58.9	6.89	AHCYL1
Q15836	Vesicle-associated membrane protein 3 OS=Homo sapiens OX=9606 GN=VAMP3 PE=1 SV=3	11.3	8.79	VAMP3
P25788	Proteasome subunit alpha type-3 OS=Homo sapiens OX=9606 GN=PSMA3 PE=1 SV=2	28.4	5.33	PSMA3
Q99729	Heterogeneous nuclear ribonucleoprotein A/B OS=Homo sapiens OX=9606 GN=HNRNPAB PE=1 SV=2	36.2	8.21	HNRNPAB
P23921	Ribonucleoside-diphosphate reductase large subunit OS=Homo sapiens OX=9606 GN=RRM1 PE=1 SV=1	90	7.15	RRM1
P31942	Heterogeneous nuclear ribonucleoprotein H3 OS=Homo sapiens OX=9606 GN=HNRNPH3 PE=1 SV=2	36.9	6.87	HNRNPH3

P61088	Ubiquitin-conjugating enzyme E2 N OS=Homo sapiens OX=9606 GN=UBE2N PE=1 SV=1	17.1	6.57	UBE2N
P54920	Alpha-soluble NSF attachment protein OS=Homo sapiens OX=9606 GN=NAPA PE=1 SV=3	33.2	5.36	NAPA
P07099	Epoxide hydrolase 1 OS=Homo sapiens OX=9606 GN=EPHX1 PE=1 SV=1	52.9	7.25	EPHX1
O43747	AP-1 complex subunit gamma-1 OS=Homo sapiens OX=9606 GN=AP1G1 PE=1 SV=5	91.3	6.8	AP1G1
O00748	Cocaine esterase OS=Homo sapiens OX=9606 GN=CES2 PE=1 SV=1	61.8	6.1	CES2
O43175	D-3-phosphoglycerate dehydrogenase OS=Homo sapiens OX=9606 GN=PHGDH PE=1 SV=4	56.6	6.71	PHGDH
P07741	Adenine phosphoribosyltransferase OS=Homo sapiens OX=9606 GN=APRT PE=1 SV=2	19.6	6.02	APRT
Q9UBQ7	Glyoxylate reductase/hydroxypyruvate reductase OS=Homo sapiens OX=9606 GN=GRHPR PE=1 SV=1	35.6	7.39	GRHPR
Q9H2U2	Inorganic pyrophosphatase 2, mitochondrial OS=Homo sapiens OX=9606 GN=PPA2 PE=1 SV=2	37.9	7.39	PPA2
P09622	Dihydrolipoyl dehydrogenase, mitochondrial OS=Homo sapiens OX=9606 GN=DLD PE=1 SV=2	54.1	7.85	DLD
Q9NR45	Sialic acid synthase OS=Homo sapiens OX=9606 GN=NANS PE=1 SV=2	40.3	6.74	NANS
P62070	Ras-related protein R-Ras2 OS=Homo sapiens OX=9606 GN=RRAS2 PE=1 SV=1	23.4	6.01	RRAS2
P49903	Selenide, water dikinase 1 OS=Homo sapiens OX=9606 GN=SEPHS1 PE=1 SV=2	42.9	5.97	SEPHS1
P27144	Adenylate kinase 4, mitochondrial OS=Homo sapiens OX=9606 GN=AK4 PE=1 SV=1	25.3	8.4	AK4
P63172	Dynein light chain Tctex-type 1 OS=Homo sapiens OX=9606 GN=DYNLT1 PE=1 SV=1	12.4	5.08	DYNLT1
P18031	Tyrosine-protein phosphatase non-receptor type 1 OS=Homo sapiens OX=9606 GN=PTPN1 PE=1 SV=1	49.9	6.27	PTPN1

Q13418	Integrin-linked protein kinase OS=Homo sapiens OX=9606 GN=ILK PE=1 SV=2	51.4	8.07	ILK
P40429	60S ribosomal protein L13a OS=Homo sapiens OX=9606 GN=RPL13A PE=1 SV=2	23.6	10.93	RPL13A
Q92973	Transportin-1 OS=Homo sapiens OX=9606 GN=TNPO1 PE=1 SV=2	102.3	4.98	TNPO1
Q13867	Bleomycin hydrolase OS=Homo sapiens OX=9606 GN=BLMH PE=1 SV=1	52.5	6.27	BLMH
P39019	40S ribosomal protein S19 OS=Homo sapiens OX=9606 GN=RPS19 PE=1 SV=2	16.1	10.32	RPS19
O00499	Myc box-dependent-interacting protein 1 OS=Homo sapiens OX=9606 GN=BIN1 PE=1 SV=1	64.7	5.06	BIN1
Q99961	Endophilin-A2 OS=Homo sapiens OX=9606 GN=SH3GL1 PE=1 SV=1	41.5	5.43	SH3GL1
Q15833	Syntaxin-binding protein 2 OS=Homo sapiens OX=9606 GN=STXBP2 PE=1 SV=2	66.4	6.55	STXBP2
O60684	Importin subunit alpha-7 OS=Homo sapiens OX=9606 GN=KPNA6 PE=1 SV=1	60	4.98	KPNA6
P00568	Adenylate kinase isoenzyme 1 OS=Homo sapiens OX=9606 GN=AK1 PE=1 SV=3	21.6	8.63	AK1
O75396	Vesicle-trafficking protein SEC22b OS=Homo sapiens OX=9606 GN=SEC22B PE=1 SV=5	24.7	8.51	SEC22B
P12081	Histidine--tRNA ligase, cytoplasmic OS=Homo sapiens OX=9606 GN=HARS1 PE=1 SV=2	57.4	5.88	HARS1
O60884	DnaJ homolog subfamily A member 2 OS=Homo sapiens OX=9606 GN=DNAJA2 PE=1 SV=1	45.7	6.48	DNAJA2
P62244	40S ribosomal protein S15a OS=Homo sapiens OX=9606 GN=RPS15A PE=1 SV=2	14.8	10.13	RPS15A
Q9BZZ5	Apoptosis inhibitor 5 OS=Homo sapiens OX=9606 GN=API5 PE=1 SV=3	59	7.34	API5
Q9UBX3	Mitochondrial dicarboxylate carrier OS=Homo sapiens OX=9606 GN=SLC25A10 PE=1 SV=2	31.3	9.54	SLC25A10

Q00577	Transcriptional activator protein Pur-alpha OS=Homo sapiens OX=9606 GN=PURA PE=1 SV=2	34.9	6.44	PURA
Q9H444	Charged multivesicular body protein 4b OS=Homo sapiens OX=9606 GN=CHMP4B PE=1 SV=1	24.9	4.82	CHMP4B
Q7L266	Isoaspartyl peptidase/L-asparaginase OS=Homo sapiens OX=9606 GN=ASRGL1 PE=1 SV=2	32	6.24	ASRGL1
P55327	Tumor protein D52 OS=Homo sapiens OX=9606 GN=TPD52 PE=1 SV=2	24.3	4.83	TPD52
Q5TFE4	5'-nucleotidase domain-containing protein 1 OS=Homo sapiens OX=9606 GN=NT5DC1 PE=1 SV=1	51.8	6.35	NT5DC1
P31689	DnaJ homolog subfamily A member 1 OS=Homo sapiens OX=9606 GN=DNAJA1 PE=1 SV=2	44.8	7.08	DNAJA1
Q96HY6	DDRGK domain-containing protein 1 OS=Homo sapiens OX=9606 GN=DDRGK1 PE=1 SV=2	35.6	5.12	DDRGK1
Q06481	Amyloid beta precursor like protein 2 OS=Homo sapiens OX=9606 GN=APLP2 PE=1 SV=2	86.9	4.79	APLP2
Q92769	Histone deacetylase 2 OS=Homo sapiens OX=9606 GN=HDAC2 PE=1 SV=2	55.3	5.91	HDAC2
P47755	F-actin-capping protein subunit alpha-2 OS=Homo sapiens OX=9606 GN=CAPZA2 PE=1 SV=3	32.9	5.85	CAPZA2
P28066	Proteasome subunit alpha type-5 OS=Homo sapiens OX=9606 GN=PSMA5 PE=1 SV=3	26.4	4.79	PSMA5
Q9NR31	GTP-binding protein SAR1a OS=Homo sapiens OX=9606 GN=SAR1A PE=1 SV=1	22.4	6.68	SAR1A
Q9Y6G9	Cytoplasmic dynein 1 light intermediate chain 1 OS=Homo sapiens OX=9606 GN=DYNC1LI1 PE=1 SV=3	56.5	6.42	DYNC1LI1
P07602	Prosaposin OS=Homo sapiens OX=9606 GN=PSAP PE=1 SV=2	58.1	5.17	PSAP
P24534	Elongation factor 1-beta OS=Homo sapiens OX=9606 GN=EEF1B2 PE=1 SV=3	24.7	4.67	EEF1B2
Q9UJU6	Drebrin-like protein OS=Homo sapiens OX=9606 GN=DBNL PE=1 SV=1	48.2	5.05	DBNL

Q08257	Quinone oxidoreductase OS=Homo sapiens OX=9606 GN=CRYZ PE=1 SV=1	35.2	8.44	CRYZ
P46778	60S ribosomal protein L21 OS=Homo sapiens OX=9606 GN=RPL21 PE=1 SV=2	18.6	10.49	RPL21
O60493	Sorting nexin-3 OS=Homo sapiens OX=9606 GN=SNX3 PE=1 SV=3	18.8	8.66	SNX3
P13646	Keratin, type I cytoskeletal 13 OS=Homo sapiens OX=9606 GN=KRT13 PE=1 SV=4	49.6	4.96	KRT13
P42766	60S ribosomal protein L35 OS=Homo sapiens OX=9606 GN=RPL35 PE=1 SV=2	14.5	11.05	RPL35
Q12907	Vesicular integral-membrane protein VIP36 OS=Homo sapiens OX=9606 GN=LMAN2 PE=1 SV=1	40.2	6.95	LMAN2
Q9NTX5	Ethylmalonyl-CoA decarboxylase OS=Homo sapiens OX=9606 GN=ECHDC1 PE=1 SV=2	33.7	8.21	ECHDC1
Q53GQ0	Very-long-chain 3-oxoacyl-CoA reductase OS=Homo sapiens OX=9606 GN=HSD17B12 PE=1 SV=2	34.3	9.32	HSD17B12
P49257	Protein ERGIC-53 OS=Homo sapiens OX=9606 GN=LMAN1 PE=1 SV=2	57.5	6.77	LMAN1
O75489	NADH dehydrogenase [ubiquinone] iron-sulfur protein 3, mitochondrial OS=Homo sapiens OX=9606 GN=NDUFS3 PE=1 SV=1	30.2	7.5	NDUFS3
P10599	Thioredoxin OS=Homo sapiens OX=9606 GN=TXN PE=1 SV=3	11.7	4.92	TXN
Q9NTJ3	Structural maintenance of chromosomes protein 4 OS=Homo sapiens OX=9606 GN=SMC4 PE=1 SV=2	147.1	6.79	SMC4
P11172	Uridine 5'-monophosphate synthase OS=Homo sapiens OX=9606 GN=UMPS PE=1 SV=1	52.2	7.24	UMPS
P61289	Proteasome activator complex subunit 3 OS=Homo sapiens OX=9606 GN=PSME3 PE=1 SV=1	29.5	5.95	PSME3
P00533	Epidermal growth factor receptor OS=Homo sapiens OX=9606 GN=EGFR PE=1 SV=2	134.2	6.68	EGFR
P62917	60S ribosomal protein L8 OS=Homo sapiens OX=9606 GN=RPL8 PE=1 SV=2	28	11.03	RPL8

P60953	Cell division control protein 42 homolog OS=Homo sapiens OX=9606 GN=CDC42 PE=1 SV=2	21.2	6.55	CDC42
Q9UGV2	Protein NDRG3 OS=Homo sapiens OX=9606 GN=NDRG3 PE=1 SV=2	41.4	5.31	NDRG3
Q96I99	Succinate--CoA ligase [GDP-forming] subunit beta, mitochondrial OS=Homo sapiens OX=9606 GN=SUCLG2 PE=1 SV=2	46.5	6.39	SUCLG2
Q07021	Complement component 1 Q subcomponent-binding protein, mitochondrial OS=Homo sapiens OX=9606 GN=C1QBP PE=1 SV=1	31.3	4.84	C1QBP
Q9HCE1	Helicase MOV-10 OS=Homo sapiens OX=9606 GN=MOV10 PE=1 SV=2	113.6	8.82	MOV10
Q8WW12	PEST proteolytic signal-containing nuclear protein OS=Homo sapiens OX=9606 GN=PCNP PE=1 SV=2	18.9	7.49	PCNP
Q01844	RNA-binding protein EWS OS=Homo sapiens OX=9606 GN=EWSR1 PE=1 SV=1	68.4	9.33	EWSR1
P37108	Signal recognition particle 14 kDa protein OS=Homo sapiens OX=9606 GN=SRP14 PE=1 SV=2	14.6	10.04	SRP14
O15355	Protein phosphatase 1G OS=Homo sapiens OX=9606 GN=PPM1G PE=1 SV=1	59.2	4.36	PPM1G
Q99615	DnaJ homolog subfamily C member 7 OS=Homo sapiens OX=9606 GN=DNAJC7 PE=1 SV=2	56.4	6.96	DNAJC7
Q14254	Flotillin-2 OS=Homo sapiens OX=9606 GN=FLOT2 PE=1 SV=2	47	5.25	FLOT2
P16070	CD44 antigen OS=Homo sapiens OX=9606 GN=CD44 PE=1 SV=3	81.5	5.33	CD44
Q9Y6C9	Mitochondrial carrier homolog 2 OS=Homo sapiens OX=9606 GN=MTCH2 PE=1 SV=1	33.3	7.97	MTCH2
P62847	40S ribosomal protein S24 OS=Homo sapiens OX=9606 GN=RPS24 PE=1 SV=1	15.4	10.78	RPS24
Q9NYL9	Tropomodulin-3 OS=Homo sapiens OX=9606 GN=TMOD3 PE=1 SV=1	39.6	5.19	TMOD3

P23229	Integrin alpha-6 OS=Homo sapiens OX=9606 GN=ITGA6 PE=1 SV=5	126.5	6.61	ITGA6
Q13596	Sorting nexin-1 OS=Homo sapiens OX=9606 GN=SNX1 PE=1 SV=3	59	5.15	SNX1
P25205	DNA replication licensing factor MCM3 OS=Homo sapiens OX=9606 GN=MCM3 PE=1 SV=3	90.9	5.77	MCM3
Q09028	Histone-binding protein RBBP4 OS=Homo sapiens OX=9606 GN=RBBP4 PE=1 SV=3	47.6	4.89	RBBP4
Q92890	Ubiquitin recognition factor in ER-associated degradation protein 1 OS=Homo sapiens OX=9606 GN=UFD1 PE=1 SV=3	34.5	6.7	UFD1
O75964	ATP synthase subunit g, mitochondrial OS=Homo sapiens OX=9606 GN=ATP5MG PE=1 SV=3	11.4	9.64	ATP5MG
Q15418	Ribosomal protein S6 kinase alpha-1 OS=Homo sapiens OX=9606 GN=RPS6KA1 PE=1 SV=2	82.7	7.83	RPS6KA1
O96008	Mitochondrial import receptor subunit TOM40 homolog OS=Homo sapiens OX=9606 GN=TOMM40 PE=1 SV=1	37.9	7.25	TOMM40
Q9UN86	Ras GTPase-activating protein-binding protein 2 OS=Homo sapiens OX=9606 GN=G3BP2 PE=1 SV=2	54.1	5.55	G3BP2
P42126	Enoyl-CoA delta isomerase 1, mitochondrial OS=Homo sapiens OX=9606 GN=ECI1 PE=1 SV=1	32.8	8.54	ECI1
Q8NBJ5	Procollagen galactosyltransferase 1 OS=Homo sapiens OX=9606 GN=COLGALT1 PE=1 SV=1	71.6	7.31	COLGALT1
Q9BUJ2	Heterogeneous nuclear ribonucleoprotein U-like protein 1 OS=Homo sapiens OX=9606 GN=HNRNPUL1 PE=1 SV=2	95.7	6.92	HNRNPUL1
O94826	Mitochondrial import receptor subunit TOM70 OS=Homo sapiens OX=9606 GN=TOMM70 PE=1 SV=1	67.4	7.12	TOMM70
P13611	Versican core protein OS=Homo sapiens OX=9606 GN=VCAN PE=1 SV=3	372.6	4.51	VCAN
Q15717	ELAV-like protein 1 OS=Homo sapiens OX=9606 GN=ELAVL1 PE=1 SV=2	36.1	9.17	ELAVL1
P62888	60S ribosomal protein L30 OS=Homo sapiens OX=9606 GN=RPL30 PE=1 SV=2	12.8	9.63	RPL30

P35611	Alpha-adducin OS=Homo sapiens OX=9606 GN=ADD1 PE=1 SV=2	80.9	5.83	ADD1
O60869	Endothelial differentiation-related factor 1 OS=Homo sapiens OX=9606 GN=EDF1 PE=1 SV=1	16.4	9.95	EDF1
Q9UBF2	Coatomer subunit gamma-2 OS=Homo sapiens OX=9606 GN=COPG2 PE=1 SV=1	97.6	5.81	COPG2
P11217	Glycogen phosphorylase, muscle form OS=Homo sapiens OX=9606 GN=PYGM PE=1 SV=6	97	7.03	PYGM
P11717	Cation-independent mannose-6-phosphate receptor OS=Homo sapiens OX=9606 GN=IGF2R PE=1 SV=3	274.2	5.94	IGF2R
P62714	Serine/threonine-protein phosphatase 2A catalytic subunit beta isoform OS=Homo sapiens OX=9606 GN=PPP2CB PE=1 SV=1	35.6	5.43	PPP2CB
P53999	Activated RNA polymerase II transcriptional coactivator p15 OS=Homo sapiens OX=9606 GN=SUB1 PE=1 SV=3	14.4	9.6	SUB1
Q03135	Caveolin-1 OS=Homo sapiens OX=9606 GN=CAV1 PE=1 SV=4	20.5	6.02	CAV1
P84095	Rho-related GTP-binding protein RhoG OS=Homo sapiens OX=9606 GN=RHOG PE=1 SV=1	21.3	8.12	RHOG
Q9UQB8	Brain-specific angiogenesis inhibitor 1-associated protein 2 OS=Homo sapiens OX=9606 GN=BAIAP2 PE=1 SV=1	60.8	8.9	BAIAP2
A0MZ66	Shootin-1 OS=Homo sapiens OX=9606 GN=SHTN1 PE=1 SV=4	71.6	5.33	SHTN1
Q9BS26	Endoplasmic reticulum resident protein 44 OS=Homo sapiens OX=9606 GN=ERP44 PE=1 SV=1	46.9	5.26	ERP44
Q6FI81	Anamorsin OS=Homo sapiens OX=9606 GN=CIAPIN1 PE=1 SV=2	33.6	5.62	CIAPIN1
P61020	Ras-related protein Rab-5B OS=Homo sapiens OX=9606 GN=RAB5B PE=1 SV=1	23.7	8.13	RAB5B
Q9NP79	Vacuolar protein sorting-associated protein VTA1 homolog OS=Homo sapiens OX=9606 GN=VTA1 PE=1 SV=1	33.9	6.29	VTA1

P52788	Spermine synthase OS=Homo sapiens OX=9606 GN=SMS PE=1 SV=2	41.2	5.02	SMS
Q9NVI7	ATPase family AAA domain-containing protein 3A OS=Homo sapiens OX=9606 GN=ATAD3A PE=1 SV=2	71.3	8.98	ATAD3A
P17096	High mobility group protein HMG-I/HMG-Y OS=Homo sapiens OX=9606 GN=HMGA1 PE=1 SV=3	11.7	10.32	HMGA1
Q9BY44	Eukaryotic translation initiation factor 2A OS=Homo sapiens OX=9606 GN=EIF2A PE=1 SV=3	64.9	8.87	EIF2A
Q9BR76	Coronin-1B OS=Homo sapiens OX=9606 GN=CORO1B PE=1 SV=1	54.2	5.88	CORO1B
Q00688	Peptidyl-prolyl cis-trans isomerase FKBP3 OS=Homo sapiens OX=9606 GN=FKBP3 PE=1 SV=1	25.2	9.28	FKBP3
P30043	Flavin reductase (NADPH) OS=Homo sapiens OX=9606 GN=BLVRB PE=1 SV=3	22.1	7.65	BLVRB
P61970	Nuclear transport factor 2 OS=Homo sapiens OX=9606 GN=NUTF2 PE=1 SV=1	14.5	5.38	NUTF2
P21281	V-type proton ATPase subunit B, brain isoform OS=Homo sapiens OX=9606 GN=ATP6V1B2 PE=1 SV=3	56.5	5.81	ATP6V1B2
P48556	26S proteasome non-ATPase regulatory subunit 8 OS=Homo sapiens OX=9606 GN=PSMD8 PE=1 SV=2	39.6	9.7	PSMD8
P62987	Ubiquitin-60S ribosomal protein L40 OS=Homo sapiens OX=9606 GN=UBA52 PE=1 SV=2	14.7	9.83	UBA52
P62834	Ras-related protein Rap-1A OS=Homo sapiens OX=9606 GN=RAP1A PE=1 SV=1	21	6.67	RAP1A
Q32P28	Prolyl 3-hydroxylase 1 OS=Homo sapiens OX=9606 GN=P3H1 PE=1 SV=2	83.3	5.14	P3H1
Q9NQC3	Reticulon-4 OS=Homo sapiens OX=9606 GN=RTN4 PE=1 SV=2	129.9	4.5	RTN4
O75915	PRA1 family protein 3 OS=Homo sapiens OX=9606 GN=ARL6IP5 PE=1 SV=1	21.6	9.77	ARL6IP5
Q9BZK7	F-box-like/WD repeat-containing protein TBL1XR1 OS=Homo sapiens OX=9606 GN=TBL1XR1 PE=1 SV=1	55.6	5.55	TBL1XR1

P49189	4-trimethylaminobutyraldehyde dehydrogenase OS=Homo sapiens OX=9606 GN=ALDH9A1 PE=1 SV=3	53.8	5.87	ALDH9A1
O95394	Phosphoacetylglucosamine mutase OS=Homo sapiens OX=9606 GN=PGM3 PE=1 SV=1	59.8	6.25	PGM3
P13861	cAMP-dependent protein kinase type II-alpha regulatory subunit OS=Homo sapiens OX=9606 GN=PRKAR2A PE=1 SV=2	45.5	5.07	PRKAR2A
O94903	Pyridoxal phosphate homeostasis protein OS=Homo sapiens OX=9606 GN=PLPBP PE=1 SV=1	30.3	7.5	PLPBP
P60866	40S ribosomal protein S20 OS=Homo sapiens OX=9606 GN=RPS20 PE=1 SV=1	13.4	9.94	RPS20
Q14651	Plastin-1 OS=Homo sapiens OX=9606 GN=PLS1 PE=1 SV=2	70.2	5.41	PLS1
P09543	2',3'-cyclic-nucleotide 3'-phosphodiesterase OS=Homo sapiens OX=9606 GN=CNP PE=1 SV=2	47.5	9.07	CNP
Q9BXS5	AP-1 complex subunit mu-1 OS=Homo sapiens OX=9606 GN=AP1M1 PE=1 SV=3	48.6	7.3	AP1M1
P43487	Ran-specific GTPase-activating protein OS=Homo sapiens OX=9606 GN=RANBP1 PE=1 SV=1	23.3	5.29	RANBP1
Q04760	Lactoylglutathione lyase OS=Homo sapiens OX=9606 GN=GLO1 PE=1 SV=4	20.8	5.31	GLO1
Q9Y2Z0	Protein SGT1 homolog OS=Homo sapiens OX=9606 GN=SUGT1 PE=1 SV=3	41	5.16	SUGT1
P62829	60S ribosomal protein L23 OS=Homo sapiens OX=9606 GN=RPL23 PE=1 SV=1	14.9	10.51	RPL23
P62314	Small nuclear ribonucleoprotein Sm D1 OS=Homo sapiens OX=9606 GN=SNRPD1 PE=1 SV=1	13.3	11.56	SNRPD1
P36957	Dihydrolipoyllysine-residue succinyltransferase component of 2-oxoglutarate dehydrogenase complex, mitochondrial OS=Homo sapiens OX=9606 GN=DLST PE=1 SV=4	48.7	8.95	DLST
P20290	Transcription factor BTF3 OS=Homo sapiens OX=9606 GN=BTF3 PE=1 SV=1	22.2	9.38	BTF3

Q01650	Large neutral amino acids transporter small subunit 1 OS=Homo sapiens OX=9606 GN=SLC7A5 PE=1 SV=2	55	7.72	SLC7A5
Q29RF7	Sister chromatid cohesion protein PDS5 homolog A OS=Homo sapiens OX=9606 GN=PDS5A PE=1 SV=1	150.7	7.91	PDS5A
P50402	Emerin OS=Homo sapiens OX=9606 GN=EMD PE=1 SV=1	29	5.5	EMD
Q13057	Bifunctional coenzyme A synthase OS=Homo sapiens OX=9606 GN=COASY PE=1 SV=4	62.3	6.99	COASY
Q99816	Tumor susceptibility gene 101 protein OS=Homo sapiens OX=9606 GN=TSG101 PE=1 SV=2	43.9	6.46	TSG101
Q13724	Mannosyl-oligosaccharide glucosidase OS=Homo sapiens OX=9606 GN=MOGS PE=1 SV=5	91.9	8.9	MOGS
P62910	60S ribosomal protein L32 OS=Homo sapiens OX=9606 GN=RPL32 PE=1 SV=2	15.9	11.33	RPL32
P30566	Adenylosuccinate lyase OS=Homo sapiens OX=9606 GN=ADSL PE=1 SV=2	54.9	7.11	ADSL
O15372	Eukaryotic translation initiation factor 3 subunit H OS=Homo sapiens OX=9606 GN=EIF3H PE=1 SV=1	39.9	6.54	EIF3H
P55265	Double-stranded RNA-specific adenosine deaminase OS=Homo sapiens OX=9606 GN=ADAR PE=1 SV=4	136	8.65	ADAR
Q8WWM7	Ataxin-2-like protein OS=Homo sapiens OX=9606 GN=ATXN2L PE=1 SV=2	113.3	8.59	ATXN2L
Q9NRV9	Heme-binding protein 1 OS=Homo sapiens OX=9606 GN=HEBP1 PE=1 SV=1	21.1	5.8	HEBP1
P02794	Ferritin heavy chain OS=Homo sapiens OX=9606 GN=FTH1 PE=1 SV=2	21.2	5.55	FTH1
O95433	Activator of 90 kDa heat shock protein ATPase homolog 1 OS=Homo sapiens OX=9606 GN=AHSA1 PE=1 SV=1	38.3	5.53	AHSA1
P35268	60S ribosomal protein L22 OS=Homo sapiens OX=9606 GN=RPL22 PE=1 SV=2	14.8	9.19	RPL22
P55010	Eukaryotic translation initiation factor 5 OS=Homo sapiens OX=9606 GN=EIF5 PE=1 SV=2	49.2	5.58	EIF5

Q86YZ3	Hornerin OS=Homo sapiens OX=9606 GN=HRNR PE=1 SV=2	282.2	10.04	HRNR
Q9UMX0	Ubiquilin-1 OS=Homo sapiens OX=9606 GN=UBQLN1 PE=1 SV=2	62.5	5.11	UBQLN1
Q9NVD7	Alpha-parvin OS=Homo sapiens OX=9606 GN=PARVA PE=1 SV=1	42.2	5.95	PARVA
P22307	Sterol carrier protein 2 OS=Homo sapiens OX=9606 GN=SCP2 PE=1 SV=2	59	6.89	SCP2
Q04837	Single-stranded DNA-binding protein, mitochondrial OS=Homo sapiens OX=9606 GN=SSBP1 PE=1 SV=1	17.2	9.6	SSBP1
Q9BQE5	Apolipoprotein L2 OS=Homo sapiens OX=9606 GN=APOL2 PE=1 SV=1	37.1	6.74	APOL2
P84098	60S ribosomal protein L19 OS=Homo sapiens OX=9606 GN=RPL19 PE=1 SV=1	23.5	11.47	RPL19
P46063	ATP-dependent DNA helicase Q1 OS=Homo sapiens OX=9606 GN=RECQL PE=1 SV=3	73.4	7.88	RECQL
P61201	COP9 signalosome complex subunit 2 OS=Homo sapiens OX=9606 GN=COPS2 PE=1 SV=1	51.6	5.53	COPS2
Q7L1Q6	Basic leucine zipper and W2 domain-containing protein 1 OS=Homo sapiens OX=9606 GN=BZW1 PE=1 SV=1	48	5.92	BZW1
Q8NE71	ATP-binding cassette sub-family F member 1 OS=Homo sapiens OX=9606 GN=ABCF1 PE=1 SV=2	95.9	6.8	ABCF1
O15400	Syntaxin-7 OS=Homo sapiens OX=9606 GN=STX7 PE=1 SV=4	29.8	5.55	STX7
P62316	Small nuclear ribonucleoprotein Sm D2 OS=Homo sapiens OX=9606 GN=SNRPD2 PE=1 SV=1	13.5	9.91	SNRPD2
P54709	Sodium/potassium-transporting ATPase subunit beta-3 OS=Homo sapiens OX=9606 GN=ATP1B3 PE=1 SV=1	31.5	8.35	ATP1B3
Q9NX46	ADP-ribose glycohydrolase ARH3 OS=Homo sapiens OX=9606 GN=ADPRS PE=1 SV=1	38.9	5.07	ADPRS
Q8WUD1	Ras-related protein Rab-2B OS=Homo sapiens OX=9606 GN=RAB2B PE=1 SV=1	24.2	7.83	RAB2B

P63279	SUMO-conjugating enzyme UBC9 OS=Homo sapiens OX=9606 GN=UBE2I PE=1 SV=1	18	8.66	UBE2I
Q13185	Chromobox protein homolog 3 OS=Homo sapiens OX=9606 GN=CBX3 PE=1 SV=4	20.8	5.33	CBX3
Q9Y6N5	Sulfide:quinone oxidoreductase, mitochondrial OS=Homo sapiens OX=9606 GN=SQOR PE=1 SV=1	49.9	9.11	SQOR
O15145	Actin-related protein 2/3 complex subunit 3 OS=Homo sapiens OX=9606 GN=ARPC3 PE=1 SV=3	20.5	8.59	ARPC3
Q13501	Sequestosome-1 OS=Homo sapiens OX=9606 GN=SQSTM1 PE=1 SV=1	47.7	5.22	SQSTM1
Q9UD71	Protein phosphatase 1 regulatory subunit 1B OS=Homo sapiens OX=9606 GN=PPP1R1B PE=1 SV=2	22.9	4.53	PPP1R1B
Q9NQR4	Omega-amidase NIT2 OS=Homo sapiens OX=9606 GN=NIT2 PE=1 SV=1	30.6	7.21	NIT2
Q9UHD9	Ubiquilin-2 OS=Homo sapiens OX=9606 GN=UBQLN2 PE=1 SV=2	65.7	5.22	UBQLN2
P18754	Regulator of chromosome condensation OS=Homo sapiens OX=9606 GN=RCC1 PE=1 SV=1	44.9	7.52	RCC1
P00492	Hypoxanthine-guanine phosphoribosyltransferase OS=Homo sapiens OX=9606 GN=HPRT1 PE=1 SV=2	24.6	6.68	HPRT1
P82979	SAP domain-containing ribonucleoprotein OS=Homo sapiens OX=9606 GN=SARNP PE=1 SV=3	23.7	6.42	SARNP
Q13247	Serine/arginine-rich splicing factor 6 OS=Homo sapiens OX=9606 GN=SRSF6 PE=1 SV=2	39.6	11.43	SRSF6
Q9UBB4	Ataxin-10 OS=Homo sapiens OX=9606 GN=ATXN10 PE=1 SV=1	53.5	5.25	ATXN10
Q07866	Kinesin light chain 1 OS=Homo sapiens OX=9606 GN=KLC1 PE=1 SV=2	65.3	6.2	KLC1
P50552	Vasodilator-stimulated phosphoprotein OS=Homo sapiens OX=9606 GN=VASP PE=1 SV=3	39.8	8.94	VASP
P57721	Poly(rC)-binding protein 3 OS=Homo sapiens OX=9606 GN=PCBP3 PE=1 SV=2	39.4	8.07	PCBP3

P13693	Translationally-controlled tumor protein OS=Homo sapiens OX=9606 GN=TPT1 PE=1 SV=1	19.6	4.93	TPT1
O95810	Caveolae-associated protein 2 OS=Homo sapiens OX=9606 GN=CAVIN2 PE=1 SV=3	47.1	5.21	CAVIN2
P43034	Platelet-activating factor acetylhydrolase IB subunit beta OS=Homo sapiens OX=9606 GN=PAFAH1B1 PE=1 SV=2	46.6	7.37	PAFAH1B1
Q9HB07	MYG1 exonuclease OS=Homo sapiens OX=9606 GN=MYG1 PE=1 SV=3	42.5	6.77	MYG1
P15586	N-acetylglucosamine-6-sulfatase OS=Homo sapiens OX=9606 GN=GNS PE=1 SV=3	62	8.31	GNS
P19525	Interferon-induced, double-stranded RNA-activated protein kinase OS=Homo sapiens OX=9606 GN=EIF2AK2 PE=1 SV=2	62.1	8.4	EIF2AK2
P01889	HLA class I histocompatibility antigen, B alpha chain OS=Homo sapiens OX=9606 GN=HLA-B PE=1 SV=3	40.4	5.85	HLA-B
Q53EL6	Programmed cell death protein 4 OS=Homo sapiens OX=9606 GN=PDCD4 PE=1 SV=2	51.7	5.21	PDCD4
P61086	Ubiquitin-conjugating enzyme E2 K OS=Homo sapiens OX=9606 GN=UBE2K PE=1 SV=3	22.4	5.44	UBE2K
Q13547	Histone deacetylase 1 OS=Homo sapiens OX=9606 GN=HDAC1 PE=1 SV=1	55.1	5.48	HDAC1
Q96IU4	Protein ABHD14B OS=Homo sapiens OX=9606 GN=ABHD14B PE=1 SV=1	22.3	6.4	ABHD14B
P46783	40S ribosomal protein S10 OS=Homo sapiens OX=9606 GN=RPS10 PE=1 SV=1	18.9	10.15	RPS10
P61353	60S ribosomal protein L27 OS=Homo sapiens OX=9606 GN=RPL27 PE=1 SV=2	15.8	10.56	RPL27
Q9Y2W1	Thyroid hormone receptor-associated protein 3 OS=Homo sapiens OX=9606 GN=THRAP3 PE=1 SV=2	108.6	10.15	THRAP3
O00560	Syntenin-1 OS=Homo sapiens OX=9606 GN=SDCBP PE=1 SV=1	32.4	7.53	SDCBP
Q13564	NEDD8-activating enzyme E1 regulatory subunit OS=Homo sapiens OX=9606 GN=NAE1 PE=1 SV=1	60.2	5.4	NAE1

Q13126	S-methyl-5'-thioadenosine phosphorylase OS=Homo sapiens OX=9606 GN=MTAP PE=1 SV=2	31.2	7.18	MTAP
P10515	Dihydrolipoyllysine-residue acetyltransferase component of pyruvate dehydrogenase complex, mitochondrial OS=Homo sapiens OX=9606 GN=DLAT PE=1 SV=3	69	7.84	DLAT
Q96C19	EF-hand domain-containing protein D2 OS=Homo sapiens OX=9606 GN=EFHD2 PE=1 SV=1	26.7	5.2	EFHD2
P32004	Neural cell adhesion molecule L1 OS=Homo sapiens OX=9606 GN=L1CAM PE=1 SV=2	139.9	6.24	L1CAM
Q92522	Histone H1.10 OS=Homo sapiens OX=9606 GN=H1-10 PE=1 SV=1	22.5	10.76	H1-10
Q9BUT1	3-hydroxybutyrate dehydrogenase type 2 OS=Homo sapiens OX=9606 GN=BDH2 PE=1 SV=2	26.7	7.65	BDH2
O43488	Aflatoxin B1 aldehyde reductase member 2 OS=Homo sapiens OX=9606 GN=AKR7A2 PE=1 SV=3	39.6	7.17	AKR7A2
Q07812	Apoptosis regulator BAX OS=Homo sapiens OX=9606 GN=BAX PE=1 SV=1	21.2	5.22	BAX
Q9BTV4	Transmembrane protein 43 OS=Homo sapiens OX=9606 GN=TMEM43 PE=1 SV=1	44.8	8.13	TMEM43
Q15404	Ras suppressor protein 1 OS=Homo sapiens OX=9606 GN=RSU1 PE=1 SV=3	31.5	8.65	RSU1
P50416	Carnitine O-palmitoyltransferase 1, liver isoform OS=Homo sapiens OX=9606 GN=CPT1A PE=1 SV=2	88.3	8.65	CPT1A
Q14019	Coactosin-like protein OS=Homo sapiens OX=9606 GN=COTL1 PE=1 SV=3	15.9	5.67	COTL1
Q9UBT2	SUMO-activating enzyme subunit 2 OS=Homo sapiens OX=9606 GN=UBA2 PE=1 SV=2	71.2	5.29	UBA2
O75955	Flotillin-1 OS=Homo sapiens OX=9606 GN=FLOT1 PE=1 SV=3	47.3	7.49	FLOT1
Q9HDC9	Adipocyte plasma membrane-associated protein OS=Homo sapiens OX=9606 GN=APMAP PE=1 SV=2	46.5	6.16	APMAP

Q13618	Cullin-3 OS=Homo sapiens OX=9606 GN=CUL3 PE=1 SV=2	88.9	8.48	CUL3
P52888	Thimet oligopeptidase OS=Homo sapiens OX=9606 GN=THOP1 PE=1 SV=2	78.8	6.05	THOP1
Q9H845	Complex I assembly factor ACAD9, mitochondrial OS=Homo sapiens OX=9606 GN=ACAD9 PE=1 SV=1	68.7	7.96	ACAD9
Q9NQW7	Xaa-Pro aminopeptidase 1 OS=Homo sapiens OX=9606 GN=XPNPEP1 PE=1 SV=3	69.9	5.67	XPNPEP1
O00629	Importin subunit alpha-3 OS=Homo sapiens OX=9606 GN=KPNA4 PE=1 SV=1	57.9	4.96	KPNA4
O75340	Programmed cell death protein 6 OS=Homo sapiens OX=9606 GN=PDCD6 PE=1 SV=1	21.9	5.4	PDCD6
O43809	Cleavage and polyadenylation specificity factor subunit 5 OS=Homo sapiens OX=9606 GN=NUDT21 PE=1 SV=1	26.2	8.82	NUDT21
Q13404	Ubiquitin-conjugating enzyme E2 variant 1 OS=Homo sapiens OX=9606 GN=UBE2V1 PE=1 SV=2	16.5	7.93	UBE2V1
Q9ULA0	Aspartyl aminopeptidase OS=Homo sapiens OX=9606 GN=DNPEP PE=1 SV=2	53.4	7.58	DNPEP
P46977	Dolichyl-diphosphooligosaccharide--protein glycosyltransferase subunit STT3A OS=Homo sapiens OX=9606 GN=STT3A PE=1 SV=2	80.5	8.07	STT3A
Q9P2R7	Succinate--CoA ligase [ADP-forming] subunit beta, mitochondrial OS=Homo sapiens OX=9606 GN=SUCLA2 PE=1 SV=3	50.3	7.42	SUCLA2
Q9Y223	Bifunctional UDP-N-acetylglucosamine 2-epimerase/N-acetylmannosamine kinase OS=Homo sapiens OX=9606 GN=GNE PE=1 SV=1	79.2	6.8	GNE
P50914	60S ribosomal protein L14 OS=Homo sapiens OX=9606 GN=RPL14 PE=1 SV=4	23.4	10.93	RPL14
P10155	60 kDa SS-A/Ro ribonucleoprotein OS=Homo sapiens OX=9606 GN=RO60 PE=1 SV=2	60.6	8.03	RO60
Q9BUL8	Programmed cell death protein 10 OS=Homo sapiens OX=9606 GN=PDCD10 PE=1 SV=1	24.7	8.19	PDCD10

Q15056	Eukaryotic translation initiation factor 4H OS=Homo sapiens OX=9606 GN=EIF4H PE=1 SV=5	27.4	7.23	EIF4H
O75367	Core histone macro-H2A.1 OS=Homo sapiens OX=9606 GN=MACROH2A1 PE=1 SV=4	39.6	9.79	MACROH2A1
Q93052	Lipoma-preferred partner OS=Homo sapiens OX=9606 GN=LPP PE=1 SV=1	65.7	7.37	LPP
O95299	NADH dehydrogenase [ubiquinone] 1 alpha subcomplex subunit 10, mitochondrial OS=Homo sapiens OX=9606 GN=NDUFA10 PE=1 SV=1	40.7	8.48	NDUFA10
Q3ZCQ8	Mitochondrial import inner membrane translocase subunit TIM50 OS=Homo sapiens OX=9606 GN=TIMM50 PE=1 SV=2	39.6	8.37	TIMM50
Q9UHV9	Prefoldin subunit 2 OS=Homo sapiens OX=9606 GN=PFDN2 PE=1 SV=1	16.6	6.58	PFDN2
Q16629	Serine/arginine-rich splicing factor 7 OS=Homo sapiens OX=9606 GN=SRSF7 PE=1 SV=1	27.4	11.82	SRSF7
P35659	Protein DEK OS=Homo sapiens OX=9606 GN=DEK PE=1 SV=1	42.6	8.56	DEK
P49721	Proteasome subunit beta type-2 OS=Homo sapiens OX=9606 GN=PSMB2 PE=1 SV=1	22.8	7.02	PSMB2
Q7Z434	Mitochondrial antiviral-signaling protein OS=Homo sapiens OX=9606 GN=MAVS PE=1 SV=2	56.5	5.52	MAVS
Q9UNX3	60S ribosomal protein L26-like 1 OS=Homo sapiens OX=9606 GN=RPL26L1 PE=1 SV=1	17.2	10.55	RPL26L1
Q04323	UBX domain-containing protein 1 OS=Homo sapiens OX=9606 GN=UBXN1 PE=1 SV=2	33.3	5.25	UBXN1
P08559	Pyruvate dehydrogenase E1 component subunit alpha, somatic form, mitochondrial OS=Homo sapiens OX=9606 GN=PDHA1 PE=1 SV=3	43.3	8.06	PDHA1
P98179	RNA-binding protein 3 OS=Homo sapiens OX=9606 GN=RBM3 PE=1 SV=1	17.2	8.91	RBM3
P26232	Catenin alpha-2 OS=Homo sapiens OX=9606 GN=CTNNA2 PE=1 SV=5	105.2	5.71	CTNNA2

O14737	Programmed cell death protein 5 OS=Homo sapiens OX=9606 GN=PDCD5 PE=1 SV=3	14.3	6.04	PDCD5
Q16576	Histone-binding protein RBBP7 OS=Homo sapiens OX=9606 GN=RBBP7 PE=1 SV=1	47.8	5.05	RBBP7
Q15185	Prostaglandin E synthase 3 OS=Homo sapiens OX=9606 GN=PTGES3 PE=1 SV=1	18.7	4.54	PTGES3
Q16795	NADH dehydrogenase [ubiquinone] 1 alpha subcomplex subunit 9, mitochondrial OS=Homo sapiens OX=9606 GN=NDUFA9 PE=1 SV=2	42.5	9.8	NDUFA9
P53990	IST1 homolog OS=Homo sapiens OX=9606 GN=IST1 PE=1 SV=1	39.7	5.35	IST1
Q9UNH7	Sorting nexin-6 OS=Homo sapiens OX=9606 GN=SNX6 PE=1 SV=1	46.6	6.16	SNX6
Q9NP72	Ras-related protein Rab-18 OS=Homo sapiens OX=9606 GN=RAB18 PE=1 SV=1	23	5.24	RAB18
Q15369	Elongin-C OS=Homo sapiens OX=9606 GN=ELOC PE=1 SV=1	12.5	4.78	ELOC
Q05193	Dynamin-1 OS=Homo sapiens OX=9606 GN=DNM1 PE=1 SV=2	97.3	7.17	DNM1
P11279	Lysosome-associated membrane glycoprotein 1 OS=Homo sapiens OX=9606 GN=LAMP1 PE=1 SV=3	44.9	8.75	LAMP1
O14744	Protein arginine N-methyltransferase 5 OS=Homo sapiens OX=9606 GN=PRMT5 PE=1 SV=4	72.6	6.29	PRMT5
P25789	Proteasome subunit alpha type-4 OS=Homo sapiens OX=9606 GN=PSMA4 PE=1 SV=1	29.5	7.72	PSMA4
Q02978	Mitochondrial 2-oxoglutarate/malate carrier protein OS=Homo sapiens OX=9606 GN=SLC25A11 PE=1 SV=3	34	9.91	SLC25A11
Q8N1F7	Nuclear pore complex protein Nup93 OS=Homo sapiens OX=9606 GN=NUP93 PE=1 SV=2	93.4	5.72	NUP93
P36915	Guanine nucleotide-binding protein-like 1 OS=Homo sapiens OX=9606 GN=GNL1 PE=1 SV=2	68.6	5.8	GNL1
Q15819	Ubiquitin-conjugating enzyme E2 variant 2 OS=Homo sapiens OX=9606 GN=UBE2V2 PE=1 SV=4	16.4	8.09	UBE2V2

P63151	Serine/threonine-protein phosphatase 2A 55 kDa regulatory subunit B alpha isoform OS=Homo sapiens OX=9606 GN=PPP2R2A PE=1 SV=1	51.7	6.2	PPP2R2A
P46939	Utrophin OS=Homo sapiens OX=9606 GN=UTRN PE=1 SV=2	394.2	5.33	UTRN
Q13347	Eukaryotic translation initiation factor 3 subunit I OS=Homo sapiens OX=9606 GN=EIF3I PE=1 SV=1	36.5	5.64	EIF3I
O00505	Importin subunit alpha-4 OS=Homo sapiens OX=9606 GN=KPNA3 PE=1 SV=2	57.8	4.94	KPNA3
Q15942	Zyxin OS=Homo sapiens OX=9606 GN=ZYX PE=1 SV=1	61.2	6.67	ZYX
P51812	Ribosomal protein S6 kinase alpha-3 OS=Homo sapiens OX=9606 GN=RPS6KA3 PE=1 SV=1	83.7	6.89	RPS6KA3
O76021	Ribosomal L1 domain-containing protein 1 OS=Homo sapiens OX=9606 GN=RSL1D1 PE=1 SV=3	54.9	10.13	RSL1D1
P47985	Cytochrome b-c1 complex subunit Rieske, mitochondrial OS=Homo sapiens OX=9606 GN=UQCRFS1 PE=1 SV=2	29.6	8.32	UQCRFS1
Q9Y6E0	Serine/threonine-protein kinase 24 OS=Homo sapiens OX=9606 GN=STK24 PE=1 SV=1	49.3	5.69	STK24
Q01085	Nucleolysin TIAR OS=Homo sapiens OX=9606 GN=TIAL1 PE=1 SV=1	41.6	7.74	TIAL1
Q12874	Splicing factor 3A subunit 3 OS=Homo sapiens OX=9606 GN=SF3A3 PE=1 SV=1	58.8	5.38	SF3A3
P60033	CD81 antigen OS=Homo sapiens OX=9606 GN=CD81 PE=1 SV=1	25.8	5.29	CD81
Q5JTV8	Torsin-1A-interacting protein 1 OS=Homo sapiens OX=9606 GN=TOR1AIP1 PE=1 SV=2	66.2	8.18	TOR1AIP1
P62913	60S ribosomal protein L11 OS=Homo sapiens OX=9606 GN=RPL11 PE=1 SV=2	20.2	9.6	RPL11
P53004	Biliverdin reductase A OS=Homo sapiens OX=9606 GN=BLVRA PE=1 SV=2	33.4	6.44	BLVRA
P60891	Ribose-phosphate pyrophosphokinase 1 OS=Homo sapiens OX=9606 GN=PRPS1 PE=1 SV=2	34.8	6.98	PRPS1

P30520	Adenylosuccinate synthetase isozyme 2 OS=Homo sapiens OX=9606 GN=ADSS2 PE=1 SV=3	50.1	6.55	ADSS2
O00469	Procollagen-lysine,2-oxoglutarate 5-dioxygenase 2 OS=Homo sapiens OX=9606 GN=PLOD2 PE=1 SV=2	84.6	6.71	PLOD2
O95865	N(G),N(G)-dimethylarginine dimethylaminohydrolase 2 OS=Homo sapiens OX=9606 GN=DDAH2 PE=1 SV=1	29.6	6.01	DDAH2
Q02218	2-oxoglutarate dehydrogenase, mitochondrial OS=Homo sapiens OX=9606 GN=OGDH PE=1 SV=3	115.9	6.86	OGDH
P20339	Ras-related protein Rab-5A OS=Homo sapiens OX=9606 GN=RAB5A PE=1 SV=2	23.6	8.15	RAB5A
Q14451	Growth factor receptor-bound protein 7 OS=Homo sapiens OX=9606 GN=GRB7 PE=1 SV=2	59.6	8.5	GRB7
P02768	Albumin OS=Homo sapiens OX=9606 GN=ALB PE=1 SV=2	69.3	6.28	ALB
P52294	Importin subunit alpha-5 OS=Homo sapiens OX=9606 GN=KPNA1 PE=1 SV=3	60.2	5.01	KPNA1
Q16836	Hydroxyacyl-coenzyme A dehydrogenase, mitochondrial OS=Homo sapiens OX=9606 GN=HADH PE=1 SV=3	34.3	8.85	HADH
Q9UJ70	N-acetyl-D-glucosamine kinase OS=Homo sapiens OX=9606 GN=NAGK PE=1 SV=4	37.4	6.24	NAGK
Q15785	Mitochondrial import receptor subunit TOM34 OS=Homo sapiens OX=9606 GN=TOMM34 PE=1 SV=2	34.5	8.98	TOMM34
Q01081	Splicing factor U2AF 35 kDa subunit OS=Homo sapiens OX=9606 GN=U2AF1 PE=1 SV=3	27.9	8.81	U2AF1
P07996	Thrombospondin-1 OS=Homo sapiens OX=9606 GN=THBS1 PE=1 SV=2	129.3	4.94	THBS1
Q86XP3	ATP-dependent RNA helicase DDX42 OS=Homo sapiens OX=9606 GN=DDX42 PE=1 SV=1	102.9	7.02	DDX42
P69905	Hemoglobin subunit alpha OS=Homo sapiens OX=9606 GN=HBA1 PE=1 SV=2	15.2	8.68	HBA1; HBA2
O75534	Cold shock domain-containing protein E1 OS=Homo sapiens OX=9606 GN=CSDE1 PE=1 SV=2	88.8	6.25	CSDE1

Q96PK6	RNA-binding protein 14 OS=Homo sapiens OX=9606 GN=RBM14 PE=1 SV=2	69.4	9.67	RBM14
O75718	Cartilage-associated protein OS=Homo sapiens OX=9606 GN=CRTAP PE=1 SV=1	46.5	5.73	CRTAP
P16435	NADPH--cytochrome P450 reductase OS=Homo sapiens OX=9606 GN=POR PE=1 SV=2	76.6	5.58	POR
Q9BXP5	Serrate RNA effector molecule homolog OS=Homo sapiens OX=9606 GN=SRRT PE=1 SV=1	100.6	5.96	SRRT
A6NDG6	Glycerol-3-phosphate phosphatase OS=Homo sapiens OX=9606 GN=PGP PE=1 SV=1	34	6.14	PGP
P36405	ADP-ribosylation factor-like protein 3 OS=Homo sapiens OX=9606 GN=ARL3 PE=1 SV=2	20.4	7.24	ARL3
Q13151	Heterogeneous nuclear ribonucleoprotein A0 OS=Homo sapiens OX=9606 GN=HNRNPA0 PE=1 SV=1	30.8	9.29	HNRNPA0
Q99439	Calponin-2 OS=Homo sapiens OX=9606 GN=CNN2 PE=1 SV=4	33.7	7.33	CNN2
Q01581	Hydroxymethylglutaryl-CoA synthase, cytoplasmic OS=Homo sapiens OX=9606 GN=HMGCS1 PE=1 SV=2	57.3	5.41	HMGCS1
Q8NE86	Calcium uniporter protein, mitochondrial OS=Homo sapiens OX=9606 GN=MCU PE=1 SV=1	39.8	8.65	MCU
Q13492	Phosphatidylinositol-binding clathrin assembly protein OS=Homo sapiens OX=9606 GN=PICALM PE=1 SV=2	70.7	7.9	PICALM
P35270	Sepiapterin reductase OS=Homo sapiens OX=9606 GN=SPR PE=1 SV=1	28	8.05	SPR
Q9BY77	Polymerase delta-interacting protein 3 OS=Homo sapiens OX=9606 GN=POLDIP3 PE=1 SV=2	46.1	9.99	POLDIP3
P26373	60S ribosomal protein L13 OS=Homo sapiens OX=9606 GN=RPL13 PE=1 SV=4	24.2	11.65	RPL13
Q13155	Aminoacyl tRNA synthase complex-interacting multifunctional protein 2 OS=Homo sapiens OX=9606 GN=AIMP2 PE=1 SV=2	35.3	8.22	AIMP2
Q96CV9	Optineurin OS=Homo sapiens OX=9606 GN=OPTN PE=1 SV=3	65.9	5.17	OPTN

Q9NVJ2	ADP-ribosylation factor-like protein 8B OS=Homo sapiens OX=9606 GN=ARL8B PE=1 SV=1	21.5	8.43	ARL8B
Q96EP5	DAZ-associated protein 1 OS=Homo sapiens OX=9606 GN=DAZAP1 PE=1 SV=1	43.4	8.56	DAZAP1
Q08170	Serine/arginine-rich splicing factor 4 OS=Homo sapiens OX=9606 GN=SRSF4 PE=1 SV=2	56.6	11.52	SRSF4
P46776	60S ribosomal protein L27a OS=Homo sapiens OX=9606 GN=RPL27A PE=1 SV=2	16.6	11	RPL27A
Q99584	Protein S100-A13 OS=Homo sapiens OX=9606 GN=S100A13 PE=1 SV=1	11.5	6.16	S100A13
Q9Y383	Putative RNA-binding protein Luc7-like 2 OS=Homo sapiens OX=9606 GN=LUC7L2 PE=1 SV=2	46.5	10.01	LUC7L2
Q14498	RNA-binding protein 39 OS=Homo sapiens OX=9606 GN=RBM39 PE=1 SV=2	59.3	10.1	RBM39
Q6P179	Endoplasmic reticulum aminopeptidase 2 OS=Homo sapiens OX=9606 GN=ERAP2 PE=1 SV=2	110.4	6.71	ERAP2
Q9BZE1	39S ribosomal protein L37, mitochondrial OS=Homo sapiens OX=9606 GN=MRPL37 PE=1 SV=2	48.1	8.59	MRPL37
A0A0B4J2D5	Putative glutamine amidotransferase-like class 1 domain-containing protein 3B, mitochondrial OS=Homo sapiens OX=9606 GN=GATD3B PE=5 SV=1	28.1	8.27	GATD3B
P36507	Dual specificity mitogen-activated protein kinase kinase 2 OS=Homo sapiens OX=9606 GN=MAP2K2 PE=1 SV=1	44.4	6.55	MAP2K2
P00918	Carbonic anhydrase 2 OS=Homo sapiens OX=9606 GN=CA2 PE=1 SV=2	29.2	7.4	CA2
Q8IZP0	Abl interactor 1 OS=Homo sapiens OX=9606 GN=ABI1 PE=1 SV=4	55	7.06	ABI1
Q9NZL9	Methionine adenosyltransferase 2 subunit beta OS=Homo sapiens OX=9606 GN=MAT2B PE=1 SV=1	37.5	7.36	MAT2B
Q9Y6B6	GTP-binding protein SAR1b OS=Homo sapiens OX=9606 GN=SAR1B PE=1 SV=1	22.4	6.11	SAR1B
P41227	N-alpha-acetyltransferase 10 OS=Homo sapiens OX=9606 GN=NAA10 PE=1 SV=1	26.4	5.64	NAA10

P51648	Aldehyde dehydrogenase family 3 member A2 OS=Homo sapiens OX=9606 GN=ALDH3A2 PE=1 SV=1	54.8	7.88	ALDH3A2
Q14108	Lysosome membrane protein 2 OS=Homo sapiens OX=9606 GN=SCARB2 PE=1 SV=2	54.3	5.14	SCARB2
P61006	Ras-related protein Rab-8A OS=Homo sapiens OX=9606 GN=RAB8A PE=1 SV=1	23.7	9.07	RAB8A
Q71UI9	Histone H2A.V OS=Homo sapiens OX=9606 GN=H2AZ2 PE=1 SV=3	13.5	10.58	H2AZ2
O43681	ATPase GET3 OS=Homo sapiens OX=9606 GN=GET3 PE=1 SV=2	38.8	4.91	GET3
O95793	Double-stranded RNA-binding protein Staufen homolog 1 OS=Homo sapiens OX=9606 GN=STAU1 PE=1 SV=2	63.1	9.44	STAU1
Q05682	Caldesmon OS=Homo sapiens OX=9606 GN=CALD1 PE=1 SV=3	93.2	5.66	CALD1
P14735	Insulin-degrading enzyme OS=Homo sapiens OX=9606 GN=IDE PE=1 SV=4	117.9	6.61	IDE
P48163	NADP-dependent malic enzyme OS=Homo sapiens OX=9606 GN=ME1 PE=1 SV=1	64.1	6.13	ME1
O94992	Protein HEXIM1 OS=Homo sapiens OX=9606 GN=HEXIM1 PE=1 SV=1	40.6	4.89	HEXIM1
Q92599	Septin-8 OS=Homo sapiens OX=9606 GN=SEPTIN8 PE=1 SV=4	55.7	6.28	SEPTIN8
Q9Y2Q3	Glutathione S-transferase kappa 1 OS=Homo sapiens OX=9606 GN=GSTK1 PE=1 SV=3	25.5	8.41	GSTK1
Q9BYT8	Neurolysin, mitochondrial OS=Homo sapiens OX=9606 GN=NLN PE=1 SV=1	80.6	6.64	NLN
O75348	V-type proton ATPase subunit G 1 OS=Homo sapiens OX=9606 GN=ATP6V1G1 PE=1 SV=3	13.7	8.79	ATP6V1G1
P22087	rRNA 2'-O-methyltransferase fibrillarin OS=Homo sapiens OX=9606 GN=FBL PE=1 SV=2	33.8	10.18	FBL
P28062	Proteasome subunit beta type-8 OS=Homo sapiens OX=9606 GN=PSMB8 PE=1 SV=3	30.3	7.43	PSMB8

Q9Y2B0	Protein canopy homolog 2 OS=Homo sapiens OX=9606 GN=CNPY2 PE=1 SV=1	20.6	4.92	CNPY2
Q13557	Calcium/calmodulin-dependent protein kinase type II subunit delta OS=Homo sapiens OX=9606 GN=CAMK2D PE=1 SV=3	56.3	7.25	CAMK2D
P61163	Alpha-centractin OS=Homo sapiens OX=9606 GN=ACTR1A PE=1 SV=1	42.6	6.64	ACTR1A
O43252	Bifunctional 3'-phosphoadenosine 5'-phosphosulfate synthase 1 OS=Homo sapiens OX=9606 GN=PAPSS1 PE=1 SV=2	70.8	6.86	PAPSS1
Q9Y5K5	Ubiquitin carboxyl-terminal hydrolase isozyme L5 OS=Homo sapiens OX=9606 GN=UCHL5 PE=1 SV=3	37.6	5.33	UCHL5
Q9NXG2	THUMP domain-containing protein 1 OS=Homo sapiens OX=9606 GN=THUMPD1 PE=1 SV=2	39.3	7.88	THUMPD1
O75822	Eukaryotic translation initiation factor 3 subunit J OS=Homo sapiens OX=9606 GN=EIF3J PE=1 SV=2	29	4.83	EIF3J
Q13242	Serine/arginine-rich splicing factor 9 OS=Homo sapiens OX=9606 GN=SRSF9 PE=1 SV=1	25.5	8.65	SRSF9
P41567	Eukaryotic translation initiation factor 1 OS=Homo sapiens OX=9606 GN=EIF1 PE=1 SV=1	12.7	7.44	EIF1
P53007	Tricarboxylate transport protein, mitochondrial OS=Homo sapiens OX=9606 GN=SLC25A1 PE=1 SV=2	34	9.89	SLC25A1
Q14683	Structural maintenance of chromosomes protein 1A OS=Homo sapiens OX=9606 GN=SMC1A PE=1 SV=2	143.1	7.64	SMC1A
O00273	DNA fragmentation factor subunit alpha OS=Homo sapiens OX=9606 GN=DFFA PE=1 SV=1	36.5	4.79	DFFA
P35080	Profilin-2 OS=Homo sapiens OX=9606 GN=PFN2 PE=1 SV=3	15	6.99	PFN2
P23634	Plasma membrane calcium-transporting ATPase 4 OS=Homo sapiens OX=9606 GN=ATP2B4 PE=1 SV=2	137.8	6.6	ATP2B4
P21912	Succinate dehydrogenase [ubiquinone] iron-sulfur subunit, mitochondrial OS=Homo sapiens OX=9606 GN=SDHB PE=1 SV=3	31.6	8.76	SDHB

P49792	E3 SUMO-protein ligase RanBP2 OS=Homo sapiens OX=9606 GN=RANBP2 PE=1 SV=2	358	6.2	RANBP2
Q01130	Serine/arginine-rich splicing factor 2 OS=Homo sapiens OX=9606 GN=SRSF2 PE=1 SV=4	25.5	11.85	SRSF2
P60903	Protein S100-A10 OS=Homo sapiens OX=9606 GN=S100A10 PE=1 SV=2	11.2	7.37	S100A10
P49821	NADH dehydrogenase [ubiquinone] flavoprotein 1, mitochondrial OS=Homo sapiens OX=9606 GN=NDUFV1 PE=1 SV=4	50.8	8.21	NDUFV1
P51571	Translocon-associated protein subunit delta OS=Homo sapiens OX=9606 GN=SSR4 PE=1 SV=1	19	6.15	SSR4
P09012	U1 small nuclear ribonucleoprotein A OS=Homo sapiens OX=9606 GN=SNRPA PE=1 SV=3	31.3	9.83	SNRPA
Q9H9B4	Sideroflexin-1 OS=Homo sapiens OX=9606 GN=SFXN1 PE=1 SV=4	35.6	9.07	SFXN1
Q15437	Protein transport protein Sec23B OS=Homo sapiens OX=9606 GN=SEC23B PE=1 SV=2	86.4	6.89	SEC23B
O75569	Interferon-inducible double-stranded RNA-dependent protein kinase activator A OS=Homo sapiens OX=9606 GN=PRKRA PE=1 SV=1	34.4	8.41	PRKRA
Q9UKY7	Protein CDV3 homolog OS=Homo sapiens OX=9606 GN=CDV3 PE=1 SV=1	27.3	6.4	CDV3
Q9UGI8	Testin OS=Homo sapiens OX=9606 GN=TES PE=1 SV=1	48	7.68	TES
Q8TBC4	NEDD8-activating enzyme E1 catalytic subunit OS=Homo sapiens OX=9606 GN=UBA3 PE=1 SV=2	51.8	5.45	UBA3
O95292	Vesicle-associated membrane protein-associated protein B/C OS=Homo sapiens OX=9606 GN=VAPB PE=1 SV=3	27.2	7.3	VAPB
Q9BXJ9	N-alpha-acetyltransferase 15, NatA auxiliary subunit OS=Homo sapiens OX=9606 GN=NAA15 PE=1 SV=1	101.2	7.42	NAA15
Q13442	28 kDa heat- and acid-stable phosphoprotein OS=Homo sapiens OX=9606 GN=PDAP1 PE=1 SV=1	20.6	8.87	PDAP1

P29992	Guanine nucleotide-binding protein subunit alpha-11 OS=Homo sapiens OX=9606 GN=GNA11 PE=1 SV=2	42.1	5.69	GNA11
P49736	DNA replication licensing factor MCM2 OS=Homo sapiens OX=9606 GN=MCM2 PE=1 SV=4	101.8	5.52	MCM2
P55036	26S proteasome non-ATPase regulatory subunit 4 OS=Homo sapiens OX=9606 GN=PSMD4 PE=1 SV=1	40.7	4.79	PSMD4
P28072	Proteasome subunit beta type-6 OS=Homo sapiens OX=9606 GN=PSMB6 PE=1 SV=4	25.3	4.92	PSMB6
Q63ZY3	KN motif and ankyrin repeat domain-containing protein 2 OS=Homo sapiens OX=9606 GN=KANK2 PE=1 SV=1	91.1	5.63	KANK2
Q5JWF2	Guanine nucleotide-binding protein G(s) subunit alpha isoforms XLas OS=Homo sapiens OX=9606 GN=GNAS PE=1 SV=2	111	5.03	GNAS
P63000	Ras-related C3 botulinum toxin substrate 1 OS=Homo sapiens OX=9606 GN=RAC1 PE=1 SV=1	21.4	8.5	RAC1
O60610	Protein diaphanous homolog 1 OS=Homo sapiens OX=9606 GN=DIAPH1 PE=1 SV=2	141.3	5.41	DIAPH1
Q02818	Nucleobindin-1 OS=Homo sapiens OX=9606 GN=NUCB1 PE=1 SV=4	53.8	5.25	NUCB1
Q52LJ0	Protein FAM98B OS=Homo sapiens OX=9606 GN=FAM98B PE=1 SV=2	45.5	8.69	FAM98B
P35269	General transcription factor IIF subunit 1 OS=Homo sapiens OX=9606 GN=GTF2F1 PE=1 SV=2	58.2	7.49	GTF2F1
P61923	Coatomer subunit zeta-1 OS=Homo sapiens OX=9606 GN=COPZ1 PE=1 SV=1	20.2	4.81	COPZ1
Q9P0K7	Ankycorbin OS=Homo sapiens OX=9606 GN=RAI14 PE=1 SV=2	110	6.21	RAI14
P00403	Cytochrome c oxidase subunit 2 OS=Homo sapiens OX=9606 GN=MT-CO2 PE=1 SV=1	25.5	4.82	MT-CO2
P11387	DNA topoisomerase 1 OS=Homo sapiens OX=9606 GN=TOP1 PE=1 SV=2	90.7	9.31	TOP1
Q14318	Peptidyl-prolyl cis-trans isomerase FKBP8 OS=Homo sapiens OX=9606 GN=FKBP8 PE=1 SV=2	44.5	4.84	FKBP8

P05067	Amyloid-beta precursor protein OS=Homo sapiens OX=9606 GN=APP PE=1 SV=3	86.9	4.82	APP
Q9UL25	Ras-related protein Rab-21 OS=Homo sapiens OX=9606 GN=RAB21 PE=1 SV=3	24.3	7.94	RAB21
O15258	Protein RER1 OS=Homo sapiens OX=9606 GN=RER1 PE=1 SV=1	22.9	9.54	RER1
Q8WWY3	U4/U6 small nuclear ribonucleoprotein Prp31 OS=Homo sapiens OX=9606 GN=PRPF31 PE=1 SV=2	55.4	5.78	PRPF31
Q9Y5Z4	Heme-binding protein 2 OS=Homo sapiens OX=9606 GN=HEBP2 PE=1 SV=1	22.9	4.63	HEBP2
P08240	Signal recognition particle receptor subunit alpha OS=Homo sapiens OX=9606 GN=SRPRA PE=1 SV=2	69.8	8.95	SRPRA
P53367	Arfaptin-1 OS=Homo sapiens OX=9606 GN=ARFIP1 PE=1 SV=2	41.7	6.7	ARFIP1
Q9UKK3	Protein mono-ADP-ribosyltransferase PARP4 OS=Homo sapiens OX=9606 GN=PARP4 PE=1 SV=3	192.5	5.66	PARP4
P11234	Ras-related protein Ral-B OS=Homo sapiens OX=9606 GN=RALB PE=1 SV=1	23.4	6.62	RALB
Q96G03	Phosphoglucomutase-2 OS=Homo sapiens OX=9606 GN=PGM2 PE=1 SV=4	68.2	6.73	PGM2
P14324	Farnesyl pyrophosphate synthase OS=Homo sapiens OX=9606 GN=FDPS PE=1 SV=4	48.2	6.15	FDPS
P39060	Collagen alpha-1(XVIII) chain OS=Homo sapiens OX=9606 GN=COL18A1 PE=1 SV=5	178.1	6.01	COL18A1
P17174	Aspartate aminotransferase, cytoplasmic OS=Homo sapiens OX=9606 GN=GOT1 PE=1 SV=3	46.2	7.01	GOT1
Q9H307	Pinin OS=Homo sapiens OX=9606 GN=PNN PE=1 SV=5	81.6	7.14	PNN
P19013	Keratin, type II cytoskeletal 4 OS=Homo sapiens OX=9606 GN=KRT4 PE=1 SV=5	56.1	6.61	KRT4
O15397	Importin-8 OS=Homo sapiens OX=9606 GN=IPO8 PE=1 SV=2	119.9	5.16	IPO8

Q96A26	Protein FAM162A OS=Homo sapiens OX=9606 GN=FAM162A PE=1 SV=2	17.3	9.77	FAM162A
Q9HC38	Glyoxalase domain-containing protein 4 OS=Homo sapiens OX=9606 GN=GLOD4 PE=1 SV=1	34.8	5.6	GLOD4
P07203	Glutathione peroxidase 1 OS=Homo sapiens OX=9606 GN=GPX1 PE=1 SV=4	22.1	6.55	GPX1
P51570	Galactokinase OS=Homo sapiens OX=9606 GN=GALK1 PE=1 SV=1	42.2	6.46	GALK1
Q9Y224	RNA transcription, translation and transport factor protein OS=Homo sapiens OX=9606 GN=RTRAF PE=1 SV=1	28.1	6.65	RTRAF
Q7L5N1	COP9 signalosome complex subunit 6 OS=Homo sapiens OX=9606 GN=COPS6 PE=1 SV=1	36.1	5.73	COPS6
Q13423	NAD(P) transhydrogenase, mitochondrial OS=Homo sapiens OX=9606 GN=NNT PE=1 SV=3	113.8	8.09	NNT
P29590	Protein PML OS=Homo sapiens OX=9606 GN=PML PE=1 SV=3	97.5	6.21	PML
Q9BRP8	Partner of Y14 and mago OS=Homo sapiens OX=9606 GN=PYM1 PE=1 SV=1	22.6	9.45	PYM1
O14908	PDZ domain-containing protein GIPC1 OS=Homo sapiens OX=9606 GN=GIPC1 PE=1 SV=2	36	6.28	GIPC1
Q6IBS0	Twinfilin-2 OS=Homo sapiens OX=9606 GN=TWF2 PE=1 SV=2	39.5	6.84	TWF2
Q15363	Transmembrane emp24 domain-containing protein 2 OS=Homo sapiens OX=9606 GN=TMED2 PE=1 SV=1	22.7	5.17	TMED2
Q9NP97	Dynein light chain roadblock-type 1 OS=Homo sapiens OX=9606 GN=DYNLRB1 PE=1 SV=3	10.9	7.25	DYNLRB1
P49756	RNA-binding protein 25 OS=Homo sapiens OX=9606 GN=RBM25 PE=1 SV=3	100.1	6.32	RBM25
Q96A33	PAT complex subunit CCDC47 OS=Homo sapiens OX=9606 GN=CCDC47 PE=1 SV=1	55.8	4.87	CCDC47
Q9H3U1	Protein unc-45 homolog A OS=Homo sapiens OX=9606 GN=UNC45A PE=1 SV=1	103	6.07	UNC45A

Q02750	Dual specificity mitogen-activated protein kinase kinase 1 OS=Homo sapiens OX=9606 GN=MAP2K1 PE=1 SV=2	43.4	6.62	MAP2K1
Q8NCW5	NAD(P)H-hydrate epimerase OS=Homo sapiens OX=9606 GN=NAXE PE=1 SV=2	31.7	7.66	NAXE
Q14566	DNA replication licensing factor MCM6 OS=Homo sapiens OX=9606 GN=MCM6 PE=1 SV=1	92.8	5.41	MCM6
Q15020	Squamous cell carcinoma antigen recognized by T-cells 3 OS=Homo sapiens OX=9606 GN=SART3 PE=1 SV=1	109.9	5.57	SART3
P36969	Phospholipid hydroperoxide glutathione peroxidase OS=Homo sapiens OX=9606 GN=GPX4 PE=1 SV=3	22.2	8.37	GPX4
P24666	Low molecular weight phosphotyrosine protein phosphatase OS=Homo sapiens OX=9606 GN=ACP1 PE=1 SV=3	18	6.74	ACP1
Q15599	Na(+)/H(+) exchange regulatory cofactor NHE-RF2 OS=Homo sapiens OX=9606 GN=SLC9A3R2 PE=1 SV=2	37.4	7.93	SLC9A3R2
Q9BZE9	Tether containing UBX domain for GLUT4 OS=Homo sapiens OX=9606 GN=ASPSCR1 PE=1 SV=1	60.1	6.64	ASPSCR1
O14964	Hepatocyte growth factor-regulated tyrosine kinase substrate OS=Homo sapiens OX=9606 GN=HGS PE=1 SV=1	86.1	6.16	HGS
Q9BWF3	RNA-binding protein 4 OS=Homo sapiens OX=9606 GN=RBM4 PE=1 SV=1	40.3	7.08	RBM4
Q9NYF8	Bcl-2-associated transcription factor 1 OS=Homo sapiens OX=9606 GN=BCLAF1 PE=1 SV=2	106.1	9.98	BCLAF1
P40616	ADP-ribosylation factor-like protein 1 OS=Homo sapiens OX=9606 GN=ARL1 PE=1 SV=1	20.4	5.72	ARL1
O60547	GDP-mannose 4,6 dehydratase OS=Homo sapiens OX=9606 GN=GMDS PE=1 SV=1	41.9	7.31	GMDS
P30837	Aldehyde dehydrogenase X, mitochondrial OS=Homo sapiens OX=9606 GN=ALDH1B1 PE=1 SV=3	57.2	6.8	
P40222	Alpha-taxilin OS=Homo sapiens OX=9606 GN=TXLNA PE=1 SV=3	61.9	6.52	TXLNA

Q9UIA9	Exportin-7 OS=Homo sapiens OX=9606 GN=XPO7 PE=1 SV=3	123.8	6.32	XPO7
O95716	Ras-related protein Rab-3D OS=Homo sapiens OX=9606 GN=RAB3D PE=1 SV=1	24.3	4.93	RAB3D
P42677	40S ribosomal protein S27 OS=Homo sapiens OX=9606 GN=RPS27 PE=1 SV=3	9.5	9.45	RPS27
P55809	Succinyl-CoA:3-ketoacid coenzyme A transferase 1, mitochondrial OS=Homo sapiens OX=9606 GN=OXCT1 PE=1 SV=1	56.1	7.46	OXCT1
Q9NZZ3	Charged multivesicular body protein 5 OS=Homo sapiens OX=9606 GN=CHMP5 PE=1 SV=1	24.6	4.83	CHMP5
Q969H8	Myeloid-derived growth factor OS=Homo sapiens OX=9606 GN=MYDGF PE=1 SV=1	18.8	6.68	MYDGF
P09417	Dihydropteridine reductase OS=Homo sapiens OX=9606 GN=QDPR PE=1 SV=2	25.8	7.37	QDPR
P40306	Proteasome subunit beta type-10 OS=Homo sapiens OX=9606 GN=PSMB10 PE=1 SV=1	28.9	7.81	PSMB10
Q86TI2	Dipeptidyl peptidase 9 OS=Homo sapiens OX=9606 GN=DPP9 PE=1 SV=3	98.2	6.46	DPP9
Q9Y3B7	39S ribosomal protein L11, mitochondrial OS=Homo sapiens OX=9606 GN=MRPL11 PE=1 SV=1	20.7	9.91	MRPL11
P23919	Thymidylate kinase OS=Homo sapiens OX=9606 GN=DTYMK PE=1 SV=4	23.8	8.27	DTYMK
P32929	Cystathionine gamma-lyase OS=Homo sapiens OX=9606 GN=CTH PE=1 SV=3	44.5	6.7	CTH
Q9H993	Damage-control phosphatase ARMT1 OS=Homo sapiens OX=9606 GN=ARMT1 PE=1 SV=1	51.1	5.76	ARMT1
O75351	Vacuolar protein sorting-associated protein 4B OS=Homo sapiens OX=9606 GN=VPS4B PE=1 SV=2	49.3	7.23	VPS4B
Q9Y5B9	FACT complex subunit SPT16 OS=Homo sapiens OX=9606 GN=SUPT16H PE=1 SV=1	119.8	5.66	SUPT16H
O15427	Monocarboxylate transporter 4 OS=Homo sapiens OX=9606 GN=SLC16A3 PE=1 SV=1	49.4	7.96	SLC16A3

Q6XQN6	Nicotinate phosphoribosyltransferase OS=Homo sapiens OX=9606 GN=NAPRT PE=1 SV=2	57.5	5.68	NAPRT
P46926	Glucosamine-6-phosphate isomerase 1 OS=Homo sapiens OX=9606 GN=GNPDA1 PE=1 SV=1	32.6	6.92	GNPDA1
Q92820	Gamma-glutamyl hydrolase OS=Homo sapiens OX=9606 GN=GGH PE=1 SV=2	35.9	7.11	GGH
P52292	Importin subunit alpha-1 OS=Homo sapiens OX=9606 GN=KPNA2 PE=1 SV=1	57.8	5.4	KPNA2
O14880	Microsomal glutathione S-transferase 3 OS=Homo sapiens OX=9606 GN=MGST3 PE=1 SV=1	16.5	9.38	MGST3
Q92882	Osteoclast-stimulating factor 1 OS=Homo sapiens OX=9606 GN=OSTF1 PE=1 SV=2	23.8	5.68	OSTF1
Q15758	Neutral amino acid transporter B(0) OS=Homo sapiens OX=9606 GN=SLC1A5 PE=1 SV=2	56.6	5.48	SLC1A5
P55196	Afadin OS=Homo sapiens OX=9606 GN=AFDN PE=1 SV=3	206.7	6.47	AFDN
Q13630	GDP-L-fucose synthase OS=Homo sapiens OX=9606 GN=GFUS PE=1 SV=1	35.9	6.6	GFUS
Q13510	Acid ceramidase OS=Homo sapiens OX=9606 GN=ASAH1 PE=1 SV=5	44.6	7.62	ASAH1
O95881	Thioredoxin domain-containing protein 12 OS=Homo sapiens OX=9606 GN=TXNDC12 PE=1 SV=1	19.2	5.4	TXNDC12
P35249	Replication factor C subunit 4 OS=Homo sapiens OX=9606 GN=RFC4 PE=1 SV=2	39.7	8.02	RFC4
Q99685	Monoglyceride lipase OS=Homo sapiens OX=9606 GN=MGLL PE=1 SV=2	33.2	6.99	MGLL
Q08209	Serine/threonine-protein phosphatase 2B catalytic subunit alpha isoform OS=Homo sapiens OX=9606 GN=PPP3CA PE=1 SV=1	58.7	5.86	PPP3CA
P55145	Mesencephalic astrocyte-derived neurotrophic factor OS=Homo sapiens OX=9606 GN=MANF PE=1 SV=3	20.7	8.69	MANF
Q9Y3U8	60S ribosomal protein L36 OS=Homo sapiens OX=9606 GN=RPL36 PE=1 SV=3	12.2	11.59	RPL36

Q9NT62	Ubiquitin-like-conjugating enzyme ATG3 OS=Homo sapiens OX=9606 GN=ATG3 PE=1 SV=1	35.8	4.74	ATG3
Q8TAQ2	SWI/SNF complex subunit SMARCC2 OS=Homo sapiens OX=9606 GN=SMARCC2 PE=1 SV=1	132.8	5.69	SMARCC2
Q96CP2	FLYWCH family member 2 OS=Homo sapiens OX=9606 GN=FLYWCH2 PE=1 SV=1	14.6	8.46	FLYWCH2
O00567	Nucleolar protein 56 OS=Homo sapiens OX=9606 GN=NOP56 PE=1 SV=4	66	9.19	NOP56
Q13526	Peptidyl-prolyl cis-trans isomerase NIMA-interacting 1 OS=Homo sapiens OX=9606 GN=PIN1 PE=1 SV=1	18.2	8.82	PIN1
Q9ULC3	Ras-related protein Rab-23 OS=Homo sapiens OX=9606 GN=RAB23 PE=1 SV=1	26.6	6.6	RAB23
Q86VS8	Protein Hook homolog 3 OS=Homo sapiens OX=9606 GN=HOOK3 PE=1 SV=2	83.1	5.17	HOOK3
Q9Y5M8	Signal recognition particle receptor subunit beta OS=Homo sapiens OX=9606 GN=SRPRB PE=1 SV=3	29.7	9.04	SRPRB
O75844	CAAX prenyl protease 1 homolog OS=Homo sapiens OX=9606 GN=ZMPSTE24 PE=1 SV=2	54.8	7.49	ZMPSTE24
P10253	Lysosomal alpha-glucosidase OS=Homo sapiens OX=9606 GN=GAA PE=1 SV=4	105.3	6	GAA
P62942	Peptidyl-prolyl cis-trans isomerase FKBP1A OS=Homo sapiens OX=9606 GN=FKBP1A PE=1 SV=2	11.9	8.16	FKBP1A
Q9H8Y8	Golgi reassembly-stacking protein 2 OS=Homo sapiens OX=9606 GN=GORASP2 PE=1 SV=3	47.1	4.82	GORASP2
Q9H7Z7	Prostaglandin E synthase 2 OS=Homo sapiens OX=9606 GN=PTGES2 PE=1 SV=1	41.9	9.16	PTGES2
P43246	DNA mismatch repair protein Msh2 OS=Homo sapiens OX=9606 GN=MSH2 PE=1 SV=1	104.7	5.77	MSH2
Q8WX93	Palladin OS=Homo sapiens OX=9606 GN=PALLD PE=1 SV=3	150.5	7.09	PALLD
O15382	Branched-chain-amino-acid aminotransferase, mitochondrial OS=Homo sapiens OX=9606 GN=BCAT2 PE=1 SV=2	44.3	8.65	BCAT2

Q12965	Unconventional myosin-Ie OS=Homo sapiens OX=9606 GN=MYO1E PE=1 SV=2	127	8.92	MYO1E
P07093	Glia-derived nexin OS=Homo sapiens OX=9606 GN=SERPINE2 PE=1 SV=1	44	9.29	SERPINE2
Q9H3P7	Golgi resident protein GCP60 OS=Homo sapiens OX=9606 GN=ACBD3 PE=1 SV=4	60.6	5.06	ACBD3
P20020	Plasma membrane calcium-transporting ATPase 1 OS=Homo sapiens OX=9606 GN=ATP2B1 PE=1 SV=4	134.6	5.91	ATP2B1
Q9NTZ6	RNA-binding protein 12 OS=Homo sapiens OX=9606 GN=RBM12 PE=1 SV=1	97.3	8.63	RBM12
Q96E11	Ribosome-recycling factor, mitochondrial OS=Homo sapiens OX=9606 GN=MRRF PE=1 SV=1	29.3	9.79	MRRF
Q9H1E3	Nuclear ubiquitous casein and cyclin-dependent kinase substrate 1 OS=Homo sapiens OX=9606 GN=NUCKS1 PE=1 SV=1	27.3	5.08	NUCKS1
P55263	Adenosine kinase OS=Homo sapiens OX=9606 GN=ADK PE=1 SV=2	40.5	6.7	ADK
Q14165	Malectin OS=Homo sapiens OX=9606 GN=MLEC PE=1 SV=1	32.2	5.41	MLEC
O75937	DnaJ homolog subfamily C member 8 OS=Homo sapiens OX=9606 GN=DNAJC8 PE=1 SV=2	29.8	9.06	DNAJC8
P35222	Catenin beta-1 OS=Homo sapiens OX=9606 GN=CTNNB1 PE=1 SV=1	85.4	5.86	CTNNB1
O75746	Calcium-binding mitochondrial carrier protein Aralar1 OS=Homo sapiens OX=9606 GN=SLC25A12 PE=1 SV=2	74.7	8.38	SLC25A12
Q13243	Serine/arginine-rich splicing factor 5 OS=Homo sapiens OX=9606 GN=SRSF5 PE=1 SV=1	31.2	11.59	SRSF5
Q9HAV0	Guanine nucleotide-binding protein subunit beta-4 OS=Homo sapiens OX=9606 GN=GNB4 PE=1 SV=3	37.5	6	GNB4
Q16890	Tumor protein D53 OS=Homo sapiens OX=9606 GN=TPD52L1 PE=1 SV=1	22.4	5.62	TPD52L1
O60762	Dolichol-phosphate mannosyltransferase subunit 1 OS=Homo sapiens OX=9606 GN=DPM1 PE=1 SV=1	29.6	9.57	DPM1

Q7L014	Probable ATP-dependent RNA helicase DDX46 OS=Homo sapiens OX=9606 GN=DDX46 PE=1 SV=2	117.3	9.29	DDX46
P11908	Ribose-phosphate pyrophosphokinase 2 OS=Homo sapiens OX=9606 GN=PRPS2 PE=1 SV=2	34.7	6.61	PRPS2
Q14677	Clathrin interactor 1 OS=Homo sapiens OX=9606 GN=CLINT1 PE=1 SV=1	68.2	6.42	CLINT1
Q9Y5K6	CD2-associated protein OS=Homo sapiens OX=9606 GN=CD2AP PE=1 SV=1	71.4	6.4	CD2AP
Q9BRF8	Serine/threonine-protein phosphatase CPPED1 OS=Homo sapiens OX=9606 GN=CPPED1 PE=1 SV=3	35.5	6.2	CPPED1
Q9UH65	Switch-associated protein 70 OS=Homo sapiens OX=9606 GN=SWAP70 PE=1 SV=1	69	5.87	SWAP70
P22059	Oxysterol-binding protein 1 OS=Homo sapiens OX=9606 GN=OSBP PE=1 SV=1	89.4	7.3	OSBP
P83916	Chromobox protein homolog 1 OS=Homo sapiens OX=9606 GN=CBX1 PE=1 SV=1	21.4	4.93	CBX1
Q14166	Tubulin--tyrosine ligase-like protein 12 OS=Homo sapiens OX=9606 GN=TTLL12 PE=1 SV=2	74.4	5.53	TTLL12
Q9NZ01	Very-long-chain enoyl-CoA reductase OS=Homo sapiens OX=9606 GN=TECR PE=1 SV=1	36	9.45	TECR
P84103	Serine/arginine-rich splicing factor 3 OS=Homo sapiens OX=9606 GN=SRSF3 PE=1 SV=1	19.3	11.65	SRSF3
Q08945	FACT complex subunit SSRP1 OS=Homo sapiens OX=9606 GN=SSRP1 PE=1 SV=1	81	6.87	SSRP1
O15260	Surfeit locus protein 4 OS=Homo sapiens OX=9606 GN=SURF4 PE=1 SV=3	30.4	7.78	SURF4
P43304	Glycerol-3-phosphate dehydrogenase, mitochondrial OS=Homo sapiens OX=9606 GN=GPD2 PE=1 SV=3	80.8	7.69	GPD2
P62877	E3 ubiquitin-protein ligase RBX1 OS=Homo sapiens OX=9606 GN=RBX1 PE=1 SV=1	12.3	6.96	RBX1
Q8N3E9	1-phosphatidylinositol 4,5-bisphosphate phosphodiesterase delta-3 OS=Homo sapiens OX=9606 GN=PLCD3 PE=1 SV=3	89.2	6.98	PLCD3

Q9Y5A9	YTH domain-containing family protein 2 OS=Homo sapiens OX=9606 GN=YTHDF2 PE=1 SV=2	62.3	8.79	YTHDF2
Q9UNF1	Melanoma-associated antigen D2 OS=Homo sapiens OX=9606 GN=MAGED2 PE=1 SV=2	64.9	9.32	MAGED2
Q9UKV3	Apoptotic chromatin condensation inducer in the nucleus OS=Homo sapiens OX=9606 GN=ACIN1 PE=1 SV=2	151.8	6.43	ACIN1
O15067	Phosphoribosylformylglycinamidine synthase OS=Homo sapiens OX=9606 GN=PFAS PE=1 SV=4	144.6	5.76	PFAS
P13073	Cytochrome c oxidase subunit 4 isoform 1, mitochondrial OS=Homo sapiens OX=9606 GN=COX4I1 PE=1 SV=1	19.6	9.51	COX4I1
P62995	Transformer-2 protein homolog beta OS=Homo sapiens OX=9606 GN=TRA2B PE=1 SV=1	33.6	11.25	TRA2B
O15118	NPC intracellular cholesterol transporter 1 OS=Homo sapiens OX=9606 GN=NPC1 PE=1 SV=2	142.1	5.36	NPC1
O15347	High mobility group protein B3 OS=Homo sapiens OX=9606 GN=HMGB3 PE=1 SV=4	23	8.37	HMGB3
O43169	Cytochrome b5 type B OS=Homo sapiens OX=9606 GN=CYB5B PE=1 SV=3	16.7	4.97	CYB5B
O43598	2'-deoxynucleoside 5'-phosphate N-hydrolase 1 OS=Homo sapiens OX=9606 GN=DNPH1 PE=1 SV=1	19.1	5.05	DNPH1
Q13617	Cullin-2 OS=Homo sapiens OX=9606 GN=CUL2 PE=1 SV=2	86.9	6.92	CUL2
Q13098	COP9 signalosome complex subunit 1 OS=Homo sapiens OX=9606 GN=GPS1 PE=1 SV=4	55.5	6.74	GPS1
Q99598	Translin-associated protein X OS=Homo sapiens OX=9606 GN=TSNAX PE=1 SV=1	33.1	6.55	TSNAX
O43847	Nardilysin OS=Homo sapiens OX=9606 GN=NRDC PE=1 SV=3	131.6	4.98	NRDC
P11166	Solute carrier family 2, facilitated glucose transporter member 1 OS=Homo sapiens OX=9606 GN=SLC2A1 PE=1 SV=2	54	8.72	SLC2A1

Q9Y512	Sorting and assembly machinery component 50 homolog OS=Homo sapiens OX=9606 GN=SAMM50 PE=1 SV=3	51.9	6.9	SAMM50
Q96C86	m7GpppX diphosphatase OS=Homo sapiens OX=9606 GN=DCPS PE=1 SV=2	38.6	6.38	DCPS
Q13153	Serine/threonine-protein kinase PAK 1 OS=Homo sapiens OX=9606 GN=PAK1 PE=1 SV=2	60.6	5.76	PAK1
O00170	AH receptor-interacting protein OS=Homo sapiens OX=9606 GN=AIP PE=1 SV=2	37.6	6.29	
Q9UNF0	Protein kinase C and casein kinase substrate in neurons protein 2 OS=Homo sapiens OX=9606 GN=PACSIN2 PE=1 SV=2	55.7	5.2	PACSIN2
O75821	Eukaryotic translation initiation factor 3 subunit G OS=Homo sapiens OX=9606 GN=EIF3G PE=1 SV=2	35.6	6.13	EIF3G
Q16630	Cleavage and polyadenylation specificity factor subunit 6 OS=Homo sapiens OX=9606 GN=CPSF6 PE=1 SV=2	59.2	7.15	CPSF6
P61758	Prefoldin subunit 3 OS=Homo sapiens OX=9606 GN=VBP1 PE=1 SV=4	22.6	7.11	VBP1
Q02543	60S ribosomal protein L18a OS=Homo sapiens OX=9606 GN=RPL18A PE=1 SV=2	20.7	10.71	RPL18A
P28799	Progranulin OS=Homo sapiens OX=9606 GN=GRN PE=1 SV=2	63.5	6.83	GRN
P05026	Sodium/potassium-transporting ATPase subunit beta-1 OS=Homo sapiens OX=9606 GN=ATP1B1 PE=1 SV=1	35	8.53	ATP1B1
Q9HAV7	GrpE protein homolog 1, mitochondrial OS=Homo sapiens OX=9606 GN=GRPEL1 PE=1 SV=2	24.3	8.12	GRPEL1
Q92520	Protein FAM3C OS=Homo sapiens OX=9606 GN=FAM3C PE=1 SV=1	24.7	8.29	FAM3C
P53041	Serine/threonine-protein phosphatase 5 OS=Homo sapiens OX=9606 GN=PPP5C PE=1 SV=1	56.8	6.28	PPP5C
P10301	Ras-related protein R-Ras OS=Homo sapiens OX=9606 GN=RRAS PE=1 SV=1	23.5	6.93	RRAS

O76031	ATP-dependent Clp protease ATP-binding subunit clpX-like, mitochondrial OS=Homo sapiens OX=9606 GN=CLPX PE=1 SV=2	69.2	7.58	CLPX
P34896	Serine hydroxymethyltransferase, cytosolic OS=Homo sapiens OX=9606 GN=SHMT1 PE=1 SV=1	53	7.71	SHMT1
Q13938	Calcyphosin OS=Homo sapiens OX=9606 GN=CAPS PE=1 SV=2	30.2	6.04	CAPS
Q9P289	Serine/threonine-protein kinase 26 OS=Homo sapiens OX=9606 GN=STK26 PE=1 SV=2	46.5	5.29	STK26
Q9GZT3	SRA stem-loop-interacting RNA-binding protein, mitochondrial OS=Homo sapiens OX=9606 GN=SLIRP PE=1 SV=1	12.3	10.24	SLIRP
O43583	Density-regulated protein OS=Homo sapiens OX=9606 GN=DENR PE=1 SV=2	22.1	5.3	DENR
P12955	Xaa-Pro dipeptidase OS=Homo sapiens OX=9606 GN=PEPD PE=1 SV=3	54.5	6	PEPD
Q7Z794	Keratin, type II cytoskeletal 1b OS=Homo sapiens OX=9606 GN=KRT77 PE=1 SV=3	61.9	5.99	KRT77
Q9NUL3	Double-stranded RNA-binding protein Staufen homolog 2 OS=Homo sapiens OX=9606 GN=STAU2 PE=1 SV=2	62.6	9.61	STAU2
Q9NUJ1	Palmitoyl-protein thioesterase ABHD10, mitochondrial OS=Homo sapiens OX=9606 GN=ABHD10 PE=1 SV=1	33.9	8.57	ABHD10
Q6P996	Pyridoxal-dependent decarboxylase domain-containing protein 1 OS=Homo sapiens OX=9606 GN=PDXDC1 PE=1 SV=2	86.7	5.38	PDXDC1
Q92905	COP9 signalosome complex subunit 5 OS=Homo sapiens OX=9606 GN=COPS5 PE=1 SV=4	37.6	6.54	COPS5
P46779	60S ribosomal protein L28 OS=Homo sapiens OX=9606 GN=RPL28 PE=1 SV=3	15.7	12.02	RPL28
P32970	CD70 antigen OS=Homo sapiens OX=9606 GN=CD70 PE=1 SV=2	21.1	8.53	CD70
Q9NR30	Nucleolar RNA helicase 2 OS=Homo sapiens OX=9606 GN=DDX21 PE=1 SV=5	87.3	9.28	DDX21

Q92930	Ras-related protein Rab-8B OS=Homo sapiens OX=9606 GN=RAB8B PE=1 SV=2	23.6	9.07	RAB8B
P48739	Phosphatidylinositol transfer protein beta isoform OS=Homo sapiens OX=9606 GN=PITPNB PE=1 SV=2	31.5	6.87	PITPNB
Q14344	Guanine nucleotide-binding protein subunit alpha-13 OS=Homo sapiens OX=9606 GN=GNA13 PE=1 SV=2	44	8	GNA13
Q8IX12	Cell division cycle and apoptosis regulator protein 1 OS=Homo sapiens OX=9606 GN=CCAR1 PE=1 SV=2	132.7	5.76	CCAR1
Q9H074	Polyadenylate-binding protein-interacting protein 1 OS=Homo sapiens OX=9606 GN=PAIP1 PE=1 SV=1	53.5	4.81	PAIP1
P06703	Protein S100-A6 OS=Homo sapiens OX=9606 GN=S100A6 PE=1 SV=1	10.2	5.48	S100A6
Q14232	Translation initiation factor eIF-2B subunit alpha OS=Homo sapiens OX=9606 GN=EIF2B1 PE=1 SV=1	33.7	7.33	EIF2B1
Q13363	C-terminal-binding protein 1 OS=Homo sapiens OX=9606 GN=CTBP1 PE=1 SV=2	47.5	6.77	CTBP1
P83731	60S ribosomal protein L24 OS=Homo sapiens OX=9606 GN=RPL24 PE=1 SV=1	17.8	11.25	RPL24
P09496	Clathrin light chain A OS=Homo sapiens OX=9606 GN=CLTA PE=1 SV=1	27.1	4.51	CLTA
Q7Z4V5	Hepatoma-derived growth factor-related protein 2 OS=Homo sapiens OX=9606 GN=HDGFL2 PE=1 SV=1	74.3	7.49	HDGFL2
Q9UNS2	COP9 signalosome complex subunit 3 OS=Homo sapiens OX=9606 GN=COPS3 PE=1 SV=3	47.8	6.65	COPS3
O15460	Prolyl 4-hydroxylase subunit alpha-2 OS=Homo sapiens OX=9606 GN=P4HA2 PE=1 SV=1	60.9	5.71	P4HA2
P33992	DNA replication licensing factor MCM5 OS=Homo sapiens OX=9606 GN=MCM5 PE=1 SV=5	82.2	8.37	MCM5
Q9UFN0	Protein NipSnap homolog 3A OS=Homo sapiens OX=9606 GN=NIPSNAP3A PE=1 SV=2	28.4	9.16	NIPSNAP3A
Q9Y5X1	Sorting nexin-9 OS=Homo sapiens OX=9606 GN=SNX9 PE=1 SV=1	66.6	5.58	SNX9

Q9H299	SH3 domain-binding glutamic acid-rich-like protein 3 OS=Homo sapiens OX=9606 GN=SH3BGRL3 PE=1 SV=1	10.4	4.93	SH3BGRL3
Q00796	Sorbitol dehydrogenase OS=Homo sapiens OX=9606 GN=SORD PE=1 SV=4	38.3	7.97	SORD
Q16186	Proteasomal ubiquitin receptor ADRM1 OS=Homo sapiens OX=9606 GN=ADRM1 PE=1 SV=2	42.1	5.07	ADRM1
Q86Y46	Keratin, type II cytoskeletal 73 OS=Homo sapiens OX=9606 GN=KRT73 PE=1 SV=1	58.9	7.23	KRT73
O00442	RNA 3'-terminal phosphate cyclase OS=Homo sapiens OX=9606 GN=RTCA PE=1 SV=1	39.3	7.85	RTCA
Q02952	A-kinase anchor protein 12 OS=Homo sapiens OX=9606 GN=AKAP12 PE=1 SV=4	191.4	4.41	AKAP12
Q06787	Synaptic functional regulator FMR1 OS=Homo sapiens OX=9606 GN=FMR1 PE=1 SV=1	71.1	7.42	FMR1
P16930	Fumarylacetoacetase OS=Homo sapiens OX=9606 GN=FAH PE=1 SV=2	46.3	6.95	FAH
P08574	Cytochrome c1, heme protein, mitochondrial OS=Homo sapiens OX=9606 GN=CYC1 PE=1 SV=3	35.4	9	CYC1
P10321	HLA class I histocompatibility antigen, C alpha chain OS=Homo sapiens OX=9606 GN=HLA-C PE=1 SV=3	40.6	6.04	HLA-C
P04062	Lysosomal acid glucosylceramidase OS=Homo sapiens OX=9606 GN=GBA PE=1 SV=3	59.7	7.61	GBA
P62266	40S ribosomal protein S23 OS=Homo sapiens OX=9606 GN=RPS23 PE=1 SV=3	15.8	10.49	RPS23
P43155	Carnitine O-acetyltransferase OS=Homo sapiens OX=9606 GN=CRAT PE=1 SV=5	70.8	8.44	CRAT
Q99436	Proteasome subunit beta type-7 OS=Homo sapiens OX=9606 GN=PSMB7 PE=1 SV=1	29.9	7.68	PSMB7
O00186	Syntaxin-binding protein 3 OS=Homo sapiens OX=9606 GN=STXBP3 PE=1 SV=2	67.7	7.8	STXBP3
Q8TEA8	D-aminoacyl-tRNA deacylase 1 OS=Homo sapiens OX=9606 GN=DTD1 PE=1 SV=2	23.4	8.24	DTD1

P62854	40S ribosomal protein S26 OS=Homo sapiens OX=9606 GN=RPS26 PE=1 SV=3	13	11	RPS26
Q9NX62	Golgi-resident adenosine 3',5'-bisphosphate 3'-phosphatase OS=Homo sapiens OX=9606 GN=BPNT2 PE=1 SV=1	38.7	6.86	BPNT2
P51452	Dual specificity protein phosphatase 3 OS=Homo sapiens OX=9606 GN=DUSP3 PE=1 SV=1	20.5	7.8	DUSP3
Q14738	Serine/threonine-protein phosphatase 2A 56 kDa regulatory subunit delta isoform OS=Homo sapiens OX=9606 GN=PPP2R5D PE=1 SV=1	69.9	8.13	PPP2R5D
Q9Y520	Protein PRRC2C OS=Homo sapiens OX=9606 GN=PRRC2C PE=1 SV=4	316.7	9.13	PRRC2C
Q8N684	Cleavage and polyadenylation specificity factor subunit 7 OS=Homo sapiens OX=9606 GN=CPSF7 PE=1 SV=1	52	8	CPSF7
O43324	Eukaryotic translation elongation factor 1 epsilon-1 OS=Homo sapiens OX=9606 GN=EEF1E1 PE=1 SV=1	19.8	8.54	EEF1E1
Q56VL3	OCIA domain-containing protein 2 OS=Homo sapiens OX=9606 GN=OCIAD2 PE=1 SV=1	16.9	9.03	OCIAD2
Q9GZS3	WD repeat-containing protein 61 OS=Homo sapiens OX=9606 GN=WDR61 PE=1 SV=1	33.6	5.47	WDR61
P45973	Chromobox protein homolog 5 OS=Homo sapiens OX=9606 GN=CBX5 PE=1 SV=1	22.2	5.86	CBX5
Q16539	Mitogen-activated protein kinase 14 OS=Homo sapiens OX=9606 GN=MAPK14 PE=1 SV=3	41.3	5.78	MAPK14
O15173	Membrane-associated progesterone receptor component 2 OS=Homo sapiens OX=9606 GN=PGRMC2 PE=1 SV=1	23.8	4.88	PGRMC2
Q96HS1	Serine/threonine-protein phosphatase PGAM5, mitochondrial OS=Homo sapiens OX=9606 GN=PGAM5 PE=1 SV=2	32	8.68	PGAM5
P61081	NEDD8-conjugating enzyme Ubc12 OS=Homo sapiens OX=9606 GN=UBE2M PE=1 SV=1	20.9	7.69	UBE2M

Q969U7	Proteasome assembly chaperone 2 OS=Homo sapiens OX=9606 GN=PSMG2 PE=1 SV=1	29.4	6.98	PSMG2
P30046	D-dopachrome decarboxylase OS=Homo sapiens OX=9606 GN=DDT PE=1 SV=3	12.7	7.3	DDT
O95747	Serine/threonine-protein kinase OSR1 OS=Homo sapiens OX=9606 GN=OXSR1 PE=1 SV=1	58	6.43	OXSR1
P43307	Translocon-associated protein subunit alpha OS=Homo sapiens OX=9606 GN=SSR1 PE=1 SV=3	32.2	4.49	SSR1
Q08380	Galectin-3-binding protein OS=Homo sapiens OX=9606 GN=LGALS3BP PE=1 SV=1	65.3	5.27	LGALS3BP
P78386	Keratin, type II cuticular Hb5 OS=Homo sapiens OX=9606 GN=KRT85 PE=1 SV=1	55.8	6.55	KRT85
P33991	DNA replication licensing factor MCM4 OS=Homo sapiens OX=9606 GN=MCM4 PE=1 SV=5	96.5	6.74	MCM4
Q9NX40	OCIA domain-containing protein 1 OS=Homo sapiens OX=9606 GN=OCIAD1 PE=1 SV=1	27.6	7.49	OCIAD1
Q96M27	Protein PRRC1 OS=Homo sapiens OX=9606 GN=PRRC1 PE=1 SV=1	46.7	5.83	PRRC1
Q9NRN7	L-aminoadipate-semialdehyde dehydrogenase-phosphopantetheinyl transferase OS=Homo sapiens OX=9606 GN=AASDHPPT PE=1 SV=2	35.8	6.8	AASDHPPT
Q86U42	Polyadenylate-binding protein 2 OS=Homo sapiens OX=9606 GN=PABPN1 PE=1 SV=3	32.7	5.06	PABPN1
O75306	NADH dehydrogenase [ubiquinone] iron-sulfur protein 2, mitochondrial OS=Homo sapiens OX=9606 GN=NDUFS2 PE=1 SV=2	52.5	7.55	NDUFS2
P63165	Small ubiquitin-related modifier 1 OS=Homo sapiens OX=9606 GN=SUMO1 PE=1 SV=1	11.6	5.52	SUMO1
Q9P015	39S ribosomal protein L15, mitochondrial OS=Homo sapiens OX=9606 GN=MRPL15 PE=1 SV=1	33.4	10.01	MRPL15
P23258	Tubulin gamma-1 chain OS=Homo sapiens OX=9606 GN=TUBG1 PE=1 SV=2	51.1	6.14	TUBG1

P24941	Cyclin-dependent kinase 2 OS=Homo sapiens OX=9606 GN=CDK2 PE=1 SV=2	33.9	8.68	CDK2
Q9Y4Z0	U6 snRNA-associated Sm-like protein LSm4 OS=Homo sapiens OX=9606 GN=LSM4 PE=1 SV=1	15.3	9.99	LSM4
Q12765	Secernin-1 OS=Homo sapiens OX=9606 GN=SCRN1 PE=1 SV=2	46.4	4.75	SCRN1
Q7L0Y3	tRNA methyltransferase 10 homolog C OS=Homo sapiens OX=9606 GN=TRMT10C PE=1 SV=2	47.3	9.36	TRMT10C
P05387	60S acidic ribosomal protein P2 OS=Homo sapiens OX=9606 GN=RPLP2 PE=1 SV=1	11.7	4.54	RPLP2
P30876	DNA-directed RNA polymerase II subunit RPB2 OS=Homo sapiens OX=9606 GN=POLR2B PE=1 SV=1	133.8	6.87	POLR2B
Q9P0J0	NADH dehydrogenase [ubiquinone] 1 alpha subcomplex subunit 13 OS=Homo sapiens OX=9606 GN=NDUFA13 PE=1 SV=3	16.7	8.43	NDUFA13
P68402	Platelet-activating factor acetylhydrolase IB subunit alpha2 OS=Homo sapiens OX=9606 GN=PAFAH1B2 PE=1 SV=1	25.6	5.92	PAFAH1B2
Q13268	Dehydrogenase/reductase SDR family member 2, mitochondrial OS=Homo sapiens OX=9606 GN=DHRS2 PE=1 SV=4	29.9	9.01	DHRS2
O43592	Exportin-T OS=Homo sapiens OX=9606 GN=XPOT PE=1 SV=2	109.9	5.39	XPOT
Q8TC12	Retinol dehydrogenase 11 OS=Homo sapiens OX=9606 GN=RDH11 PE=1 SV=2	35.4	8.82	RDH11
O00233	26S proteasome non-ATPase regulatory subunit 9 OS=Homo sapiens OX=9606 GN=PSMD9 PE=1 SV=3	24.7	6.95	PSMD9
P62330	ADP-ribosylation factor 6 OS=Homo sapiens OX=9606 GN=ARF6 PE=1 SV=2	20.1	8.95	ARF6
P21291	Cysteine and glycine-rich protein 1 OS=Homo sapiens OX=9606 GN=CSRP1 PE=1 SV=3	20.6	8.57	CSRP1
P25685	DnaJ homolog subfamily B member 1 OS=Homo sapiens OX=9606 GN=DNAJB1 PE=1 SV=4	38	8.63	DNAJB1

Q12888	TP53-binding protein 1 OS=Homo sapiens OX=9606 GN=TP53BP1 PE=1 SV=2	213.4	4.7	TP53BP1
Q92804	TATA-binding protein-associated factor 2N OS=Homo sapiens OX=9606 GN=TAF15 PE=1 SV=1	61.8	8.02	TAF15
Q9NR56	Muscleblind-like protein 1 OS=Homo sapiens OX=9606 GN=MBNL1 PE=1 SV=2	41.8	8.9	MBNL1
P09874	Poly [ADP-ribose] polymerase 1 OS=Homo sapiens OX=9606 GN=PARP1 PE=1 SV=4	113	8.88	PARP1
O00625	Pirin OS=Homo sapiens OX=9606 GN=PIR PE=1 SV=1	32.1	6.92	PIR
Q9UI09	NADH dehydrogenase [ubiquinone] 1 alpha subcomplex subunit 12 OS=Homo sapiens OX=9606 GN=NDUFA12 PE=1 SV=1	17.1	9.63	NDUFA12
Q6PKG0	La-related protein 1 OS=Homo sapiens OX=9606 GN=LARP1 PE=1 SV=2	123.4	8.82	LARP1
Q13131	5'-AMP-activated protein kinase catalytic subunit alpha-1 OS=Homo sapiens OX=9606 GN=PRKAA1 PE=1 SV=4	64	8.12	PRKAA1
P11233	Ras-related protein Ral-A OS=Homo sapiens OX=9606 GN=RALA PE=1 SV=1	23.6	7.11	RALA
P50148	Guanine nucleotide-binding protein G(q) subunit alpha OS=Homo sapiens OX=9606 GN=GNAQ PE=1 SV=4	42.1	5.68	GNAQ
P46108	Adapter molecule crk OS=Homo sapiens OX=9606 GN=CRK PE=1 SV=2	33.8	5.55	CRK
O94832	Unconventional myosin-Id OS=Homo sapiens OX=9606 GN=MYO1D PE=1 SV=2	116.1	9.39	MYO1D
Q9Y570	Protein phosphatase methylesterase 1 OS=Homo sapiens OX=9606 GN=PPME1 PE=1 SV=3	42.3	5.97	PPME1
Q9UBQ0	Vacuolar protein sorting-associated protein 29 OS=Homo sapiens OX=9606 GN=VPS29 PE=1 SV=1	20.5	6.79	VPS29
Q9NR12	PDZ and LIM domain protein 7 OS=Homo sapiens OX=9606 GN=PDLIM7 PE=1 SV=1	49.8	8.41	PDLIM7
P35250	Replication factor C subunit 2 OS=Homo sapiens OX=9606 GN=RFC2 PE=1 SV=3	39.1	6.44	RFC2

Q9Y305	Acyl-coenzyme A thioesterase 9, mitochondrial OS=Homo sapiens OX=9606 GN=ACOT9 PE=1 SV=2	49.9	8.6	ACOT9
Q13620	Cullin-4B OS=Homo sapiens OX=9606 GN=CUL4B PE=1 SV=4	103.9	7.37	CUL4B
P25398	40S ribosomal protein S12 OS=Homo sapiens OX=9606 GN=RPS12 PE=1 SV=3	14.5	7.21	RPS12
Q9BZK3	Putative nascent polypeptide-associated complex subunit alpha-like protein OS=Homo sapiens OX=9606 GN=NACA4P PE=5 SV=1	23.3	4.59	NACA4P
Q9NQ29	Putative RNA-binding protein Luc7-like 1 OS=Homo sapiens OX=9606 GN=LUC7L PE=1 SV=1	43.7	9.92	LUC7L
Q8WXF1	Paraspeckle component 1 OS=Homo sapiens OX=9606 GN=PSPC1 PE=1 SV=1	58.7	6.67	PSPC1
Q9H6T3	RNA polymerase II-associated protein 3 OS=Homo sapiens OX=9606 GN=RPAP3 PE=1 SV=2	75.7	6.84	RPAP3
O75368	SH3 domain-binding glutamic acid-rich-like protein OS=Homo sapiens OX=9606 GN=SH3BGRL PE=1 SV=1	12.8	5.25	SH3BGRL
P09669	Cytochrome c oxidase subunit 6C OS=Homo sapiens OX=9606 GN=COX6C PE=1 SV=2	8.8	10.39	COX6C
P62993	Growth factor receptor-bound protein 2 OS=Homo sapiens OX=9606 GN=GRB2 PE=1 SV=1	25.2	6.32	GRB2
Q9UBV8	Peflin OS=Homo sapiens OX=9606 GN=PEF1 PE=1 SV=1	30.4	6.54	PEF1
Q70UQ0	Inhibitor of nuclear factor kappa-B kinase-interacting protein OS=Homo sapiens OX=9606 GN=IKBIP PE=1 SV=1	39.3	9.17	IKBIP
Q96N66	Lysophospholipid acyltransferase 7 OS=Homo sapiens OX=9606 GN=MBOAT7 PE=1 SV=2	52.7	8.97	MBOAT7
Q9NUQ6	SPATS2-like protein OS=Homo sapiens OX=9606 GN=SPATS2L PE=1 SV=2	61.7	9.64	SPATS2L
Q9H832	Ubiquitin-conjugating enzyme E2 Z OS=Homo sapiens OX=9606 GN=UBE2Z PE=1 SV=2	38.2	5.62	UBE2Z
Q9NYK5	39S ribosomal protein L39, mitochondrial OS=Homo sapiens OX=9606 GN=MRPL39 PE=1 SV=3	38.7	7.65	MRPL39

Q14320	Protein FAM50A OS=Homo sapiens OX=9606 GN=FAM50A PE=1 SV=2	40.2	6.83	FAM50A
Q96G23	Ceramide synthase 2 OS=Homo sapiens OX=9606 GN=CERS2 PE=1 SV=1	44.8	8.98	CERS2
P48449	Lanosterol synthase OS=Homo sapiens OX=9606 GN=LSS PE=1 SV=1	83.3	6.61	LSS
P59998	Actin-related protein 2/3 complex subunit 4 OS=Homo sapiens OX=9606 GN=ARPC4 PE=1 SV=3	19.7	8.43	ARPC4
P67812	Signal peptidase complex catalytic subunit SEC11A OS=Homo sapiens OX=9606 GN=SEC11A PE=1 SV=1	20.6	9.48	SEC11A
O95456	Proteasome assembly chaperone 1 OS=Homo sapiens OX=9606 GN=PSMG1 PE=1 SV=1	32.8	7.17	PSMG1
Q9Y333	U6 snRNA-associated Sm-like protein LSm2 OS=Homo sapiens OX=9606 GN=LSM2 PE=1 SV=1	10.8	6.52	LSM2
Q15428	Splicing factor 3A subunit 2 OS=Homo sapiens OX=9606 GN=SF3A2 PE=1 SV=2	49.2	9.64	SF3A2
Q6Y7W6	GRB10-interacting GYF protein 2 OS=Homo sapiens OX=9606 GN=GIGYF2 PE=1 SV=1	150	5.54	GIGYF2
Q9Y5X3	Sorting nexin-5 OS=Homo sapiens OX=9606 GN=SNX5 PE=1 SV=1	46.8	6.76	SNX5
O95777	U6 snRNA-associated Sm-like protein LSm8 OS=Homo sapiens OX=9606 GN=LSM8 PE=1 SV=3	10.4	4.48	LSM8
P11047	Laminin subunit gamma-1 OS=Homo sapiens OX=9606 GN=LAMC1 PE=1 SV=3	177.5	5.12	LAMC1
Q9NVS9	Pyridoxine-5'-phosphate oxidase OS=Homo sapiens OX=9606 GN=PNPO PE=1 SV=1	30	7.06	PNPO
Q96PU8	Protein quaking OS=Homo sapiens OX=9606 GN=QKI PE=1 SV=1	37.6	8.56	QKI
O60271	C-Jun-amino-terminal kinase-interacting protein 4 OS=Homo sapiens OX=9606 GN=SPAG9 PE=1 SV=4	146.1	5.15	SPAG9
P56377	AP-1 complex subunit sigma-2 OS=Homo sapiens OX=9606 GN=AP1S2 PE=1 SV=1	18.6	5.47	AP1S2

P62851	40S ribosomal protein S25 OS=Homo sapiens OX=9606 GN=RPS25 PE=1 SV=1	13.7	10.11	RPS25
Q7L9L4	MOB kinase activator 1B OS=Homo sapiens OX=9606 GN=MOB1B PE=1 SV=3	25.1	6.73	MOB1B
O43837	Isocitrate dehydrogenase [NAD] subunit beta, mitochondrial OS=Homo sapiens OX=9606 GN=IDH3B PE=1 SV=2	42.2	8.46	IDH3B
Q15370	Elongin-B OS=Homo sapiens OX=9606 GN=ELOB PE=1 SV=1	13.1	4.88	ELOB
Q7Z739	YTH domain-containing family protein 3 OS=Homo sapiens OX=9606 GN=YTHDF3 PE=1 SV=1	63.8	9.04	YTHDF3
P49354	Protein farnesyltransferase/geranylgeranyltransferase type-1 subunit alpha OS=Homo sapiens OX=9606 GN=FNTA PE=1 SV=1	44.4	5.08	FNTA
Q9Y3L5	Ras-related protein Rap-2c OS=Homo sapiens OX=9606 GN=RAP2C PE=1 SV=1	20.7	4.94	RAP2C
Q6RW13	Type-1 angiotensin II receptor-associated protein OS=Homo sapiens OX=9606 GN=AGTRAP PE=1 SV=1	17.4	6.14	AGTRAP
Q9Y376	Calcium-binding protein 39 OS=Homo sapiens OX=9606 GN=CAB39 PE=1 SV=1	39.8	6.89	CAB39
Q15102	Platelet-activating factor acetylhydrolase IB subunit alpha1 OS=Homo sapiens OX=9606 GN=PAFAH1B3 PE=1 SV=1	25.7	6.84	PAFAH1B3
P18077	60S ribosomal protein L35a OS=Homo sapiens OX=9606 GN=RPL35A PE=1 SV=2	12.5	11.06	RPL35A
Q08J23	RNA cytosine C(5)-methyltransferase NSUN2 OS=Homo sapiens OX=9606 GN=NSUN2 PE=1 SV=2	86.4	6.77	NSUN2
P22830	Ferrochelatase, mitochondrial OS=Homo sapiens OX=9606 GN=FECH PE=1 SV=2	47.8	8.73	FECH
P31949	Protein S100-A11 OS=Homo sapiens OX=9606 GN=S100A11 PE=1 SV=2	11.7	7.12	S100A11
P18615	Negative elongation factor E OS=Homo sapiens OX=9606 GN=NELFE PE=1 SV=3	43.2	9.33	NELFE

Q9Y2X3	Nucleolar protein 58 OS=Homo sapiens OX=9606 GN=NOP58 PE=1 SV=1	59.5	8.92	NOP58
A5YKK6	CCR4-NOT transcription complex subunit 1 OS=Homo sapiens OX=9606 GN=CNOT1 PE=1 SV=2	266.8	7.11	CNOT1
O75521	Enoyl-CoA delta isomerase 2 OS=Homo sapiens OX=9606 GN=ECI2 PE=1 SV=4	43.6	9	ECI2
Q8TEX9	Importin-4 OS=Homo sapiens OX=9606 GN=IPO4 PE=1 SV=2	118.6	4.96	IPO4
Q14694	Ubiquitin carboxyl-terminal hydrolase 10 OS=Homo sapiens OX=9606 GN=USP10 PE=1 SV=2	87.1	5.31	USP10
P06730	Eukaryotic translation initiation factor 4E OS=Homo sapiens OX=9606 GN=EIF4E PE=1 SV=2	25.1	6.15	EIF4E
Q9UM00	Calcium load-activated calcium channel OS=Homo sapiens OX=9606 GN=TMCO1 PE=1 SV=2	27.1	10.26	TMCO1
Q93008	Probable ubiquitin carboxyl-terminal hydrolase FAF-X OS=Homo sapiens OX=9606 GN=USP9X PE=1 SV=4	290.3	5.8	USP9X
P61769	Beta-2-microglobulin OS=Homo sapiens OX=9606 GN=B2M PE=1 SV=1	13.7	6.52	B2M
P20336	Ras-related protein Rab-3A OS=Homo sapiens OX=9606 GN=RAB3A PE=1 SV=1	25	5.03	RAB3A
P19784	Casein kinase II subunit alpha' OS=Homo sapiens OX=9606 GN=CSNK2A2 PE=1 SV=1	41.2	8.56	CSNK2A2
Q9UHY1	Nuclear receptor-binding protein OS=Homo sapiens OX=9606 GN=NRBP1 PE=1 SV=1	59.8	5.08	NRBP1
Q9Y295	Developmentally-regulated GTP-binding protein 1 OS=Homo sapiens OX=9606 GN=DRG1 PE=1 SV=1	40.5	8.9	DRG1
Q13951	Core-binding factor subunit beta OS=Homo sapiens OX=9606 GN=CBFB PE=1 SV=2	21.5	6.6	CBFB
Q96A72	Protein mago nashi homolog 2 OS=Homo sapiens OX=9606 GN=MAGOHB PE=1 SV=1	17.3	6.39	MAGOHB
P57105	Synaptojanin-2-binding protein OS=Homo sapiens OX=9606 GN=SYNJ2BP PE=1 SV=2	15.9	6.3	SYNJ2BP

Q9Y2T2	AP-3 complex subunit mu-1 OS=Homo sapiens OX=9606 GN=AP3M1 PE=1 SV=1	46.9	6.93	AP3M1
Q9H4G0	Band 4.1-like protein 1 OS=Homo sapiens OX=9606 GN=EPB41L1 PE=1 SV=2	98.4	5.62	EPB41L1
Q12996	Cleavage stimulation factor subunit 3 OS=Homo sapiens OX=9606 GN=CSTF3 PE=1 SV=1	82.9	8.12	CSTF3
O60488	Long-chain-fatty-acid--CoA ligase 4 OS=Homo sapiens OX=9606 GN=ACSL4 PE=1 SV=2	79.1	8.38	ACSL4
P51153	Ras-related protein Rab-13 OS=Homo sapiens OX=9606 GN=RAB13 PE=1 SV=1	22.8	9.19	RAB13
Q15843	NEDD8 OS=Homo sapiens OX=9606 GN=NEDD8 PE=1 SV=1	9.1	8.43	NEDD8
O00425	Insulin-like growth factor 2 mRNA-binding protein 3 OS=Homo sapiens OX=9606 GN=IGF2BP3 PE=1 SV=2	63.7	8.87	IGF2BP3
Q9HAB8	Phosphopantothenate--cysteine ligase OS=Homo sapiens OX=9606 GN=PPCS PE=1 SV=2	34	6.71	PPCS
P61956	Small ubiquitin-related modifier 2 OS=Homo sapiens OX=9606 GN=SUMO2 PE=1 SV=3	10.9	5.5	SUMO2
P46976	Glycogenin-1 OS=Homo sapiens OX=9606 GN=GYG1 PE=1 SV=4	39.4	5.53	GYG1
Q9H3N1	Thioredoxin-related transmembrane protein 1 OS=Homo sapiens OX=9606 GN=TMX1 PE=1 SV=1	31.8	4.98	TMX1
Q9Y237	Peptidyl-prolyl cis-trans isomerase NIMA-interacting 4 OS=Homo sapiens OX=9606 GN=PIN4 PE=1 SV=1	13.8	9.77	PIN4
P61326	Protein mago nashi homolog OS=Homo sapiens OX=9606 GN=MAGOH PE=1 SV=1	17.2	6.11	MAGOH
Q9BPX5	Actin-related protein 2/3 complex subunit 5-like protein OS=Homo sapiens OX=9606 GN=ARPC5L PE=1 SV=1	16.9	6.6	ARPC5L
Q8NC56	LEM domain-containing protein 2 OS=Homo sapiens OX=9606 GN=LEMD2 PE=1 SV=1	56.9	9	LEMD2
Q86X55	Histone-arginine methyltransferase CARM1 OS=Homo sapiens OX=9606 GN=CARM1 PE=1 SV=3	65.8	6.73	CARM1

Q9UGP8	Translocation protein SEC63 homolog OS=Homo sapiens OX=9606 GN=SEC63 PE=1 SV=2	87.9	5.31	SEC63
P61457	Pterin-4-alpha-carbinolamine dehydratase OS=Homo sapiens OX=9606 GN=PCBD1 PE=1 SV=2	12	6.8	PCBD1
Q96TC7	Regulator of microtubule dynamics protein 3 OS=Homo sapiens OX=9606 GN=RMDN3 PE=1 SV=2	52.1	5.1	RMDN3
Q9UKG1	DCC-interacting protein 13-alpha OS=Homo sapiens OX=9606 GN=APPL1 PE=1 SV=1	79.6	5.41	APPL1
Q9BRK5	45 kDa calcium-binding protein OS=Homo sapiens OX=9606 GN=SDF4 PE=1 SV=1	41.8	4.86	SDF4
Q8TEM1	Nuclear pore membrane glycoprotein 210 OS=Homo sapiens OX=9606 GN=NUP210 PE=1 SV=3	205	6.81	NUP210
Q2M389	WASH complex subunit 4 OS=Homo sapiens OX=9606 GN=WASHC4 PE=1 SV=2	136.3	7.44	WASHC4
P56134	ATP synthase subunit f, mitochondrial OS=Homo sapiens OX=9606 GN=ATP5MF PE=1 SV=3	10.9	9.67	ATP5MF
O14745	Na(+)/H(+) exchange regulatory cofactor NHE-RF1 OS=Homo sapiens OX=9606 GN=SLC9A3R1 PE=1 SV=4	38.8	5.77	SLC9A3R1
Q8IV08	5'-3' exonuclease PLD3 OS=Homo sapiens OX=9606 GN=PLD3 PE=1 SV=1	54.7	6.47	PLD3
P61225	Ras-related protein Rap-2b OS=Homo sapiens OX=9606 GN=RAP2B PE=1 SV=1	20.5	4.81	RAP2B
Q14141	Septin-6 OS=Homo sapiens OX=9606 GN=SEPTIN6 PE=1 SV=4	49.7	6.67	SEPTIN6
Q9BWS9	Chitinase domain-containing protein 1 OS=Homo sapiens OX=9606 GN=CHID1 PE=1 SV=1	44.9	8.63	CHID1
Q96CT7	Coiled-coil domain-containing protein 124 OS=Homo sapiens OX=9606 GN=CCDC124 PE=1 SV=1	25.8	9.54	CCDC124
Q9BPW8	Protein NipSnap homolog 1 OS=Homo sapiens OX=9606 GN=NIPSNAP1 PE=1 SV=1	33.3	9.31	NIPSNAP1
O95470	Sphingosine-1-phosphate lyase 1 OS=Homo sapiens OX=9606 GN=SGPL1 PE=1 SV=3	63.5	9.16	SGPL1

P54619	5'-AMP-activated protein kinase subunit gamma-1 OS=Homo sapiens OX=9606 GN=PRKAG1 PE=1 SV=1	37.6	6.92	PRKAG1
O75477	Erlin-1 OS=Homo sapiens OX=9606 GN=ERLIN1 PE=1 SV=2	39.1	7.87	ERLIN1
Q9NXV6	CDKN2A-interacting protein OS=Homo sapiens OX=9606 GN=CDKN2AIP PE=1 SV=3	61.1	9.01	CDKN2AIP
Q96JB5	CDK5 regulatory subunit-associated protein 3 OS=Homo sapiens OX=9606 GN=CDK5RAP3 PE=1 SV=2	56.9	4.75	CDK5RAP3
P29317	Ephrin type-A receptor 2 OS=Homo sapiens OX=9606 GN=EPHA2 PE=1 SV=2	108.2	6.23	EPHA2
Q8IWE2	Protein NOXP20 OS=Homo sapiens OX=9606 GN=FAM114A1 PE=1 SV=2	60.7	4.68	FAM114A1
Q6P9B6	MTOR-associated protein MEAK7 OS=Homo sapiens OX=9606 GN=MEAK7 PE=1 SV=2	51	6.24	MEAK7
Q969X5	Endoplasmic reticulum-Golgi intermediate compartment protein 1 OS=Homo sapiens OX=9606 GN=ERGIC1 PE=1 SV=1	32.6	7.06	ERGIC1
P42785	Lysosomal Pro-X carboxypeptidase OS=Homo sapiens OX=9606 GN=PRCP PE=1 SV=1	55.8	7.21	PRCP
Q9P000	COMM domain-containing protein 9 OS=Homo sapiens OX=9606 GN=COMMD9 PE=1 SV=2	21.8	5.88	COMMD9
Q9BYD1	39S ribosomal protein L13, mitochondrial OS=Homo sapiens OX=9606 GN=MRPL13 PE=1 SV=1	20.7	9.16	MRPL13
P62745	Rho-related GTP-binding protein RhoB OS=Homo sapiens OX=9606 GN=RHOB PE=1 SV=1	22.1	5.24	RHOB
P29218	Inositol monophosphatase 1 OS=Homo sapiens OX=9606 GN=IMPA1 PE=1 SV=1	30.2	5.26	IMPA1
P18583	Protein SON OS=Homo sapiens OX=9606 GN=SON PE=1 SV=4	263.7	5.64	SON
Q96A35	39S ribosomal protein L24, mitochondrial OS=Homo sapiens OX=9606 GN=MRPL24 PE=1 SV=1	24.9	9.29	MRPL24

Q9HCC0	Methylcrotonoyl-CoA carboxylase beta chain, mitochondrial OS=Homo sapiens OX=9606 GN=MCCC2 PE=1 SV=1	61.3	7.68	MCCC2
P63313	Thymosin beta-10 OS=Homo sapiens OX=9606 GN=TMSB10 PE=1 SV=2	5	5.36	TMSB10
P06132	Uroporphyrinogen decarboxylase OS=Homo sapiens OX=9606 GN=UROD PE=1 SV=2	40.8	6.14	UROD
P61960	Ubiquitin-fold modifier 1 OS=Homo sapiens OX=9606 GN=UFM1 PE=1 SV=1	9.1	9.31	UFM1
Q15287	RNA-binding protein with serine-rich domain 1 OS=Homo sapiens OX=9606 GN=RNPS1 PE=1 SV=1	34.2	11.84	RNPS1
Q9NP81	Serine--tRNA ligase, mitochondrial OS=Homo sapiens OX=9606 GN=SARS2 PE=1 SV=1	58.2	8.13	SARS2
Q9P287	BRCA2 and CDKN1A-interacting protein OS=Homo sapiens OX=9606 GN=BCCIP PE=1 SV=1	36	4.61	BCCIP
Q9BYD3	39S ribosomal protein L4, mitochondrial OS=Homo sapiens OX=9606 GN=MRPL4 PE=1 SV=1	34.9	9.72	MRPL4
Q5D862	Filaggrin-2 OS=Homo sapiens OX=9606 GN=FLG2 PE=1 SV=1	247.9	8.31	FLG2
P63173	60S ribosomal protein L38 OS=Homo sapiens OX=9606 GN=RPL38 PE=1 SV=2	8.2	10.1	RPL38
Q00765	Receptor expression-enhancing protein 5 OS=Homo sapiens OX=9606 GN=REEP5 PE=1 SV=3	21.5	8.1	REEP5
P31483	Nucleolysin TIA-1 isoform p40 OS=Homo sapiens OX=9606 GN=TIA1 PE=1 SV=3	42.9	7.74	TIA1
P51398	28S ribosomal protein S29, mitochondrial OS=Homo sapiens OX=9606 GN=DAP3 PE=1 SV=1	45.5	8.88	DAP3
P07947	Tyrosine-protein kinase Yes OS=Homo sapiens OX=9606 GN=YES1 PE=1 SV=3	60.8	6.74	YES1
O14828	Secretory carrier-associated membrane protein 3 OS=Homo sapiens OX=9606 GN=SCAMP3 PE=1 SV=3	38.3	7.64	SCAMP3
Q969V3	Nicalin OS=Homo sapiens OX=9606 GN=NCLN PE=1 SV=2	62.9	6.89	NCLN

Q15738	Sterol-4-alpha-carboxylate 3-dehydrogenase, decarboxylating OS=Homo sapiens OX=9606 GN=NSDHL PE=1 SV=2	41.9	8.06	NSDHL
Q8IYB3	Serine/arginine repetitive matrix protein 1 OS=Homo sapiens OX=9606 GN=SRRM1 PE=1 SV=2	102.3	11.84	SRRM1
Q92542	Nicastrin OS=Homo sapiens OX=9606 GN=NCSTN PE=1 SV=2	78.4	5.99	NCSTN
P51970	NADH dehydrogenase [ubiquinone] 1 alpha subcomplex subunit 8 OS=Homo sapiens OX=9606 GN=NDUFA8 PE=1 SV=3	20.1	7.65	NDUFA8
P61513	60S ribosomal protein L37a OS=Homo sapiens OX=9606 GN=RPL37A PE=1 SV=2	10.3	10.43	RPL37A
Q96IX5	ATP synthase membrane subunit K, mitochondrial OS=Homo sapiens OX=9606 GN=ATP5MK PE=1 SV=1	6.5	9.76	ATP5MK
P56545	C-terminal-binding protein 2 OS=Homo sapiens OX=9606 GN=CTBP2 PE=1 SV=1	48.9	6.95	CTBP2
O43813	Glutathione S-transferase LANCL1 OS=Homo sapiens OX=9606 GN=LANCL1 PE=1 SV=1	45.3	7.75	LANCL1
Q16666	Gamma-interferon-inducible protein 16 OS=Homo sapiens OX=9606 GN=IFI16 PE=1 SV=3	88.2	9.28	IFI16
Q9GZP4	PITH domain-containing protein 1 OS=Homo sapiens OX=9606 GN=PITHD1 PE=1 SV=1	24.2	5.74	PITHD1
Q9H2U1	ATP-dependent DNA/RNA helicase DHX36 OS=Homo sapiens OX=9606 GN=DHX36 PE=1 SV=2	114.7	7.68	DHX36
Q9BZV1	UBX domain-containing protein 6 OS=Homo sapiens OX=9606 GN=UBXN6 PE=1 SV=1	49.7	6.89	UBXN6
P05114	Non-histone chromosomal protein HMG-14 OS=Homo sapiens OX=9606 GN=HMGN1 PE=1 SV=3	10.7	9.6	HMGN1
Q14849	StAR-related lipid transfer protein 3 OS=Homo sapiens OX=9606 GN=STARD3 PE=1 SV=2	50.5	8.37	STARD3
P61619	Protein transport protein Sec61 subunit alpha isoform 1 OS=Homo sapiens OX=9606 GN=SEC61A1 PE=1 SV=2	52.2	8.06	SEC61A1

P42566	Epidermal growth factor receptor substrate 15 OS=Homo sapiens OX=9606 GN=EPS15 PE=1 SV=2	98.6	4.64	EPS15
Q8N8N7	Prostaglandin reductase 2 OS=Homo sapiens OX=9606 GN=PTGR2 PE=1 SV=1	38.5	5.41	PTGR2
P35244	Replication protein A 14 kDa subunit OS=Homo sapiens OX=9606 GN=RPA3 PE=1 SV=1	13.6	5.08	RPA3
Q13428	Treacle protein OS=Homo sapiens OX=9606 GN=TCOF1 PE=1 SV=3	152	9.04	TCOF1
P14678	Small nuclear ribonucleoprotein-associated proteins B and B' OS=Homo sapiens OX=9606 GN=SNRPB PE=1 SV=2	24.6	11.19	SNRPB
Q9UPN3	Microtubule-actin cross-linking factor 1, isoforms 1/2/3/5 OS=Homo sapiens OX=9606 GN=MACF1 PE=1 SV=4	837.8	5.39	MACF1
P84243	Histone H3.3 OS=Homo sapiens OX=9606 GN=H3-3A PE=1 SV=2	15.3	11.27	H3-3A; H3-3B
P49589	Cysteine--tRNA ligase, cytoplasmic OS=Homo sapiens OX=9606 GN=CARS1 PE=1 SV=3	85.4	6.76	CARS1
O00139	Kinesin-like protein KIF2A OS=Homo sapiens OX=9606 GN=KIF2A PE=1 SV=3	79.9	6.68	KIF2A
Q9NVH1	DnaJ homolog subfamily C member 11 OS=Homo sapiens OX=9606 GN=DNAJC11 PE=1 SV=2	63.2	8.4	DNAJC11
Q14558	Phosphoribosyl pyrophosphate synthase-associated protein 1 OS=Homo sapiens OX=9606 GN=PRPSAP1 PE=1 SV=2	39.4	7.2	PRPSAP1
Q9BVK6	Transmembrane emp24 domain-containing protein 9 OS=Homo sapiens OX=9606 GN=TMED9 PE=1 SV=2	27.3	8.02	TMED9
Q9Y3Y2	Chromatin target of PRMT1 protein OS=Homo sapiens OX=9606 GN=CHTOP PE=1 SV=2	26.4	12.23	CHTOP
Q9Y6M9	NADH dehydrogenase [ubiquinone] 1 beta subcomplex subunit 9 OS=Homo sapiens OX=9606 GN=NDUFB9 PE=1 SV=3	21.8	8.38	NDUFB9

Q92878	DNA repair protein RAD50 OS=Homo sapiens OX=9606 GN=RAD50 PE=1 SV=1	153.8	6.89	RAD50
P63167	Dynein light chain 1, cytoplasmic OS=Homo sapiens OX=9606 GN=DYNLL1 PE=1 SV=1	10.4	7.4	DYNLL1
P35914	Hydroxymethylglutaryl-CoA lyase, mitochondrial OS=Homo sapiens OX=9606 GN=HMGCL PE=1 SV=2	34.3	8.54	HMGCL
Q96S97	Myeloid-associated differentiation marker OS=Homo sapiens OX=9606 GN=MYADM PE=1 SV=2	35.3	8.15	MYADM
Q8TCJ2	Dolichyl-diphosphooligosaccharide--protein glycosyltransferase subunit STT3B OS=Homo sapiens OX=9606 GN=STT3B PE=1 SV=1	93.6	8.91	STT3B
O75165	DnaJ homolog subfamily C member 13 OS=Homo sapiens OX=9606 GN=DNAJC13 PE=1 SV=5	254.3	6.74	DNAJC13
P09661	U2 small nuclear ribonucleoprotein A' OS=Homo sapiens OX=9606 GN=SNRPA1 PE=1 SV=2	28.4	8.62	SNRPA1
Q9Y3C6	Peptidyl-prolyl cis-trans isomerase-like 1 OS=Homo sapiens OX=9606 GN=PPIL1 PE=1 SV=1	18.2	7.99	PPIL1
Q04206	Transcription factor p65 OS=Homo sapiens OX=9606 GN=RELA PE=1 SV=2	60.2	5.68	RELA
Q71UM5	40S ribosomal protein S27-like OS=Homo sapiens OX=9606 GN=RPS27L PE=1 SV=3	9.5	9.45	RPS27L
Q9UMY4	Sorting nexin-12 OS=Homo sapiens OX=9606 GN=SNX12 PE=1 SV=4	18.9	8.44	SNX12
Q14573	Inositol 1,4,5-trisphosphate receptor type 3 OS=Homo sapiens OX=9606 GN=ITPR3 PE=1 SV=2	303.9	6.48	ITPR3
O75400	Pre-mRNA-processing factor 40 homolog A OS=Homo sapiens OX=9606 GN=PRPF40A PE=1 SV=2	108.7	7.56	PRPF40A
Q9UHY7	Enolase-phosphatase E1 OS=Homo sapiens OX=9606 GN=ENOPH1 PE=1 SV=1	28.9	4.78	ENOPH1
Q9UIJ7	GTP:AMP phosphotransferase AK3, mitochondrial OS=Homo sapiens OX=9606 GN=AK3 PE=1 SV=4	25.6	9.16	AK3
O14907	Tax1-binding protein 3 OS=Homo sapiens OX=9606 GN=TAX1BP3 PE=1 SV=2	13.7	8.48	TAX1BP3

P19404	NADH dehydrogenase [ubiquinone] flavoprotein 2, mitochondrial OS=Homo sapiens OX=9606 GN=NDUFV2 PE=1 SV=2	27.4	8.06	NDUFV2
Q96N67	Dedicator of cytokinesis protein 7 OS=Homo sapiens OX=9606 GN=DOCK7 PE=1 SV=4	242.4	6.8	DOCK7
Q96JJ7	Protein disulfide-isomerase TMX3 OS=Homo sapiens OX=9606 GN=TMX3 PE=1 SV=2	51.8	4.91	TMX3
P52789	Hexokinase-2 OS=Homo sapiens OX=9606 GN=HK2 PE=1 SV=2	102.3	6.05	HK2
Q10713	Mitochondrial-processing peptidase subunit alpha OS=Homo sapiens OX=9606 GN=PMPCA PE=1 SV=2	58.2	6.92	PMPCA
Q16204	Coiled-coil domain-containing protein 6 OS=Homo sapiens OX=9606 GN=CCDC6 PE=1 SV=2	53.3	7.34	CCDC6
P22033	Methylmalonyl-CoA mutase, mitochondrial OS=Homo sapiens OX=9606 GN=MMUT PE=1 SV=4	83.1	6.93	MMUT
Q92572	AP-3 complex subunit sigma-1 OS=Homo sapiens OX=9606 GN=AP3S1 PE=1 SV=1	21.7	5.39	AP3S1
P62857	40S ribosomal protein S28 OS=Homo sapiens OX=9606 GN=RPS28 PE=1 SV=1	7.8	10.7	RPS28
P26440	Isovaleryl-CoA dehydrogenase, mitochondrial OS=Homo sapiens OX=9606 GN=IVD PE=1 SV=2	46.6	8.05	IVD
P40123	Adenylyl cyclase-associated protein 2 OS=Homo sapiens OX=9606 GN=CAP2 PE=1 SV=1	52.8	6.37	CAP2
Q9UN37	Vacuolar protein sorting-associated protein 4A OS=Homo sapiens OX=9606 GN=VPS4A PE=1 SV=1	48.9	7.8	VPS4A
Q9Y4F1	FERM, ARHGEF and pleckstrin domain-containing protein 1 OS=Homo sapiens OX=9606 GN=FARP1 PE=1 SV=1	118.6	8.15	FARP1
Q14C86	GTPase-activating protein and VPS9 domain-containing protein 1 OS=Homo sapiens OX=9606 GN=GAPVD1 PE=1 SV=2	164.9	5.22	GAPVD1
P49406	39S ribosomal protein L19, mitochondrial OS=Homo sapiens OX=9606 GN=MRPL19 PE=1 SV=2	33.5	9.5	MRPL19

O75439	Mitochondrial-processing peptidase subunit beta OS=Homo sapiens OX=9606 GN=PMPCB PE=1 SV=2	54.3	6.83	PMPCB
P28065	Proteasome subunit beta type-9 OS=Homo sapiens OX=9606 GN=PSMB9 PE=1 SV=2	23.3	5.03	PSMB9
Q99459	Cell division cycle 5-like protein OS=Homo sapiens OX=9606 GN=CDC5L PE=1 SV=2	92.2	8.18	CDC5L
P57088	Transmembrane protein 33 OS=Homo sapiens OX=9606 GN=TMEM33 PE=1 SV=2	28	9.7	TMEM33
O43615	Mitochondrial import inner membrane translocase subunit TIM44 OS=Homo sapiens OX=9606 GN=TIMM44 PE=1 SV=2	51.3	8.32	TIMM44
Q9NXR7	BRISC and BRCA1-A complex member 2 OS=Homo sapiens OX=9606 GN=BABAM2 PE=1 SV=2	43.5	5.81	BABAM2
Q99426	Tubulin-folding cofactor B OS=Homo sapiens OX=9606 GN=TBCB PE=1 SV=2	27.3	5.15	TBCB
Q9NX63	MICOS complex subunit MIC19 OS=Homo sapiens OX=9606 GN=CHCHD3 PE=1 SV=1	26.1	8.28	CHCHD3
P16083	Ribosyldihydronicotinamide dehydrogenase [quinone] OS=Homo sapiens OX=9606 GN=NQO2 PE=1 SV=5	25.9	6.29	NQO2
P14927	Cytochrome b-c1 complex subunit 7 OS=Homo sapiens OX=9606 GN=UQCRB PE=1 SV=2	13.5	8.78	UQCRB
P60983	Glia maturation factor beta OS=Homo sapiens OX=9606 GN=GMFB PE=1 SV=2	16.7	5.29	GMFB
Q8WU90	Zinc finger CCCH domain-containing protein 15 OS=Homo sapiens OX=9606 GN=ZC3H15 PE=1 SV=1	48.6	5.31	ZC3H15
P78406	mRNA export factor OS=Homo sapiens OX=9606 GN=RAE1 PE=1 SV=1	40.9	7.83	RAE1
Q9P035	Very-long-chain (3R)-3-hydroxyacyl-CoA dehydratase 3 OS=Homo sapiens OX=9606 GN=HACD3 PE=1 SV=2	43.1	8.94	HACD3
P32321	Deoxycytidylate deaminase OS=Homo sapiens OX=9606 GN=DCTD PE=1 SV=2	20	7.56	DCTD
P00374	Dihydrofolate reductase OS=Homo sapiens OX=9606 GN=DHFR PE=1 SV=2	21.4	7.42	DHFR

Q9NYB9	Abl interactor 2 OS=Homo sapiens OX=9606 GN=ABI2 PE=1 SV=1	55.6	6.16	ABI2
P42226	Signal transducer and activator of transcription 6 OS=Homo sapiens OX=9606 GN=STAT6 PE=1 SV=1	94.1	6.23	STAT6
Q9H0C8	Integrin-linked kinase-associated serine/threonine phosphatase 2C OS=Homo sapiens OX=9606 GN=ILKAP PE=1 SV=1	42.9	7.09	ILKAP
Q14011	Cold-inducible RNA-binding protein OS=Homo sapiens OX=9606 GN=CIRBP PE=1 SV=1	18.6	9.51	CIRBP
P60468	Protein transport protein Sec61 subunit beta OS=Homo sapiens OX=9606 GN=SEC61B PE=1 SV=2	10	11.56	SEC61B
P33316	Deoxyuridine 5'-triphosphate nucleotidohydrolase, mitochondrial OS=Homo sapiens OX=9606 GN=DUT PE=1 SV=4	26.5	9.36	DUT
P49959	Double-strand break repair protein MRE11 OS=Homo sapiens OX=9606 GN=MRE11 PE=1 SV=3	80.5	5.9	MRE11
P20338	Ras-related protein Rab-4A OS=Homo sapiens OX=9606 GN=RAB4A PE=1 SV=3	24.4	6.07	RAB4A
P09038	Fibroblast growth factor 2 OS=Homo sapiens OX=9606 GN=FGF2 PE=1 SV=3	30.8	11.18	FGF2
Q92747	Actin-related protein 2/3 complex subunit 1A OS=Homo sapiens OX=9606 GN=ARPC1A PE=1 SV=2	41.5	8.18	ARPC1A
O95168	NADH dehydrogenase [ubiquinone] 1 beta subcomplex subunit 4 OS=Homo sapiens OX=9606 GN=NDUFB4 PE=1 SV=3	15.2	9.85	NDUFB4
P48147	Prolyl endopeptidase OS=Homo sapiens OX=9606 GN=PREP PE=1 SV=2	80.6	5.86	PREP
Q6P587	Acylpyruvase FAHD1, mitochondrial OS=Homo sapiens OX=9606 GN=FAHD1 PE=1 SV=2	24.8	7.39	FAHD1
Q9H3K2	Growth hormone-inducible transmembrane protein OS=Homo sapiens OX=9606 GN=GHITM PE=1 SV=2	37.2	9.94	GHITM
P62306	Small nuclear ribonucleoprotein F OS=Homo sapiens OX=9606 GN=SNRPF PE=1 SV=1	9.7	4.67	SNRPF

Q9H9J2	39S ribosomal protein L44, mitochondrial OS=Homo sapiens OX=9606 GN=MRPL44 PE=1 SV=1	37.5	8.4	MRPL44
P43897	Elongation factor Ts, mitochondrial OS=Homo sapiens OX=9606 GN=TSFM PE=1 SV=2	35.4	8.38	TSFM
Q14126	Desmoglein-2 OS=Homo sapiens OX=9606 GN=DSG2 PE=1 SV=2	122.2	5.24	DSG2
Q8NBJ7	Inactive C-alpha-formylglycine-generating enzyme 2 OS=Homo sapiens OX=9606 GN=SUMF2 PE=1 SV=2	33.8	8	SUMF2
Q9Y6W5	Wiskott-Aldrich syndrome protein family member 2 OS=Homo sapiens OX=9606 GN=WASF2 PE=1 SV=3	54.3	5.53	WASF2
Q14790	Caspase-8 OS=Homo sapiens OX=9606 GN=CASP8 PE=1 SV=1	55.4	5.1	CASP8
Q01469	Fatty acid-binding protein 5 OS=Homo sapiens OX=9606 GN=FABP5 PE=1 SV=3	15.2	7.01	FABP5
O94776	Metastasis-associated protein MTA2 OS=Homo sapiens OX=9606 GN=MTA2 PE=1 SV=1	75	9.66	MTA2
Q5JTZ9	Alanine--tRNA ligase, mitochondrial OS=Homo sapiens OX=9606 GN=AARS2 PE=1 SV=1	107.3	6.27	AARS2
Q07157	Tight junction protein ZO-1 OS=Homo sapiens OX=9606 GN=TJP1 PE=1 SV=3	195.3	6.7	TJP1
P52434	DNA-directed RNA polymerases I, II, and III subunit RPABC3 OS=Homo sapiens OX=9606 GN=POLR2H PE=1 SV=4	17.1	4.68	POLR2H
Q5XKP0	MICOS complex subunit MIC13 OS=Homo sapiens OX=9606 GN=MICOS13 PE=1 SV=1	13.1	9.42	MICOS13
Q8WVY7	Ubiquitin-like domain-containing CTD phosphatase 1 OS=Homo sapiens OX=9606 GN=UBLCP1 PE=1 SV=2	36.8	6.46	UBLCP1
Q96IZ0	PRKC apoptosis WT1 regulator protein OS=Homo sapiens OX=9606 GN=PAWR PE=1 SV=1	36.5	5.41	PAWR
P55854	Small ubiquitin-related modifier 3 OS=Homo sapiens OX=9606 GN=SUMO3 PE=1 SV=2	11.6	5.49	SUMO3
Q15021	Condensin complex subunit 1 OS=Homo sapiens OX=9606 GN=NCAPD2 PE=1 SV=3	157.1	6.61	NCAPD2

O43765	Small glutamine-rich tetratricopeptide repeat-containing protein alpha OS=Homo sapiens OX=9606 GN=SGTA PE=1 SV=1	34	4.87	SGTA
P08579	U2 small nuclear ribonucleoprotein B'' OS=Homo sapiens OX=9606 GN=SNRPB2 PE=1 SV=1	25.5	9.72	SNRPB2
P49757	Protein numb homolog OS=Homo sapiens OX=9606 GN=NUMB PE=1 SV=2	70.8	8.51	NUMB
P0DMN0	Sulfotransferase 1A4 OS=Homo sapiens OX=9606 GN=SULT1A4 PE=1 SV=1	34.2	6.01	SULT1A4
P50897	Palmitoyl-protein thioesterase 1 OS=Homo sapiens OX=9606 GN=PPT1 PE=1 SV=1	34.2	6.52	PPT1
P50851	Lipopolysaccharide-responsive and beige-like anchor protein OS=Homo sapiens OX=9606 GN=LRBA PE=1 SV=4	318.9	5.6	LRBA
Q9BRJ2	39S ribosomal protein L45, mitochondrial OS=Homo sapiens OX=9606 GN=MRPL45 PE=1 SV=2	35.3	9.03	MRPL45
P21266	Glutathione S-transferase Mu 3 OS=Homo sapiens OX=9606 GN=GSTM3 PE=1 SV=3	26.5	5.54	GSTM3
Q969G5	Caveolae-associated protein 3 OS=Homo sapiens OX=9606 GN=CAVIN3 PE=1 SV=3	27.7	6.43	CAVIN3
Q9Y5S9	RNA-binding protein 8A OS=Homo sapiens OX=9606 GN=RBM8A PE=1 SV=1	19.9	5.72	RBM8A
Q08752	Peptidyl-prolyl cis-trans isomerase D OS=Homo sapiens OX=9606 GN=PPID PE=1 SV=3	40.7	7.21	PPID
Q86Y82	Syntaxin-12 OS=Homo sapiens OX=9606 GN=STX12 PE=1 SV=1	31.6	5.59	STX12
Q9Y314	Nitric oxide synthase-interacting protein OS=Homo sapiens OX=9606 GN=NOSIP PE=1 SV=1	33.2	8.82	NOSIP
Q8NBF2	NHL repeat-containing protein 2 OS=Homo sapiens OX=9606 GN=NHLRC2 PE=1 SV=1	79.4	5.55	NHLRC2
Q8TB61	Adenosine 3'-phospho 5'-phosphosulfate transporter 1 OS=Homo sapiens OX=9606 GN=SLC35B2 PE=1 SV=1	47.5	9.16	SLC35B2
P30622	CAP-Gly domain-containing linker protein 1 OS=Homo sapiens OX=9606 GN=CLIP1 PE=1 SV=2	162.1	5.36	CLIP1

Q12959	Disks large homolog 1 OS=Homo sapiens OX=9606 GN=DLG1 PE=1 SV=2	100.4	5.76	DLG1
Q9GZT8	NIF3-like protein 1 OS=Homo sapiens OX=9606 GN=NIF3L1 PE=1 SV=2	41.9	6.65	NIF3L1
Q96GG9	DCN1-like protein 1 OS=Homo sapiens OX=9606 GN=DCUN1D1 PE=1 SV=1	30.1	5.34	DCUN1D1
O75663	TIP41-like protein OS=Homo sapiens OX=9606 GN=TIPRL PE=1 SV=2	31.4	5.91	TIPRL
O00743	Serine/threonine-protein phosphatase 6 catalytic subunit OS=Homo sapiens OX=9606 GN=PPP6C PE=1 SV=1	35.1	5.69	PPP6C
P19388	DNA-directed RNA polymerases I, II, and III subunit RPABC1 OS=Homo sapiens OX=9606 GN=POLR2E PE=1 SV=4	24.5	5.95	POLR2E
O15511	Actin-related protein 2/3 complex subunit 5 OS=Homo sapiens OX=9606 GN=ARPC5 PE=1 SV=3	16.3	5.67	ARPC5
Q96DG6	Carboxymethylenebutenolidase homolog OS=Homo sapiens OX=9606 GN=CMBL PE=1 SV=1	28	7.18	CMBL
Q9BUH6	Protein PAXX OS=Homo sapiens OX=9606 GN=PAXX PE=1 SV=2	21.6	5.48	PAXX
P46109	Crk-like protein OS=Homo sapiens OX=9606 GN=CRKL PE=1 SV=1	33.8	6.74	CRKL
P36404	ADP-ribosylation factor-like protein 2 OS=Homo sapiens OX=9606 GN=ARL2 PE=1 SV=4	20.9	6.34	ARL2
Q9BRA2	Thioredoxin domain-containing protein 17 OS=Homo sapiens OX=9606 GN=TXNDC17 PE=1 SV=1	13.9	5.52	TXNDC17
Q9UNN5	FAS-associated factor 1 OS=Homo sapiens OX=9606 GN=FAF1 PE=1 SV=2	73.9	4.88	FAF1
P02765	Alpha-2-HS-glycoprotein OS=Homo sapiens OX=9606 GN=AHSG PE=1 SV=2	39.3	5.72	AHSG
Q96GM5	SWI/SNF-related matrix-associated actin-dependent regulator of chromatin subfamily D member 1 OS=Homo sapiens OX=9606 GN=SMARCD1 PE=1 SV=2	58.2	9.25	SMARCD1

P14174	Macrophage migration inhibitory factor OS=Homo sapiens OX=9606 GN=MIF PE=1 SV=4	12.5	7.88	MIF
P53597	Succinate--CoA ligase [ADP/GDP-forming] subunit alpha, mitochondrial OS=Homo sapiens OX=9606 GN=SUCLG1 PE=1 SV=4	36.2	8.79	SUCLG1
Q9Y547	Intraflagellar transport protein 25 homolog OS=Homo sapiens OX=9606 GN=HSPB11 PE=1 SV=1	16.3	5.03	HSPB11
P10619	Lysosomal protective protein OS=Homo sapiens OX=9606 GN=CTSA PE=1 SV=2	54.4	6.61	CTSA
P61803	Dolichyl-diphosphooligosaccharide--protein glycosyltransferase subunit DAD1 OS=Homo sapiens OX=9606 GN=DAD1 PE=1 SV=3	12.5	7.08	DAD1
Q13573	SNW domain-containing protein 1 OS=Homo sapiens OX=9606 GN=SNW1 PE=1 SV=1	61.5	9.52	SNW1
P45877	Peptidyl-prolyl cis-trans isomerase C OS=Homo sapiens OX=9606 GN=PPIC PE=1 SV=1	22.7	8.4	PPIC
Q12849	G-rich sequence factor 1 OS=Homo sapiens OX=9606 GN=GRSF1 PE=1 SV=3	53.1	6.19	GRSF1
P32320	Cytidine deaminase OS=Homo sapiens OX=9606 GN=CDA PE=1 SV=2	16.2	6.92	CDA
O75475	PC4 and SFRS1-interacting protein OS=Homo sapiens OX=9606 GN=PSIP1 PE=1 SV=1	60.1	9.13	PSIP1
O95197	Reticulon-3 OS=Homo sapiens OX=9606 GN=RTN3 PE=1 SV=2	112.5	4.96	RTN3
O00592	Podocalyxin OS=Homo sapiens OX=9606 GN=PODXL PE=1 SV=2	58.6	5.49	PODXL
O14936	Peripheral plasma membrane protein CASK OS=Homo sapiens OX=9606 GN=CASK PE=1 SV=3	105.1	6.43	CASK
P42574	Caspase-3 OS=Homo sapiens OX=9606 GN=CASP3 PE=1 SV=2	31.6	6.54	CASP3
P30533	Alpha-2-macroglobulin receptor-associated protein OS=Homo sapiens OX=9606 GN=LRPAP1 PE=1 SV=1	41.4	8.78	LRPAP1

**P07477**	Trypsin-1 OS=Homo sapiens OX=9606 GN=PRSS1 PE=1 SV=1	26.5	6.51	PRSS1
**O75976**	Carboxypeptidase D OS=Homo sapiens OX=9606 GN=CPD PE=1 SV=2	152.8	6.05	CPD
**Q92544**	Transmembrane 9 superfamily member 4 OS=Homo sapiens OX=9606 GN=TM9SF4 PE=1 SV=2	74.5	6.54	TM9SF4
**Q9H3K6**	BolA-like protein 2 OS=Homo sapiens OX=9606 GN=BOLA2 PE=1 SV=1	10.1	6.52	BOLA2; BOLA2B
**P56385**	ATP synthase subunit e, mitochondrial OS=Homo sapiens OX=9606 GN=ATP5ME PE=1 SV=2	7.9	9.35	ATP5ME
**Q16270**	Insulin-like growth factor-binding protein 7 OS=Homo sapiens OX=9606 GN=IGFBP7 PE=1 SV=1	29.1	7.9	IGFBP7
**P63208**	S-phase kinase-associated protein 1 OS=Homo sapiens OX=9606 GN=SKP1 PE=1 SV=2	18.6	4.54	SKP1
**O96000**	NADH dehydrogenase [ubiquinone] 1 beta subcomplex subunit 10 OS=Homo sapiens OX=9606 GN=NDUFB10 PE=1 SV=3	20.8	8.48	NDUFB10
**A0FGR8**	Extended synaptotagmin-2 OS=Homo sapiens OX=9606 GN=ESYT2 PE=1 SV=1	102.3	9.26	ESYT2
**Q9H0B6**	Kinesin light chain 2 OS=Homo sapiens OX=9606 GN=KLC2 PE=1 SV=1	68.9	7.15	KLC2
**O75608**	Acyl-protein thioesterase 1 OS=Homo sapiens OX=9606 GN=LYPLA1 PE=1 SV=1	24.7	6.77	LYPLA1
**P09758**	Tumor-associated calcium signal transducer 2 OS=Homo sapiens OX=9606 GN=TACSTD2 PE=1 SV=3	35.7	8.87	TACSTD2
**Q5TEC6**	Histone HIST2H3PS2 OS=Homo sapiens OX=9606 GN=H3-2 PE=1 SV=1	15.4	11.27	H3-7
**Q9H4G4**	Golgi-associated plant pathogenesis-related protein 1 OS=Homo sapiens OX=9606 GN=GLIPR2 PE=1 SV=3	17.2	9.41	GLIPR2
**P47813**	Eukaryotic translation initiation factor 1A, X-chromosomal OS=Homo sapiens OX=9606 GN=EIF1AX PE=1 SV=2	16.5	5.24	EIF1AX

Q16775	Hydroxyacylglutathione hydrolase, mitochondrial OS=Homo sapiens OX=9606 GN=HAGH PE=1 SV=2	33.8	8.12	HAGH
Q9Y6A4	Cilia- and flagella-associated protein 20 OS=Homo sapiens OX=9606 GN=CFAP20 PE=1 SV=1	22.8	9.76	CFAP20
Q9H1B7	Probable E3 ubiquitin-protein ligase IRF2BPL OS=Homo sapiens OX=9606 GN=IRF2BPL PE=1 SV=1	82.6	8.24	IRF2BPL
Q9NPJ3	Acyl-coenzyme A thioesterase 13 OS=Homo sapiens OX=9606 GN=ACOT13 PE=1 SV=1	15	9.14	ACOT13
Q13637	Ras-related protein Rab-32 OS=Homo sapiens OX=9606 GN=RAB32 PE=1 SV=3	25	6.54	RAB32
Q5VW32	BRO1 domain-containing protein BROX OS=Homo sapiens OX=9606 GN=BROX PE=1 SV=1	46.4	7.65	BROX
O95302	Peptidyl-prolyl cis-trans isomerase FKBP9 OS=Homo sapiens OX=9606 GN=FKBP9 PE=1 SV=2	63	5.08	FKBP9
Q9BYD2	39S ribosomal protein L9, mitochondrial OS=Homo sapiens OX=9606 GN=MRPL9 PE=1 SV=2	30.2	10.08	MRPL9
P67870	Casein kinase II subunit beta OS=Homo sapiens OX=9606 GN=CSNK2B PE=1 SV=1	24.9	5.55	CSNK2B
Q9BXW7	Haloacid dehalogenase-like hydrolase domain-containing 5 OS=Homo sapiens OX=9606 GN=HDHD5 PE=1 SV=1	46.3	8.13	HDHD5
Q9NUQ8	ATP-binding cassette sub-family F member 3 OS=Homo sapiens OX=9606 GN=ABCF3 PE=1 SV=2	79.7	6.34	ABCF3
Q9UBR2	Cathepsin Z OS=Homo sapiens OX=9606 GN=CTSZ PE=1 SV=1	33.8	7.11	CTSZ
Q9BQA1	Methylosome protein 50 OS=Homo sapiens OX=9606 GN=WDR77 PE=1 SV=1	36.7	5.17	WDR77
Q9Y624	Junctional adhesion molecule A OS=Homo sapiens OX=9606 GN=F11R PE=1 SV=1	32.6	7.9	F11R
Q6PGP7	Tetratricopeptide repeat protein 37 OS=Homo sapiens OX=9606 GN=TTC37 PE=1 SV=1	175.4	7.53	TTC37

Q86Y39	NADH dehydrogenase [ubiquinone] 1 alpha subcomplex subunit 11 OS=Homo sapiens OX=9606 GN=NDUFA11 PE=1 SV=3	14.8	8.72	NDUFA11
Q9P0V9	Septin-10 OS=Homo sapiens OX=9606 GN=SEPTIN10 PE=1 SV=2	52.6	6.8	SEPTIN10
O75688	Protein phosphatase 1B OS=Homo sapiens OX=9606 GN=PPM1B PE=1 SV=1	52.6	5.05	PPM1B
P48509	CD151 antigen OS=Homo sapiens OX=9606 GN=CD151 PE=1 SV=3	28.3	7.47	CD151
O95232	Luc7-like protein 3 OS=Homo sapiens OX=9606 GN=LUC7L3 PE=1 SV=2	51.4	9.79	LUC7L3
Q53H96	Pyrroline-5-carboxylate reductase 3 OS=Homo sapiens OX=9606 GN=PYCR3 PE=1 SV=3	28.6	7.72	PYCR3
Q09161	Nuclear cap-binding protein subunit 1 OS=Homo sapiens OX=9606 GN=NCBP1 PE=1 SV=1	91.8	6.43	NCBP1
O00754	Lysosomal alpha-mannosidase OS=Homo sapiens OX=9606 GN=MAN2B1 PE=1 SV=3	113.7	7.28	MAN2B1
P49770	Translation initiation factor eIF-2B subunit beta OS=Homo sapiens OX=9606 GN=EIF2B2 PE=1 SV=3	39	6.16	EIF2B2
Q13619	Cullin-4A OS=Homo sapiens OX=9606 GN=CUL4A PE=1 SV=3	87.6	8.13	CUL4A
Q9BYD6	39S ribosomal protein L1, mitochondrial OS=Homo sapiens OX=9606 GN=MRPL1 PE=1 SV=2	36.9	8.78	MRPL1
Q15286	Ras-related protein Rab-35 OS=Homo sapiens OX=9606 GN=RAB35 PE=1 SV=1	23	8.29	RAB35
Q9H488	GDP-fucose protein O-fucosyltransferase 1 OS=Homo sapiens OX=9606 GN=POFUT1 PE=1 SV=1	43.9	8.53	POFUT1
O95571	Persulfide dioxygenase ETHE1, mitochondrial OS=Homo sapiens OX=9606 GN=ETHE1 PE=1 SV=2	27.9	6.83	ETHE1
P62312	U6 snRNA-associated Sm-like protein LSm6 OS=Homo sapiens OX=9606 GN=LSM6 PE=1 SV=1	9.1	9.58	LSM6
P47914	60S ribosomal protein L29 OS=Homo sapiens OX=9606 GN=RPL29 PE=1 SV=2	17.7	11.66	RPL29

Q6UW68	Transmembrane protein 205 OS=Homo sapiens OX=9606 GN=TMEM205 PE=1 SV=1	21.2	8.62	TMEM205
Q15006	ER membrane protein complex subunit 2 OS=Homo sapiens OX=9606 GN=EMC2 PE=1 SV=1	34.8	6.57	EMC2
Q9UQ35	Serine/arginine repetitive matrix protein 2 OS=Homo sapiens OX=9606 GN=SRRM2 PE=1 SV=2	299.4	12.06	SRRM2
Q9H6W3	Ribosomal oxygenase 1 OS=Homo sapiens OX=9606 GN=RIOX1 PE=1 SV=2	71	6.46	RIOX1
P56556	NADH dehydrogenase [ubiquinone] 1 alpha subcomplex subunit 6 OS=Homo sapiens OX=9606 GN=NDUFA6 PE=1 SV=4	15.1	9.98	NDUFA6
P53602	Diphosphomevalonate decarboxylase OS=Homo sapiens OX=9606 GN=MVD PE=1 SV=1	43.4	7.23	MVD
P21283	V-type proton ATPase subunit C 1 OS=Homo sapiens OX=9606 GN=ATP6V1C1 PE=1 SV=4	43.9	7.46	ATP6V1C1
Q8IXI1	Mitochondrial Rho GTPase 2 OS=Homo sapiens OX=9606 GN=RHOT2 PE=1 SV=2	68.1	5.86	RHOT2
Q9Y3B4	Splicing factor 3B subunit 6 OS=Homo sapiens OX=9606 GN=SF3B6 PE=1 SV=1	14.6	9.38	SF3B6
Q9P1F3	Costars family protein ABRACL OS=Homo sapiens OX=9606 GN=ABRACL PE=1 SV=1	9.1	6.29	ABRACL
P30519	Heme oxygenase 2 OS=Homo sapiens OX=9606 GN=HMOX2 PE=1 SV=2	36	5.41	HMOX2
Q8IY67	Ribonucleoprotein PTB-binding 1 OS=Homo sapiens OX=9606 GN=RAVER1 PE=1 SV=1	63.8	8.48	RAVER1
O15031	Plexin-B2 OS=Homo sapiens OX=9606 GN=PLXNB2 PE=1 SV=3	205	6.24	PLXNB2
Q13084	39S ribosomal protein L28, mitochondrial OS=Homo sapiens OX=9606 GN=MRPL28 PE=1 SV=4	30.1	8.29	MRPL28
O14732	Inositol monophosphatase 2 OS=Homo sapiens OX=9606 GN=IMPA2 PE=1 SV=1	31.3	6.61	IMPA2

Q9BTT0	Acidic leucine-rich nuclear phosphoprotein 32 family member E OS=Homo sapiens OX=9606 GN=ANP32E PE=1 SV=1	30.7	3.85	ANP32E
Q9Y450	HBS1-like protein OS=Homo sapiens OX=9606 GN=HBS1L PE=1 SV=1	75.4	6.61	HBS1L
P26885	Peptidyl-prolyl cis-trans isomerase FKBP2 OS=Homo sapiens OX=9606 GN=FKBP2 PE=1 SV=2	15.6	9.13	FKBP2
Q14739	Delta(14)-sterol reductase LBR OS=Homo sapiens OX=9606 GN=LBR PE=1 SV=2	70.7	9.36	LBR
P30049	ATP synthase subunit delta, mitochondrial OS=Homo sapiens OX=9606 GN=ATP5F1D PE=1 SV=2	17.5	5.49	ATP5F1D
Q9NTJ5	Phosphatidylinositol-3-phosphatase SAC1 OS=Homo sapiens OX=9606 GN=SACM1L PE=1 SV=2	66.9	7.12	SACM1L
Q9BTZ2	Dehydrogenase/reductase SDR family member 4 OS=Homo sapiens OX=9606 GN=DHRS4 PE=1 SV=3	29.5	8.56	DHRS4
Q8WVV9	Heterogeneous nuclear ribonucleoprotein L-like OS=Homo sapiens OX=9606 GN=HNRNPLL PE=1 SV=1	60	7.72	HNRNPLL
Q92764	Keratin, type I cuticular Ha5 OS=Homo sapiens OX=9606 GN=KRT35 PE=1 SV=5	50.3	4.91	KRT35
P18827	Syndecan-1 OS=Homo sapiens OX=9606 GN=SDC1 PE=1 SV=3	32.4	4.63	SDC1
Q15005	Signal peptidase complex subunit 2 OS=Homo sapiens OX=9606 GN=SPCS2 PE=1 SV=3	25	8.47	SPCS2
Q96KG9	N-terminal kinase-like protein OS=Homo sapiens OX=9606 GN=SCYL1 PE=1 SV=1	89.6	6.3	SCYL1
Q9H008	Phospholysine phosphohistidine inorganic pyrophosphate phosphatase OS=Homo sapiens OX=9606 GN=LHPP PE=1 SV=2	29.1	6.15	LHPP
Q9H479	Fructosamine-3-kinase OS=Homo sapiens OX=9606 GN=FN3K PE=1 SV=1	35.1	7.55	FN3K
P48059	LIM and senescent cell antigen-like-containing domain protein 1 OS=Homo sapiens OX=9606 GN=LIMS1 PE=1 SV=4	37.2	8.05	LIMS1

Q96RQ1	Endoplasmic reticulum-Golgi intermediate compartment protein 2 OS=Homo sapiens OX=9606 GN=ERGIC2 PE=1 SV=2	42.5	6.77	ERGIC2
P16333	Cytoplasmic protein NCK1 OS=Homo sapiens OX=9606 GN=NCK1 PE=1 SV=1	42.8	6.47	NCK1
Q9BW91	ADP-ribose pyrophosphatase, mitochondrial OS=Homo sapiens OX=9606 GN=NUDT9 PE=1 SV=1	39.1	8.22	NUDT9
P01111	GTPase NRas OS=Homo sapiens OX=9606 GN=NRAS PE=1 SV=1	21.2	5.17	NRAS
P29372	DNA-3-methyladenine glycosylase OS=Homo sapiens OX=9606 GN=MPG PE=1 SV=3	32.8	9.57	MPG
Q6NVY1	3-hydroxyisobutyryl-CoA hydrolase, mitochondrial OS=Homo sapiens OX=9606 GN=HIBCH PE=1 SV=2	43.5	8.19	HIBCH
O43291	Kunitz-type protease inhibitor 2 OS=Homo sapiens OX=9606 GN=SPINT2 PE=1 SV=2	28.2	8.29	SPINT2
Q9NZA1	Chloride intracellular channel protein 5 OS=Homo sapiens OX=9606 GN=CLIC5 PE=1 SV=3	46.5	4.82	CLIC5
O14929	Histone acetyltransferase type B catalytic subunit OS=Homo sapiens OX=9606 GN=HAT1 PE=1 SV=1	49.5	5.69	HAT1
O14776	Transcription elongation regulator 1 OS=Homo sapiens OX=9606 GN=TCERG1 PE=1 SV=2	123.8	8.65	TCERG1
P62633	CCHC-type zinc finger nucleic acid binding protein OS=Homo sapiens OX=9606 GN=CNBP PE=1 SV=1	19.5	7.71	CNBP
Q13405	39S ribosomal protein L49, mitochondrial OS=Homo sapiens OX=9606 GN=MRPL49 PE=1 SV=1	19.2	9.45	MRPL49
Q9NPQ8	Synembryn-A OS=Homo sapiens OX=9606 GN=RIC8A PE=1 SV=3	59.7	5.33	RIC8A
Q8N357	Solute carrier family 35 member F6 OS=Homo sapiens OX=9606 GN=SLC35F6 PE=1 SV=1	40.2	6.93	SLC35F6
Q12769	Nuclear pore complex protein Nup160 OS=Homo sapiens OX=9606 GN=NUP160 PE=1 SV=3	162	5.5	NUP160
P49023	Paxillin OS=Homo sapiens OX=9606 GN=PXN PE=1 SV=3	64.5	6.19	PXN

**O00193**	Small acidic protein OS=Homo sapiens OX=9606 GN=SMAP PE=1 SV=1	20.3	4.72	SMAP
**P08590**	Myosin light chain 3 OS=Homo sapiens OX=9606 GN=MYL3 PE=1 SV=3	21.9	5.1	MYL3
**P42285**	Exosome RNA helicase MTR4 OS=Homo sapiens OX=9606 GN=MTREX PE=1 SV=3	117.7	6.52	MTREX
**Q15424**	Scaffold attachment factor B1 OS=Homo sapiens OX=9606 GN=SAFB PE=1 SV=4	102.6	5.47	SAFB
**Q9UKV8**	Protein argonaute-2 OS=Homo sapiens OX=9606 GN=AGO2 PE=1 SV=3	97.1	9.19	AGO2
**Q9H7C9**	Mth938 domain-containing protein OS=Homo sapiens OX=9606 GN=AAMDC PE=1 SV=1	13.3	8.46	AAMDC
**Q99653**	Calcineurin B homologous protein 1 OS=Homo sapiens OX=9606 GN=CHP1 PE=1 SV=3	22.4	5.1	CHP1
**P54105**	Methylosome subunit pICln OS=Homo sapiens OX=9606 GN=CLNS1A PE=1 SV=1	26.2	4.11	CLNS1A
**P60510**	Serine/threonine-protein phosphatase 4 catalytic subunit OS=Homo sapiens OX=9606 GN=PPP4C PE=1 SV=1	35.1	5.06	PPP4C
**Q9BWM7**	Sideroflexin-3 OS=Homo sapiens OX=9606 GN=SFXN3 PE=1 SV=3	35.5	9.1	SFXN3
**P25325**	3-mercaptopyruvate sulfurtransferase OS=Homo sapiens OX=9606 GN=MPST PE=1 SV=3	33.2	6.6	MPST
**P01116**	GTPase KRas OS=Homo sapiens OX=9606 GN=KRAS PE=1 SV=1	21.6	6.77	KRAS
**P51151**	Ras-related protein Rab-9A OS=Homo sapiens OX=9606 GN=RAB9A PE=1 SV=1	22.8	5.47	RAB9A
**Q9UDW1**	Cytochrome b-c1 complex subunit 9 OS=Homo sapiens OX=9606 GN=UQCR10 PE=1 SV=3	7.3	9.47	UQCR10
**Q9Y316**	Protein MEMO1 OS=Homo sapiens OX=9606 GN=MEMO1 PE=1 SV=1	33.7	7.14	MEMO1
**Q86YS6**	Ras-related protein Rab-43 OS=Homo sapiens OX=9606 GN=RAB43 PE=1 SV=1	23.3	5.64	RAB43

Q96IJ6	Mannose-1-phosphate guanyltransferase alpha OS=Homo sapiens OX=9606 GN=GMPPA PE=1 SV=1	46.3	7.21	GMPPA
O95674	Phosphatidate cytidylyltransferase 2 OS=Homo sapiens OX=9606 GN=CDS2 PE=1 SV=1	51.4	7.09	CDS2
Q9Y2L1	Exosome complex exonuclease RRP44 OS=Homo sapiens OX=9606 GN=DIS3 PE=1 SV=2	108.9	7.14	DIS3
Q96FJ2	Dynein light chain 2, cytoplasmic OS=Homo sapiens OX=9606 GN=DYNLL2 PE=1 SV=1	10.3	7.37	DYNLL2
O15212	Prefoldin subunit 6 OS=Homo sapiens OX=9606 GN=PFDN6 PE=1 SV=1	14.6	8.88	PFDN6
A1L0T0	2-hydroxyacyl-CoA lyase 2 OS=Homo sapiens OX=9606 GN=ILVBL PE=1 SV=2	67.8	8.15	ILVBL
P29144	Tripeptidyl-peptidase 2 OS=Homo sapiens OX=9606 GN=TPP2 PE=1 SV=4	138.3	6.32	TPP2
P09497	Clathrin light chain B OS=Homo sapiens OX=9606 GN=CLTB PE=1 SV=1	25.2	4.64	CLTB
Q9H061	Transmembrane protein 126A OS=Homo sapiens OX=9606 GN=TMEM126A PE=1 SV=1	21.5	9.26	TMEM126A
O15357	Phosphatidylinositol 3,4,5-trisphosphate 5-phosphatase 2 OS=Homo sapiens OX=9606 GN=INPPL1 PE=1 SV=2	138.5	6.54	INPPL1
P46736	Lys-63-specific deubiquitinase BRCC36 OS=Homo sapiens OX=9606 GN=BRCC3 PE=1 SV=2	36	5.92	BRCC3
Q9UEY8	Gamma-adducin OS=Homo sapiens OX=9606 GN=ADD3 PE=1 SV=1	79.1	6.32	ADD3
Q13642	Four and a half LIM domains protein 1 OS=Homo sapiens OX=9606 GN=FHL1 PE=1 SV=4	36.2	8.97	FHL1
O00584	Ribonuclease T2 OS=Homo sapiens OX=9606 GN=RNASET2 PE=1 SV=2	29.5	7.08	RNASET2
P55769	NHP2-like protein 1 OS=Homo sapiens OX=9606 GN=SNU13 PE=1 SV=3	14.2	8.46	SNU13
O75781	Paralemmin-1 OS=Homo sapiens OX=9606 GN=PALM PE=1 SV=2	42.1	5	PALM

O43920	NADH dehydrogenase [ubiquinone] iron-sulfur protein 5 OS=Homo sapiens OX=9606 GN=NDUFS5 PE=1 SV=3	12.5	9.14	NDUFS5
Q9Y241	HIG1 domain family member 1A, mitochondrial OS=Homo sapiens OX=9606 GN=HIGD1A PE=1 SV=1	10.1	9.79	HIGD1A
Q92615	La-related protein 4B OS=Homo sapiens OX=9606 GN=LARP4B PE=1 SV=3	80.5	6.92	LARP4B
P48637	Glutathione synthetase OS=Homo sapiens OX=9606 GN=GSS PE=1 SV=1	52.4	5.92	GSS
Q6WCQ1	Myosin phosphatase Rho-interacting protein OS=Homo sapiens OX=9606 GN=MPRIP PE=1 SV=3	116.5	6.21	MPRIP
Q01629	Interferon-induced transmembrane protein 2 OS=Homo sapiens OX=9606 GN=IFITM2 PE=1 SV=2	14.6	7.42	IFITM2
O94760	N(G),N(G)-dimethylarginine dimethylaminohydrolase 1 OS=Homo sapiens OX=9606 GN=DDAH1 PE=1 SV=3	31.1	5.81	DDAH1
Q2TAY7	WD40 repeat-containing protein SMU1 OS=Homo sapiens OX=9606 GN=SMU1 PE=1 SV=2	57.5	7.18	SMU1
P52594	Arf-GAP domain and FG repeat-containing protein 1 OS=Homo sapiens OX=9606 GN=AGFG1 PE=1 SV=2	58.2	8.63	AGFG1
P61916	NPC intracellular cholesterol transporter 2 OS=Homo sapiens OX=9606 GN=NPC2 PE=1 SV=1	16.6	7.65	NPC2
Q9NWH9	SAFB-like transcription modulator OS=Homo sapiens OX=9606 GN=SLTM PE=1 SV=2	117.1	7.87	SLTM
Q9Y3D9	28S ribosomal protein S23, mitochondrial OS=Homo sapiens OX=9606 GN=MRPS23 PE=1 SV=2	21.8	8.9	MRPS23
Q96L92	Sorting nexin-27 OS=Homo sapiens OX=9606 GN=SNX27 PE=1 SV=2	61.2	6.49	SNX27
O60784	Target of Myb protein 1 OS=Homo sapiens OX=9606 GN=TOM1 PE=1 SV=2	53.8	4.7	TOM1
Q9BQ39	ATP-dependent RNA helicase DDX50 OS=Homo sapiens OX=9606 GN=DDX50 PE=1 SV=1	82.5	9.17	DDX50
Q6UXN9	WD repeat-containing protein 82 OS=Homo sapiens OX=9606 GN=WDR82 PE=1 SV=1	35.1	7.69	WDR82

Q9HB90	Ras-related GTP-binding protein C OS=Homo sapiens OX=9606 GN=RRAGC PE=1 SV=1	44.2	5.1	RRAGC
P36543	V-type proton ATPase subunit E 1 OS=Homo sapiens OX=9606 GN=ATP6V1E1 PE=1 SV=1	26.1	8	ATP6V1E1
Q9Y2V2	Calcium-regulated heat-stable protein 1 OS=Homo sapiens OX=9606 GN=CARHSP1 PE=1 SV=2	15.9	8.21	CARHSP1
Q96RS6	NudC domain-containing protein 1 OS=Homo sapiens OX=9606 GN=NUDCD1 PE=1 SV=2	66.7	5.11	NUDCD1
Q15427	Splicing factor 3B subunit 4 OS=Homo sapiens OX=9606 GN=SF3B4 PE=1 SV=1	44.4	8.56	SF3B4
Q6NUM9	All-trans-retinol 13,14-reductase OS=Homo sapiens OX=9606 GN=RETSAT PE=1 SV=2	66.8	8.28	RETSAT
Q8TCS8	Polyribonucleotide nucleotidyltransferase 1, mitochondrial OS=Homo sapiens OX=9606 GN=PNPT1 PE=1 SV=2	85.9	7.77	PNPT1
Q08722	Leukocyte surface antigen CD47 OS=Homo sapiens OX=9606 GN=CD47 PE=1 SV=1	35.2	7.21	CD47
Q86TX2	Acyl-coenzyme A thioesterase 1 OS=Homo sapiens OX=9606 GN=ACOT1 PE=1 SV=1	46.2	7.34	ACOT1
Q92665	28S ribosomal protein S31, mitochondrial OS=Homo sapiens OX=9606 GN=MRPS31 PE=1 SV=3	45.3	9.29	MRPS31
Q9NTI5	Sister chromatid cohesion protein PDS5 homolog B OS=Homo sapiens OX=9606 GN=PDS5B PE=1 SV=1	164.6	8.47	PDS5B
O75884	Serine hydrolase RBBP9 OS=Homo sapiens OX=9606 GN=RBBP9 PE=1 SV=2	21	6.2	RBBP9
Q8N983	39S ribosomal protein L43, mitochondrial OS=Homo sapiens OX=9606 GN=MRPL43 PE=1 SV=1	23.4	8.65	MRPL43
P15289	Arylsulfatase A OS=Homo sapiens OX=9606 GN=ARSA PE=1 SV=3	53.6	6.07	ARSA
Q8IXB1	DnaJ homolog subfamily C member 10 OS=Homo sapiens OX=9606 GN=DNAJC10 PE=1 SV=2	91	7.18	DNAJC10
P62308	Small nuclear ribonucleoprotein G OS=Homo sapiens OX=9606 GN=SNRPG PE=1 SV=1	8.5	8.88	SNRPG

Q7Z4H3	5'-deoxynucleotidase HDDC2 OS=Homo sapiens OX=9606 GN=HDDC2 PE=1 SV=1	23.4	5.49	HDDC2
Q6UB35	Monofunctional C1-tetrahydrofolate synthase, mitochondrial OS=Homo sapiens OX=9606 GN=MTHFD1L PE=1 SV=1	105.7	8.06	MTHFD1L
Q14192	Four and a half LIM domains protein 2 OS=Homo sapiens OX=9606 GN=FHL2 PE=1 SV=3	32.2	7.55	FHL2
P13473	Lysosome-associated membrane glycoprotein 2 OS=Homo sapiens OX=9606 GN=LAMP2 PE=1 SV=2	44.9	5.63	LAMP2
Q5GLZ8	Probable E3 ubiquitin-protein ligase HERC4 OS=Homo sapiens OX=9606 GN=HERC4 PE=1 SV=1	118.5	6.19	HERC4
Q9HD20	Endoplasmic reticulum transmembrane helix translocase OS=Homo sapiens OX=9606 GN=ATP13A1 PE=1 SV=2	132.9	8.13	ATP13A1
Q92530	Proteasome inhibitor PI31 subunit OS=Homo sapiens OX=9606 GN=PSMF1 PE=1 SV=2	29.8	5.74	PSMF1
O00264	Membrane-associated progesterone receptor component 1 OS=Homo sapiens OX=9606 GN=PGRMC1 PE=1 SV=3	21.7	4.7	PGRMC1
Q9UBS4	DnaJ homolog subfamily B member 11 OS=Homo sapiens OX=9606 GN=DNAJB11 PE=1 SV=1	40.5	6.18	DNAJB11
O75494	Serine/arginine-rich splicing factor 10 OS=Homo sapiens OX=9606 GN=SRSF10 PE=1 SV=1	31.3	11.27	SRSF10
P84090	Enhancer of rudimentary homolog OS=Homo sapiens OX=9606 GN=ERH PE=1 SV=1	12.3	5.92	ERH
P54725	UV excision repair protein RAD23 homolog A OS=Homo sapiens OX=9606 GN=RAD23A PE=1 SV=1	39.6	4.58	RAD23A
O14672	Disintegrin and metalloproteinase domain-containing protein 10 OS=Homo sapiens OX=9606 GN=ADAM10 PE=1 SV=1	84.1	7.77	ADAM10
O75251	NADH dehydrogenase [ubiquinone] iron-sulfur protein 7, mitochondrial OS=Homo sapiens OX=9606 GN=NDUFS7 PE=1 SV=3	23.5	9.99	NDUFS7

Q9Y4E1	WASH complex subunit 2C OS=Homo sapiens OX=9606 GN=WASHC2C PE=1 SV=4	144.8	4.79	WASHC2C
Q96K76	Ubiquitin carboxyl-terminal hydrolase 47 OS=Homo sapiens OX=9606 GN=USP47 PE=1 SV=3	157.2	5.08	USP47
O43760	Synaptogyrin-2 OS=Homo sapiens OX=9606 GN=SYNGR2 PE=1 SV=1	24.8	4.94	SYNGR2
Q9NQG5	Regulation of nuclear pre-mRNA domain-containing protein 1B OS=Homo sapiens OX=9606 GN=RPRD1B PE=1 SV=1	36.9	5.97	RPRD1B
Q9UBC2	Epidermal growth factor receptor substrate 15-like 1 OS=Homo sapiens OX=9606 GN=EPS15L1 PE=1 SV=1	94.2	5.11	EPS15L1
Q16740	ATP-dependent Clp protease proteolytic subunit, mitochondrial OS=Homo sapiens OX=9606 GN=CLPP PE=1 SV=1	30.2	8.09	CLPP
Q9NWV4	CXXC motif containing zinc binding protein OS=Homo sapiens OX=9606 GN=CZIB PE=1 SV=1	18	5.01	CZIB
Q99805	Transmembrane 9 superfamily member 2 OS=Homo sapiens OX=9606 GN=TM9SF2 PE=1 SV=1	75.7	7.44	TM9SF2
Q12846	Syntaxin-4 OS=Homo sapiens OX=9606 GN=STX4 PE=1 SV=2	34.2	6.28	STX4
Q9BXB5	Oxysterol-binding protein-related protein 10 OS=Homo sapiens OX=9606 GN=OSBPL10 PE=1 SV=2	83.9	8.31	OSBPL10
P49585	Choline-phosphate cytidylyltransferase A OS=Homo sapiens OX=9606 GN=PCYT1A PE=1 SV=2	41.7	7.25	PCYT1A
P49207	60S ribosomal protein L34 OS=Homo sapiens OX=9606 GN=RPL34 PE=1 SV=3	13.3	11.47	RPL34
Q8WXX5	DnaJ homolog subfamily C member 9 OS=Homo sapiens OX=9606 GN=DNAJC9 PE=1 SV=1	29.9	5.73	DNAJC9
Q15645	Pachytene checkpoint protein 2 homolog OS=Homo sapiens OX=9606 GN=TRIP13 PE=1 SV=2	48.5	6.09	TRIP13
Q9H3H3	UPF0696 protein C11orf68 OS=Homo sapiens OX=9606 GN=C11orf68 PE=1 SV=3	31.4	6.32	C11orf68

Q8WUH6	Transmembrane protein 263 OS=Homo sapiens OX=9606 GN=TMEM263 PE=1 SV=1	11.7	9.32	TMEM263
Q9HD42	Charged multivesicular body protein 1a OS=Homo sapiens OX=9606 GN=CHMP1A PE=1 SV=1	21.7	8.06	CHMP1A
P49458	Signal recognition particle 9 kDa protein OS=Homo sapiens OX=9606 GN=SRP9 PE=1 SV=2	10.1	7.97	SRP9
Q9P0S9	Transmembrane protein 14C OS=Homo sapiens OX=9606 GN=TMEM14C PE=1 SV=1	11.6	9.88	TMEM14C
Q9BW27	Nuclear pore complex protein Nup85 OS=Homo sapiens OX=9606 GN=NUP85 PE=1 SV=1	75	5.55	NUP85
O75116	Rho-associated protein kinase 2 OS=Homo sapiens OX=9606 GN=ROCK2 PE=1 SV=4	160.8	6.02	ROCK2
Q9NXA8	NAD-dependent protein deacylase sirtuin-5, mitochondrial OS=Homo sapiens OX=9606 GN=SIRT5 PE=1 SV=2	33.9	8.47	SIRT5
Q8IY17	Patatin-like phospholipase domain-containing protein 6 OS=Homo sapiens OX=9606 GN=PNPLA6 PE=1 SV=3	150.9	7.74	PNPLA6
O00217	NADH dehydrogenase [ubiquinone] iron-sulfur protein 8, mitochondrial OS=Homo sapiens OX=9606 GN=NDUFS8 PE=1 SV=1	23.7	6.34	NDUFS8
O95816	BAG family molecular chaperone regulator 2 OS=Homo sapiens OX=9606 GN=BAG2 PE=1 SV=1	23.8	6.7	BAG2
P61421	V-type proton ATPase subunit d 1 OS=Homo sapiens OX=9606 GN=ATP6V0D1 PE=1 SV=1	40.3	5	ATP6V0D1
Q92879	CUGBP Elav-like family member 1 OS=Homo sapiens OX=9606 GN=CELF1 PE=1 SV=2	52	8.46	CELF1
Q15651	High mobility group nucleosome-binding domain-containing protein 3 OS=Homo sapiens OX=9606 GN=HMGN3 PE=1 SV=2	10.7	9.66	HMGN3
Q05519	Serine/arginine-rich splicing factor 11 OS=Homo sapiens OX=9606 GN=SRSF11 PE=1 SV=1	53.5	10.52	SRSF11
Q5BKZ1	DBIRD complex subunit ZNF326 OS=Homo sapiens OX=9606 GN=ZNF326 PE=1 SV=2	65.6	5.15	ZNF326

O75607	Nucleoplasmin-3 OS=Homo sapiens OX=9606 GN=NPM3 PE=1 SV=3	19.3	4.63	NPM3
Q99986	Serine/threonine-protein kinase VRK1 OS=Homo sapiens OX=9606 GN=VRK1 PE=1 SV=1	45.4	8.91	VRK1
Q96D71	RalBP1-associated Eps domain-containing protein 1 OS=Homo sapiens OX=9606 GN=REPS1 PE=1 SV=3	86.6	5.69	REPS1
Q99700	Ataxin-2 OS=Homo sapiens OX=9606 GN=ATXN2 PE=1 SV=2	140.2	9.57	ATXN2
P62318	Small nuclear ribonucleoprotein Sm D3 OS=Homo sapiens OX=9606 GN=SNRPD3 PE=1 SV=1	13.9	10.32	SNRPD3
P19022	Cadherin-2 OS=Homo sapiens OX=9606 GN=CDH2 PE=1 SV=4	99.7	4.81	CDH2
O43676	NADH dehydrogenase [ubiquinone] 1 beta subcomplex subunit 3 OS=Homo sapiens OX=9606 GN=NDUFB3 PE=1 SV=3	11.4	9.2	NDUFB3
P00450	Ceruloplasmin OS=Homo sapiens OX=9606 GN=CP PE=1 SV=1	122.1	5.72	CP
Q8NF37	Lysophosphatidylcholine acyltransferase 1 OS=Homo sapiens OX=9606 GN=LPCAT1 PE=1 SV=2	59.1	6.02	LPCAT1
Q9BUR5	MICOS complex subunit MIC26 OS=Homo sapiens OX=9606 GN=APOO PE=1 SV=1	22.3	9.13	APOO
Q12913	Receptor-type tyrosine-protein phosphatase eta OS=Homo sapiens OX=9606 GN=PTPRJ PE=1 SV=3	145.9	5.58	PTPRJ
Q92797	Symplekin OS=Homo sapiens OX=9606 GN=SYMPK PE=1 SV=2	141.1	6.13	SYMPK
Q9Y2S6	Translation machinery-associated protein 7 OS=Homo sapiens OX=9606 GN=TMA7 PE=1 SV=1	7.1	9.99	TMA7
P55735	Protein SEC13 homolog OS=Homo sapiens OX=9606 GN=SEC13 PE=1 SV=3	35.5	5.48	SEC13
P48729	Casein kinase I isoform alpha OS=Homo sapiens OX=9606 GN=CSNK1A1 PE=1 SV=2	38.9	9.57	CSNK1A1
Q9Y5P6	Mannose-1-phosphate guanyltransferase beta OS=Homo sapiens OX=9606 GN=GMPPB PE=1 SV=2	39.8	6.61	GMPPB

Q96DV4	39S ribosomal protein L38, mitochondrial OS=Homo sapiens OX=9606 GN=MRPL38 PE=1 SV=2	44.6	7.53	MRPL38
Q7Z6B7	SLIT-ROBO Rho GTPase-activating protein 1 OS=Homo sapiens OX=9606 GN=SRGAP1 PE=1 SV=1	124.2	6.83	SRGAP1
P55039	Developmentally-regulated GTP-binding protein 2 OS=Homo sapiens OX=9606 GN=DRG2 PE=1 SV=1	40.7	8.88	DRG2
Q9H2V7	Protein spinster homolog 1 OS=Homo sapiens OX=9606 GN=SPNS1 PE=1 SV=1	56.6	6.64	SPNS1
P08962	CD63 antigen OS=Homo sapiens OX=9606 GN=CD63 PE=1 SV=2	25.6	7.81	CD63
P61009	Signal peptidase complex subunit 3 OS=Homo sapiens OX=9606 GN=SPCS3 PE=1 SV=1	20.3	8.62	SPCS3
Q8NFH4	Nucleoporin Nup37 OS=Homo sapiens OX=9606 GN=NUP37 PE=1 SV=1	36.7	5.92	NUP37
P30626	Sorcin OS=Homo sapiens OX=9606 GN=SRI PE=1 SV=1	21.7	5.59	SRI
P48426	Phosphatidylinositol 5-phosphate 4-kinase type-2 alpha OS=Homo sapiens OX=9606 GN=PIP4K2A PE=1 SV=2	46.2	6.99	PIP4K2A
Q3SXM5	Inactive hydroxysteroid dehydrogenase-like protein 1 OS=Homo sapiens OX=9606 GN=HSDL1 PE=1 SV=3	37	8.72	HSDL1
P15151	Poliovirus receptor OS=Homo sapiens OX=9606 GN=PVR PE=1 SV=2	45.3	6.52	PVR
P53985	Monocarboxylate transporter 1 OS=Homo sapiens OX=9606 GN=SLC16A1 PE=1 SV=3	53.9	8.66	SLC16A1
Q9Y6E2	Basic leucine zipper and W2 domain-containing protein 2 OS=Homo sapiens OX=9606 GN=BZW2 PE=1 SV=1	48.1	6.68	BZW2
P08572	Collagen alpha-2(IV) chain OS=Homo sapiens OX=9606 GN=COL4A2 PE=1 SV=4	167.4	8.66	COL4A2
P45954	Short/branched chain specific acyl-CoA dehydrogenase, mitochondrial OS=Homo sapiens OX=9606 GN=ACADSB PE=1 SV=1	47.5	6.99	ACADSB
Q8IXM3	39S ribosomal protein L41, mitochondrial OS=Homo sapiens OX=9606 GN=MRPL41 PE=1 SV=1	15.4	9.57	MRPL41

O15013	Rho guanine nucleotide exchange factor 10 OS=Homo sapiens OX=9606 GN=ARHGEF10 PE=1 SV=4	151.5	5.68	ARHGEF10
O75616	GTPase Era, mitochondrial OS=Homo sapiens OX=9606 GN=ERAL1 PE=1 SV=2	48.3	8.84	ERAL1
Q8N1N4	Keratin, type II cytoskeletal 78 OS=Homo sapiens OX=9606 GN=KRT78 PE=1 SV=2	56.8	6.02	KRT78
Q99627	COP9 signalosome complex subunit 8 OS=Homo sapiens OX=9606 GN=COPS8 PE=1 SV=1	23.2	5.38	COPS8
Q96RU3	Formin-binding protein 1 OS=Homo sapiens OX=9606 GN=FNBP1 PE=1 SV=2	71.3	5.72	FNBP1
Q86V48	Leucine zipper protein 1 OS=Homo sapiens OX=9606 GN=LUZP1 PE=1 SV=2	120.2	8.5	LUZP1
Q8WXH0	Nesprin-2 OS=Homo sapiens OX=9606 GN=SYNE2 PE=1 SV=3	795.9	5.36	SYNE2
P19623	Spermidine synthase OS=Homo sapiens OX=9606 GN=SRM PE=1 SV=1	33.8	5.49	SRM
Q92575	UBX domain-containing protein 4 OS=Homo sapiens OX=9606 GN=UBXN4 PE=1 SV=2	56.7	6.38	UBXN4
Q02252	Methylmalonate-semialdehyde dehydrogenase [acylating], mitochondrial OS=Homo sapiens OX=9606 GN=ALDH6A1 PE=1 SV=2	57.8	8.5	ALDH6A1
Q14671	Pumilio homolog 1 OS=Homo sapiens OX=9606 GN=PUM1 PE=1 SV=3	126.4	6.84	PUM1
O00330	Pyruvate dehydrogenase protein X component, mitochondrial OS=Homo sapiens OX=9606 GN=PDHX PE=1 SV=3	54.1	8.66	PDHX
Q96GC9	Vacuole membrane protein 1 OS=Homo sapiens OX=9606 GN=VMP1 PE=1 SV=1	46.2	6.95	VMP1
O15533	Tapasin OS=Homo sapiens OX=9606 GN=TAPBP PE=1 SV=1	47.6	7.15	TAPBP
Q14696	LRP chaperone MESD OS=Homo sapiens OX=9606 GN=MESD PE=1 SV=2	26.1	7.78	MESD

P07311	Acylphosphatase-1 OS=Homo sapiens OX=9606 GN=ACYP1 PE=1 SV=2	11.3	9.31	ACYP1
P48507	Glutamate--cysteine ligase regulatory subunit OS=Homo sapiens OX=9606 GN=GCLM PE=1 SV=1	30.7	6.02	GCLM
Q9Y3Z3	Deoxynucleoside triphosphate triphosphohydrolase SAMHD1 OS=Homo sapiens OX=9606 GN=SAMHD1 PE=1 SV=2	72.2	7.14	SAMHD1
Q8TD19	Serine/threonine-protein kinase Nek9 OS=Homo sapiens OX=9606 GN=NEK9 PE=1 SV=2	107.1	5.74	NEK9
Q9UPQ0	LIM and calponin homology domains-containing protein 1 OS=Homo sapiens OX=9606 GN=LIMCH1 PE=1 SV=4	121.8	6.47	LIMCH1
P10586	Receptor-type tyrosine-protein phosphatase F OS=Homo sapiens OX=9606 GN=PTPRF PE=1 SV=2	212.7	6.3	PTPRF
P26358	DNA (cytosine-5)-methyltransferase 1 OS=Homo sapiens OX=9606 GN=DNMT1 PE=1 SV=2	183.1	7.75	DNMT1
Q5T6F2	Ubiquitin-associated protein 2 OS=Homo sapiens OX=9606 GN=UBAP2 PE=1 SV=1	117	7.34	UBAP2
Q96I25	Splicing factor 45 OS=Homo sapiens OX=9606 GN=RBM17 PE=1 SV=1	44.9	5.97	RBM17
Q8NFQ8	Torsin-1A-interacting protein 2 OS=Homo sapiens OX=9606 GN=TOR1AIP2 PE=1 SV=1	51.2	4.96	TOR1AIP2
Q9UEE9	Craniofacial development protein 1 OS=Homo sapiens OX=9606 GN=CFDP1 PE=1 SV=1	33.6	4.81	CFDP1
Q3LXA3	Triokinase/FMN cyclase OS=Homo sapiens OX=9606 GN=TKFC PE=1 SV=2	58.9	7.49	TKFC
Q9UI12	V-type proton ATPase subunit H OS=Homo sapiens OX=9606 GN=ATP6V1H PE=1 SV=1	55.8	6.48	ATP6V1H
Q16850	Lanosterol 14-alpha demethylase OS=Homo sapiens OX=9606 GN=CYP51A1 PE=1 SV=4	57.2	8.53	CYP51A1
P23786	Carnitine O-palmitoyltransferase 2, mitochondrial OS=Homo sapiens OX=9606 GN=CPT2 PE=1 SV=2	73.7	8.18	CPT2
P62304	Small nuclear ribonucleoprotein E OS=Homo sapiens OX=9606 GN=SNRPE PE=1 SV=1	10.8	9.44	SNRPE

Q9Y3B8	Oligoribonuclease, mitochondrial OS=Homo sapiens OX=9606 GN=REXO2 PE=1 SV=3	26.8	6.87	REXO2
Q15126	Phosphomevalonate kinase OS=Homo sapiens OX=9606 GN=PMVK PE=1 SV=3	22	5.73	PMVK
Q8TC07	TBC1 domain family member 15 OS=Homo sapiens OX=9606 GN=TBC1D15 PE=1 SV=2	79.4	5.67	TBC1D15
Q9Y2S7	Polymerase delta-interacting protein 2 OS=Homo sapiens OX=9606 GN=POLDIP2 PE=1 SV=1	42	8.63	POLDIP2
O15162	Phospholipid scramblase 1 OS=Homo sapiens OX=9606 GN=PLSCR1 PE=1 SV=1	35	4.94	PLSCR1
P02647	Apolipoprotein A-I OS=Homo sapiens OX=9606 GN=APOA1 PE=1 SV=1	30.8	5.76	APOA1
P37268	Squalene synthase OS=Homo sapiens OX=9606 GN=FDFT1 PE=1 SV=1	48.1	6.54	FDFT1
O43815	Striatin OS=Homo sapiens OX=9606 GN=STRN PE=1 SV=4	86.1	5.27	STRN
Q53FT3	Protein Hikeshi OS=Homo sapiens OX=9606 GN=HIKESHI PE=1 SV=2	21.6	5.45	HIKESHI
Q7Z4H8	Protein O-glucosyltransferase 3 OS=Homo sapiens OX=9606 GN=POGLUT3 PE=1 SV=2	58.5	8.24	POGLUT3
P61964	WD repeat-containing protein 5 OS=Homo sapiens OX=9606 GN=WDR5 PE=1 SV=1	36.6	8.27	WDR5
P62899	60S ribosomal protein L31 OS=Homo sapiens OX=9606 GN=RPL31 PE=1 SV=1	14.5	10.54	RPL31
Q9Y3A3	MOB-like protein phocein OS=Homo sapiens OX=9606 GN=MOB4 PE=1 SV=1	26	5.78	MOB4
Q9H3Z4	DnaJ homolog subfamily C member 5 OS=Homo sapiens OX=9606 GN=DNAJC5 PE=1 SV=1	22.1	5.07	DNAJC5
O75832	26S proteasome non-ATPase regulatory subunit 10 OS=Homo sapiens OX=9606 GN=PSMD10 PE=1 SV=1	24.4	6.1	PSMD10
Q9BRX8	Peroxiredoxin-like 2A OS=Homo sapiens OX=9606 GN=PRXL2A PE=1 SV=3	25.7	8.84	PRXL2A

Q93050	V-type proton ATPase 116 kDa subunit a1 OS=Homo sapiens OX=9606 GN=ATP6V0A1 PE=1 SV=3	96.4	6.43	ATP6V0A1
P02787	Serotransferrin OS=Homo sapiens OX=9606 GN=TF PE=1 SV=3	77	7.12	TF
Q8N0X7	Spartin OS=Homo sapiens OX=9606 GN=SPART PE=1 SV=1	72.8	5.91	SPART
P60059	Protein transport protein Sec61 subunit gamma OS=Homo sapiens OX=9606 GN=SEC61G PE=1 SV=1	7.7	9.99	SEC61G
Q9GZQ8	Microtubule-associated proteins 1A/1B light chain 3B OS=Homo sapiens OX=9606 GN=MAP1LC3B PE=1 SV=3	14.7	8.94	MAP1LC3B
O95163	Elongator complex protein 1 OS=Homo sapiens OX=9606 GN=ELP1 PE=1 SV=3	150.2	5.94	ELP1
Q7Z3J2	VPS35 endosomal protein-sorting factor-like OS=Homo sapiens OX=9606 GN=VPS35L PE=1 SV=2	109.5	7.21	VPS35L
P57764	Gasdermin-D OS=Homo sapiens OX=9606 GN=GSDMD PE=1 SV=1	52.8	5.08	GSDMD
Q63HN8	E3 ubiquitin-protein ligase RNF213 OS=Homo sapiens OX=9606 GN=RNF213 PE=1 SV=3	591	6.48	RNF213
Q9Y3E5	Peptidyl-tRNA hydrolase 2, mitochondrial OS=Homo sapiens OX=9606 GN=PTRH2 PE=1 SV=1	19.2	8.73	PTRH2
Q9BY43	Charged multivesicular body protein 4a OS=Homo sapiens OX=9606 GN=CHMP4A PE=1 SV=3	25.1	4.7	CHMP4A
Q15800	Methylsterol monooxygenase 1 OS=Homo sapiens OX=9606 GN=MSMO1 PE=1 SV=1	35.2	7.23	MSMO1
Q9BV57	1,2-dihydroxy-3-keto-5-methylthiopentene dioxygenase OS=Homo sapiens OX=9606 GN=ADI1 PE=1 SV=1	21.5	5.68	ADI1
P53582	Methionine aminopeptidase 1 OS=Homo sapiens OX=9606 GN=METAP1 PE=1 SV=2	43.2	7.17	METAP1
Q9NUP9	Protein lin-7 homolog C OS=Homo sapiens OX=9606 GN=LIN7C PE=1 SV=1	21.8	8.43	LIN7C
P23434	Glycine cleavage system H protein, mitochondrial OS=Homo sapiens OX=9606 GN=GCSH PE=1 SV=2	18.9	4.88	GCSH

Q8IVM0	Coiled-coil domain-containing protein 50 OS=Homo sapiens OX=9606 GN=CCDC50 PE=1 SV=1	35.8	6.65	CCDC50
P62273	40S ribosomal protein S29 OS=Homo sapiens OX=9606 GN=RPS29 PE=1 SV=2	6.7	10.13	RPS29
Q9GZX9	Twisted gastrulation protein homolog 1 OS=Homo sapiens OX=9606 GN=TWSG1 PE=1 SV=1	25	5.34	TWSG1
O14562	Ubiquitin domain-containing protein UBFD1 OS=Homo sapiens OX=9606 GN=UBFD1 PE=1 SV=2	33.4	5.77	UBFD1
Q9UHQ9	NADH-cytochrome b5 reductase 1 OS=Homo sapiens OX=9606 GN=CYB5R1 PE=1 SV=1	34.1	9.38	CYB5R1
Q86X76	Deaminated glutathione amidase OS=Homo sapiens OX=9606 GN=NIT1 PE=1 SV=2	35.9	7.74	NIT1
Q9NP92	39S ribosomal protein S30, mitochondrial OS=Homo sapiens OX=9606 GN=MRPS30 PE=1 SV=2	50.3	7.97	MRPS30
Q9NV70	Exocyst complex component 1 OS=Homo sapiens OX=9606 GN=EXOC1 PE=1 SV=4	101.9	6.61	EXOC1
Q92888	Rho guanine nucleotide exchange factor 1 OS=Homo sapiens OX=9606 GN=ARHGEF1 PE=1 SV=2	102.4	5.66	ARHGEF1
Q13425	Beta-2-syntrophin OS=Homo sapiens OX=9606 GN=SNTB2 PE=1 SV=1	57.9	8.82	SNTB2
Q86W92	Liprin-beta-1 OS=Homo sapiens OX=9606 GN=PPFIBP1 PE=1 SV=2	114	5.55	PPFIBP1
Q9Y4E8	Ubiquitin carboxyl-terminal hydrolase 15 OS=Homo sapiens OX=9606 GN=USP15 PE=1 SV=3	112.3	5.22	USP15
P19838	Nuclear factor NF-kappa-B p105 subunit OS=Homo sapiens OX=9606 GN=NFKB1 PE=1 SV=2	105.3	5.4	NFKB1
P62328	Thymosin beta-4 OS=Homo sapiens OX=9606 GN=TMSB4X PE=1 SV=2	5.1	5.06	TMSB4X
Q9Y3B3	Transmembrane emp24 domain-containing protein 7 OS=Homo sapiens OX=9606 GN=TMED7 PE=1 SV=2	25.2	6.89	TMED7
Q53H82	Endoribonuclease LACTB2 OS=Homo sapiens OX=9606 GN=LACTB2 PE=1 SV=2	32.8	6.8	LACTB2

Q969Q0	60S ribosomal protein L36a-like OS=Homo sapiens OX=9606 GN=RPL36AL PE=1 SV=3	12.5	10.65	RPL36AL
Q9GZZ1	N-alpha-acetyltransferase 50 OS=Homo sapiens OX=9606 GN=NAA50 PE=1 SV=1	19.4	8.81	NAA50
Q9NPA8	Transcription and mRNA export factor ENY2 OS=Homo sapiens OX=9606 GN=ENY2 PE=1 SV=1	11.5	9.33	ENY2
O75694	Nuclear pore complex protein Nup155 OS=Homo sapiens OX=9606 GN=NUP155 PE=1 SV=1	155.1	6.16	NUP155
P27986	Phosphatidylinositol 3-kinase regulatory subunit alpha OS=Homo sapiens OX=9606 GN=PIK3R1 PE=1 SV=2	83.5	6.16	PIK3R1
Q9NQT5	Exosome complex component RRP40 OS=Homo sapiens OX=9606 GN=EXOSC3 PE=1 SV=3	29.6	8.1	EXOSC3
O43172	U4/U6 small nuclear ribonucleoprotein Prp4 OS=Homo sapiens OX=9606 GN=PRPF4 PE=1 SV=2	58.4	7.42	PRPF4
P13807	Glycogen [starch] synthase, muscle OS=Homo sapiens OX=9606 GN=GYS1 PE=1 SV=2	83.7	6.18	GYS1
P09132	Signal recognition particle 19 kDa protein OS=Homo sapiens OX=9606 GN=SRP19 PE=1 SV=3	16.1	9.85	SRP19
Q9HCN4	GPN-loop GTPase 1 OS=Homo sapiens OX=9606 GN=GPN1 PE=1 SV=1	41.7	4.92	GPN1
O15305	Phosphomannomutase 2 OS=Homo sapiens OX=9606 GN=PMM2 PE=1 SV=1	28.1	6.77	PMM2
Q9H0U3	Magnesium transporter protein 1 OS=Homo sapiens OX=9606 GN=MAGT1 PE=1 SV=1	38	9.63	MAGT1
Q5VZM2	Ras-related GTP-binding protein B OS=Homo sapiens OX=9606 GN=RRAGB PE=1 SV=1	43.2	6.21	RRAGB
Q9NZL4	Hsp70-binding protein 1 OS=Homo sapiens OX=9606 GN=HSPBP1 PE=1 SV=2	39.3	5.21	HSPBP1
Q12768	WASH complex subunit 5 OS=Homo sapiens OX=9606 GN=WASHC5 PE=1 SV=1	134.2	6.98	WASHC5
P30825	High affinity cationic amino acid transporter 1 OS=Homo sapiens OX=9606 GN=SLC7A1 PE=1 SV=1	67.6	5.43	SLC7A1

Q06710	Paired box protein Pax-8 OS=Homo sapiens OX=9606 GN=PAX8 PE=1 SV=2	48.2	7.84	PAX8
Q53H12	Acylglycerol kinase, mitochondrial OS=Homo sapiens OX=9606 GN=AGK PE=1 SV=2	47.1	8.09	AGK
Q7LG56	Ribonucleoside-diphosphate reductase subunit M2 B OS=Homo sapiens OX=9606 GN=RRM2B PE=1 SV=1	40.7	4.97	RRM2B
Q9NYP7	Elongation of very long chain fatty acids protein 5 OS=Homo sapiens OX=9606 GN=ELOVL5 PE=1 SV=1	35.3	9.42	ELOVL5
Q15642	Cdc42-interacting protein 4 OS=Homo sapiens OX=9606 GN=TRIP10 PE=1 SV=3	68.3	5.73	TRIP10
Q15165	Serum paraoxonase/arylesterase 2 OS=Homo sapiens OX=9606 GN=PON2 PE=1 SV=4	39.4	5.6	PON2
Q92783	Signal transducing adapter molecule 1 OS=Homo sapiens OX=9606 GN=STAM PE=1 SV=3	59.1	4.82	STAM
O43570	Carbonic anhydrase 12 OS=Homo sapiens OX=9606 GN=CA12 PE=1 SV=1	39.4	7.23	CA12
P52701	DNA mismatch repair protein Msh6 OS=Homo sapiens OX=9606 GN=MSH6 PE=1 SV=2	152.7	6.9	MSH6
Q9NRP0	Oligosaccharyltransferase complex subunit OSTC OS=Homo sapiens OX=9606 GN=OSTC PE=1 SV=1	16.8	9.13	OSTC
Q92609	TBC1 domain family member 5 OS=Homo sapiens OX=9606 GN=TBC1D5 PE=1 SV=1	88.9	6.54	TBC1D5
Q9Y5Y2	Cytosolic Fe-S cluster assembly factor NUBP2 OS=Homo sapiens OX=9606 GN=NUBP2 PE=1 SV=1	28.8	5.83	NUBP2
Q05048	Cleavage stimulation factor subunit 1 OS=Homo sapiens OX=9606 GN=CSTF1 PE=1 SV=1	48.3	6.58	CSTF1
P62861	40S ribosomal protein S30 OS=Homo sapiens OX=9606 GN=FAU PE=1 SV=1	6.6	12.15	Fau; FAU
P13984	General transcription factor IIF subunit 2 OS=Homo sapiens OX=9606 GN=GTF2F2 PE=1 SV=2	28.4	9.23	GTF2F2
Q9P206	Uncharacterized protein KIAA1522 OS=Homo sapiens OX=9606 GN=KIAA1522 PE=1 SV=2	107	9.73	KIAA1522

O43633	Charged multivesicular body protein 2a OS=Homo sapiens OX=9606 GN=CHMP2A PE=1 SV=1	25.1	5.97	CHMP2A
Q9HD33	39S ribosomal protein L47, mitochondrial OS=Homo sapiens OX=9606 GN=MRPL47 PE=1 SV=2	29.4	10.37	MRPL47
Q16637	Survival motor neuron protein OS=Homo sapiens OX=9606 GN=SMN1 PE=1 SV=1	31.8	6.55	SMN1; SMN2
Q9UPT8	Zinc finger CCCH domain-containing protein 4 OS=Homo sapiens OX=9606 GN=ZC3H4 PE=1 SV=3	140.2	6.27	ZC3H4
Q9UEU0	Vesicle transport through interaction with t-SNAREs homolog 1B OS=Homo sapiens OX=9606 GN=VTI1B PE=1 SV=3	26.7	9.04	VTI1B
P13716	Delta-aminolevulinic acid dehydratase OS=Homo sapiens OX=9606 GN=ALAD PE=1 SV=1	36.3	6.79	ALAD
Q99720	Sigma non-opioid intracellular receptor 1 OS=Homo sapiens OX=9606 GN=SIGMAR1 PE=1 SV=1	25.1	5.96	SIGMAR1
Q6WKZ4	Rab11 family-interacting protein 1 OS=Homo sapiens OX=9606 GN=RAB11FIP1 PE=1 SV=3	137.1	5.43	RAB11FIP1
O60826	Coiled-coil domain-containing protein 22 OS=Homo sapiens OX=9606 GN=CCDC22 PE=1 SV=1	70.7	6.74	CCDC22
Q8N3C0	Activating signal cointegrator 1 complex subunit 3 OS=Homo sapiens OX=9606 GN=ASCC3 PE=1 SV=3	251.3	7.09	ASCC3
P28676	Grancalcin OS=Homo sapiens OX=9606 GN=GCA PE=1 SV=2	24	5.21	GCA
Q53T59	HCLS1-binding protein 3 OS=Homo sapiens OX=9606 GN=HS1BP3 PE=1 SV=1	42.8	5.01	HS1BP3
Q7Z2W9	39S ribosomal protein L21, mitochondrial OS=Homo sapiens OX=9606 GN=MRPL21 PE=1 SV=2	22.8	9.89	MRPL21
O75935	Dynactin subunit 3 OS=Homo sapiens OX=9606 GN=DCTN3 PE=1 SV=1	21.1	5.47	DCTN3
Q9Y282	Endoplasmic reticulum-Golgi intermediate compartment protein 3 OS=Homo sapiens OX=9606 GN=ERGIC3 PE=1 SV=1	43.2	6.06	ERGIC3

P05204	Non-histone chromosomal protein HMG-17 OS=Homo sapiens OX=9606 GN=HMGN2 PE=1 SV=3	9.4	9.99	HMGN2
P52298	Nuclear cap-binding protein subunit 2 OS=Homo sapiens OX=9606 GN=NCBP2 PE=1 SV=1	18	8.21	NCBP2
Q9Y2W2	WW domain-binding protein 11 OS=Homo sapiens OX=9606 GN=WBP11 PE=1 SV=1	70	8.38	WBP11
Q15323	Keratin, type I cuticular Ha1 OS=Homo sapiens OX=9606 GN=KRT31 PE=1 SV=3	47.2	4.88	KRT31
P35573	Glycogen debranching enzyme OS=Homo sapiens OX=9606 GN=AGL PE=1 SV=3	174.7	6.76	AGL
O15042	U2 snRNP-associated SURP motif-containing protein OS=Homo sapiens OX=9606 GN=U2SURP PE=1 SV=2	118.2	8.47	U2SURP
P49711	Transcriptional repressor CTCF OS=Homo sapiens OX=9606 GN=CTCF PE=1 SV=1	82.7	6.96	CTCF
Q9BY32	Inosine triphosphate pyrophosphatase OS=Homo sapiens OX=9606 GN=ITPA PE=1 SV=2	21.4	5.66	ITPA
Q9UHB6	LIM domain and actin-binding protein 1 OS=Homo sapiens OX=9606 GN=LIMA1 PE=1 SV=1	85.2	6.84	LIMA1
O14933	Ubiquitin/ISG15-conjugating enzyme E2 L6 OS=Homo sapiens OX=9606 GN=UBE2L6 PE=1 SV=4	17.8	7.88	UBE2L6
Q8WWI5	Choline transporter-like protein 1 OS=Homo sapiens OX=9606 GN=SLC44A1 PE=1 SV=1	73.3	8.6	SLC44A1
Q9Y4Y9	U6 snRNA-associated Sm-like protein LSm5 OS=Homo sapiens OX=9606 GN=LSM5 PE=1 SV=3	9.9	4.54	LSM5
P52735	Guanine nucleotide exchange factor VAV2 OS=Homo sapiens OX=9606 GN=VAV2 PE=1 SV=2	101.2	7.08	VAV2
Q8N668	COMM domain-containing protein 1 OS=Homo sapiens OX=9606 GN=COMMD1 PE=1 SV=1	21.2	6.2	COMMD1
Q9Y3E7	Charged multivesicular body protein 3 OS=Homo sapiens OX=9606 GN=CHMP3 PE=1 SV=3	25.1	5.12	CHMP3
Q9NZJ7	Mitochondrial carrier homolog 1 OS=Homo sapiens OX=9606 GN=MTCH1 PE=1 SV=1	41.5	9.32	MTCH1

P14902	Indoleamine 2,3-dioxygenase 1 OS=Homo sapiens OX=9606 GN=IDO1 PE=1 SV=1	45.3	7.3	IDO1
Q5TDH0	Protein DDI1 homolog 2 OS=Homo sapiens OX=9606 GN=DDI2 PE=1 SV=1	44.5	5.05	DDI2
P16278	Beta-galactosidase OS=Homo sapiens OX=9606 GN=GLB1 PE=1 SV=2	76	6.57	GLB1
Q9BV86	N-terminal Xaa-Pro-Lys N-methyltransferase 1 OS=Homo sapiens OX=9606 GN=NTMT1 PE=1 SV=3	25.4	5.52	NTMT1
Q96D46	60S ribosomal export protein NMD3 OS=Homo sapiens OX=9606 GN=NMD3 PE=1 SV=1	57.6	7.14	NMD3
Q9H0P0	Cytosolic 5'-nucleotidase 3A OS=Homo sapiens OX=9606 GN=NT5C3A PE=1 SV=3	37.9	7.12	NT5C3A
Q9UNL2	Translocon-associated protein subunit gamma OS=Homo sapiens OX=9606 GN=SSR3 PE=1 SV=1	21.1	9.61	SSR3
Q9Y4P1	Cysteine protease ATG4B OS=Homo sapiens OX=9606 GN=ATG4B PE=1 SV=2	44.3	5.07	ATG4B
O95861	3'(2'),5'-bisphosphate nucleotidase 1 OS=Homo sapiens OX=9606 GN=BPNT1 PE=1 SV=1	33.4	5.69	BPNT1
Q96EK5	KIF-binding protein OS=Homo sapiens OX=9606 GN=KIFBP PE=1 SV=1	71.8	5.49	KIFBP
P15927	Replication protein A 32 kDa subunit OS=Homo sapiens OX=9606 GN=RPA2 PE=1 SV=1	29.2	6.15	RPA2
Q8N6H7	ADP-ribosylation factor GTPase-activating protein 2 OS=Homo sapiens OX=9606 GN=ARFGAP2 PE=1 SV=1	56.7	7.99	ARFGAP2
Q9UBP9	PTB domain-containing engulfment adapter protein 1 OS=Homo sapiens OX=9606 GN=GULP1 PE=1 SV=1	34.5	7.9	GULP1
P07108	Acyl-CoA-binding protein OS=Homo sapiens OX=9606 GN=DBI PE=1 SV=2	10	6.57	DBI
Q9Y6Y0	Influenza virus NS1A-binding protein OS=Homo sapiens OX=9606 GN=IVNS1ABP PE=1 SV=3	71.7	5.53	IVNS1ABP
Q9NR50	Translation initiation factor eIF-2B subunit gamma OS=Homo sapiens OX=9606 GN=EIF2B3 PE=1 SV=1	50.2	6.47	EIF2B3

Q9NSK0	Kinesin light chain 4 OS=Homo sapiens OX=9606 GN=KLC4 PE=1 SV=3	68.6	6.18	KLC4
Q9NPD3	Exosome complex component RRP41 OS=Homo sapiens OX=9606 GN=EXOSC4 PE=1 SV=3	26.4	6.52	EXOSC4
Q3MHD2	Protein LSM12 homolog OS=Homo sapiens OX=9606 GN=LSM12 PE=1 SV=2	21.7	7.74	LSM12
Q4G0F5	Vacuolar protein sorting-associated protein 26B OS=Homo sapiens OX=9606 GN=VPS26B PE=1 SV=2	39.1	7.36	VPS26B
O43264	Centromere/kinetochore protein zw10 homolog OS=Homo sapiens OX=9606 GN=ZW10 PE=1 SV=3	88.8	6.27	ZW10
Q9GZP8	Immortalization up-regulated protein OS=Homo sapiens OX=9606 GN=IMUP PE=1 SV=1	10.9	9.73	IMUP
Q9Y3A5	Ribosome maturation protein SBDS OS=Homo sapiens OX=9606 GN=SBDS PE=1 SV=4	28.7	8.75	SBDS
P07686	Beta-hexosaminidase subunit beta OS=Homo sapiens OX=9606 GN=HEXB PE=1 SV=3	63.1	6.76	HEXB
Q8N5M9	Protein jagunal homolog 1 OS=Homo sapiens OX=9606 GN=JAGN1 PE=1 SV=1	21.1	9.73	JAGN1
Q9NQT8	Kinesin-like protein KIF13B OS=Homo sapiens OX=9606 GN=KIF13B PE=1 SV=2	202.7	5.88	KIF13B
Q96S66	Chloride channel CLIC-like protein 1 OS=Homo sapiens OX=9606 GN=CLCC1 PE=1 SV=1	62	5.55	CLCC1
Q96EY1	DnaJ homolog subfamily A member 3, mitochondrial OS=Homo sapiens OX=9606 GN=DNAJA3 PE=1 SV=2	52.5	9.26	DNAJA3
P05386	60S acidic ribosomal protein P1 OS=Homo sapiens OX=9606 GN=RPLP1 PE=1 SV=1	11.5	4.32	RPLP1
P49006	MARCKS-related protein OS=Homo sapiens OX=9606 GN=MARCKSL1 PE=1 SV=2	19.5	4.67	MARCKSL1
O95819	Mitogen-activated protein kinase kinase kinase kinase 4 OS=Homo sapiens OX=9606 GN=MAP4K4 PE=1 SV=2	142	7.46	MAP4K4
Q9Y653	Adhesion G-protein coupled receptor G1 OS=Homo sapiens OX=9606 GN=ADGRG1 PE=1 SV=2	77.7	8.48	ADGRG1

O15116	U6 snRNA-associated Sm-like protein LSm1 OS=Homo sapiens OX=9606 GN=LSM1 PE=1 SV=1	15.2	5.22	LSM1
Q96FV2	Secernin-2 OS=Homo sapiens OX=9606 GN=SCRN2 PE=1 SV=3	46.6	5.67	SCRN2
O00468	Agrin OS=Homo sapiens OX=9606 GN=AGRN PE=1 SV=6	217.2	6.39	AGRN
O94906	Pre-mRNA-processing factor 6 OS=Homo sapiens OX=9606 GN=PRPF6 PE=1 SV=1	106.9	8.25	PRPF6
P17612	cAMP-dependent protein kinase catalytic subunit alpha OS=Homo sapiens OX=9606 GN=PRKACA PE=1 SV=2	40.6	8.79	PRKACA
P81605	Dermcidin OS=Homo sapiens OX=9606 GN=DCD PE=1 SV=2	11.3	6.54	DCD
O14548	Cytochrome c oxidase subunit 7A-related protein, mitochondrial OS=Homo sapiens OX=9606 GN=COX7A2L PE=1 SV=2	12.6	9.42	COX7A2L
P27105	Stomatin OS=Homo sapiens OX=9606 GN=STOM PE=1 SV=3	31.7	7.88	STOM
Q8IX01	SURP and G-patch domain-containing protein 2 OS=Homo sapiens OX=9606 GN=SUGP2 PE=1 SV=2	120.1	7.28	SUGP2
O95298	NADH dehydrogenase [ubiquinone] 1 subunit C2 OS=Homo sapiens OX=9606 GN=NDUFC2 PE=1 SV=1	14.2	8.98	NDUFC2
Q13217	DnaJ homolog subfamily C member 3 OS=Homo sapiens OX=9606 GN=DNAJC3 PE=1 SV=1	57.5	6.15	DNAJC3
Q9Y2Q5	Ragulator complex protein LAMTOR2 OS=Homo sapiens OX=9606 GN=LAMTOR2 PE=1 SV=1	13.5	5.4	LAMTOR2
Q9UK41	Vacuolar protein sorting-associated protein 28 homolog OS=Homo sapiens OX=9606 GN=VPS28 PE=1 SV=1	25.4	5.54	VPS28
O43353	Receptor-interacting serine/threonine-protein kinase 2 OS=Homo sapiens OX=9606 GN=RIPK2 PE=1 SV=2	61.2	7.09	RIPK2
Q86Y56	Dynein axonemal assembly factor 5 OS=Homo sapiens OX=9606 GN=DNAAF5 PE=1 SV=4	93.5	6.42	DNAAF5
Q9NR28	Diablo homolog, mitochondrial OS=Homo sapiens OX=9606 GN=DIABLO PE=1 SV=1	27.1	5.9	DIABLO

Q8WZA0	Protein LZIC OS=Homo sapiens OX=9606 GN=LZIC PE=1 SV=1	21.5	4.94	LZIC
Q96EY7	Pentatricopeptide repeat domain-containing protein 3, mitochondrial OS=Homo sapiens OX=9606 GN=PTCD3 PE=1 SV=3	78.5	6.42	PTCD3
Q96FZ7	Charged multivesicular body protein 6 OS=Homo sapiens OX=9606 GN=CHMP6 PE=1 SV=3	23.5	5.31	CHMP6
Q9NRF8	CTP synthase 2 OS=Homo sapiens OX=9606 GN=CTPS2 PE=1 SV=1	65.6	6.9	CTPS2
Q9BTE1	Dynactin subunit 5 OS=Homo sapiens OX=9606 GN=DCTN5 PE=1 SV=1	20.1	8.02	DCTN5
O60216	Double-strand-break repair protein rad21 homolog OS=Homo sapiens OX=9606 GN=RAD21 PE=1 SV=2	71.6	4.65	RAD21
Q8IVS2	Malonyl-CoA-acyl carrier protein transacylase, mitochondrial OS=Homo sapiens OX=9606 GN=MCAT PE=1 SV=2	42.9	8.72	MCAT
Q5T6V5	Queuosine salvage protein OS=Homo sapiens OX=9606 GN=C9orf64 PE=1 SV=1	39	5.88	C9orf64
Q6IAA8	Ragulator complex protein LAMTOR1 OS=Homo sapiens OX=9606 GN=LAMTOR1 PE=1 SV=2	17.7	5.15	LAMTOR1
P50750	Cyclin-dependent kinase 9 OS=Homo sapiens OX=9606 GN=CDK9 PE=1 SV=3	42.8	8.79	CDK9
Q9Y2R9	28S ribosomal protein S7, mitochondrial OS=Homo sapiens OX=9606 GN=MRPS7 PE=1 SV=2	28.1	9.99	MRPS7
Q9BS40	Latexin OS=Homo sapiens OX=9606 GN=LXN PE=1 SV=2	25.7	5.78	LXN
Q9UBI6	Guanine nucleotide-binding protein G(I)/G(S)/G(O) subunit gamma-12 OS=Homo sapiens OX=9606 GN=GNG12 PE=1 SV=3	8	8.97	GNG12
Q9P003	Protein cornichon homolog 4 OS=Homo sapiens OX=9606 GN=CNIH4 PE=1 SV=1	16.1	6.65	CNIH4
O00422	Histone deacetylase complex subunit SAP18 OS=Homo sapiens OX=9606 GN=SAP18 PE=1 SV=1	17.6	9.35	SAP18

Q9UL15	BAG family molecular chaperone regulator 5 OS=Homo sapiens OX=9606 GN=BAG5 PE=1 SV=1	51.2	6.05	BAG5
Q5MNZ6	WD repeat domain phosphoinositide-interacting protein 3 OS=Homo sapiens OX=9606 GN=WDR45B PE=1 SV=2	38.1	7.59	WDR45B
O75940	Survival of motor neuron-related-splicing factor 30 OS=Homo sapiens OX=9606 GN=SMNDC1 PE=1 SV=1	26.7	7.24	SMNDC1
P22612	cAMP-dependent protein kinase catalytic subunit gamma OS=Homo sapiens OX=9606 GN=PRKACG PE=1 SV=3	40.4	8.59	PRKACG
Q8WX92	Negative elongation factor B OS=Homo sapiens OX=9606 GN=NELFB PE=1 SV=1	65.7	6.13	NELFB
Q9H0X4	Protein FAM234A OS=Homo sapiens OX=9606 GN=FAM234A PE=1 SV=1	59.6	6.28	FAM234A
Q96DB5	Regulator of microtubule dynamics protein 1 OS=Homo sapiens OX=9606 GN=RMDN1 PE=1 SV=1	35.8	8.5	RMDN1
P78318	Immunoglobulin-binding protein 1 OS=Homo sapiens OX=9606 GN=IGBP1 PE=1 SV=1	39.2	5.38	IGBP1
Q9Y478	5'-AMP-activated protein kinase subunit beta-1 OS=Homo sapiens OX=9606 GN=PRKAB1 PE=1 SV=4	30.4	6.4	PRKAB1
Q9BXR0	Queuine tRNA-ribosyltransferase catalytic subunit 1 OS=Homo sapiens OX=9606 GN=QTRT1 PE=1 SV=3	44	7.23	QTRT1
Q9P2K8	eIF-2-alpha kinase GCN2 OS=Homo sapiens OX=9606 GN=EIF2AK4 PE=1 SV=3	186.8	6.28	EIF2AK4
P51649	Succinate-semialdehyde dehydrogenase, mitochondrial OS=Homo sapiens OX=9606 GN=ALDH5A1 PE=1 SV=2	57.2	8.28	ALDH5A1
O95487	Protein transport protein Sec24B OS=Homo sapiens OX=9606 GN=SEC24B PE=1 SV=2	137.3	6.67	SEC24B
P04004	Vitronectin OS=Homo sapiens OX=9606 GN=VTN PE=1 SV=1	54.3	5.8	VTN
Q8IYI6	Exocyst complex component 8 OS=Homo sapiens OX=9606 GN=EXOC8 PE=1 SV=2	81.7	5.49	EXOC8
Q9H2H8	Peptidyl-prolyl cis-trans isomerase-like 3 OS=Homo sapiens OX=9606 GN=PPIL3 PE=1 SV=1	18.1	6.79	PPIL3

P61077	Ubiquitin-conjugating enzyme E2 D3 OS=Homo sapiens OX=9606 GN=UBE2D3 PE=1 SV=1	16.7	7.8	UBE2D3
Q53GS9	U4/U6.U5 tri-snRNP-associated protein 2 OS=Homo sapiens OX=9606 GN=USP39 PE=1 SV=2	65.3	8.91	USP39
Q9H1K4	Mitochondrial glutamate carrier 2 OS=Homo sapiens OX=9606 GN=SLC25A18 PE=1 SV=1	33.8	9.25	SLC25A18
O00399	Dynactin subunit 6 OS=Homo sapiens OX=9606 GN=DCTN6 PE=1 SV=1	20.7	6.32	DCTN6
P06865	Beta-hexosaminidase subunit alpha OS=Homo sapiens OX=9606 GN=HEXA PE=1 SV=2	60.7	5.16	HEXA
Q8NBX0	Saccharopine dehydrogenase-like oxidoreductase OS=Homo sapiens OX=9606 GN=SCCPDH PE=1 SV=1	47.1	9.14	SCCPDH
O95721	Synaptosomal-associated protein 29 OS=Homo sapiens OX=9606 GN=SNAP29 PE=1 SV=1	29	5.81	SNAP29
Q6IQ22	Ras-related protein Rab-12 OS=Homo sapiens OX=9606 GN=RAB12 PE=1 SV=3	27.2	8.41	RAB12
P29083	General transcription factor IIE subunit 1 OS=Homo sapiens OX=9606 GN=GTF2E1 PE=1 SV=2	49.4	4.82	GTF2E1
Q9BZF1	Oxysterol-binding protein-related protein 8 OS=Homo sapiens OX=9606 GN=OSBPL8 PE=1 SV=3	101.1	6.96	OSBPL8
Q9P0J7	E3 ubiquitin-protein ligase KCMF1 OS=Homo sapiens OX=9606 GN=KCMF1 PE=1 SV=2	41.9	5.66	KCMF1
P07902	Galactose-1-phosphate uridylyltransferase OS=Homo sapiens OX=9606 GN=GALT PE=1 SV=3	43.3	6.99	GALT
Q7Z4W1	L-xylulose reductase OS=Homo sapiens OX=9606 GN=DCXR PE=1 SV=2	25.9	8.1	DCXR
Q9GZU8	PSME3-interacting protein OS=Homo sapiens OX=9606 GN=PSME3IP1 PE=1 SV=1	28.9	5.45	PSME3IP1
Q8TBX8	Phosphatidylinositol 5-phosphate 4-kinase type-2 gamma OS=Homo sapiens OX=9606 GN=PIP4K2C PE=1 SV=3	47.3	6.84	PIP4K2C
Q9HA64	Ketosamine-3-kinase OS=Homo sapiens OX=9606 GN=FN3KRP PE=1 SV=2	34.4	7.33	FN3KRP

Q9UG63	ATP-binding cassette sub-family F member 2 OS=Homo sapiens OX=9606 GN=ABCF2 PE=1 SV=2	71.2	7.37	ABCF2
Q9UBQ5	Eukaryotic translation initiation factor 3 subunit K OS=Homo sapiens OX=9606 GN=EIF3K PE=1 SV=1	25	4.93	EIF3K
Q15067	Peroxisomal acyl-coenzyme A oxidase 1 OS=Homo sapiens OX=9606 GN=ACOX1 PE=1 SV=3	74.4	8.16	ACOX1
P27449	V-type proton ATPase 16 kDa proteolipid subunit OS=Homo sapiens OX=9606 GN=ATP6V0C PE=1 SV=1	15.7	8.44	ATP6V0C
P20839	Inosine-5'-monophosphate dehydrogenase 1 OS=Homo sapiens OX=9606 GN=IMPDH1 PE=1 SV=2	55.4	6.9	IMPDH1
P11182	Lipoamide acyltransferase component of branched-chain alpha-keto acid dehydrogenase complex, mitochondrial OS=Homo sapiens OX=9606 GN=DBT PE=1 SV=3	53.5	8.51	DBT
P16422	Epithelial cell adhesion molecule OS=Homo sapiens OX=9606 GN=EPCAM PE=1 SV=2	34.9	7.46	EPCAM
Q96GA7	Serine dehydratase-like OS=Homo sapiens OX=9606 GN=SDSL PE=1 SV=1	34.7	6.89	SDSL
Q9NVZ3	Adaptin ear-binding coat-associated protein 2 OS=Homo sapiens OX=9606 GN=NECAP2 PE=1 SV=1	28.3	8.38	NECAP2
P21926	CD9 antigen OS=Homo sapiens OX=9606 GN=CD9 PE=1 SV=4	25.4	7.15	CD9
Q9H9T3	Elongator complex protein 3 OS=Homo sapiens OX=9606 GN=ELP3 PE=1 SV=2	62.2	8.88	ELP3
Q86TB9	Protein PAT1 homolog 1 OS=Homo sapiens OX=9606 GN=PATL1 PE=1 SV=2	86.8	6.67	PATL1
P16455	Methylated-DNA--protein-cysteine methyltransferase OS=Homo sapiens OX=9606 GN=MGMT PE=1 SV=1	21.6	8.1	MGMT
Q8IXM2	Chromatin complexes subunit BAP18 OS=Homo sapiens OX=9606 GN=BAP18 PE=1 SV=1	17.9	7.33	BAP18
Q96K17	Transcription factor BTF3 homolog 4 OS=Homo sapiens OX=9606 GN=BTF3L4 PE=1 SV=1	17.3	6.35	BTF3L4
Q9NP61	ADP-ribosylation factor GTPase-activating protein 3 OS=Homo sapiens OX=9606 GN=ARFGAP3 PE=1 SV=1	56.9	7.36	ARFGAP3

O95218	Zinc finger Ran-binding domain-containing protein 2 OS=Homo sapiens OX=9606 GN=ZRANB2 PE=1 SV=2	37.4	10.01	ZRANB2
Q96EY8	Corrinoid adenosyltransferase OS=Homo sapiens OX=9606 GN=MMAB PE=1 SV=1	27.4	8.6	MMAB
Q9NWV8	BRISC and BRCA1-A complex member 1 OS=Homo sapiens OX=9606 GN=BABAM1 PE=1 SV=1	36.5	4.64	BABAM1
Q5ZPR3	CD276 antigen OS=Homo sapiens OX=9606 GN=CD276 PE=1 SV=1	57.2	4.91	CD276
Q7L5D6	Golgi to ER traffic protein 4 homolog OS=Homo sapiens OX=9606 GN=GET4 PE=1 SV=1	36.5	5.41	GET4
Q9Y2D2	UDP-N-acetylglucosamine transporter OS=Homo sapiens OX=9606 GN=SLC35A3 PE=1 SV=1	36	9.16	SLC35A3
Q12974	Protein tyrosine phosphatase type IVA 2 OS=Homo sapiens OX=9606 GN=PTP4A2 PE=1 SV=1	19.1	8.37	PTP4A2
Q9Y371	Endophilin-B1 OS=Homo sapiens OX=9606 GN=SH3GLB1 PE=1 SV=1	40.8	6.04	SH3GLB1
Q9NRF9	DNA polymerase epsilon subunit 3 OS=Homo sapiens OX=9606 GN=POLE3 PE=1 SV=1	16.8	4.74	POLE3
P53801	Pituitary tumor-transforming gene 1 protein-interacting protein OS=Homo sapiens OX=9606 GN=PTTG1IP PE=1 SV=1	20.3	8.79	PTTG1IP
Q15291	Retinoblastoma-binding protein 5 OS=Homo sapiens OX=9606 GN=RBBP5 PE=1 SV=2	59.1	5.1	RBBP5
O43447	Peptidyl-prolyl cis-trans isomerase H OS=Homo sapiens OX=9606 GN=PPIH PE=1 SV=1	19.2	8.07	PPIH
Q96S52	GPI transamidase component PIG-S OS=Homo sapiens OX=9606 GN=PIGS PE=1 SV=3	61.6	6.49	PIGS
P52815	39S ribosomal protein L12, mitochondrial OS=Homo sapiens OX=9606 GN=MRPL12 PE=1 SV=2	21.3	8.87	MRPL12
Q9Y3D6	Mitochondrial fission 1 protein OS=Homo sapiens OX=9606 GN=FIS1 PE=1 SV=2	16.9	8.79	FIS1
Q96FN4	Copine-2 OS=Homo sapiens OX=9606 GN=CPNE2 PE=1 SV=3	61.2	6.07	CPNE2

Q96C01	Protein FAM136A OS=Homo sapiens OX=9606 GN=FAM136A PE=1 SV=1	15.6	7.61	FAM136A
P19174	1-phosphatidylinositol 4,5-bisphosphate phosphodiesterase gamma-1 OS=Homo sapiens OX=9606 GN=PLCG1 PE=1 SV=1	148.4	6.05	PLCG1
P02792	Ferritin light chain OS=Homo sapiens OX=9606 GN=FTL PE=1 SV=2	20	5.78	FTL
Q5T1M5	FK506-binding protein 15 OS=Homo sapiens OX=9606 GN=FKBP15 PE=1 SV=2	133.5	5.2	FKBP15
Q96S19	Methyltransferase-like 26 OS=Homo sapiens OX=9606 GN=METTL26 PE=1 SV=2	22.6	7.83	METTL26
Q99747	Gamma-soluble NSF attachment protein OS=Homo sapiens OX=9606 GN=NAPG PE=1 SV=1	34.7	5.41	NAPG
O60831	PRA1 family protein 2 OS=Homo sapiens OX=9606 GN=PRAF2 PE=1 SV=1	19.2	9.19	PRAF2
Q8NI27	THO complex subunit 2 OS=Homo sapiens OX=9606 GN=THOC2 PE=1 SV=2	182.7	8.44	THOC2
P61165	Transmembrane protein 258 OS=Homo sapiens OX=9606 GN=TMEM258 PE=1 SV=1	9.1	5.83	TMEM258
Q9UH99	SUN domain-containing protein 2 OS=Homo sapiens OX=9606 GN=SUN2 PE=1 SV=3	80.3	6.73	SUN2
Q969S3	Zinc finger protein 622 OS=Homo sapiens OX=9606 GN=ZNF622 PE=1 SV=1	54.2	6.15	ZNF622
Q9BYG3	MKI67 FHA domain-interacting nucleolar phosphoprotein OS=Homo sapiens OX=9606 GN=NIFK PE=1 SV=1	34.2	9.88	NIFK
Q9Y6Y8	SEC23-interacting protein OS=Homo sapiens OX=9606 GN=SEC23IP PE=1 SV=1	111	5.54	SEC23IP
Q96T51	RUN and FYVE domain-containing protein 1 OS=Homo sapiens OX=9606 GN=RUFY1 PE=1 SV=2	79.8	5.74	RUFY1
Q14728	Major facilitator superfamily domain-containing protein 10 OS=Homo sapiens OX=9606 GN=MFSD10 PE=1 SV=1	48.3	9.6	MFSD10

P12931	Proto-oncogene tyrosine-protein kinase Src OS=Homo sapiens OX=9606 GN=SRC PE=1 SV=3	59.8	7.42	SRC
Q9HC35	Echinoderm microtubule-associated protein-like 4 OS=Homo sapiens OX=9606 GN=EML4 PE=1 SV=3	108.8	6.4	EML4
P09234	U1 small nuclear ribonucleoprotein C OS=Homo sapiens OX=9606 GN=SNRPC PE=1 SV=1	17.4	9.67	SNRPC
Q8IZ83	Aldehyde dehydrogenase family 16 member A1 OS=Homo sapiens OX=9606 GN=ALDH16A1 PE=1 SV=2	85.1	6.79	ALDH16A1
P35658	Nuclear pore complex protein Nup214 OS=Homo sapiens OX=9606 GN=NUP214 PE=1 SV=2	213.5	7.47	NUP214
O14773	Tripeptidyl-peptidase 1 OS=Homo sapiens OX=9606 GN=TPP1 PE=1 SV=2	61.2	6.48	TPP1
Q8IYB5	Stromal membrane-associated protein 1 OS=Homo sapiens OX=9606 GN=SMAP1 PE=1 SV=2	50.4	8.75	SMAP1
Q9UFW8	CGG triplet repeat-binding protein 1 OS=Homo sapiens OX=9606 GN=CGGBP1 PE=1 SV=2	18.8	8.95	CGGBP1
Q8NBK3	Formylglycine-generating enzyme OS=Homo sapiens OX=9606 GN=SUMF1 PE=1 SV=3	40.5	6.65	SUMF1
Q00169	Phosphatidylinositol transfer protein alpha isoform OS=Homo sapiens OX=9606 GN=PITPNA PE=1 SV=2	31.8	6.55	PITPNA
O75381	Peroxisomal membrane protein PEX14 OS=Homo sapiens OX=9606 GN=PEX14 PE=1 SV=1	41.2	4.94	PEX14
Q96A65	Exocyst complex component 4 OS=Homo sapiens OX=9606 GN=EXOC4 PE=1 SV=1	110.4	6.49	EXOC4
O75525	KH domain-containing, RNA-binding, signal transduction-associated protein 3 OS=Homo sapiens OX=9606 GN=KHDRBS3 PE=1 SV=1	38.8	7.61	KHDRBS3
P19387	DNA-directed RNA polymerase II subunit RPB3 OS=Homo sapiens OX=9606 GN=POLR2C PE=1 SV=2	31.4	4.92	POLR2C
Q8WZA9	Immunity-related GTPase family Q protein OS=Homo sapiens OX=9606 GN=IRGQ PE=1 SV=1	62.7	4.88	IRGQ
Q5VZF2	Muscleblind-like protein 2 OS=Homo sapiens OX=9606 GN=MBNL2 PE=1 SV=2	40.5	8.38	MBNL2

Q9Y3C8	Ubiquitin-fold modifier-conjugating enzyme 1 OS=Homo sapiens OX=9606 GN=UFC1 PE=1 SV=3	19.4	7.4	UFC1
Q8N5K1	CDGSH iron-sulfur domain-containing protein 2 OS=Homo sapiens OX=9606 GN=CISD2 PE=1 SV=1	15.3	9.61	CISD2
Q8TDX7	Serine/threonine-protein kinase Nek7 OS=Homo sapiens OX=9606 GN=NEK7 PE=1 SV=1	34.5	8.25	NEK7
Q96DE0	U8 snoRNA-decapping enzyme OS=Homo sapiens OX=9606 GN=NUDT16 PE=1 SV=2	21.3	6.89	NUDT16
Q13445	Transmembrane emp24 domain-containing protein 1 OS=Homo sapiens OX=9606 GN=TMED1 PE=1 SV=1	25.2	4.48	TMED1
Q7Z2W4	Zinc finger CCCH-type antiviral protein 1 OS=Homo sapiens OX=9606 GN=ZC3HAV1 PE=1 SV=3	101.4	8.4	ZC3HAV1
Q03001	Dystonin OS=Homo sapiens OX=9606 GN=DST PE=1 SV=4	860.1	5.25	DST
Q13586	Stromal interaction molecule 1 OS=Homo sapiens OX=9606 GN=STIM1 PE=1 SV=3	77.4	6.67	STIM1
P49790	Nuclear pore complex protein Nup153 OS=Homo sapiens OX=9606 GN=NUP153 PE=1 SV=2	153.8	8.73	NUP153
O15027	Protein transport protein Sec16A OS=Homo sapiens OX=9606 GN=SEC16A PE=1 SV=4	251.7	5.8	SEC16A
Q9NQ84	G-protein coupled receptor family C group 5 member C OS=Homo sapiens OX=9606 GN=GPRC5C PE=1 SV=2	48.2	8.43	GPRC5C
O00151	PDZ and LIM domain protein 1 OS=Homo sapiens OX=9606 GN=PDLIM1 PE=1 SV=4	36	7.02	PDLIM1
Q52LW3	Rho GTPase-activating protein 29 OS=Homo sapiens OX=9606 GN=ARHGAP29 PE=1 SV=2	142	6.74	ARHGAP29
Q8NFH3	Nucleoporin Nup43 OS=Homo sapiens OX=9606 GN=NUP43 PE=1 SV=1	42.1	5.63	NUP43
Q9BRP4	Proteasomal ATPase-associated factor 1 OS=Homo sapiens OX=9606 GN=PAAF1 PE=1 SV=2	42.2	6.32	PAAF1
Q9NRX2	39S ribosomal protein L17, mitochondrial OS=Homo sapiens OX=9606 GN=MRPL17 PE=1 SV=1	20	10.11	MRPL17

O43181	NADH dehydrogenase [ubiquinone] iron-sulfur protein 4, mitochondrial OS=Homo sapiens OX=9606 GN=NDUFS4 PE=1 SV=1	20.1	10.3	NDUFS4
Q9BZE4	GTP-binding protein 4 OS=Homo sapiens OX=9606 GN=GTPBP4 PE=1 SV=3	73.9	9.5	GTPBP4
Q8IZL8	Proline-, glutamic acid- and leucine-rich protein 1 OS=Homo sapiens OX=9606 GN=PELP1 PE=1 SV=2	119.6	4.34	PELP1
Q9NVE7	4'-phosphopantetheine phosphatase OS=Homo sapiens OX=9606 GN=PANK4 PE=1 SV=1	85.9	6.28	PANK4
Q9BVC6	Transmembrane protein 109 OS=Homo sapiens OX=9606 GN=TMEM109 PE=1 SV=1	26.2	10.48	TMEM109
O76054	SEC14-like protein 2 OS=Homo sapiens OX=9606 GN=SEC14L2 PE=1 SV=1	46.1	7.84	SEC14L2
Q9H4A3	Serine/threonine-protein kinase WNK1 OS=Homo sapiens OX=9606 GN=WNK1 PE=1 SV=2	250.6	6.34	WNK1
Q8N4C6	Ninein OS=Homo sapiens OX=9606 GN=NIN PE=1 SV=4	243.1	5.03	NIN
Q712K3	Ubiquitin-conjugating enzyme E2 R2 OS=Homo sapiens OX=9606 GN=UBE2R2 PE=1 SV=1	27.1	4.42	UBE2R2
O60341	Lysine-specific histone demethylase 1A OS=Homo sapiens OX=9606 GN=KDM1A PE=1 SV=2	92.8	6.52	KDM1A
Q6PI48	Aspartate--tRNA ligase, mitochondrial OS=Homo sapiens OX=9606 GN=DARS2 PE=1 SV=1	73.5	8.02	DARS2
P11117	Lysosomal acid phosphatase OS=Homo sapiens OX=9606 GN=ACP2 PE=1 SV=3	48.3	6.74	ACP2
P56378	ATP synthase subunit ATP5MJ, mitochondrial OS=Homo sapiens OX=9606 GN=ATP5MJ PE=1 SV=1	6.7	10.08	ATP5MJ
Q96CS3	FAS-associated factor 2 OS=Homo sapiens OX=9606 GN=FAF2 PE=1 SV=2	52.6	5.62	FAF2
P14854	Cytochrome c oxidase subunit 6B1 OS=Homo sapiens OX=9606 GN=COX6B1 PE=1 SV=2	10.2	7.05	COX6B1
O00161	Synaptosomal-associated protein 23 OS=Homo sapiens OX=9606 GN=SNAP23 PE=1 SV=1	23.3	5.01	SNAP23

Q92506	(3R)-3-hydroxyacyl-CoA dehydrogenase OS=Homo sapiens OX=9606 GN=HSD17B8 PE=1 SV=2	27	6.54	HSD17B8
P53384	Cytosolic Fe-S cluster assembly factor NUBP1 OS=Homo sapiens OX=9606 GN=NUBP1 PE=1 SV=2	34.5	5.33	NUBP1
Q9H0A0	RNA cytidine acetyltransferase OS=Homo sapiens OX=9606 GN=NAT10 PE=1 SV=2	115.7	8.27	NAT10
O75817	Ribonuclease P protein subunit p20 OS=Homo sapiens OX=9606 GN=POP7 PE=1 SV=2	15.6	8.94	POP7
Q96CN7	Isochorismatase domain-containing protein 1 OS=Homo sapiens OX=9606 GN=ISOC1 PE=1 SV=3	32.2	7.39	ISOC1
O14735	CDP-diacylglycerol--inositol 3-phosphatidyltransferase OS=Homo sapiens OX=9606 GN=CDIPT PE=1 SV=1	23.5	8.03	CDIPT
Q969E2	Secretory carrier-associated membrane protein 4 OS=Homo sapiens OX=9606 GN=SCAMP4 PE=1 SV=1	25.7	8.82	SCAMP4
Q9UI10	Translation initiation factor eIF-2B subunit delta OS=Homo sapiens OX=9606 GN=EIF2B4 PE=1 SV=2	57.5	9.38	EIF2B4
Q10471	Polypeptide N-acetylgalactosaminyltransferase 2 OS=Homo sapiens OX=9606 GN=GALNT2 PE=1 SV=1	64.7	8.35	GALNT2
Q96KB5	Lymphokine-activated killer T-cell-originated protein kinase OS=Homo sapiens OX=9606 GN=PBK PE=1 SV=3	36.1	5.12	PBK
O43670	BUB3-interacting and GLEBS motif-containing protein ZNF207 OS=Homo sapiens OX=9606 GN=ZNF207 PE=1 SV=1	50.7	9.1	ZNF207
Q9P0I2	ER membrane protein complex subunit 3 OS=Homo sapiens OX=9606 GN=EMC3 PE=1 SV=3	29.9	6.81	EMC3
E9PRG8	Uncharacterized protein C11orf98 OS=Homo sapiens OX=9606 GN=C11orf98 PE=4 SV=2	14.2	11.53	C11orf98
O43854	EGF-like repeat and discoidin I-like domain-containing protein 3 OS=Homo sapiens OX=9606 GN=EDIL3 PE=1 SV=1	53.7	7.28	EDIL3
P56211	cAMP-regulated phosphoprotein 19 OS=Homo sapiens OX=9606 GN=ARPP19 PE=1 SV=2	12.3	9.09	ARPP19

Q16537	Serine/threonine-protein phosphatase 2A 56 kDa regulatory subunit epsilon isoform OS=Homo sapiens OX=9606 GN=PPP2R5E PE=1 SV=1	54.7	6.95	PPP2R5E
P82921	28S ribosomal protein S21, mitochondrial OS=Homo sapiens OX=9606 GN=MRPS21 PE=1 SV=3	10.7	9.92	MRPS21
Q9H3M7	Thioredoxin-interacting protein OS=Homo sapiens OX=9606 GN=TXNIP PE=1 SV=1	43.6	7.5	TXNIP
Q14789	Golgin subfamily B member 1 OS=Homo sapiens OX=9606 GN=GOLGB1 PE=1 SV=2	375.8	5	GOLGB1
Q99470	Stromal cell-derived factor 2 OS=Homo sapiens OX=9606 GN=SDF2 PE=1 SV=2	23	7.33	SDF2
Q92692	Nectin-2 OS=Homo sapiens OX=9606 GN=NECTIN2 PE=1 SV=1	57.7	4.82	NECTIN2
P62891	60S ribosomal protein L39 OS=Homo sapiens OX=9606 GN=RPL39 PE=1 SV=2	6.4	12.56	RPL39
Q9H3P2	Negative elongation factor A OS=Homo sapiens OX=9606 GN=NELFA PE=1 SV=3	57.2	9.03	NELFA
Q15813	Tubulin-specific chaperone E OS=Homo sapiens OX=9606 GN=TBCE PE=1 SV=1	59.3	6.76	TBCE
Q5VSL9	Striatin-interacting protein 1 OS=Homo sapiens OX=9606 GN=STRIP1 PE=1 SV=1	95.5	6.29	STRIP1
Q9HBM1	Kinetochore protein Spc25 OS=Homo sapiens OX=9606 GN=SPC25 PE=1 SV=1	26.1	8	SPC25
Q8N5M4	Tetratricopeptide repeat protein 9C OS=Homo sapiens OX=9606 GN=TTC9C PE=1 SV=1	20	8.92	TTC9C
Q92538	Golgi-specific brefeldin A-resistance guanine nucleotide exchange factor 1 OS=Homo sapiens OX=9606 GN=GBF1 PE=1 SV=2	206.3	5.73	GBF1
O95825	Quinone oxidoreductase-like protein 1 OS=Homo sapiens OX=9606 GN=CRYZL1 PE=1 SV=2	38.7	5.78	CRYZL1
Q9BSJ2	Gamma-tubulin complex component 2 OS=Homo sapiens OX=9606 GN=TUBGCP2 PE=1 SV=2	102.5	6.84	TUBGCP2

Q9UBW8	COP9 signalosome complex subunit 7a OS=Homo sapiens OX=9606 GN=COPS7A PE=1 SV=1	30.3	8.22	COPS7A
Q9NVH0	Exonuclease 3'-5' domain-containing protein 2 OS=Homo sapiens OX=9606 GN=EXD2 PE=1 SV=2	70.3	8.32	EXD2
Q9UBG0	C-type mannose receptor 2 OS=Homo sapiens OX=9606 GN=MRC2 PE=1 SV=2	166.6	5.83	MRC2
O60832	H/ACA ribonucleoprotein complex subunit DKC1 OS=Homo sapiens OX=9606 GN=DKC1 PE=1 SV=3	57.6	9.42	DKC1
P51692	Signal transducer and activator of transcription 5B OS=Homo sapiens OX=9606 GN=STAT5B PE=1 SV=2	89.8	6.05	STAT5B
Q08431	Lactadherin OS=Homo sapiens OX=9606 GN=MFGE8 PE=1 SV=3	43.1	8.15	MFGE8
Q8TBQ9	Protein kish-A OS=Homo sapiens OX=9606 GN=TMEM167A PE=1 SV=1	8.1	8.95	TMEM167A
Q9UIQ6	Leucyl-cystinyl aminopeptidase OS=Homo sapiens OX=9606 GN=LNPEP PE=1 SV=3	117.3	5.73	LNPEP
Q99442	Translocation protein SEC62 OS=Homo sapiens OX=9606 GN=SEC62 PE=1 SV=1	45.8	7.12	SEC62
Q00535	Cyclin-dependent-like kinase 5 OS=Homo sapiens OX=9606 GN=CDK5 PE=1 SV=3	33.3	7.66	CDK5
Q3YEC7	Rab-like protein 6 OS=Homo sapiens OX=9606 GN=RABL6 PE=1 SV=2	79.5	5.22	RABL6
Q9BQ67	Glutamate-rich WD repeat-containing protein 1 OS=Homo sapiens OX=9606 GN=GRWD1 PE=1 SV=1	49.4	4.92	GRWD1
Q709F0	Acyl-CoA dehydrogenase family member 11 OS=Homo sapiens OX=9606 GN=ACAD11 PE=1 SV=3	87.2	8.02	ACAD11
Q0VGL1	Ragulator complex protein LAMTOR4 OS=Homo sapiens OX=9606 GN=LAMTOR4 PE=1 SV=1	10.7	6.54	LAMTOR4
Q9H3S7	Tyrosine-protein phosphatase non-receptor type 23 OS=Homo sapiens OX=9606 GN=PTPN23 PE=1 SV=1	178.9	6.92	PTPN23
Q8TAE8	Growth arrest and DNA damage-inducible proteins-interacting protein 1 OS=Homo sapiens OX=9606 GN=GADD45GIP1 PE=1 SV=1	25.4	10.02	GADD45GIP1

Q96LR5	Ubiquitin-conjugating enzyme E2 E2 OS=Homo sapiens OX=9606 GN=UBE2E2 PE=1 SV=1	22.2	7.71	UBE2E2
Q7Z7F7	39S ribosomal protein L55, mitochondrial OS=Homo sapiens OX=9606 GN=MRPL55 PE=1 SV=1	15.1	11.15	MRPL55
P00846	ATP synthase subunit a OS=Homo sapiens OX=9606 GN=MT-ATP6 PE=1 SV=1	24.8	10.1	MT-ATP6
Q9C0B1	Alpha-ketoglutarate-dependent dioxygenase FTO OS=Homo sapiens OX=9606 GN=FTO PE=1 SV=3	58.2	5.22	FTO
O43290	U4/U6.U5 tri-snRNP-associated protein 1 OS=Homo sapiens OX=9606 GN=SART1 PE=1 SV=1	90.2	6.13	SART1
Q8IZQ5	Selenoprotein H OS=Homo sapiens OX=9606 GN=SELENOH PE=1 SV=2	13.4	9.74	SELENOH
Q9Y315	Deoxyribose-phosphate aldolase OS=Homo sapiens OX=9606 GN=DERA PE=1 SV=2	35.2	8.94	DERA
Q86YP4	Transcriptional repressor p66-alpha OS=Homo sapiens OX=9606 GN=GATAD2A PE=1 SV=1	68	9.94	GATAD2A
Q9H1E5	Thioredoxin-related transmembrane protein 4 OS=Homo sapiens OX=9606 GN=TMX4 PE=1 SV=1	38.9	4.37	TMX4
Q9HBH0	Rho-related GTP-binding protein RhoF OS=Homo sapiens OX=9606 GN=RHOF PE=1 SV=1	23.6	8.65	RHOF
O75352	Mannose-P-dolichol utilization defect 1 protein OS=Homo sapiens OX=9606 GN=MPDU1 PE=1 SV=2	26.6	8.94	MPDU1
Q9H4A6	Golgi phosphoprotein 3 OS=Homo sapiens OX=9606 GN=GOLPH3 PE=1 SV=1	33.8	6.44	GOLPH3
Q567U6	Coiled-coil domain-containing protein 93 OS=Homo sapiens OX=9606 GN=CCDC93 PE=1 SV=2	73.2	8.15	CCDC93
P83876	Thioredoxin-like protein 4A OS=Homo sapiens OX=9606 GN=TXNL4A PE=1 SV=1	16.8	5.85	TXNL4A
Q13136	Liprin-alpha-1 OS=Homo sapiens OX=9606 GN=PPFIA1 PE=1 SV=1	135.7	6.29	PPFIA1
Q8WWC4	m-AAA protease-interacting protein 1, mitochondrial OS=Homo sapiens OX=9606 GN=MAIP1 PE=1 SV=1	32.5	9.17	MAIP1

Q9BYG5	Partitioning defective 6 homolog beta OS=Homo sapiens OX=9606 GN=PARD6B PE=1 SV=1	41.2	5.58	PARD6B
Q01658	Protein Dr1 OS=Homo sapiens OX=9606 GN=DR1 PE=1 SV=1	19.4	4.75	DR1
Q5VTU8	ATP synthase subunit epsilon-like protein, mitochondrial OS=Homo sapiens OX=9606 GN=ATP5F1EP2 PE=1 SV=1	5.8	10.14	ATP5F1EP2
P35240	Merlin OS=Homo sapiens OX=9606 GN=NF2 PE=1 SV=1	69.6	6.47	NF2
Q9UN81	LINE-1 retrotransposable element ORF1 protein OS=Homo sapiens OX=9606 GN=L1RE1 PE=1 SV=1	40	9.51	L1RE1
P49137	MAP kinase-activated protein kinase 2 OS=Homo sapiens OX=9606 GN=MAPKAPK2 PE=1 SV=1	45.5	8.68	MAPKAPK2
Q9H6E4	Coiled-coil domain-containing protein 134 OS=Homo sapiens OX=9606 GN=CCDC134 PE=1 SV=1	26.5	8.85	CCDC134
O75431	Metaxin-2 OS=Homo sapiens OX=9606 GN=MTX2 PE=1 SV=1	29.7	6.29	MTX2
Q9UQ88	Cyclin-dependent kinase 11A OS=Homo sapiens OX=9606 GN=CDK11A PE=1 SV=4	91.3	5.36	CDK11A
Q8IWS0	PHD finger protein 6 OS=Homo sapiens OX=9606 GN=PHF6 PE=1 SV=1	41.3	8.68	PHF6
Q9NZ09	Ubiquitin-associated protein 1 OS=Homo sapiens OX=9606 GN=UBAP1 PE=1 SV=1	55	5.11	UBAP1
Q01968	Inositol polyphosphate 5-phosphatase OCRL OS=Homo sapiens OX=9606 GN=OCRL PE=1 SV=3	104.1	6.55	OCRL
Q16540	39S ribosomal protein L23, mitochondrial OS=Homo sapiens OX=9606 GN=MRPL23 PE=1 SV=1	17.8	9.69	MRPL23
Q96EK6	Glucosamine 6-phosphate N-acetyltransferase OS=Homo sapiens OX=9606 GN=GNPNAT1 PE=1 SV=1	20.7	7.99	GNPNAT1
Q99538	Legumain OS=Homo sapiens OX=9606 GN=LGMN PE=1 SV=1	49.4	6.55	LGMN
Q7Z3U7	Protein MON2 homolog OS=Homo sapiens OX=9606 GN=MON2 PE=1 SV=3	190.2	6.06	MON2

O60264	SWI/SNF-related matrix-associated actin-dependent regulator of chromatin subfamily A member 5 OS=Homo sapiens OX=9606 GN=SMARCA5 PE=1 SV=1	121.8	8.09	SMARCA5
Q13523	Serine/threonine-protein kinase PRP4 homolog OS=Homo sapiens OX=9606 GN=PRPF4B PE=1 SV=3	116.9	10.26	PRPF4B
Q9Y3P9	Rab GTPase-activating protein 1 OS=Homo sapiens OX=9606 GN=RABGAP1 PE=1 SV=3	121.7	5.25	RABGAP1
Q2NL82	Pre-rRNA-processing protein TSR1 homolog OS=Homo sapiens OX=9606 GN=TSR1 PE=1 SV=1	91.8	7.42	TSR1
Q9NWU5	39S ribosomal protein L22, mitochondrial OS=Homo sapiens OX=9606 GN=MRPL22 PE=1 SV=1	23.6	9.94	MRPL22
Q9H0R4	Haloacid dehalogenase-like hydrolase domain-containing protein 2 OS=Homo sapiens OX=9606 GN=HDHD2 PE=1 SV=1	28.5	6.24	HDHD2
Q96FX7	tRNA (adenine(58)-N(1))-methyltransferase catalytic subunit TRMT61A OS=Homo sapiens OX=9606 GN=TRMT61A PE=1 SV=1	31.4	7.36	TRMT61A
Q9H9Q2	COP9 signalosome complex subunit 7b OS=Homo sapiens OX=9606 GN=COPS7B PE=1 SV=1	29.6	6.15	COPS7B
O94900	Thymocyte selection-associated high mobility group box protein TOX OS=Homo sapiens OX=9606 GN=TOX PE=1 SV=3	57.5	7.33	TOX
Q9BV40	Vesicle-associated membrane protein 8 OS=Homo sapiens OX=9606 GN=VAMP8 PE=1 SV=1	11.4	7.34	VAMP8
Q5RKV6	Exosome complex component MTR3 OS=Homo sapiens OX=9606 GN=EXOSC6 PE=1 SV=1	28.2	6.28	EXOSC6
Q9NS69	Mitochondrial import receptor subunit TOM22 homolog OS=Homo sapiens OX=9606 GN=TOMM22 PE=1 SV=3	15.5	4.34	TOMM22
P29558	RNA-binding motif, single-stranded-interacting protein 1 OS=Homo sapiens OX=9606 GN=RBMS1 PE=1 SV=3	44.5	8.79	RBMS1
P18858	DNA ligase 1 OS=Homo sapiens OX=9606 GN=LIG1 PE=1 SV=1	101.7	5.62	LIG1

Q92504	Zinc transporter SLC39A7 OS=Homo sapiens OX=9606 GN=SLC39A7 PE=1 SV=2	50.1	6.87	SLC39A7
Q13188	Serine/threonine-protein kinase 3 OS=Homo sapiens OX=9606 GN=STK3 PE=1 SV=2	56.3	5.24	STK3
Q9UNE7	E3 ubiquitin-protein ligase CHIP OS=Homo sapiens OX=9606 GN=STUB1 PE=1 SV=2	34.8	5.87	STUB1
Q10589	Bone marrow stromal antigen 2 OS=Homo sapiens OX=9606 GN=BST2 PE=1 SV=1	19.8	5.6	BST2
O60524	Nuclear export mediator factor NEMF OS=Homo sapiens OX=9606 GN=NEMF PE=1 SV=4	122.9	6.35	NEMF
Q6P1N9	Putative deoxyribonuclease TATDN1 OS=Homo sapiens OX=9606 GN=TATDN1 PE=1 SV=2	33.6	6.96	TATDN1
Q2M2I8	AP2-associated protein kinase 1 OS=Homo sapiens OX=9606 GN=AAK1 PE=1 SV=3	103.8	6.6	AAK1
Q96SU4	Oxysterol-binding protein-related protein 9 OS=Homo sapiens OX=9606 GN=OSBPL9 PE=1 SV=2	83.1	6.18	OSBPL9
Q9NVT9	Armadillo repeat-containing protein 1 OS=Homo sapiens OX=9606 GN=ARMC1 PE=1 SV=1	31.3	5.74	ARMC1
P98194	Calcium-transporting ATPase type 2C member 1 OS=Homo sapiens OX=9606 GN=ATP2C1 PE=1 SV=3	100.5	6.74	ATP2C1
Q9UQ13	Leucine-rich repeat protein SHOC-2 OS=Homo sapiens OX=9606 GN=SHOC2 PE=1 SV=2	64.8	8.46	SHOC2
Q9HD15	Steroid receptor RNA activator 1 OS=Homo sapiens OX=9606 GN=SRA1 PE=1 SV=1	25.7	7.03	
Q96SB4	SRSF protein kinase 1 OS=Homo sapiens OX=9606 GN=SRPK1 PE=1 SV=2	74.3	6.16	SRPK1
Q96HV5	Transmembrane protein 41A OS=Homo sapiens OX=9606 GN=TMEM41A PE=1 SV=1	29.6	9.03	TMEM41A
Q66PJ3	ADP-ribosylation factor-like protein 6-interacting protein 4 OS=Homo sapiens OX=9606 GN=ARL6IP4 PE=1 SV=2	44.9	10.93	
Q96MV1	TLC domain-containing protein 4 OS=Homo sapiens OX=9606 GN=TLCD4 PE=1 SV=1	30	9.33	TLCD4

Q7LBR1	Charged multivesicular body protein 1b OS=Homo sapiens OX=9606 GN=CHMP1B PE=1 SV=1	22.1	8.1	CHMP1B
Q9NZT2	Opioid growth factor receptor OS=Homo sapiens OX=9606 GN=OGFR PE=1 SV=3	73.3	4.84	OGFR
P46199	Translation initiation factor IF-2, mitochondrial OS=Homo sapiens OX=9606 GN=MTIF2 PE=1 SV=2	81.3	7.15	MTIF2
Q6NXS1	Protein phosphatase inhibitor 2 family member B OS=Homo sapiens OX=9606 GN=PPP1R2B PE=1 SV=2	23.1	4.87	PPP1R2B
Q8NB37	Glutamine amidotransferase-like class 1 domain-containing protein 1 OS=Homo sapiens OX=9606 GN=GATD1 PE=1 SV=1	23.3	6.61	GATD1
Q96G46	tRNA-dihydrouridine(47) synthase [NAD(P)(+)]-like OS=Homo sapiens OX=9606 GN=DUS3L PE=1 SV=2	72.5	8.05	DUS3L
Q99735	Microsomal glutathione S-transferase 2 OS=Homo sapiens OX=9606 GN=MGST2 PE=1 SV=1	16.6	9.55	MGST2
Q6VEQ5	WAS protein family homolog 2 OS=Homo sapiens OX=9606 GN=WASH2P PE=2 SV=2	50.3	5.71	WASH2P
Q15070	Mitochondrial inner membrane protein OXA1L OS=Homo sapiens OX=9606 GN=OXA1L PE=1 SV=3	48.5	9.45	OXA1L
Q6VY07	Phosphofurin acidic cluster sorting protein 1 OS=Homo sapiens OX=9606 GN=PACS1 PE=1 SV=2	104.8	7.74	PACS1
Q15796	Mothers against decapentaplegic homolog 2 OS=Homo sapiens OX=9606 GN=SMAD2 PE=1 SV=1	52.3	6.58	SMAD2
O14579	Coatomer subunit epsilon OS=Homo sapiens OX=9606 GN=COPE PE=1 SV=3	34.5	5.12	COPE
Q9NQ50	39S ribosomal protein L40, mitochondrial OS=Homo sapiens OX=9606 GN=MRPL40 PE=1 SV=1	24.5	9.63	MRPL40
Q96JB2	Conserved oligomeric Golgi complex subunit 3 OS=Homo sapiens OX=9606 GN=COG3 PE=1 SV=3	94	5.57	COG3
Q9BUN8	Derlin-1 OS=Homo sapiens OX=9606 GN=DERL1 PE=1 SV=1	28.8	9.51	DERL1
Q15121	Astrocytic phosphoprotein PEA-15 OS=Homo sapiens OX=9606 GN=PEA15 PE=1 SV=2	15	5.02	PEA15

Q08379	Golgin subfamily A member 2 OS=Homo sapiens OX=9606 GN=GOLGA2 PE=1 SV=3	113	5.02	GOLGA2
Q9Y697	Cysteine desulfurase, mitochondrial OS=Homo sapiens OX=9606 GN=NFS1 PE=1 SV=3	50.2	8.31	NFS1
P55042	GTP-binding protein RAD OS=Homo sapiens OX=9606 GN=RRAD PE=1 SV=2	33.2	8.88	RRAD
Q6YP21	Kynurenine--oxoglutarate transaminase 3 OS=Homo sapiens OX=9606 GN=KYAT3 PE=1 SV=1	51.4	8.19	KYAT3
Q14653	Interferon regulatory factor 3 OS=Homo sapiens OX=9606 GN=IRF3 PE=1 SV=1	47.2	5.34	IRF3
Q8IZ81	ELMO domain-containing protein 2 OS=Homo sapiens OX=9606 GN=ELMOD2 PE=1 SV=1	34.9	8.05	ELMOD2
P04049	RAF proto-oncogene serine/threonine-protein kinase OS=Homo sapiens OX=9606 GN=RAF1 PE=1 SV=1	73	9.2	RAF1
Q9NPL8	Complex I assembly factor TIMMDC1, mitochondrial OS=Homo sapiens OX=9606 GN=TIMMDC1 PE=1 SV=2	32.2	8.5	TIMMDC1
P61966	AP-1 complex subunit sigma-1A OS=Homo sapiens OX=9606 GN=AP1S1 PE=1 SV=1	18.7	5.73	AP1S1
Q13616	Cullin-1 OS=Homo sapiens OX=9606 GN=CUL1 PE=1 SV=2	89.6	8	CUL1
Q9Y496	Kinesin-like protein KIF3A OS=Homo sapiens OX=9606 GN=KIF3A PE=1 SV=4	80	6.54	KIF3A
P01023	Alpha-2-macroglobulin OS=Homo sapiens OX=9606 GN=A2M PE=1 SV=3	163.2	6.46	A2M
P53680	AP-2 complex subunit sigma OS=Homo sapiens OX=9606 GN=AP2S1 PE=1 SV=2	17	6.18	AP2S1
Q96A57	Transmembrane protein 230 OS=Homo sapiens OX=9606 GN=TMEM230 PE=1 SV=1	13.2	9.31	TMEM230
P52948	Nuclear pore complex protein Nup98-Nup96 OS=Homo sapiens OX=9606 GN=NUP98 PE=1 SV=4	197.5	6.4	NUP98
Q08AM6	Protein VAC14 homolog OS=Homo sapiens OX=9606 GN=VAC14 PE=1 SV=1	87.9	6.13	VAC14

O60476	Mannosyl-oligosaccharide 1,2-alpha-mannosidase IB OS=Homo sapiens OX=9606 GN=MAN1A2 PE=1 SV=1	73	7.61	MAN1A2
Q9BPX3	Condensin complex subunit 3 OS=Homo sapiens OX=9606 GN=NCAPG PE=1 SV=1	114.3	5.59	NCAPG
O60942	mRNA-capping enzyme OS=Homo sapiens OX=9606 GN=RNGTT PE=1 SV=1	68.5	8.13	RNGTT
Q96GK7	Fumarylacetoacetate hydrolase domain-containing protein 2A OS=Homo sapiens OX=9606 GN=FAHD2A PE=1 SV=1	34.6	8.24	FAHD2A
Q969T9	WW domain-binding protein 2 OS=Homo sapiens OX=9606 GN=WBP2 PE=1 SV=1	28.1	5.91	WBP2
P82673	28S ribosomal protein S35, mitochondrial OS=Homo sapiens OX=9606 GN=MRPS35 PE=1 SV=1	36.8	8.24	MRPS35
Q13868	Exosome complex component RRP4 OS=Homo sapiens OX=9606 GN=EXOSC2 PE=1 SV=2	32.8	7.5	EXOSC2
Q9Y287	Integral membrane protein 2B OS=Homo sapiens OX=9606 GN=ITM2B PE=1 SV=1	30.3	5.14	ITM2B
P17152	Transmembrane protein 11, mitochondrial OS=Homo sapiens OX=9606 GN=TMEM11 PE=1 SV=1	21.5	7.36	TMEM11
Q9P2B4	CTTNBP2 N-terminal-like protein OS=Homo sapiens OX=9606 GN=CTTNBP2NL PE=1 SV=2	70.1	8.06	CTTNBP2NL
Q99614	Tetratricopeptide repeat protein 1 OS=Homo sapiens OX=9606 GN=TTC1 PE=1 SV=1	33.5	4.84	TTC1
P51159	Ras-related protein Rab-27A OS=Homo sapiens OX=9606 GN=RAB27A PE=1 SV=3	24.9	5.22	RAB27A
P18887	DNA repair protein XRCC1 OS=Homo sapiens OX=9606 GN=XRCC1 PE=1 SV=2	69.4	6.39	
Q96DI7	U5 small nuclear ribonucleoprotein 40 kDa protein OS=Homo sapiens OX=9606 GN=SNRNP40 PE=1 SV=1	39.3	8.1	SNRNP40
Q9P2I0	Cleavage and polyadenylation specificity factor subunit 2 OS=Homo sapiens OX=9606 GN=CPSF2 PE=1 SV=2	88.4	5.11	CPSF2
Q9UPU5	Ubiquitin carboxyl-terminal hydrolase 24 OS=Homo sapiens OX=9606 GN=USP24 PE=1 SV=3	294.2	6.14	USP24

Q13144	Translation initiation factor eIF-2B subunit epsilon OS=Homo sapiens OX=9606 GN=EIF2B5 PE=1 SV=3	80.3	5.08	EIF2B5
Q9UII4	E3 ISG15--protein ligase HERC5 OS=Homo sapiens OX=9606 GN=HERC5 PE=1 SV=2	116.8	7.65	HERC5
Q96ST3	Paired amphipathic helix protein Sin3a OS=Homo sapiens OX=9606 GN=SIN3A PE=1 SV=2	145.1	7.25	SIN3A
Q9NVV5	Androgen-induced gene 1 protein OS=Homo sapiens OX=9606 GN=AIG1 PE=1 SV=3	27.4	7.17	AIG1
Q96EC8	Protein YIPF6 OS=Homo sapiens OX=9606 GN=YIPF6 PE=1 SV=2	26.2	5.64	YIPF6
Q96RP9	Elongation factor G, mitochondrial OS=Homo sapiens OX=9606 GN=GFM1 PE=1 SV=2	83.4	7.01	GFM1
Q9UBU9	Nuclear RNA export factor 1 OS=Homo sapiens OX=9606 GN=NXF1 PE=1 SV=1	70.1	8.51	NXF1
Q96Q11	CCA tRNA nucleotidyltransferase 1, mitochondrial OS=Homo sapiens OX=9606 GN=TRNT1 PE=1 SV=2	50.1	8.1	TRNT1
Q9UHG0	Doublecortin domain-containing protein 2 OS=Homo sapiens OX=9606 GN=DCDC2 PE=1 SV=2	52.8	6.13	DCDC2
Q8WV24	Pleckstrin homology-like domain family A member 1 OS=Homo sapiens OX=9606 GN=PHLDA1 PE=1 SV=4	45	9.89	PHLDA1
Q16527	Cysteine and glycine-rich protein 2 OS=Homo sapiens OX=9606 GN=CSRP2 PE=1 SV=3	20.9	8.62	CSRP2
O94788	Retinal dehydrogenase 2 OS=Homo sapiens OX=9606 GN=ALDH1A2 PE=1 SV=3	56.7	6.05	ALDH1A2
Q96J01	THO complex subunit 3 OS=Homo sapiens OX=9606 GN=THOC3 PE=1 SV=1	38.7	6.09	THOC3
Q9NUQ2	1-acyl-sn-glycerol-3-phosphate acyltransferase epsilon OS=Homo sapiens OX=9606 GN=AGPAT5 PE=1 SV=3	42	9.1	AGPAT5
Q08623	Pseudouridine-5'-phosphatase OS=Homo sapiens OX=9606 GN=PUDP PE=1 SV=3	25.2	5.31	PUDP
Q66K14	TBC1 domain family member 9B OS=Homo sapiens OX=9606 GN=TBC1D9B PE=1 SV=3	140.4	5.25	TBC1D9B

Q4VC31	Protein MIX23 OS=Homo sapiens OX=9606 GN=MIX23 PE=1 SV=1	16.6	7.81	MIX23
Q9UHA4	Ragulator complex protein LAMTOR3 OS=Homo sapiens OX=9606 GN=LAMTOR3 PE=1 SV=1	13.6	7.34	LAMTOR3
O60443	Gasdermin-E OS=Homo sapiens OX=9606 GN=GSDME PE=1 SV=2	54.5	5.17	GSDME
P51809	Vesicle-associated membrane protein 7 OS=Homo sapiens OX=9606 GN=VAMP7 PE=1 SV=3	24.9	8.6	VAMP7
Q8NFV4	Protein ABHD11 OS=Homo sapiens OX=9606 GN=ABHD11 PE=1 SV=1	34.7	9.48	ABHD11
Q9GZZ9	Ubiquitin-like modifier-activating enzyme 5 OS=Homo sapiens OX=9606 GN=UBA5 PE=1 SV=1	44.8	4.84	UBA5
Q9UK59	Lariat debranching enzyme OS=Homo sapiens OX=9606 GN=DBR1 PE=1 SV=2	61.5	5.47	DBR1
Q9NWY4	Histone PARylation factor 1 OS=Homo sapiens OX=9606 GN=HPF1 PE=1 SV=2	39.4	6.8	HPF1
Q99848	Probable rRNA-processing protein EBP2 OS=Homo sapiens OX=9606 GN=EBNA1BP2 PE=1 SV=2	34.8	10.1	EBNA1BP2
Q8TCE6	DENN domain-containing protein 10 OS=Homo sapiens OX=9606 GN=DENND10 PE=1 SV=1	40.5	6.61	DENND10
Q96BN8	Ubiquitin thioesterase otulin OS=Homo sapiens OX=9606 GN=OTULIN PE=1 SV=3	40.2	5.47	OTULIN
Q15262	Receptor-type tyrosine-protein phosphatase kappa OS=Homo sapiens OX=9606 GN=PTPRK PE=1 SV=2	162	5.9	PTPRK
Q9BRX2	Protein pelota homolog OS=Homo sapiens OX=9606 GN=PELO PE=1 SV=2	43.3	6.34	PELO
Q7RTP0	Magnesium transporter NIPA1 OS=Homo sapiens OX=9606 GN=NIPA1 PE=1 SV=1	34.5	8.37	NIPA1
Q92974	Rho guanine nucleotide exchange factor 2 OS=Homo sapiens OX=9606 GN=ARHGEF2 PE=1 SV=4	111.5	7.27	ARHGEF2
O43688	Phospholipid phosphatase 2 OS=Homo sapiens OX=9606 GN=PLPP2 PE=1 SV=1	32.6	8.35	PLPP2

Q9NP77	RNA polymerase II subunit A C-terminal domain phosphatase SSU72 OS=Homo sapiens OX=9606 GN=SSU72 PE=1 SV=1	22.6	5.33	SSU72
Q96H20	Vacuolar-sorting protein SNF8 OS=Homo sapiens OX=9606 GN=SNF8 PE=1 SV=1	28.8	6.65	SNF8
P30530	Tyrosine-protein kinase receptor UFO OS=Homo sapiens OX=9606 GN=AXL PE=1 SV=4	98.3	5.39	AXL
Q68EM7	Rho GTPase-activating protein 17 OS=Homo sapiens OX=9606 GN=ARHGAP17 PE=1 SV=1	95.4	7.62	ARHGAP17
Q9BYX2	TBC1 domain family member 2A OS=Homo sapiens OX=9606 GN=TBC1D2 PE=1 SV=3	105.3	6.58	TBC1D2
Q6PJT7	Zinc finger CCCH domain-containing protein 14 OS=Homo sapiens OX=9606 GN=ZC3H14 PE=1 SV=1	82.8	7.31	ZC3H14
Q9Y2L5	Trafficking protein particle complex subunit 8 OS=Homo sapiens OX=9606 GN=TRAPPC8 PE=1 SV=2	160.9	6.87	TRAPPC8
Q8N6M0	Deubiquitinase OTUD6B OS=Homo sapiens OX=9606 GN=OTUD6B PE=1 SV=1	33.8	6.05	OTUD6B
P49750	YLP motif-containing protein 1 OS=Homo sapiens OX=9606 GN=YLPM1 PE=1 SV=4	241.5	6.55	YLPM1
Q9UQN3	Charged multivesicular body protein 2b OS=Homo sapiens OX=9606 GN=CHMP2B PE=1 SV=1	23.9	8.76	CHMP2B
Q13563	Polycystin-2 OS=Homo sapiens OX=9606 GN=PKD2 PE=1 SV=3	109.6	5.69	PKD2
O15270	Serine palmitoyltransferase 2 OS=Homo sapiens OX=9606 GN=SPTLC2 PE=1 SV=1	62.9	7.78	SPTLC2
O00418	Eukaryotic elongation factor 2 kinase OS=Homo sapiens OX=9606 GN=EEF2K PE=1 SV=2	82.1	5.33	EEF2K
O75150	E3 ubiquitin-protein ligase BRE1B OS=Homo sapiens OX=9606 GN=RNF40 PE=1 SV=5	113.6	6.29	RNF40
Q9BU23	Lipase maturation factor 2 OS=Homo sapiens OX=9606 GN=LMF2 PE=1 SV=2	79.6	10.1	LMF2
Q15477	Helicase SKI2W OS=Homo sapiens OX=9606 GN=SKIV2L PE=1 SV=3	137.7	6.06	SKIV2L

Q9H2M9	Rab3 GTPase-activating protein non-catalytic subunit OS=Homo sapiens OX=9606 GN=RAB3GAP2 PE=1 SV=1	155.9	5.62	RAB3GAP2
Q9BSD7	Cancer-related nucleoside-triphosphatase OS=Homo sapiens OX=9606 GN=NTPCR PE=1 SV=1	20.7	9.54	NTPCR
Q9NZD2	Glycolipid transfer protein OS=Homo sapiens OX=9606 GN=GLTP PE=1 SV=3	23.8	7.39	GLTP
Q9Y673	Dolichyl-phosphate beta-glucosyltransferase OS=Homo sapiens OX=9606 GN=ALG5 PE=1 SV=1	36.9	9.28	ALG5
Q96I59	Probable asparagine--tRNA ligase, mitochondrial OS=Homo sapiens OX=9606 GN=NARS2 PE=1 SV=3	54.1	7.24	NARS2
Q969G3	SWI/SNF-related matrix-associated actin-dependent regulator of chromatin subfamily E member 1 OS=Homo sapiens OX=9606 GN=SMARCE1 PE=1 SV=2	46.6	4.88	SMARCE1
Q96EK4	THAP domain-containing protein 11 OS=Homo sapiens OX=9606 GN=THAP11 PE=1 SV=2	34.4	8.98	THAP11
Q16762	Thiosulfate sulfurtransferase OS=Homo sapiens OX=9606 GN=TST PE=1 SV=4	33.4	7.25	TST
Q92845	Kinesin-associated protein 3 OS=Homo sapiens OX=9606 GN=KIFAP3 PE=1 SV=2	91.1	5.08	KIFAP3
Q9C037	E3 ubiquitin-protein ligase TRIM4 OS=Homo sapiens OX=9606 GN=TRIM4 PE=1 SV=2	57.4	8.1	TRIM4
P17050	Alpha-N-acetylgalactosaminidase OS=Homo sapiens OX=9606 GN=NAGA PE=1 SV=2	46.5	5.19	NAGA
Q16563	Synaptophysin-like protein 1 OS=Homo sapiens OX=9606 GN=SYPL1 PE=1 SV=1	28.5	8.43	SYPL1
Q9Y2S2	Lambda-crystallin homolog OS=Homo sapiens OX=9606 GN=CRYL1 PE=1 SV=3	35.4	6.18	CRYL1
Q9BT73	Proteasome assembly chaperone 3 OS=Homo sapiens OX=9606 GN=PSMG3 PE=1 SV=1	13.1	7.88	PSMG3
Q8IWB7	WD repeat and FYVE domain-containing protein 1 OS=Homo sapiens OX=9606 GN=WDFY1 PE=1 SV=1	46.3	7.33	WDFY1
P80217	Interferon-induced 35 kDa protein OS=Homo sapiens OX=9606 GN=IFI35 PE=1 SV=5	31.5	6.09	IFI35

Q9Y6G5	COMM domain-containing protein 10 OS=Homo sapiens OX=9606 GN=COMMD10 PE=1 SV=1	23	6.54	COMMD10
O43414	ERI1 exoribonuclease 3 OS=Homo sapiens OX=9606 GN=ERI3 PE=1 SV=2	37.2	8.07	ERI3
A6NHR9	Structural maintenance of chromosomes flexible hinge domain-containing protein 1 OS=Homo sapiens OX=9606 GN=SMCHD1 PE=1 SV=2	226.2	7.3	SMCHD1
Q8WVJ2	NudC domain-containing protein 2 OS=Homo sapiens OX=9606 GN=NUDCD2 PE=1 SV=1	17.7	5.07	NUDCD2
Q8N766	ER membrane protein complex subunit 1 OS=Homo sapiens OX=9606 GN=EMC1 PE=1 SV=1	111.7	7.66	EMC1
P30405	Peptidyl-prolyl cis-trans isomerase F, mitochondrial OS=Homo sapiens OX=9606 GN=PPIF PE=1 SV=1	22	9.38	PPIF
Q6NXE6	Armadillo repeat-containing protein 6 OS=Homo sapiens OX=9606 GN=ARMC6 PE=1 SV=2	54.1	6.24	ARMC6
B1AK53	Espin OS=Homo sapiens OX=9606 GN=ESPN PE=1 SV=1	91.7	6.93	ESPN
P29762	Cellular retinoic acid-binding protein 1 OS=Homo sapiens OX=9606 GN=CRABP1 PE=1 SV=2	15.6	5.38	CRABP1
Q8TE77	Protein phosphatase Slingshot homolog 3 OS=Homo sapiens OX=9606 GN=SSH3 PE=1 SV=2	73	5.3	SSH3
Q96AX1	Vacuolar protein sorting-associated protein 33A OS=Homo sapiens OX=9606 GN=VPS33A PE=1 SV=1	67.6	6.96	VPS33A
O95166	Gamma-aminobutyric acid receptor-associated protein OS=Homo sapiens OX=9606 GN=GABARAP PE=1 SV=1	13.9	8.79	GABARAP
O95352	Ubiquitin-like modifier-activating enzyme ATG7 OS=Homo sapiens OX=9606 GN=ATG7 PE=1 SV=1	77.9	6.24	ATG7
Q66K74	Microtubule-associated protein 1S OS=Homo sapiens OX=9606 GN=MAP1S PE=1 SV=2	112.1	7.3	MAP1S
Q6P1M0	Long-chain fatty acid transport protein 4 OS=Homo sapiens OX=9606 GN=SLC27A4 PE=1 SV=1	72	8.47	SLC27A4
Q9Y394	Dehydrogenase/reductase SDR family member 7 OS=Homo sapiens OX=9606 GN=DHRS7 PE=1 SV=1	38.3	8.32	DHRS7

Q3ZAQ7	Vacuolar ATPase assembly integral membrane protein VMA21 OS=Homo sapiens OX=9606 GN=VMA21 PE=1 SV=1	11.3	7.24	VMA21
P50443	Sulfate transporter OS=Homo sapiens OX=9606 GN=SLC26A2 PE=1 SV=2	81.6	8.38	SLC26A2
Q96P16	Regulation of nuclear pre-mRNA domain-containing protein 1A OS=Homo sapiens OX=9606 GN=RPRD1A PE=1 SV=1	35.7	7.55	RPRD1A
Q99496	E3 ubiquitin-protein ligase RING2 OS=Homo sapiens OX=9606 GN=RNF2 PE=1 SV=1	37.6	6.84	RNF2
P49914	5-formyltetrahydrofolate cyclo-ligase OS=Homo sapiens OX=9606 GN=MTHFS PE=1 SV=2	23.2	7.88	MTHFS
Q96JJ3	Engulfment and cell motility protein 2 OS=Homo sapiens OX=9606 GN=ELMO2 PE=1 SV=2	82.6	5.9	ELMO2
O95479	GDH/6PGL endoplasmic bifunctional protein OS=Homo sapiens OX=9606 GN=H6PD PE=1 SV=2	88.8	7.3	H6PD
Q9UBL3	Set1/Ash2 histone methyltransferase complex subunit ASH2 OS=Homo sapiens OX=9606 GN=ASH2L PE=1 SV=1	68.7	5.69	ASH2L
O14972	Vacuolar protein sorting-associated protein 26C OS=Homo sapiens OX=9606 GN=VPS26C PE=1 SV=1	33	7.68	VPS26C
O60568	Multifunctional procollagen lysine hydroxylase and glycosyltransferase LH3 OS=Homo sapiens OX=9606 GN=PLOD3 PE=1 SV=1	84.7	6.05	PLOD3
O00461	Golgi integral membrane protein 4 OS=Homo sapiens OX=9606 GN=GOLIM4 PE=1 SV=1	81.8	4.77	GOLIM4
O15294	UDP-N-acetylglucosamine--peptide N-acetylglucosaminyltransferase 110 kDa subunit OS=Homo sapiens OX=9606 GN=OGT PE=1 SV=3	116.9	6.7	OGT
O00178	GTP-binding protein 1 OS=Homo sapiens OX=9606 GN=GTPBP1 PE=1 SV=3	72.4	8.34	GTPBP1
Q13546	Receptor-interacting serine/threonine-protein kinase 1 OS=Homo sapiens OX=9606 GN=RIPK1 PE=1 SV=3	75.9	6.33	RIPK1

O00483	Cytochrome c oxidase subunit NDUFA4 OS=Homo sapiens OX=9606 GN=NDUFA4 PE=1 SV=1	9.4	9.38	NDUFA4
O94855	Protein transport protein Sec24D OS=Homo sapiens OX=9606 GN=SEC24D PE=1 SV=2	112.9	7.25	SEC24D
Q9P013	Spliceosome-associated protein CWC15 homolog OS=Homo sapiens OX=9606 GN=CWC15 PE=1 SV=2	26.6	5.71	CWC15
P20674	Cytochrome c oxidase subunit 5A, mitochondrial OS=Homo sapiens OX=9606 GN=COX5A PE=1 SV=2	16.8	6.79	COX5A
Q9BQ61	Telomerase RNA component interacting RNase OS=Homo sapiens OX=9606 GN=TRIR PE=1 SV=1	18.4	9.44	TRIR
Q8WXE1	ATR-interacting protein OS=Homo sapiens OX=9606 GN=ATRIP PE=1 SV=1	85.8	6.32	ATRIP
P02100	Hemoglobin subunit epsilon OS=Homo sapiens OX=9606 GN=HBE1 PE=1 SV=2	16.2	8.63	HBE1
Q13601	KRR1 small subunit processome component homolog OS=Homo sapiens OX=9606 GN=KRR1 PE=1 SV=4	43.6	9.77	KRR1
O75629	Protein CREG1 OS=Homo sapiens OX=9606 GN=CREG1 PE=1 SV=1	24.1	7.59	CREG1
Q8NFY4	Semaphorin-6D OS=Homo sapiens OX=9606 GN=SEMA6D PE=1 SV=1	119.8	8.51	SEMA6D
Q9H0S4	Probable ATP-dependent RNA helicase DDX47 OS=Homo sapiens OX=9606 GN=DDX47 PE=1 SV=1	50.6	9.1	DDX47
Q92621	Nuclear pore complex protein Nup205 OS=Homo sapiens OX=9606 GN=NUP205 PE=1 SV=3	227.8	6.19	NUP205
O43819	Protein SCO2 homolog, mitochondrial OS=Homo sapiens OX=9606 GN=SCO2 PE=1 SV=3	29.8	8.85	SCO2
Q92989	Polyribonucleotide 5'-hydroxyl-kinase Clp1 OS=Homo sapiens OX=9606 GN=CLP1 PE=1 SV=1	47.6	6.62	CLP1
Q86WQ0	Nuclear receptor 2C2-associated protein OS=Homo sapiens OX=9606 GN=NR2C2AP PE=1 SV=1	15.9	6.16	NR2C2AP
Q92600	CCR4-NOT transcription complex subunit 9 OS=Homo sapiens OX=9606 GN=CNOT9 PE=1 SV=1	33.6	8.03	CNOT9

Q6ZXV5	Protein O-mannosyl-transferase TMTC3 OS=Homo sapiens OX=9606 GN=TMTC3 PE=1 SV=2	103.9	8.87	TMTC3
Q96RQ3	Methylcrotonoyl-CoA carboxylase subunit alpha, mitochondrial OS=Homo sapiens OX=9606 GN=MCCC1 PE=1 SV=3	80.4	7.78	MCCC1
Q7L2E3	ATP-dependent RNA helicase DHX30 OS=Homo sapiens OX=9606 GN=DHX30 PE=1 SV=1	133.9	8.78	DHX30
Q92552	28S ribosomal protein S27, mitochondrial OS=Homo sapiens OX=9606 GN=MRPS27 PE=1 SV=3	47.6	6.18	MRPS27
Q9BX68	Adenosine 5'-monophosphoramidase HINT2 OS=Homo sapiens OX=9606 GN=HINT2 PE=1 SV=1	17.2	9.16	HINT2
P16220	Cyclic AMP-responsive element-binding protein 1 OS=Homo sapiens OX=9606 GN=CREB1 PE=1 SV=3	35.1	5.27	CREB1
P53611	Geranylgeranyl transferase type-2 subunit beta OS=Homo sapiens OX=9606 GN=RABGGTB PE=1 SV=2	36.9	5.03	RABGGTB
P13726	Tissue factor OS=Homo sapiens OX=9606 GN=F3 PE=1 SV=1	33	7.03	F3
Q96FZ2	Abasic site processing protein HMCES OS=Homo sapiens OX=9606 GN=HMCES PE=1 SV=1	40.5	8.15	HMCES
Q9NUM4	Transmembrane protein 106B OS=Homo sapiens OX=9606 GN=TMEM106B PE=1 SV=2	31.1	6.99	TMEM106B
Q8TF05	Serine/threonine-protein phosphatase 4 regulatory subunit 1 OS=Homo sapiens OX=9606 GN=PPP4R1 PE=1 SV=1	106.9	4.77	PPP4R1
O43719	HIV Tat-specific factor 1 OS=Homo sapiens OX=9606 GN=HTATSF1 PE=1 SV=1	85.8	4.4	HTATSF1
P46937	Transcriptional coactivator YAP1 OS=Homo sapiens OX=9606 GN=YAP1 PE=1 SV=2	54.4	5.17	YAP1
Q969L2	Protein MAL2 OS=Homo sapiens OX=9606 GN=MAL2 PE=1 SV=1	19.1	6.24	MAL2
P42892	Endothelin-converting enzyme 1 OS=Homo sapiens OX=9606 GN=ECE1 PE=1 SV=2	87.1	5.88	ECE1

Q9Y5Q9	General transcription factor 3C polypeptide 3 OS=Homo sapiens OX=9606 GN=GTF3C3 PE=1 SV=1	101.2	5.07	GTF3C3
Q08378	Golgin subfamily A member 3 OS=Homo sapiens OX=9606 GN=GOLGA3 PE=1 SV=2	167.3	5.44	GOLGA3
Q9Y3E0	Vesicle transport protein GOT1B OS=Homo sapiens OX=9606 GN=GOLT1B PE=1 SV=1	15.4	10.36	GOLT1B
P37235	Hippocalcin-like protein 1 OS=Homo sapiens OX=9606 GN=HPCAL1 PE=1 SV=3	22.3	5.35	HPCAL1
Q9HD26	Golgi-associated PDZ and coiled-coil motif-containing protein OS=Homo sapiens OX=9606 GN=GOPC PE=1 SV=1	50.5	5.92	GOPC
Q9Y6I9	Testis-expressed protein 264 OS=Homo sapiens OX=9606 GN=TEX264 PE=1 SV=1	34.2	4.86	TEX264
Q9P032	NADH dehydrogenase [ubiquinone] 1 alpha subcomplex assembly factor 4 OS=Homo sapiens OX=9606 GN=NDUFAF4 PE=1 SV=1	20.3	8.82	NDUFAF4
O14949	Cytochrome b-c1 complex subunit 8 OS=Homo sapiens OX=9606 GN=UQCRQ PE=1 SV=4	9.9	10.08	UQCRQ
Q8IY81	pre-rRNA 2'-O-ribose RNA methyltransferase FTSJ3 OS=Homo sapiens OX=9606 GN=FTSJ3 PE=1 SV=2	96.5	8.4	FTSJ3
O95999	B-cell lymphoma/leukemia 10 OS=Homo sapiens OX=9606 GN=BCL10 PE=1 SV=1	26.2	5.74	BCL10
Q02413	Desmoglein-1 OS=Homo sapiens OX=9606 GN=DSG1 PE=1 SV=2	113.7	5.03	DSG1
O43657	Tetraspanin-6 OS=Homo sapiens OX=9606 GN=TSPAN6 PE=1 SV=1	27.5	8.1	TSPAN6
Q9BSH4	Translational activator of cytochrome c oxidase 1 OS=Homo sapiens OX=9606 GN=TACO1 PE=1 SV=1	32.5	8.13	TACO1
P57772	Selenocysteine-specific elongation factor OS=Homo sapiens OX=9606 GN=EEFSEC PE=1 SV=4	65.3	8.35	EEFSEC
Q9P2X0	Dolichol-phosphate mannosyltransferase subunit 3 OS=Homo sapiens OX=9606 GN=DPM3 PE=1 SV=2	10.1	5.94	DPM3

P42345	Serine/threonine-protein kinase mTOR OS=Homo sapiens OX=9606 GN=MTOR PE=1 SV=1	288.7	7.17	MTOR
Q96QR8	Transcriptional activator protein Pur-beta OS=Homo sapiens OX=9606 GN=PURB PE=1 SV=3	33.2	5.43	PURB
Q96G01	Protein bicaudal D homolog 1 OS=Homo sapiens OX=9606 GN=BICD1 PE=1 SV=3	110.7	5.81	BICD1
Q6P3W7	SCY1-like protein 2 OS=Homo sapiens OX=9606 GN=SCYL2 PE=1 SV=1	103.6	8.22	SCYL2
Q12841	Follistatin-related protein 1 OS=Homo sapiens OX=9606 GN=FSTL1 PE=1 SV=1	35	5.52	FSTL1
O00471	Exocyst complex component 5 OS=Homo sapiens OX=9606 GN=EXOC5 PE=1 SV=1	81.8	6.71	EXOC5
Q8IWZ3	Ankyrin repeat and KH domain-containing protein 1 OS=Homo sapiens OX=9606 GN=ANKHD1 PE=1 SV=1	269.3	5.73	ANKHD1
P49407	Beta-arrestin-1 OS=Homo sapiens OX=9606 GN=ARRB1 PE=1 SV=2	47	6.2	ARRB1
P51668	Ubiquitin-conjugating enzyme E2 D1 OS=Homo sapiens OX=9606 GN=UBE2D1 PE=1 SV=1	16.6	7.42	UBE2D1
Q96CU9	FAD-dependent oxidoreductase domain-containing protein 1 OS=Homo sapiens OX=9606 GN=FOXRED1 PE=1 SV=2	53.8	7.78	FOXRED1
Q14554	Protein disulfide-isomerase A5 OS=Homo sapiens OX=9606 GN=PDIA5 PE=1 SV=1	59.6	7.91	PDIA5
Q5JVF3	PCI domain-containing protein 2 OS=Homo sapiens OX=9606 GN=PCID2 PE=1 SV=2	46	8.53	PCID2
Q03154	Aminoacylase-1 OS=Homo sapiens OX=9606 GN=ACY1 PE=1 SV=1	45.9	6.18	ACY1
P13987	CD59 glycoprotein OS=Homo sapiens OX=9606 GN=CD59 PE=1 SV=1	14.2	6.48	CD59
Q9NPF4	Probable tRNA N6-adenosine threonylcarbamoyltransferase OS=Homo sapiens OX=9606 GN=OSGEP PE=1 SV=1	36.4	6.35	OSGEP

Q5BJH7	Protein YIF1B OS=Homo sapiens OX=9606 GN=YIF1B PE=1 SV=1	34.4	9.16	YIF1B
Q4KWH8	1-phosphatidylinositol 4,5-bisphosphate phosphodiesterase eta-1 OS=Homo sapiens OX=9606 GN=PLCH1 PE=1 SV=1	189.1	7.74	PLCH1
P41743	Protein kinase C iota type OS=Homo sapiens OX=9606 GN=PRKCI PE=1 SV=2	68.2	5.85	PRKCI
Q9UPY5	Cystine/glutamate transporter OS=Homo sapiens OX=9606 GN=SLC7A11 PE=1 SV=1	55.4	9.19	SLC7A11
Q9UKS6	Protein kinase C and casein kinase substrate in neurons protein 3 OS=Homo sapiens OX=9606 GN=PACSIN3 PE=1 SV=2	48.5	6.18	PACSIN3
Q96HU8	GTP-binding protein Di-Ras2 OS=Homo sapiens OX=9606 GN=DIRAS2 PE=1 SV=1	22.5	8.76	DIRAS2
Q9H2D6	TRIO and F-actin-binding protein OS=Homo sapiens OX=9606 GN=TRIOBP PE=1 SV=3	261.2	8.48	TRIOBP
Q96KR1	Zinc finger RNA-binding protein OS=Homo sapiens OX=9606 GN=ZFR PE=1 SV=2	116.9	9.04	ZFR
P50583	Bis(5'-nucleosyl)-tetraphosphatase [asymmetrical] OS=Homo sapiens OX=9606 GN=NUDT2 PE=1 SV=3	16.8	5.35	NUDT2
Q96GC5	39S ribosomal protein L48, mitochondrial OS=Homo sapiens OX=9606 GN=MRPL48 PE=1 SV=2	23.9	8.98	MRPL48
Q6PJ69	Tripartite motif-containing protein 65 OS=Homo sapiens OX=9606 GN=TRIM65 PE=1 SV=3	57.3	6.7	TRIM65
P49356	Protein farnesyltransferase subunit beta OS=Homo sapiens OX=9606 GN=FNTB PE=1 SV=1	48.7	5.82	FNTB
Q9C0H2	Protein tweety homolog 3 OS=Homo sapiens OX=9606 GN=TTYH3 PE=1 SV=3	57.5	5.39	TTYH3
Q8N138	ORM1-like protein 3 OS=Homo sapiens OX=9606 GN=ORMDL3 PE=1 SV=1	17.5	9.64	ORMDL3
Q6MZZ7	Calpain-13 OS=Homo sapiens OX=9606 GN=CAPN13 PE=1 SV=2	76.6	7.39	CAPN13

Q8TEQ6	Gem-associated protein 5 OS=Homo sapiens OX=9606 GN=GEMIN5 PE=1 SV=3	168.5	6.62	GEMIN5
P78508	ATP-sensitive inward rectifier potassium channel 10 OS=Homo sapiens OX=9606 GN=KCNJ10 PE=1 SV=1	42.5	8.06	KCNJ10
P78345	Ribonuclease P protein subunit p38 OS=Homo sapiens OX=9606 GN=RPP38 PE=1 SV=2	31.8	9.92	RPP38
P33947	ER lumen protein-retaining receptor 2 OS=Homo sapiens OX=9606 GN=KDELR2 PE=1 SV=1	24.4	8.72	KDELR2
Q9Y4K3	TNF receptor-associated factor 6 OS=Homo sapiens OX=9606 GN=TRAF6 PE=1 SV=1	59.5	6.44	TRAF6
O00712	Nuclear factor 1 B-type OS=Homo sapiens OX=9606 GN=NFIB PE=1 SV=2	47.4	8.87	NFIB
O14653	Golgi SNAP receptor complex member 2 OS=Homo sapiens OX=9606 GN=GOSR2 PE=1 SV=2	24.8	8.06	GOSR2
O75380	NADH dehydrogenase [ubiquinone] iron-sulfur protein 6, mitochondrial OS=Homo sapiens OX=9606 GN=NDUFS6 PE=1 SV=1	13.7	8.28	NDUFS6
P41223	Protein BUD31 homolog OS=Homo sapiens OX=9606 GN=BUD31 PE=1 SV=2	17	8.82	BUD31
Q13505	Metaxin-1 OS=Homo sapiens OX=9606 GN=MTX1 PE=1 SV=3	51.4	9.79	MTX1
Q9UNI6	Dual specificity protein phosphatase 12 OS=Homo sapiens OX=9606 GN=DUSP12 PE=1 SV=1	37.7	6.84	DUSP12
Q7Z5R6	Amyloid beta A4 precursor protein-binding family B member 1-interacting protein OS=Homo sapiens OX=9606 GN=APBB1IP PE=1 SV=1	73.1	5.59	APBB1IP
Q8ND56	Protein LSM14 homolog A OS=Homo sapiens OX=9606 GN=LSM14A PE=1 SV=3	50.5	9.52	LSM14A
O75792	Ribonuclease H2 subunit A OS=Homo sapiens OX=9606 GN=RNASEH2A PE=1 SV=2	33.4	5.25	RNASEH2A
Q9P016	Thymocyte nuclear protein 1 OS=Homo sapiens OX=9606 GN=THYN1 PE=1 SV=1	25.7	9.25	THYN1

P78346	Ribonuclease P protein subunit p30 OS=Homo sapiens OX=9606 GN=RPP30 PE=1 SV=1	29.3	8.91	RPP30
Q9BU61	NADH dehydrogenase [ubiquinone] 1 alpha subcomplex assembly factor 3 OS=Homo sapiens OX=9606 GN=NDUFAF3 PE=1 SV=1	20.3	8.22	NDUFAF3
Q9NZ45	CDGSH iron-sulfur domain-containing protein 1 OS=Homo sapiens OX=9606 GN=CISD1 PE=1 SV=1	12.2	9.09	CISD1
Q8N183	NADH dehydrogenase [ubiquinone] 1 alpha subcomplex assembly factor 2 OS=Homo sapiens OX=9606 GN=NDUFAF2 PE=1 SV=1	19.8	8.97	NDUFAF2
Q14919	Dr1-associated corepressor OS=Homo sapiens OX=9606 GN=DRAP1 PE=1 SV=3	22.3	5.17	DRAP1
A1L3X0	Elongation of very long chain fatty acids protein 7 OS=Homo sapiens OX=9606 GN=ELOVL7 PE=1 SV=1	33.3	9.26	ELOVL7
P37198	Nuclear pore glycoprotein p62 OS=Homo sapiens OX=9606 GN=NUP62 PE=1 SV=3	53.2	5.31	NUP62
Q9NRW7	Vacuolar protein sorting-associated protein 45 OS=Homo sapiens OX=9606 GN=VPS45 PE=1 SV=1	65	8.24	VPS45
O15498	Synaptobrevin homolog YKT6 OS=Homo sapiens OX=9606 GN=YKT6 PE=1 SV=1	22.4	6.92	YKT6
Q03519	Antigen peptide transporter 2 OS=Homo sapiens OX=9606 GN=TAP2 PE=1 SV=1	75.6	8.02	TAP2
P02788	Lactotransferrin OS=Homo sapiens OX=9606 GN=LTF PE=1 SV=6	78.1	8.12	LTF
Q9NR77	Peroxisomal membrane protein 2 OS=Homo sapiens OX=9606 GN=PXMP2 PE=1 SV=3	22.2	10.58	PXMP2
Q7Z7H5	Transmembrane emp24 domain-containing protein 4 OS=Homo sapiens OX=9606 GN=TMED4 PE=1 SV=1	25.9	8.28	TMED4
P30536	Translocator protein OS=Homo sapiens OX=9606 GN=TSPO PE=1 SV=3	18.8	9.36	TSPO
Q9Y6K9	NF-kappa-B essential modulator OS=Homo sapiens OX=9606 GN=IKBKG PE=1 SV=2	48.2	5.71	IKBKG

Q5T750	Skin-specific protein 32 OS=Homo sapiens OX=9606 GN=XP32 PE=1 SV=1	26.2	7.97	XP32
P21964	Catechol O-methyltransferase OS=Homo sapiens OX=9606 GN=COMT PE=1 SV=2	30	5.47	COMT
Q15388	Mitochondrial import receptor subunit TOM20 homolog OS=Homo sapiens OX=9606 GN=TOMM20 PE=1 SV=1	16.3	8.6	TOMM20
Q14CX7	N-alpha-acetyltransferase 25, NatB auxiliary subunit OS=Homo sapiens OX=9606 GN=NAA25 PE=1 SV=1	112.2	6.64	NAA25
P35610	Sterol O-acyltransferase 1 OS=Homo sapiens OX=9606 GN=SOAT1 PE=1 SV=3	64.7	8.94	SOAT1
Q96H79	Zinc finger CCCH-type antiviral protein 1-like OS=Homo sapiens OX=9606 GN=ZC3HAV1L PE=1 SV=2	32.9	8.13	ZC3HAV1L
Q9BT09	Protein canopy homolog 3 OS=Homo sapiens OX=9606 GN=CNPY3 PE=1 SV=1	30.7	5.49	CNPY3
Q96GX9	Methylthioribulose-1-phosphate dehydratase OS=Homo sapiens OX=9606 GN=APIP PE=1 SV=1	27.1	7.12	APIP
O95807	Transmembrane protein 50A OS=Homo sapiens OX=9606 GN=TMEM50A PE=1 SV=1	17.4	5.87	TMEM50A
Q13287	N-myc-interactor OS=Homo sapiens OX=9606 GN=NMI PE=1 SV=2	35	5.34	NMI
Q14197	Peptidyl-tRNA hydrolase ICT1, mitochondrial OS=Homo sapiens OX=9606 GN=MRPL58 PE=1 SV=1	23.6	10.07	MRPL58
Q2TAA2	Isoamyl acetate-hydrolyzing esterase 1 homolog OS=Homo sapiens OX=9606 GN=IAH1 PE=1 SV=1	27.6	5.3	IAH1
Q99959	Plakophilin-2 OS=Homo sapiens OX=9606 GN=PKP2 PE=1 SV=2	97.4	9.33	PKP2
P56199	Integrin alpha-1 OS=Homo sapiens OX=9606 GN=ITGA1 PE=1 SV=2	130.8	6.29	ITGA1
Q9NY12	H/ACA ribonucleoprotein complex subunit 1 OS=Homo sapiens OX=9606 GN=GAR1 PE=1 SV=1	22.3	10.92	GAR1
P05161	Ubiquitin-like protein ISG15 OS=Homo sapiens OX=9606 GN=ISG15 PE=1 SV=5	17.9	7.44	ISG15

Q9UL33	Trafficking protein particle complex subunit 2-like protein OS=Homo sapiens OX=9606 GN=TRAPPC2L PE=1 SV=1	16.1	6.77	TRAPPC2L
P48506	Glutamate--cysteine ligase catalytic subunit OS=Homo sapiens OX=9606 GN=GCLC PE=1 SV=2	72.7	6.09	GCLC
Q14116	Interleukin-18 OS=Homo sapiens OX=9606 GN=IL18 PE=1 SV=1	22.3	4.67	IL18
Q8TD16	Protein bicaudal D homolog 2 OS=Homo sapiens OX=9606 GN=BICD2 PE=1 SV=1	93.5	5.44	BICD2
Q9NRG0	Chromatin accessibility complex protein 1 OS=Homo sapiens OX=9606 GN=CHRAC1 PE=1 SV=1	14.7	5.1	CHRAC1
Q8IXM6	Nurim OS=Homo sapiens OX=9606 GN=NRM PE=1 SV=1	29.4	8.63	NRM
Q8N3D4	EH domain-binding protein 1-like protein 1 OS=Homo sapiens OX=9606 GN=EHBP1L1 PE=1 SV=2	161.8	4.83	EHBP1L1
Q92785	Zinc finger protein ubi-d4 OS=Homo sapiens OX=9606 GN=DPF2 PE=1 SV=2	44.1	6.33	DPF2
P54687	Branched-chain-amino-acid aminotransferase, cytosolic OS=Homo sapiens OX=9606 GN=BCAT1 PE=1 SV=3	42.9	5.3	BCAT1
Q9GZR7	ATP-dependent RNA helicase DDX24 OS=Homo sapiens OX=9606 GN=DDX24 PE=1 SV=1	96.3	9.06	DDX24
Q13907	Isopentenyl-diphosphate Delta-isomerase 1 OS=Homo sapiens OX=9606 GN=IDI1 PE=1 SV=2	26.3	6.34	IDI1
Q8WW59	SPRY domain-containing protein 4 OS=Homo sapiens OX=9606 GN=SPRYD4 PE=1 SV=2	23.1	6.93	SPRYD4
Q13485	Mothers against decapentaplegic homolog 4 OS=Homo sapiens OX=9606 GN=SMAD4 PE=1 SV=1	60.4	6.99	SMAD4
P61962	DDB1- and CUL4-associated factor 7 OS=Homo sapiens OX=9606 GN=DCAF7 PE=1 SV=1	38.9	5.52	DCAF7
Q5H9R7	Serine/threonine-protein phosphatase 6 regulatory subunit 3 OS=Homo sapiens OX=9606 GN=PPP6R3 PE=1 SV=2	97.6	4.6	PPP6R3

Q7Z3K3	Pogo transposable element with ZNF domain OS=Homo sapiens OX=9606 GN=POGZ PE=1 SV=2	155.2	7.4	POGZ
P55210	Caspase-7 OS=Homo sapiens OX=9606 GN=CASP7 PE=1 SV=1	34.3	6.07	CASP7
Q15750	TGF-beta-activated kinase 1 and MAP3K7-binding protein 1 OS=Homo sapiens OX=9606 GN=TAB1 PE=1 SV=1	54.6	5.52	TAB1
P27707	Deoxycytidine kinase OS=Homo sapiens OX=9606 GN=DCK PE=1 SV=1	30.5	5.21	DCK
P05166	Propionyl-CoA carboxylase beta chain, mitochondrial OS=Homo sapiens OX=9606 GN=PCCB PE=1 SV=3	58.2	7.64	PCCB
Q8WUY1	Protein THEM6 OS=Homo sapiens OX=9606 GN=THEM6 PE=1 SV=2	23.9	9.55	THEM6
P49810	Presenilin-2 OS=Homo sapiens OX=9606 GN=PSEN2 PE=1 SV=1	50.1	4.59	PSEN2
P50336	Protoporphyrinogen oxidase OS=Homo sapiens OX=9606 GN=PPOX PE=1 SV=1	50.7	8.16	PPOX
Q05209	Tyrosine-protein phosphatase non-receptor type 12 OS=Homo sapiens OX=9606 GN=PTPN12 PE=1 SV=3	88.1	5.62	PTPN12
Q9NPI6	mRNA-decapping enzyme 1A OS=Homo sapiens OX=9606 GN=DCP1A PE=1 SV=3	63.2	6.25	DCP1A
Q8WUF5	RelA-associated inhibitor OS=Homo sapiens OX=9606 GN=PPP1R13L PE=1 SV=4	89	6.81	PPP1R13L
O43148	mRNA cap guanine-N7 methyltransferase OS=Homo sapiens OX=9606 GN=RNMT PE=1 SV=1	54.8	6.61	RNMT
Q9BUP3	Oxidoreductase HTATIP2 OS=Homo sapiens OX=9606 GN=HTATIP2 PE=1 SV=2	27	8.38	HTATIP2
Q9GZN8	UPF0687 protein C20orf27 OS=Homo sapiens OX=9606 GN=C20orf27 PE=1 SV=3	19.3	6.84	C20orf27
P48723	Heat shock 70 kDa protein 13 OS=Homo sapiens OX=9606 GN=HSPA13 PE=1 SV=1	51.9	5.76	HSPA13
O95297	Myelin protein zero-like protein 1 OS=Homo sapiens OX=9606 GN=MPZL1 PE=1 SV=1	29.1	8.72	MPZL1

Q658Y4	Protein FAM91A1 OS=Homo sapiens OX=9606 GN=FAM91A1 PE=1 SV=3	93.9	6.39	FAM91A1
Q8NEW0	Zinc transporter 7 OS=Homo sapiens OX=9606 GN=SLC30A7 PE=2 SV=1	41.6	6.95	SLC30A7
O96005	Cleft lip and palate transmembrane protein 1 OS=Homo sapiens OX=9606 GN=CLPTM1 PE=1 SV=1	76	6.3	CLPTM1
P51608	Methyl-CpG-binding protein 2 OS=Homo sapiens OX=9606 GN=MECP2 PE=1 SV=1	52.4	9.95	MECP2
Q92979	Ribosomal RNA small subunit methyltransferase NEP1 OS=Homo sapiens OX=9606 GN=EMG1 PE=1 SV=4	26.7	9.17	EMG1
Q8NFP7	Diphosphoinositol polyphosphate phosphohydrolase 3-alpha OS=Homo sapiens OX=9606 GN=NUDT10 PE=1 SV=1	18.5	5.68	NUDT10
Q9HD45	Transmembrane 9 superfamily member 3 OS=Homo sapiens OX=9606 GN=TM9SF3 PE=1 SV=2	67.8	7.21	TM9SF3
Q5T160	Probable arginine--tRNA ligase, mitochondrial OS=Homo sapiens OX=9606 GN=RARS2 PE=1 SV=1	65.5	8.21	RARS2
P51532	Transcription activator BRG1 OS=Homo sapiens OX=9606 GN=SMARCA4 PE=1 SV=2	184.5	7.88	SMARCA4
Q9UI30	Multifunctional methyltransferase subunit TRM112-like protein OS=Homo sapiens OX=9606 GN=TRMT112 PE=1 SV=1	14.2	5.26	TRMT112
Q8N511	Transmembrane protein 199 OS=Homo sapiens OX=9606 GN=TMEM199 PE=1 SV=1	23.1	9.01	TMEM199
O15230	Laminin subunit alpha-5 OS=Homo sapiens OX=9606 GN=LAMA5 PE=1 SV=8	399.5	7.02	LAMA5
Q01970	1-phosphatidylinositol 4,5-bisphosphate phosphodiesterase beta-3 OS=Homo sapiens OX=9606 GN=PLCB3 PE=1 SV=2	138.7	5.9	PLCB3
Q9Y2R0	Cytochrome c oxidase assembly factor 3 homolog, mitochondrial OS=Homo sapiens OX=9606 GN=COA3 PE=1 SV=1	11.7	9.6	COA3

Q9BYC5	Alpha-(1,6)-fucosyltransferase OS=Homo sapiens OX=9606 GN=FUT8 PE=1 SV=2	66.5	7.66	FUT8
Q9H1Y0	Autophagy protein 5 OS=Homo sapiens OX=9606 GN=ATG5 PE=1 SV=2	32.4	5.77	ATG5
Q7Z392	Trafficking protein particle complex subunit 11 OS=Homo sapiens OX=9606 GN=TRAPPC11 PE=1 SV=2	128.8	7.14	TRAPPC11
Q12972	Nuclear inhibitor of protein phosphatase 1 OS=Homo sapiens OX=9606 GN=PPP1R8 PE=1 SV=2	38.5	7.37	PPP1R8
P46379	Large proline-rich protein BAG6 OS=Homo sapiens OX=9606 GN=BAG6 PE=1 SV=2	119.3	5.6	BAG6
Q13191	E3 ubiquitin-protein ligase CBL-B OS=Homo sapiens OX=9606 GN=CBLB PE=1 SV=2	109.4	7.88	CBLB
Q68CP4	Heparan-alpha-glucosaminide N-acetyltransferase OS=Homo sapiens OX=9606 GN=HGSNAT PE=1 SV=2	73.2	8.4	HGSNAT
Q9UJW0	Dynactin subunit 4 OS=Homo sapiens OX=9606 GN=DCTN4 PE=1 SV=1	52.3	7.34	DCTN4
P40938	Replication factor C subunit 3 OS=Homo sapiens OX=9606 GN=RFC3 PE=1 SV=2	40.5	8.34	RFC3
Q9UK45	U6 snRNA-associated Sm-like protein LSm7 OS=Homo sapiens OX=9606 GN=LSM7 PE=1 SV=1	11.6	5.27	LSM7
Q06265	Exosome complex component RRP45 OS=Homo sapiens OX=9606 GN=EXOSC9 PE=1 SV=3	48.9	5.29	EXOSC9
Q5UIP0	Telomere-associated protein RIF1 OS=Homo sapiens OX=9606 GN=RIF1 PE=1 SV=2	274.3	5.52	RIF1
Q9UBB5	Methyl-CpG-binding domain protein 2 OS=Homo sapiens OX=9606 GN=MBD2 PE=1 SV=1	43.2	10.04	MBD2
P22681	E3 ubiquitin-protein ligase CBL OS=Homo sapiens OX=9606 GN=CBL PE=1 SV=2	99.6	6.54	CBL
Q04941	Proteolipid protein 2 OS=Homo sapiens OX=9606 GN=PLP2 PE=1 SV=1	16.7	7.24	PLP2
Q86VH2	Kinesin-like protein KIF27 OS=Homo sapiens OX=9606 GN=KIF27 PE=1 SV=1	160.2	7.28	KIF27

Q86WA6	Valacyclovir hydrolase OS=Homo sapiens OX=9606 GN=BPHL PE=1 SV=1	32.5	9.14	BPHL
Q13123	Protein Red OS=Homo sapiens OX=9606 GN=IK PE=1 SV=3	65.6	6.64	IK
Q9BVQ7	Spermatogenesis-associated protein 5-like protein 1 OS=Homo sapiens OX=9606 GN=SPATA5L1 PE=1 SV=2	80.7	8.09	SPATA5L1
P53634	Dipeptidyl peptidase 1 OS=Homo sapiens OX=9606 GN=CTSC PE=1 SV=2	51.8	6.99	CTSC
Q8IXJ9	Polycomb group protein ASXL1 OS=Homo sapiens OX=9606 GN=ASXL1 PE=1 SV=4	165.3	6.21	ASXL1
Q9NU23	LYR motif-containing protein 2 OS=Homo sapiens OX=9606 GN=LYRM2 PE=1 SV=1	10.4	10.46	LYRM2
O95749	Geranylgeranyl pyrophosphate synthase OS=Homo sapiens OX=9606 GN=GGPS1 PE=1 SV=1	34.8	6.14	GGPS1
Q8TCG1	Protein CIP2A OS=Homo sapiens OX=9606 GN=CIP2A PE=1 SV=2	102.1	6.23	CIP2A
O14530	Thioredoxin domain-containing protein 9 OS=Homo sapiens OX=9606 GN=TXNDC9 PE=1 SV=2	26.5	5.88	TXNDC9
Q9Y4D8	Probable E3 ubiquitin-protein ligase HECTD4 OS=Homo sapiens OX=9606 GN=HECTD4 PE=1 SV=5	439.1	6.19	HECTD4
Q9NRY5	Protein FAM114A2 OS=Homo sapiens OX=9606 GN=FAM114A2 PE=1 SV=4	55.4	4.88	FAM114A2
Q8N3U4	Cohesin subunit SA-2 OS=Homo sapiens OX=9606 GN=STAG2 PE=1 SV=3	141.2	5.43	STAG2
P62310	U6 snRNA-associated Sm-like protein LSm3 OS=Homo sapiens OX=9606 GN=LSM3 PE=1 SV=2	11.8	4.7	LSM3
Q7Z408	CUB and sushi domain-containing protein 2 OS=Homo sapiens OX=9606 GN=CSMD2 PE=1 SV=2	379.8	6.09	CSMD2
Q8N878	FERM domain-containing protein 1 OS=Homo sapiens OX=9606 GN=FRMD1 PE=1 SV=2	62.5	8.21	FRMD1
O75146	Huntingtin-interacting protein 1-related protein OS=Homo sapiens OX=9606 GN=HIP1R PE=1 SV=2	119.3	6.67	HIP1R

Q93034	Cullin-5 OS=Homo sapiens OX=9606 GN=CUL5 PE=1 SV=4	90.9	7.94	CUL5
B2RXF5	Zinc finger and BTB domain-containing protein 42 OS=Homo sapiens OX=9606 GN=ZBTB42 PE=1 SV=2	46.5	8.47	ZBTB42
Q9GZY8	Mitochondrial fission factor OS=Homo sapiens OX=9606 GN=MFF PE=1 SV=1	38.4	8.95	MFF
Q9H446	RWD domain-containing protein 1 OS=Homo sapiens OX=9606 GN=RWDD1 PE=1 SV=1	27.9	4.2	RWDD1
Q9BZL1	Ubiquitin-like protein 5 OS=Homo sapiens OX=9606 GN=UBL5 PE=1 SV=1	8.5	8.44	UBL5
Q8NBN7	Retinol dehydrogenase 13 OS=Homo sapiens OX=9606 GN=RDH13 PE=1 SV=2	35.9	8.1	RDH13
O60673	DNA polymerase zeta catalytic subunit OS=Homo sapiens OX=9606 GN=REV3L PE=1 SV=2	352.6	8.47	REV3L
Q9H694	Protein bicaudal C homolog 1 OS=Homo sapiens OX=9606 GN=BICC1 PE=1 SV=2	104.8	8.54	BICC1
Q02880	DNA topoisomerase 2-beta OS=Homo sapiens OX=9606 GN=TOP2B PE=1 SV=3	183.2	8	TOP2B
Q12789	General transcription factor 3C polypeptide 1 OS=Homo sapiens OX=9606 GN=GTF3C1 PE=1 SV=4	238.7	7.3	GTF3C1
Q96CM8	Medium-chain acyl-CoA ligase ACSF2, mitochondrial OS=Homo sapiens OX=9606 GN=ACSF2 PE=1 SV=2	68.1	7.55	ACSF2
Q9P2B2	Prostaglandin F2 receptor negative regulator OS=Homo sapiens OX=9606 GN=PTGFRN PE=1 SV=2	98.5	6.61	PTGFRN
P01024	Complement C3 OS=Homo sapiens OX=9606 GN=C3 PE=1 SV=2	187	6.4	C3
Q7Z417	Nuclear fragile X mental retardation-interacting protein 2 OS=Homo sapiens OX=9606 GN=NUFIP2 PE=1 SV=1	76.1	8.7	NUFIP2
Q8IWX8	Calcium homeostasis endoplasmic reticulum protein OS=Homo sapiens OX=9606 GN=CHERP PE=1 SV=3	103.6	9.04	CHERP
Q13464	Rho-associated protein kinase 1 OS=Homo sapiens OX=9606 GN=ROCK1 PE=1 SV=1	158.1	5.9	ROCK1

Q8N9N7	Leucine-rich repeat-containing protein 57 OS=Homo sapiens OX=9606 GN=LRRC57 PE=1 SV=1	26.7	8.43	LRRC57
Q9BV44	THUMP domain-containing protein 3 OS=Homo sapiens OX=9606 GN=THUMPD3 PE=1 SV=1	57	6.37	THUMPD3
Q2TAL8	Transcriptional regulator QRICH1 OS=Homo sapiens OX=9606 GN=QRICH1 PE=1 SV=1	86.4	5.87	QRICH1
Q9Y5I4	Protocadherin alpha-C2 OS=Homo sapiens OX=9606 GN=PCDHAC2 PE=2 SV=1	109.4	5.41	PCDHAC2
Q86TU7	Actin-histidine N-methyltransferase OS=Homo sapiens OX=9606 GN=SETD3 PE=1 SV=1	67.2	5.96	SETD3
P51553	Isocitrate dehydrogenase [NAD] subunit gamma, mitochondrial OS=Homo sapiens OX=9606 GN=IDH3G PE=1 SV=1	42.8	8.5	IDH3G
P31944	Caspase-14 OS=Homo sapiens OX=9606 GN=CASP14 PE=1 SV=2	27.7	5.58	CASP14
O15269	Serine palmitoyltransferase 1 OS=Homo sapiens OX=9606 GN=SPTLC1 PE=1 SV=1	52.7	6.01	SPTLC1
O60725	Protein-S-isoprenylcysteine O-methyltransferase OS=Homo sapiens OX=9606 GN=ICMT PE=1 SV=1	31.9	7.96	ICMT
Q8NFD5	AT-rich interactive domain-containing protein 1B OS=Homo sapiens OX=9606 GN=ARID1B PE=1 SV=2	236	6.73	ARID1B
Q9Y2K1	Zinc finger and BTB domain-containing protein 1 OS=Homo sapiens OX=9606 GN=ZBTB1 PE=1 SV=3	82	6.39	ZBTB1
Q8IYJ2	Uncharacterized protein C10orf67, mitochondrial OS=Homo sapiens OX=9606 GN=C10orf67 PE=1 SV=3	63.6	8.84	C10orf67
Q9NVS2	39S ribosomal protein S18a, mitochondrial OS=Homo sapiens OX=9606 GN=MRPS18A PE=1 SV=1	22.2	10.33	MRPS18A
P07358	Complement component C8 beta chain OS=Homo sapiens OX=9606 GN=C8B PE=1 SV=3	67	8.13	C8B
Q6P1M3	LLGL scribble cell polarity complex component 2 OS=Homo sapiens OX=9606 GN=LLGL2 PE=1 SV=2	113.4	7.52	LLGL2
P00414	Cytochrome c oxidase subunit 3 OS=Homo sapiens OX=9606 GN=MT-CO3 PE=1 SV=2	29.9	7.31	MT-CO3

P98095	Fibulin-2 OS=Homo sapiens OX=9606 GN=FBLN2 PE=1 SV=2	126.5	4.82	FBLN2
Q9UI14	Prenylated Rab acceptor protein 1 OS=Homo sapiens OX=9606 GN=RABAC1 PE=1 SV=1	20.6	7.34	RABAC1
Q8WUM0	Nuclear pore complex protein Nup133 OS=Homo sapiens OX=9606 GN=NUP133 PE=1 SV=2	128.9	5.1	NUP133
Q15042	Rab3 GTPase-activating protein catalytic subunit OS=Homo sapiens OX=9606 GN=RAB3GAP1 PE=1 SV=3	110.5	5.55	RAB3GAP1
Q8NGS6	Olfactory receptor 13C3 OS=Homo sapiens OX=9606 GN=OR13C3 PE=2 SV=2	38.7	8.54	OR13C3
Q6JEL2	Kelch-like protein 10 OS=Homo sapiens OX=9606 GN=KLHL10 PE=1 SV=1	68.9	5.68	KLHL10
Q96BW9	Phosphatidate cytidylyltransferase, mitochondrial OS=Homo sapiens OX=9606 GN=TAMM41 PE=1 SV=2	51	7.94	TAMM41
O60437	Periplakin OS=Homo sapiens OX=9606 GN=PPL PE=1 SV=4	204.6	5.6	PPL
Q9NVP1	ATP-dependent RNA helicase DDX18 OS=Homo sapiens OX=9606 GN=DDX18 PE=1 SV=2	75.4	9.5	DDX18
Q9BV38	WD repeat-containing protein 18 OS=Homo sapiens OX=9606 GN=WDR18 PE=1 SV=2	47.4	6.7	WDR18
Q96BW5	Phosphotriesterase-related protein OS=Homo sapiens OX=9606 GN=PTER PE=1 SV=1	39	6.52	PTER
Q9UP83	Conserved oligomeric Golgi complex subunit 5 OS=Homo sapiens OX=9606 GN=COG5 PE=1 SV=3	92.7	6.6	COG5
O95817	BAG family molecular chaperone regulator 3 OS=Homo sapiens OX=9606 GN=BAG3 PE=1 SV=3	61.6	6.95	BAG3
Q9Y657	Spindlin-1 OS=Homo sapiens OX=9606 GN=SPIN1 PE=1 SV=3	29.6	6.96	SPIN1
Q15131	Cyclin-dependent kinase 10 OS=Homo sapiens OX=9606 GN=CDK10 PE=1 SV=1	41	8.84	CDK10
Q9Y679	Lipid droplet-regulating VLDL assembly factor AUP1 OS=Homo sapiens OX=9606 GN=AUP1 PE=1 SV=2	45.8	8.65	AUP1

P10606	Cytochrome c oxidase subunit 5B, mitochondrial OS=Homo sapiens OX=9606 GN=COX5B PE=1 SV=2	13.7	8.81	COX5B
Q9Y446	Plakophilin-3 OS=Homo sapiens OX=9606 GN=PKP3 PE=1 SV=1	87	9.32	PKP3
O95372	Acyl-protein thioesterase 2 OS=Homo sapiens OX=9606 GN=LYPLA2 PE=1 SV=1	24.7	7.23	LYPLA2
Q10570	Cleavage and polyadenylation specificity factor subunit 1 OS=Homo sapiens OX=9606 GN=CPSF1 PE=1 SV=2	160.8	6.4	CPSF1
Q8NG11	Tetraspanin-14 OS=Homo sapiens OX=9606 GN=TSPAN14 PE=1 SV=1	30.7	6.84	TSPAN14
P82932	28S ribosomal protein S6, mitochondrial OS=Homo sapiens OX=9606 GN=MRPS6 PE=1 SV=3	14.2	9.26	MRPS6
Q53TN4	Plasma membrane ascorbate-dependent reductase CYBRD1 OS=Homo sapiens OX=9606 GN=CYBRD1 PE=1 SV=1	31.6	8.76	CYBRD1
Q8IWJ2	GRIP and coiled-coil domain-containing protein 2 OS=Homo sapiens OX=9606 GN=GCC2 PE=1 SV=4	195.8	5.14	GCC2
Q8NHP8	Putative phospholipase B-like 2 OS=Homo sapiens OX=9606 GN=PLBD2 PE=1 SV=2	65.4	6.8	PLBD2
Q86WB0	Nuclear-interacting partner of ALK OS=Homo sapiens OX=9606 GN=ZC3HC1 PE=1 SV=1	55.2	5.62	ZC3HC1
Q9BV35	Calcium-binding mitochondrial carrier protein SCaMC-3 OS=Homo sapiens OX=9606 GN=SLC25A23 PE=1 SV=2	52.3	7.33	SLC25A23
P82909	28S ribosomal protein S36, mitochondrial OS=Homo sapiens OX=9606 GN=MRPS36 PE=1 SV=2	11.5	9.99	MRPS36
Q9NS61	Kv channel-interacting protein 2 OS=Homo sapiens OX=9606 GN=KCNIP2 PE=1 SV=3	30.9	5.11	KCNIP2
Q06203	Amidophosphoribosyltransferase OS=Homo sapiens OX=9606 GN=PPAT PE=1 SV=1	57.4	6.76	PPAT
Q9UBX7	Kallikrein-11 OS=Homo sapiens OX=9606 GN=KLK11 PE=1 SV=2	31	8.94	KLK11
Q9Y2I6	Ninein-like protein OS=Homo sapiens OX=9606 GN=NINL PE=1 SV=2	156.2	5.06	NINL

Q8NB25	Protein FAM184A OS=Homo sapiens OX=9606 GN=FAM184A PE=1 SV=3	132.9	5.83	FAM184A
Q9C0E2	Exportin-4 OS=Homo sapiens OX=9606 GN=XPO4 PE=1 SV=2	130.1	5.05	XPO4
Q4V328	GRIP1-associated protein 1 OS=Homo sapiens OX=9606 GN=GRIPAP1 PE=1 SV=2	95.9	5.11	GRIPAP1
Q8IW35	Centrosomal protein of 97 kDa OS=Homo sapiens OX=9606 GN=CEP97 PE=1 SV=1	96.9	5.02	CEP97
Q6UW63	Protein O-glucosyltransferase 2 OS=Homo sapiens OX=9606 GN=POGLUT2 PE=1 SV=1	58	7.71	POGLUT2
Q96RT7	Gamma-tubulin complex component 6 OS=Homo sapiens OX=9606 GN=TUBGCP6 PE=1 SV=3	200.4	6.32	TUBGCP6
O75051	Plexin-A2 OS=Homo sapiens OX=9606 GN=PLXNA2 PE=1 SV=4	211	6.48	PLXNA2
Q9HCU4	Cadherin EGF LAG seven-pass G-type receptor 2 OS=Homo sapiens OX=9606 GN=CELSR2 PE=1 SV=1	317.3	5.31	CELSR2
P17568	NADH dehydrogenase [ubiquinone] 1 beta subcomplex subunit 7 OS=Homo sapiens OX=9606 GN=NDUFB7 PE=1 SV=4	16.4	8.92	NDUFB7
Q9H1K1	Iron-sulfur cluster assembly enzyme ISCU, mitochondrial OS=Homo sapiens OX=9606 GN=ISCU PE=1 SV=2	18	9.48	ISCU
Q9UBM7	7-dehydrocholesterol reductase OS=Homo sapiens OX=9606 GN=DHCR7 PE=1 SV=1	54.5	8.7	DHCR7
Q13952	Nuclear transcription factor Y subunit gamma OS=Homo sapiens OX=9606 GN=NFYC PE=1 SV=3	50.3	6.1	NFYC
Q9Y6I3	Epsin-1 OS=Homo sapiens OX=9606 GN=EPN1 PE=1 SV=2	60.3	4.83	EPN1
Q13033	Striatin-3 OS=Homo sapiens OX=9606 GN=STRN3 PE=1 SV=3	87.2	5.36	STRN3
Q8N9T8	Protein KRI1 homolog OS=Homo sapiens OX=9606 GN=KRI1 PE=1 SV=3	82.5	5.14	KRI1
Q9UJX2	Cell division cycle protein 23 homolog OS=Homo sapiens OX=9606 GN=CDC23 PE=1 SV=3	68.8	7.02	CDC23

O95182	NADH dehydrogenase [ubiquinone] 1 alpha subcomplex subunit 7 OS=Homo sapiens OX=9606 GN=NDUFA7 PE=1 SV=3	12.5	10.18	NDUFA7
Q9Y5L0	Transportin-3 OS=Homo sapiens OX=9606 GN=TNPO3 PE=1 SV=3	104.1	5.57	TNPO3
Q16678	Cytochrome P450 1B1 OS=Homo sapiens OX=9606 GN=CYP1B1 PE=1 SV=2	60.8	8.98	CYP1B1
Q15904	V-type proton ATPase subunit S1 OS=Homo sapiens OX=9606 GN=ATP6AP1 PE=1 SV=2	52	6.14	ATP6AP1
Q86VM9	Zinc finger CCCH domain-containing protein 18 OS=Homo sapiens OX=9606 GN=ZC3H18 PE=1 SV=2	106.3	8.32	ZC3H18
Q13572	Inositol-tetrakisphosphate 1-kinase OS=Homo sapiens OX=9606 GN=ITPK1 PE=1 SV=2	45.6	6.16	ITPK1
O60934	Nibrin OS=Homo sapiens OX=9606 GN=NBN PE=1 SV=1	84.9	6.9	NBN
Q14160	Protein scribble homolog OS=Homo sapiens OX=9606 GN=SCRIB PE=1 SV=4	174.8	5.07	
Q0VDF9	Heat shock 70 kDa protein 14 OS=Homo sapiens OX=9606 GN=HSPA14 PE=1 SV=1	54.8	5.59	HSPA14
Q13232	Nucleoside diphosphate kinase 3 OS=Homo sapiens OX=9606 GN=NME3 PE=1 SV=2	19	7.84	NME3
Q05086	Ubiquitin-protein ligase E3A OS=Homo sapiens OX=9606 GN=UBE3A PE=1 SV=4	100.6	5.22	UBE3A
P57740	Nuclear pore complex protein Nup107 OS=Homo sapiens OX=9606 GN=NUP107 PE=1 SV=1	106.3	5.43	NUP107
P52657	Transcription initiation factor IIA subunit 2 OS=Homo sapiens OX=9606 GN=GTF2A2 PE=1 SV=1	12.4	6.62	GTF2A2
O95447	Lebercilin-like protein OS=Homo sapiens OX=9606 GN=LCA5L PE=1 SV=1	76.5	9.48	LCA5L
Q8TAV4	Stomatin-like protein 3 OS=Homo sapiens OX=9606 GN=STOML3 PE=1 SV=1	32.1	8.63	STOML3
O94886	CSC1-like protein 1 OS=Homo sapiens OX=9606 GN=TMEM63A PE=1 SV=3	92.1	7.27	TMEM63A

P42336	Phosphatidylinositol 4,5-bisphosphate 3-kinase catalytic subunit alpha isoform OS=Homo sapiens OX=9606 GN=PIK3CA PE=1 SV=2	124.2	7.23	PIK3CA
P0CF97	Protein FAM200B OS=Homo sapiens OX=9606 GN=FAM200B PE=3 SV=1	76	8.07	FAM200B
O95260	Arginyl-tRNA--protein transferase 1 OS=Homo sapiens OX=9606 GN=ATE1 PE=1 SV=2	59.1	7.93	ATE1
Q8IZ52	Chondroitin sulfate synthase 2 OS=Homo sapiens OX=9606 GN=CHPF PE=1 SV=2	85.4	6.93	CHPF
P24462	Cytochrome P450 3A7 OS=Homo sapiens OX=9606 GN=CYP3A7 PE=1 SV=3	57.4	9.09	CYP3A7
Q9BRQ6	MICOS complex subunit MIC25 OS=Homo sapiens OX=9606 GN=CHCHD6 PE=1 SV=1	26.4	8.85	CHCHD6
Q9H175	Cysteine/serine-rich nuclear protein 2 OS=Homo sapiens OX=9606 GN=CSRNP2 PE=1 SV=1	59.6	4.77	CSRNP2
P20962	Parathymosin OS=Homo sapiens OX=9606 GN=PTMS PE=1 SV=2	11.5	4.16	PTMS
Q96JM3	Chromosome alignment-maintaining phosphoprotein 1 OS=Homo sapiens OX=9606 GN=CHAMP1 PE=1 SV=2	89	8.44	CHAMP1
O75323	Protein NipSnap homolog 2 OS=Homo sapiens OX=9606 GN=NIPSNAP2 PE=1 SV=1	33.7	9.36	NIPSNAP2
Q9ULP9	TBC1 domain family member 24 OS=Homo sapiens OX=9606 GN=TBC1D24 PE=1 SV=2	62.9	7.36	TBC1D24
O00267	Transcription elongation factor SPT5 OS=Homo sapiens OX=9606 GN=SUPT5H PE=1 SV=1	120.9	5.06	SUPT5H
Q99732	Lipopolysaccharide-induced tumor necrosis factor-alpha factor OS=Homo sapiens OX=9606 GN=LITAF PE=1 SV=2	17.1	6.44	LITAF
P62341	Thioredoxin reductase-like selenoprotein T OS=Homo sapiens OX=9606 GN=SELENOT PE=1 SV=2	22.3	8.6	SELENOT
Q6YHK3	CD109 antigen OS=Homo sapiens OX=9606 GN=CD109 PE=1 SV=2	161.6	5.85	CD109
Q13257	Mitotic spindle assembly checkpoint protein MAD2A OS=Homo sapiens OX=9606 GN=MAD2L1 PE=1 SV=1	23.5	5.08	MAD2L1

Q9UKX7	Nuclear pore complex protein Nup50 OS=Homo sapiens OX=9606 GN=NUP50 PE=1 SV=2	50.1	7.06	NUP50
Q4J6C6	Prolyl endopeptidase-like OS=Homo sapiens OX=9606 GN=PREPL PE=1 SV=1	83.9	6.38	PREPL
Q587I9	Vesicle transport protein SFT2C OS=Homo sapiens OX=9606 GN=SFT2D3 PE=2 SV=1	21.8	9.89	SFT2D3
Q9H6D7	HAUS augmin-like complex subunit 4 OS=Homo sapiens OX=9606 GN=HAUS4 PE=1 SV=1	42.4	5.68	HAUS4
Q8NCX0	Coiled-coil domain-containing protein 150 OS=Homo sapiens OX=9606 GN=CCDC150 PE=1 SV=2	128.7	7.02	CCDC150
Q9NSV4	Protein diaphanous homolog 3 OS=Homo sapiens OX=9606 GN=DIAPH3 PE=1 SV=4	136.8	7.03	DIAPH3
Q5SZD4	Glycine N-acyltransferase-like protein 3 OS=Homo sapiens OX=9606 GN=GLYATL3 PE=2 SV=3	32.7	8.84	GLYATL3
O00244	Copper transport protein ATOX1 OS=Homo sapiens OX=9606 GN=ATOX1 PE=1 SV=1	7.4	7.24	ATOX1
Q9Y6X4	Soluble lamin-associated protein of 75 kDa OS=Homo sapiens OX=9606 GN=FAM169A PE=1 SV=2	74.9	4.6	FAM169A
Q9BSR8	Protein YIPF4 OS=Homo sapiens OX=9606 GN=YIPF4 PE=1 SV=1	27.1	4.65	YIPF4
Q9UEW8	STE20/SPS1-related proline-alanine-rich protein kinase OS=Homo sapiens OX=9606 GN=STK39 PE=1 SV=3	59.4	6.29	STK39
Q06546	GA-binding protein alpha chain OS=Homo sapiens OX=9606 GN=GABPA PE=1 SV=1	51.3	4.97	GABPA
Q9BPU6	Dihydropyrimidinase-related protein 5 OS=Homo sapiens OX=9606 GN=DPYSL5 PE=1 SV=1	61.4	7.2	DPYSL5
P29034	Protein S100-A2 OS=Homo sapiens OX=9606 GN=S100A2 PE=1 SV=3	11.1	4.78	S100A2
Q9NVH6	Trimethyllysine dioxygenase, mitochondrial OS=Homo sapiens OX=9606 GN=TMLHE PE=1 SV=1	49.5	7.72	TMLHE
Q8IYG6	Leucine-rich repeat-containing protein 56 OS=Homo sapiens OX=9606 GN=LRRC56 PE=1 SV=1	58.7	7.9	LRRC56

Q9UG01	Intraflagellar transport protein 172 homolog OS=Homo sapiens OX=9606 GN=IFT172 PE=1 SV=2	197.5	6.13	IFT172
P57678	Gem-associated protein 4 OS=Homo sapiens OX=9606 GN=GEMIN4 PE=1 SV=2	120	6.04	GEMIN4
Q96BJ3	Axin interactor, dorsalization-associated protein OS=Homo sapiens OX=9606 GN=AIDA PE=1 SV=1	35	6.55	AIDA
Q9HCH0	Nck-associated protein 5-like OS=Homo sapiens OX=9606 GN=NCKAP5L PE=1 SV=3	139.3	8.06	
Q9NS91	E3 ubiquitin-protein ligase RAD18 OS=Homo sapiens OX=9606 GN=RAD18 PE=1 SV=2	56.2	7.58	RAD18
O43617	Trafficking protein particle complex subunit 3 OS=Homo sapiens OX=9606 GN=TRAPPC3 PE=1 SV=1	20.3	4.96	TRAPPC3
O14495	Phospholipid phosphatase 3 OS=Homo sapiens OX=9606 GN=PLPP3 PE=1 SV=1	35.1	9.14	PLPP3
O14521	Succinate dehydrogenase [ubiquinone] cytochrome b small subunit, mitochondrial OS=Homo sapiens OX=9606 GN=SDHD PE=1 SV=1	17	8.63	SDHD
Q15018	BRISC complex subunit Abraxas 2 OS=Homo sapiens OX=9606 GN=ABRAXAS2 PE=1 SV=2	46.9	6.21	ABRAXAS2
Q5EBL8	PDZ domain-containing protein 11 OS=Homo sapiens OX=9606 GN=PDZD11 PE=1 SV=2	16.1	7.21	PDZD11
Q9Y2U5	Mitogen-activated protein kinase kinase kinase 2 OS=Homo sapiens OX=9606 GN=MAP3K2 PE=1 SV=2	69.7	8	MAP3K2
Q96C10	Probable ATP-dependent RNA helicase DHX58 OS=Homo sapiens OX=9606 GN=DHX58 PE=1 SV=1	76.6	7.37	DHX58
Q5H9L2	Transcription elongation factor A protein-like 5 OS=Homo sapiens OX=9606 GN=TCEAL5 PE=1 SV=1	23.3	4.81	TCEAL5
P54840	Glycogen [starch] synthase, liver OS=Homo sapiens OX=9606 GN=GYS2 PE=1 SV=2	80.9	6.83	GYS2
Q8NDY3	[Protein ADP-ribosylarginine] hydrolase-like protein 1 OS=Homo sapiens OX=9606 GN=ADPRHL1 PE=2 SV=1	40.1	5.85	ADPRHL1
Q96JK9	Mastermind-like protein 3 OS=Homo sapiens OX=9606 GN=MAML3 PE=1 SV=4	122.2	7.52	MAML3

P28340	DNA polymerase delta catalytic subunit OS=Homo sapiens OX=9606 GN=POLD1 PE=1 SV=2	123.6	7.03	POLD1
Q8WXQ8	Carboxypeptidase A5 OS=Homo sapiens OX=9606 GN=CPA5 PE=2 SV=1	49	6.73	CPA5
O00482	Nuclear receptor subfamily 5 group A member 2 OS=Homo sapiens OX=9606 GN=NR5A2 PE=1 SV=2	61.3	7.88	NR5A2
Q8IWU2	Serine/threonine-protein kinase LMTK2 OS=Homo sapiens OX=9606 GN=LMTK2 PE=1 SV=2	164.8	4.48	LMTK2
Q96CX2	BTB/POZ domain-containing protein KCTD12 OS=Homo sapiens OX=9606 GN=KCTD12 PE=1 SV=1	35.7	5.64	KCTD12
Q9P2K3	REST corepressor 3 OS=Homo sapiens OX=9606 GN=RCOR3 PE=1 SV=2	55.5	8.27	RCOR3
Q8WUY3	Protein prune homolog 2 OS=Homo sapiens OX=9606 GN=PRUNE2 PE=1 SV=3	340.4	4.45	PRUNE2
Q9NZ43	Vesicle transport protein USE1 OS=Homo sapiens OX=9606 GN=USE1 PE=1 SV=2	29.4	9.07	USE1
Q99543	DnaJ homolog subfamily C member 2 OS=Homo sapiens OX=9606 GN=DNAJC2 PE=1 SV=4	72	8.7	DNAJC2
Q8IWW6	Rho GTPase-activating protein 12 OS=Homo sapiens OX=9606 GN=ARHGAP12 PE=1 SV=1	96.2	7.61	ARHGAP12
O60909	Beta-1,4-galactosyltransferase 2 OS=Homo sapiens OX=9606 GN=B4GALT2 PE=1 SV=1	41.9	9.38	B4GALT2
Q56NI9	N-acetyltransferase ESCO2 OS=Homo sapiens OX=9606 GN=ESCO2 PE=1 SV=1	68.3	9.39	ESCO2
P53701	Holocytochrome c-type synthase OS=Homo sapiens OX=9606 GN=HCCS PE=1 SV=1	30.6	6.68	HCCS
O00401	Neural Wiskott-Aldrich syndrome protein OS=Homo sapiens OX=9606 GN=WASL PE=1 SV=2	54.8	7.93	WASL
O00142	Thymidine kinase 2, mitochondrial OS=Homo sapiens OX=9606 GN=TK2 PE=1 SV=4	31	8.46	TK2
Q96AP0	Adrenocortical dysplasia protein homolog OS=Homo sapiens OX=9606 GN=ACD PE=1 SV=4	48.9	5.3	ACD

Q8N6T3	ADP-ribosylation factor GTPase-activating protein 1 OS=Homo sapiens OX=9606 GN=ARFGAP1 PE=1 SV=2	44.6	5.66	ARFGAP1
P49840	Glycogen synthase kinase-3 alpha OS=Homo sapiens OX=9606 GN=GSK3A PE=1 SV=2	50.9	8.75	GSK3A
Q5SW79	Centrosomal protein of 170 kDa OS=Homo sapiens OX=9606 GN=CEP170 PE=1 SV=1	175.2	7.11	CEP170
O43660	Pleiotropic regulator 1 OS=Homo sapiens OX=9606 GN=PLRG1 PE=1 SV=1	57.2	9.17	PLRG1
Q9NPR2	Semaphorin-4B OS=Homo sapiens OX=9606 GN=SEMA4B PE=1 SV=4	92.7	7.05	SEMA4B
Q96RP7	Galactose-3-O-sulfotransferase 4 OS=Homo sapiens OX=9606 GN=GAL3ST4 PE=1 SV=1	54.1	10.18	GAL3ST4
Q86X83	COMM domain-containing protein 2 OS=Homo sapiens OX=9606 GN=COMMD2 PE=1 SV=2	22.7	6.73	COMMD2
Q9UBN7	Histone deacetylase 6 OS=Homo sapiens OX=9606 GN=HDAC6 PE=1 SV=2	131.3	5.3	HDAC6
Q8NGA1	Olfactory receptor 1M1 OS=Homo sapiens OX=9606 GN=OR1M1 PE=2 SV=1	34.8	8.91	OR1M1
Q6WQI6	Putative cancer susceptibility gene HEPN1 protein OS=Homo sapiens OX=9606 GN=HEPN1 PE=5 SV=2	10.3	8.28	HEPN1
Q96P63	Serpin B12 OS=Homo sapiens OX=9606 GN=SERPINB12 PE=1 SV=1	46.2	5.53	SERPINB12
Q8IUR7	Armadillo repeat-containing protein 8 OS=Homo sapiens OX=9606 GN=ARMC8 PE=1 SV=2	75.5	6.73	ARMC8
O75925	E3 SUMO-protein ligase PIAS1 OS=Homo sapiens OX=9606 GN=PIAS1 PE=1 SV=2	71.8	7.3	PIAS1
P05165	Propionyl-CoA carboxylase alpha chain, mitochondrial OS=Homo sapiens OX=9606 GN=PCCA PE=1 SV=4	80	7.52	PCCA
Q16222	UDP-N-acetylhexosamine pyrophosphorylase OS=Homo sapiens OX=9606 GN=UAP1 PE=1 SV=3	58.7	6.33	UAP1
A2RTX5	Threonine--tRNA ligase 2, cytoplasmic OS=Homo sapiens OX=9606 GN=TARS3 PE=1 SV=1	92.6	6.05	TARS3

Q9NR16	Scavenger receptor cysteine-rich type 1 protein M160 OS=Homo sapiens OX=9606 GN=CD163L1 PE=1 SV=2	159.1	5.76	CD163L1
Q8NEU8	DCC-interacting protein 13-beta OS=Homo sapiens OX=9606 GN=APPL2 PE=1 SV=3	74.4	4.94	APPL2
Q6NUN0	Acyl-coenzyme A synthetase ACSM5, mitochondrial OS=Homo sapiens OX=9606 GN=ACSM5 PE=1 SV=2	64.7	8.4	ACSM5
O75152	Zinc finger CCCH domain-containing protein 11A OS=Homo sapiens OX=9606 GN=ZC3H11A PE=1 SV=3	89.1	8.37	ZC3H11A
Q00872	Myosin-binding protein C, slow-type OS=Homo sapiens OX=9606 GN=MYBPC1 PE=1 SV=2	128.2	6.04	MYBPC1
P50895	Basal cell adhesion molecule OS=Homo sapiens OX=9606 GN=BCAM PE=1 SV=2	67.4	5.81	BCAM
Q6N021	Methylcytosine dioxygenase TET2 OS=Homo sapiens OX=9606 GN=TET2 PE=1 SV=3	223.7	7.99	TET2
P15328	Folate receptor alpha OS=Homo sapiens OX=9606 GN=FOLR1 PE=1 SV=3	29.8	7.97	FOLR1
P05107	Integrin beta-2 OS=Homo sapiens OX=9606 GN=ITGB2 PE=1 SV=2	84.7	6.95	ITGB2
Q9Y6K5	2'-5'-oligoadenylate synthase 3 OS=Homo sapiens OX=9606 GN=OAS3 PE=1 SV=3	121.1	8.4	OAS3
P49336	Cyclin-dependent kinase 8 OS=Homo sapiens OX=9606 GN=CDK8 PE=1 SV=1	53.3	8.57	CDK8
A0AUZ9	KAT8 regulatory NSL complex subunit 1-like protein OS=Homo sapiens OX=9606 GN=KANSL1L PE=1 SV=2	112.2	8.44	KANSL1L
O15211	Ral guanine nucleotide dissociation stimulator-like 2 OS=Homo sapiens OX=9606 GN=RGL2 PE=1 SV=1	83.5	6.18	RGL2
Q9NW64	Pre-mRNA-splicing factor RBM22 OS=Homo sapiens OX=9606 GN=RBM22 PE=1 SV=1	46.9	8.54	RBM22
Q03164	Histone-lysine N-methyltransferase 2A OS=Homo sapiens OX=9606 GN=KMT2A PE=1 SV=5	431.5	9.09	KMT2A
P19878	Neutrophil cytosol factor 2 OS=Homo sapiens OX=9606 GN=NCF2 PE=1 SV=2	59.7	6.16	NCF2

Q9P0V3	SH3 domain-binding protein 4 OS=Homo sapiens OX=9606 GN=SH3BP4 PE=1 SV=1	107.4	7.71	SH3BP4
O60494	Cubilin OS=Homo sapiens OX=9606 GN=CUBN PE=1 SV=5	398.5	5.35	CUBN
O95400	CD2 antigen cytoplasmic tail-binding protein 2 OS=Homo sapiens OX=9606 GN=CD2BP2 PE=1 SV=1	37.6	4.61	CD2BP2
Q9P2H0	Centrosomal protein of 126 kDa OS=Homo sapiens OX=9606 GN=CEP126 PE=1 SV=3	125.8	8.76	CEP126
Q99988	Growth/differentiation factor 15 OS=Homo sapiens OX=9606 GN=GDF15 PE=1 SV=3	34.1	9.66	GDF15
Q96QG7	Myotubularin-related protein 9 OS=Homo sapiens OX=9606 GN=MTMR9 PE=1 SV=1	63.4	6.39	MTMR9
Q8IWR0	Zinc finger CCCH domain-containing protein 7A OS=Homo sapiens OX=9606 GN=ZC3H7A PE=1 SV=1	110.5	7.3	ZC3H7A
Q8TE85	Grainyhead-like protein 3 homolog OS=Homo sapiens OX=9606 GN=GRHL3 PE=1 SV=3	70.3	6.84	GRHL3
P82933	28S ribosomal protein S9, mitochondrial OS=Homo sapiens OX=9606 GN=MRPS9 PE=1 SV=2	45.8	9.51	MRPS9
Q07817	Bcl-2-like protein 1 OS=Homo sapiens OX=9606 GN=BCL2L1 PE=1 SV=1	26	4.93	BCL2L1
Q9UNE2	Rab effector Noc2 OS=Homo sapiens OX=9606 GN=RPH3AL PE=1 SV=1	34.4	8.98	RPH3AL
P78426	Homeobox protein Nkx-6.1 OS=Homo sapiens OX=9606 GN=NKX6-1 PE=1 SV=2	37.8	9.45	NKX6-1
O43674	NADH dehydrogenase [ubiquinone] 1 beta subcomplex subunit 5, mitochondrial OS=Homo sapiens OX=9606 GN=NDUFB5 PE=1 SV=1	21.7	9.63	NDUFB5
Q8N1G2	Cap-specific mRNA (nucleoside-2'-O-)-methyltransferase 1 OS=Homo sapiens OX=9606 GN=CMTR1 PE=1 SV=1	95.3	7.05	CMTR1
Q7KZ85	Transcription elongation factor SPT6 OS=Homo sapiens OX=9606 GN=SUPT6H PE=1 SV=2	198.9	4.91	SUPT6H

P21953	2-oxoisovalerate dehydrogenase subunit beta, mitochondrial OS=Homo sapiens OX=9606 GN=BCKDHB PE=1 SV=2	43.1	6.29	BCKDHB
P52564	Dual specificity mitogen-activated protein kinase kinase 6 OS=Homo sapiens OX=9606 GN=MAP2K6 PE=1 SV=1	37.5	7.39	MAP2K6
Q8IVT5	Kinase suppressor of Ras 1 OS=Homo sapiens OX=9606 GN=KSR1 PE=1 SV=3	102.1	8.66	KSR1
Q9HD34	LYR motif-containing protein 4 OS=Homo sapiens OX=9606 GN=LYRM4 PE=1 SV=1	10.8	10.73	LYRM4
Q9NZN9	Aryl-hydrocarbon-interacting protein-like 1 OS=Homo sapiens OX=9606 GN=AIPL1 PE=1 SV=2	43.9	5.95	AIPL1
Q8N2C7	Protein unc-80 homolog OS=Homo sapiens OX=9606 GN=UNC80 PE=1 SV=2	363.2	6.86	UNC80
Q8WU58	Protein FAM222B OS=Homo sapiens OX=9606 GN=FAM222B PE=1 SV=1	59.6	9.23	FAM222B
Q8NF91	Nesprin-1 OS=Homo sapiens OX=9606 GN=SYNE1 PE=1 SV=4	1010.5	5.53	SYNE1
O00115	Deoxyribonuclease-2-alpha OS=Homo sapiens OX=9606 GN=DNASE2 PE=1 SV=2	39.6	8.05	DNASE2
O60637	Tetraspanin-3 OS=Homo sapiens OX=9606 GN=TSPAN3 PE=2 SV=1	28	5.81	TSPAN3
A0A087X0K7	Probable non-functional T cell receptor beta variable 17 OS=Homo sapiens OX=9606 GN=TRBV17 PE=1 SV=6	13	7.94	TRBV17
Q8WYA6	Beta-catenin-like protein 1 OS=Homo sapiens OX=9606 GN=CTNNBL1 PE=1 SV=1	65.1	5.05	CTNNBL1
Q01433	AMP deaminase 2 OS=Homo sapiens OX=9606 GN=AMPD2 PE=1 SV=2	100.6	6.93	AMPD2
Q6DKJ4	Nucleoredoxin OS=Homo sapiens OX=9606 GN=NXN PE=1 SV=2	48.4	4.97	NXN
P23142	Fibulin-1 OS=Homo sapiens OX=9606 GN=FBLN1 PE=1 SV=4	77.2	5.22	FBLN1
D6RIA3	Uncharacterized protein C4orf54 OS=Homo sapiens OX=9606 GN=C4orf54 PE=2 SV=1	190	8.98	C4orf54

Q8N584	Tetratricopeptide repeat protein 39C OS=Homo sapiens OX=9606 GN=TTC39C PE=2 SV=2	65.8	6.99	TTC39C
P17900	Ganglioside GM2 activator OS=Homo sapiens OX=9606 GN=GM2A PE=1 SV=4	20.8	5.31	GM2A
Q9H7T0	Cation channel sperm-associated protein subunit beta OS=Homo sapiens OX=9606 GN=CATSPERB PE=2 SV=2	126.8	7.17	CATSPERB
Q8N957	Ankyrin repeat and fibronectin type-III domain-containing protein 1 OS=Homo sapiens OX=9606 GN=ANKFN1 PE=2 SV=2	87.5	8.4	ANKFN1
O75674	TOM1-like protein 1 OS=Homo sapiens OX=9606 GN=TOM1L1 PE=1 SV=2	53	5.24	TOM1L1
Q9UKD2	mRNA turnover protein 4 homolog OS=Homo sapiens OX=9606 GN=MRTO4 PE=1 SV=2	27.5	8.29	MRTO4
Q5VYS8	Terminal uridylyltransferase 7 OS=Homo sapiens OX=9606 GN=TUT7 PE=1 SV=1	171.1	6.83	TUT7
O60925	Prefoldin subunit 1 OS=Homo sapiens OX=9606 GN=PFDN1 PE=1 SV=2	14.2	6.81	PFDN1
P25490	Transcriptional repressor protein YY1 OS=Homo sapiens OX=9606 GN=YY1 PE=1 SV=2	44.7	6.25	YY1
Q5THK1	Protein PRR14L OS=Homo sapiens OX=9606 GN=PRR14L PE=1 SV=1	237.2	6.33	PRR14L
P43250	G protein-coupled receptor kinase 6 OS=Homo sapiens OX=9606 GN=GRK6 PE=1 SV=2	65.9	8	GRK6
P20849	Collagen alpha-1(IX) chain OS=Homo sapiens OX=9606 GN=COL9A1 PE=1 SV=3	91.8	8.72	COL9A1
Q15027	Arf-GAP with coiled-coil, ANK repeat and PH domain-containing protein 1 OS=Homo sapiens OX=9606 GN=ACAP1 PE=1 SV=1	81.5	7.66	ACAP1
Q15031	Probable leucine--tRNA ligase, mitochondrial OS=Homo sapiens OX=9606 GN=LARS2 PE=1 SV=2	101.9	8.22	LARS2
P42858	Huntingtin OS=Homo sapiens OX=9606 GN=HTT PE=1 SV=2	347.4	6.2	HTT

Q9HA65	TBC1 domain family member 17 OS=Homo sapiens OX=9606 GN=TBC1D17 PE=1 SV=2	72.7	5.19	
Q76NI1	Kinase non-catalytic C-lobe domain-containing protein 1 OS=Homo sapiens OX=9606 GN=KNDC1 PE=2 SV=2	191.3	6.16	KNDC1
Q8IZ21	Phosphatase and actin regulator 4 OS=Homo sapiens OX=9606 GN=PHACTR4 PE=1 SV=1	78.2	6.62	PHACTR4
Q13402	Unconventional myosin-VIIa OS=Homo sapiens OX=9606 GN=MYO7A PE=1 SV=2	254.2	8.57	MYO7A
Q96JN2	Coiled-coil domain-containing protein 136 OS=Homo sapiens OX=9606 GN=CCDC136 PE=1 SV=3	134	4.65	CCDC136
Q05397	Focal adhesion kinase 1 OS=Homo sapiens OX=9606 GN=PTK2 PE=1 SV=2	119.2	6.62	PTK2
Q5SXH7	Pleckstrin homology domain-containing family S member 1 OS=Homo sapiens OX=9606 GN=PLEKHS1 PE=1 SV=4	52.4	5.2	PLEKHS1
Q9Y210	Short transient receptor potential channel 6 OS=Homo sapiens OX=9606 GN=TRPC6 PE=1 SV=1	106.3	6.67	TRPC6
O95436	Sodium-dependent phosphate transport protein 2B OS=Homo sapiens OX=9606 GN=SLC34A2 PE=1 SV=3	75.7	8.15	SLC34A2
P24928	DNA-directed RNA polymerase II subunit RPB1 OS=Homo sapiens OX=9606 GN=POLR2A PE=1 SV=2	217	7.37	POLR2A
Q5SYE7	NHS-like protein 1 OS=Homo sapiens OX=9606 GN=NHSL1 PE=1 SV=2	170.6	6.96	NHSL1
B5MCY1	Tudor domain-containing protein 15 OS=Homo sapiens OX=9606 GN=TDRD15 PE=2 SV=1	221.6	7.03	TDRD15
Q13416	Origin recognition complex subunit 2 OS=Homo sapiens OX=9606 GN=ORC2 PE=1 SV=2	65.9	6.51	ORC2
Q9HCG8	Pre-mRNA-splicing factor CWC22 homolog OS=Homo sapiens OX=9606 GN=CWC22 PE=1 SV=3	105.4	7.03	CWC22
Q5T653	39S ribosomal protein L2, mitochondrial OS=Homo sapiens OX=9606 GN=MRPL2 PE=1 SV=2	33.3	11.3	MRPL2
Q9ULX9	Transcription factor MafF OS=Homo sapiens OX=9606 GN=MAFF PE=1 SV=2	17.7	9.8	MAFF

D6RBQ6	Ubiquitin carboxyl-terminal hydrolase 17-like protein 17 OS=Homo sapiens OX=9606 GN=USP17L17 PE=3 SV=1	59.6	7.99	USP17L17
Q9H159	Cadherin-19 OS=Homo sapiens OX=9606 GN=CDH19 PE=2 SV=1	86.9	4.73	CDH19
P33241	Lymphocyte-specific protein 1 OS=Homo sapiens OX=9606 GN=LSP1 PE=1 SV=1	37.2	4.74	LSP1
Q8NHV9	Rhox homeobox family member 1 OS=Homo sapiens OX=9606 GN=RHOXF1 PE=1 SV=1	20.5	6.11	RHOXF1
Q86V40	Metalloprotease TIKI1 OS=Homo sapiens OX=9606 GN=TRABD2A PE=2 SV=3	57.6	8.85	TRABD2A
P83111	Serine beta-lactamase-like protein LACTB, mitochondrial OS=Homo sapiens OX=9606 GN=LACTB PE=1 SV=2	60.7	8.53	LACTB
Q13325	Interferon-induced protein with tetratricopeptide repeats 5 OS=Homo sapiens OX=9606 GN=IFIT5 PE=1 SV=1	55.8	7.4	IFIT5
Q9HA77	Probable cysteine--tRNA ligase, mitochondrial OS=Homo sapiens OX=9606 GN=CARS2 PE=1 SV=1	62.2	8.34	CARS2
O75031	Heat shock factor 2-binding protein OS=Homo sapiens OX=9606 GN=HSF2BP PE=1 SV=1	37.6	5.53	HSF2BP
P49593	Protein phosphatase 1F OS=Homo sapiens OX=9606 GN=PPM1F PE=1 SV=3	49.8	5.1	PPM1F
Q15058	Kinesin-like protein KIF14 OS=Homo sapiens OX=9606 GN=KIF14 PE=1 SV=1	186.4	7.91	KIF14
Q8IYS2	Uncharacterized protein KIAA2013 OS=Homo sapiens OX=9606 GN=KIAA2013 PE=1 SV=1	69.1	8.19	KIAA2013
Q96N16	Janus kinase and microtubule-interacting protein 1 OS=Homo sapiens OX=9606 GN=JAKMIP1 PE=1 SV=1	73.2	6.1	JAKMIP1
Q13362	Serine/threonine-protein phosphatase 2A 56 kDa regulatory subunit gamma isoform OS=Homo sapiens OX=9606 GN=PPP2R5C PE=1 SV=3	61	6.87	PPP2R5C
Q676U5	Autophagy-related protein 16-1 OS=Homo sapiens OX=9606 GN=ATG16L1 PE=1 SV=2	68.2	6.64	ATG16L1

Q99567	Nuclear pore complex protein Nup88 OS=Homo sapiens OX=9606 GN=NUP88 PE=1 SV=2	83.5	5.69	NUP88
P48165	Gap junction alpha-8 protein OS=Homo sapiens OX=9606 GN=GJA8 PE=1 SV=3	48.2	5.27	GJA8
Q9ULL8	Protein Shroom4 OS=Homo sapiens OX=9606 GN=SHROOM4 PE=1 SV=3	164.8	6.65	SHROOM4
Q96QU4	Protein FRG2-like-1 OS=Homo sapiens OX=9606 GN=FRG2B PE=2 SV=1	30.5	8.03	FRG2B
O95159	Zinc finger protein-like 1 OS=Homo sapiens OX=9606 GN=ZFPL1 PE=1 SV=2	34.1	8.07	ZFPL1
Q8TB22	Spermatogenesis-associated protein 20 OS=Homo sapiens OX=9606 GN=SPATA20 PE=2 SV=3	87.8	7.43	SPATA20
P16035	Metalloproteinase inhibitor 2 OS=Homo sapiens OX=9606 GN=TIMP2 PE=1 SV=2	24.4	7.49	TIMP2
Q99808	Equilibrative nucleoside transporter 1 OS=Homo sapiens OX=9606 GN=SLC29A1 PE=1 SV=3	50.2	8.29	SLC29A1
Q8N7Z5	Ankyrin repeat domain-containing protein 31 OS=Homo sapiens OX=9606 GN=ANKRD31 PE=2 SV=2	210.7	6.2	ANKRD31
Q5VZK9	F-actin-uncapping protein LRRC16A OS=Homo sapiens OX=9606 GN=CARMIL1 PE=1 SV=1	151.5	7.85	CARMIL1
Q9H7C4	Syncoilin OS=Homo sapiens OX=9606 GN=SYNC PE=1 SV=3	55.3	4.61	SYNC
Q9BPX6	Calcium uptake protein 1, mitochondrial OS=Homo sapiens OX=9606 GN=MICU1 PE=1 SV=1	54.3	8.41	MICU1
P03956	Interstitial collagenase OS=Homo sapiens OX=9606 GN=MMP1 PE=1 SV=3	54	6.96	MMP1
P54252	Ataxin-3 OS=Homo sapiens OX=9606 GN=ATXN3 PE=1 SV=5	41.2	4.78	ATXN3
Q8TCU6	Phosphatidylinositol 3,4,5-trisphosphate-dependent Rac exchanger 1 protein OS=Homo sapiens OX=9606 GN=PREX1 PE=1 SV=3	186.1	6.44	PREX1
A8MWX3	Putative WAS protein family homolog 4 OS=Homo sapiens OX=9606 GN=WASH4P PE=5 SV=1	51.6	6.67	WASH4P

O15056	Synaptojanin-2 OS=Homo sapiens OX=9606 GN=SYNJ2 PE=1 SV=3	165.4	7.34	SYNJ2
Q8N140	EP300-interacting inhibitor of differentiation 3 OS=Homo sapiens OX=9606 GN=EID3 PE=1 SV=1	38.1	5.15	EID3
Q96A49	Synapse-associated protein 1 OS=Homo sapiens OX=9606 GN=SYAP1 PE=1 SV=1	39.9	4.53	SYAP1
Q8TDZ2	[F-actin]-monooxygenase MICAL1 OS=Homo sapiens OX=9606 GN=MICAL1 PE=1 SV=2	117.8	6.4	MICAL1
Q7Z3B4	Nucleoporin p54 OS=Homo sapiens OX=9606 GN=NUP54 PE=1 SV=2	55.4	7.02	NUP54
Q86W42	THO complex subunit 6 homolog OS=Homo sapiens OX=9606 GN=THOC6 PE=1 SV=1	37.5	7.43	THOC6
O75197	Low-density lipoprotein receptor-related protein 5 OS=Homo sapiens OX=9606 GN=LRP5 PE=1 SV=2	179	5.34	LRP5
O75122	CLIP-associating protein 2 OS=Homo sapiens OX=9606 GN=CLASP2 PE=1 SV=3	141	8.4	CLASP2
Q96RL7	Vacuolar protein sorting-associated protein 13A OS=Homo sapiens OX=9606 GN=VPS13A PE=1 SV=2	360	6.33	VPS13A
