# Supplementary material for: Proteomics and Bioinformatics Investigations Link Overexpression of FGF8 and Associated Hub Genes to the Progression of Ovarian Cancer and Poor Prognosis
Source: Biochem Res Int. 2024 Sep 13;2024:4288753. doi: 10.1155/2024/4288753 (PMC11415250; doi:10.1155/2024/4288753)
Supplement: Supplementary Materials — Supplementary Figure 1: venn diagram of proteins identified in FGF8-silenced and FGF8-expressing ovarian cancer cells (SKOV3). Supplementary File 1: proteins identified in ovarian cancer cells (SKOV3). Supplementary File 2: differentially expressed proteins identified by volcano plot analysis. Supplementary File 3: enrichment of GO terms and pathways among downregulated proteins in FGF8-silenced ovarian cancer cells (SKOV3). [file 4288753.f1.zip › Supplementary file 2.pdf]

Supplementary file 2: Differentially expressed proteins identified by volcano plot analysis.

Accession	Description	FC	log2(FC)	raw.pval
D6RBQ6	Ubiquitin carboxyl-terminal hydrolase 17-like protein 17 OS=Homo sapiens OX=9606 GN=USP17L17 PE=3 SV=1	4.9764	2.3151	0.038838
P28799	Progranulin OS=Homo sapiens OX=9606 GN=GRN PE=1 SV=2	2.4522	1.2941	0.016237
P35914	Hydroxymethylglutaryl-CoA lyase, mitochondrial OS=Homo sapiens OX=9606 GN=HMGCL PE=1 SV=2	2.2947	1.1983	0.048561
P04179	Superoxide dismutase [Mn], mitochondrial OS=Homo sapiens OX=9606 GN=SOD2 PE=1 SV=3	2.2345	1.1599	0.041962
P24666	Low molecular weight phosphotyrosine protein phosphatase OS=Homo sapiens OX=9606 GN=ACP1 PE=1 SV=3	0.49877	-1.0036	0.044375
Q96PK6	RNA-binding protein 14 OS=Homo sapiens OX=9606 GN=RBM14 PE=1 SV=2	0.49831	-1.0049	0.03822
P53004	Biliverdin reductase A OS=Homo sapiens OX=9606 GN=BLVRA PE=1 SV=2	0.49771	-1.0066	0.026733
P00403	Cytochrome c oxidase subunit 2 OS=Homo sapiens OX=9606 GN=MT-CO2 PE=1 SV=1	0.49705	-1.0085	0.029561
Q9Y2W1	Thyroid hormone receptor-associated protein 3 OS=Homo sapiens OX=9606 GN=THRAP3 PE=1 SV=2	0.49613	-1.0112	0.006593
P53680	AP-2 complex subunit sigma OS=Homo sapiens OX=9606 GN=AP2S1 PE=1 SV=2	0.49597	-1.0117	0.004391
P29966	Myristoylated alanine-rich C-kinase substrate OS=Homo sapiens OX=9606 GN=MARCKS PE=1 SV=4	0.49589	-1.0119	0.020684
P53007	Tricarboxylate transport protein, mitochondrial OS=Homo sapiens OX=9606 GN=SLC25A1 PE=1 SV=2	0.49571	-1.0124	0.02875
O43414	ERI1 exoribonuclease 3 OS=Homo sapiens OX=9606 GN=ERI3 PE=1 SV=2	0.49566	-1.0126	0.020701
P18124	60S ribosomal protein L7 OS=Homo sapiens OX=9606 GN=RPL7 PE=1 SV=1	0.49383	-1.0179	0.006284
Q8TEM1	Nuclear pore membrane glycoprotein 210 OS=Homo sapiens OX=9606 GN=NUP210 PE=1 SV=3	0.49346	-1.019	0.026533
Q9UNH7	Sorting nexin-6 OS=Homo sapiens OX=9606 GN=SNX6 PE=1 SV=1	0.49334	-1.0194	0.022058
P30050	60S ribosomal protein L12 OS=Homo sapiens OX=9606 GN=RPL12 PE=1 SV=1	0.49284	-1.0208	0.021437
P62851	40S ribosomal protein S25 OS=Homo sapiens OX=9606 GN=RPS25 PE=1 SV=1	0.49211	-1.0229	0.007736
O14949	Cytochrome b-c1 complex subunit 8 OS=Homo sapiens OX=9606 GN=UQCRQ PE=1 SV=4	0.49187	-1.0237	0.018576

Q13642	Four and a half LIM domains protein 1 OS=Homo sapiens OX=9606 GN=FHL1 PE=1 SV=4	0.49148	-1.0248	0.007683
P31948	Stress-induced-phosphoprotein 1 OS=Homo sapiens OX=9606 GN=STIP1 PE=1 SV=1	0.49105	-1.026	0.021957
Q16851	UTP--glucose-1-phosphate uridylyltransferase OS=Homo sapiens OX=9606 GN=UGP2 PE=1 SV=5	0.49056	-1.0275	0.009228
Q15365	Poly(rC)-binding protein 1 OS=Homo sapiens OX=9606 GN=PCBP1 PE=1 SV=2	0.48938	-1.031	0.027055
Q9NTK5	Obg-like ATPase 1 OS=Homo sapiens OX=9606 GN=OLA1 PE=1 SV=2	0.489	-1.0321	0.0276
Q14157	Ubiquitin-associated protein 2-like OS=Homo sapiens OX=9606 GN=UBAP2L PE=1 SV=2	0.4886	-1.0333	0.034682
Q08752	Peptidyl-prolyl cis-trans isomerase D OS=Homo sapiens OX=9606 GN=PPID PE=1 SV=3	0.4876	-1.0362	0.015721
P29317	Ephrin type-A receptor 2 OS=Homo sapiens OX=9606 GN=EPHA2 PE=1 SV=2	0.48726	-1.0372	0.037795
Q07817	Bcl-2-like protein 1 OS=Homo sapiens OX=9606 GN=BCL2L1 PE=1 SV=1	0.48646	-1.0396	0.004895
Q9UM54	Unconventional myosin-VI OS=Homo sapiens OX=9606 GN=MYO6 PE=1 SV=4	0.48616	-1.0405	0.038206
P62312	U6 snRNA-associated Sm-like protein LSm6 OS=Homo sapiens OX=9606 GN=LSM6 PE=1 SV=1	0.48607	-1.0408	0.046036
O75674	TOM1-like protein 1 OS=Homo sapiens OX=9606 GN=TOM1L1 PE=1 SV=2	0.48565	-1.042	0.002722
Q06203	Amidophosphoribosyltransferase OS=Homo sapiens OX=9606 GN=PPAT PE=1 SV=1	0.48489	-1.0443	0.031052
Q9Y6K9	NF-kappa-B essential modulator OS=Homo sapiens OX=9606 GN=IKBKG PE=1 SV=2	0.48489	-1.0443	0.031052
P17987	T-complex protein 1 subunit alpha OS=Homo sapiens OX=9606 GN=TCP1 PE=1 SV=1	0.4844	-1.0457	0.027733
O75083	WD repeat-containing protein 1 OS=Homo sapiens OX=9606 GN=WDR1 PE=1 SV=4	0.48401	-1.0469	0.016987
O95573	Fatty acid CoA ligase Acsl3 OS=Homo sapiens OX=9606 GN=ACSL3 PE=1 SV=3	0.48273	-1.0507	0.043226
Q16537	Serine/threonine-protein phosphatase 2A 56 kDa regulatory subunit epsilon isoform OS=Homo sapiens OX=9606 GN=PPP2R5E PE=1 SV=1	0.48254	-1.0513	0.013766
Q4VC31	Protein MIX23 OS=Homo sapiens OX=9606 GN=MIX23 PE=1 SV=1	0.48195	-1.053	0.042572
Q9Y450	HBS1-like protein OS=Homo sapiens OX=9606 GN=HBS1L PE=1 SV=1	0.48133	-1.0549	0.032885
P08729	Keratin, type II cytoskeletal 7 OS=Homo sapiens OX=9606 GN=KRT7 PE=1 SV=5	0.48116	-1.0554	0.030612

Q9NTJ3	Structural maintenance of chromosomes protein 4 OS=Homo sapiens OX=9606 GN=SMC4 PE=1 SV=2	0.48101	-1.0559	0.020326
Q9P2R7	Succinate--CoA ligase [ADP-forming] subunit beta, mitochondrial OS=Homo sapiens OX=9606 GN=SUCLA2 PE=1 SV=3	0.47998	-1.059	0.02788
Q15131	Cyclin-dependent kinase 10 OS=Homo sapiens OX=9606 GN=CDK10 PE=1 SV=1	0.47982	-1.0594	0.010365
P23368	NAD-dependent malic enzyme, mitochondrial OS=Homo sapiens OX=9606 GN=ME2 PE=1 SV=1	0.4798	-1.0595	0.029998
Q96C19	EF-hand domain-containing protein D2 OS=Homo sapiens OX=9606 GN=EFHD2 PE=1 SV=1	0.47961	-1.0601	0.006443
O43598	2'-deoxynucleoside 5'-phosphate N-hydrolase 1 OS=Homo sapiens OX=9606 GN=DNPH1 PE=1 SV=1	0.47879	-1.0625	0.022467
Q14677	Clathrin interactor 1 OS=Homo sapiens OX=9606 GN=CLINT1 PE=1 SV=1	0.47742	-1.0667	0.029753
P62191	26S proteasome regulatory subunit 4 OS=Homo sapiens OX=9606 GN=PSMC1 PE=1 SV=1	0.4774	-1.0667	0.016457
Q9NYU2	UDP-glucose:glycoprotein glucosyltransferase 1 OS=Homo sapiens OX=9606 GN=UGGT1 PE=1 SV=3	0.47729	-1.0671	0.022566
P62753	40S ribosomal protein S6 OS=Homo sapiens OX=9606 GN=RPS6 PE=1 SV=1	0.47679	-1.0686	0.020715
Q9Y266	Nuclear migration protein nudC OS=Homo sapiens OX=9606 GN=NUDC PE=1 SV=1	0.47553	-1.0724	0.016244
O94874	E3 UFM1-protein ligase 1 OS=Homo sapiens OX=9606 GN=UFL1 PE=1 SV=2	0.47505	-1.0738	0.013825
Q2TAL8	Transcriptional regulator QRICH1 OS=Homo sapiens OX=9606 GN=QRICH1 PE=1 SV=1	0.47486	-1.0744	0.024622
Q6IQ22	Ras-related protein Rab-12 OS=Homo sapiens OX=9606 GN=RAB12 PE=1 SV=3	0.47419	-1.0765	0.010969
O15013	Rho guanine nucleotide exchange factor 10 OS=Homo sapiens OX=9606 GN=ARHGEF10 PE=1 SV=4	0.47395	-1.0772	0.021162
P24534	Elongation factor 1-beta OS=Homo sapiens OX=9606 GN=EEF1B2 PE=1 SV=3	0.47347	-1.0786	0.04824
P49207	60S ribosomal protein L34 OS=Homo sapiens OX=9606 GN=RPL34 PE=1 SV=3	0.47328	-1.0792	0.048155
Q01130	Serine/arginine-rich splicing factor 2 OS=Homo sapiens OX=9606 GN=SRSF2 PE=1 SV=4	0.47317	-1.0796	0.005071
Q9Y6W5	Wiskott-Aldrich syndrome protein family member 2 OS=Homo sapiens OX=9606 GN=WASF2 PE=1 SV=3	0.47288	-1.0805	0.017716
P28482	Mitogen-activated protein kinase 1 OS=Homo sapiens OX=9606 GN=MAPK1 PE=1 SV=3	0.4725	-1.0816	0.015161

O00154	Cytosolic acyl coenzyme A thioester hydrolase OS=Homo sapiens OX=9606 GN=ACOT7 PE=1 SV=3	0.47151	-1.0846	0.045694
Q9NY33	Dipeptidyl peptidase 3 OS=Homo sapiens OX=9606 GN=DPP3 PE=1 SV=2	0.47129	-1.0853	0.035882
P27694	Replication protein A 70 kDa DNA-binding subunit OS=Homo sapiens OX=9606 GN=RPA1 PE=1 SV=2	0.47034	-1.0882	0.047786
Q9ULX9	Transcription factor MafF OS=Homo sapiens OX=9606 GN=MAFF PE=1 SV=2	0.47018	-1.0887	0.041056
P35637	RNA-binding protein FUS OS=Homo sapiens OX=9606 GN=FUS PE=1 SV=1	0.47015	-1.0888	0.014614
Q29RF7	Sister chromatid cohesion protein PDS5 homolog A OS=Homo sapiens OX=9606 GN=PDS5A PE=1 SV=1	0.47015	-1.0888	0.001454
P61586	Transforming protein RhoA OS=Homo sapiens OX=9606 GN=RHOA PE=1 SV=1	0.47002	-1.0892	0.017033
O43760	Synaptogyrin-2 OS=Homo sapiens OX=9606 GN=SYNGR2 PE=1 SV=1	0.46886	-1.0928	0.007891
O75031	Heat shock factor 2-binding protein OS=Homo sapiens OX=9606 GN=HSF2BP PE=1 SV=1	0.46872	-1.0932	0.033224
Q99497	Parkinson disease protein 7 OS=Homo sapiens OX=9606 GN=PARK7 PE=1 SV=2	0.46839	-1.0942	0.010677
Q8TAE8	Growth arrest and DNA damage-inducible proteins-interacting protein 1 OS=Homo sapiens OX=9606 GN=GADD45GIP1 PE=1 SV=1	0.46833	-1.0944	0.009436
Q16629	Serine/arginine-rich splicing factor 7 OS=Homo sapiens OX=9606 GN=SRSF7 PE=1 SV=1	0.46813	-1.095	0.020871
P63220	40S ribosomal protein S21 OS=Homo sapiens OX=9606 GN=RPS21 PE=1 SV=1	0.46766	-1.0965	0.003584
P52943	Cysteine-rich protein 2 OS=Homo sapiens OX=9606 GN=CRIP2 PE=1 SV=1	0.46751	-1.0969	0.02132
Q5VTU8	ATP synthase subunit epsilon-like protein, mitochondrial OS=Homo sapiens OX=9606 GN=ATP5F1EP2 PE=1 SV=1	0.46669	-1.0995	0.000614
P42285	Exosome RNA helicase MTR4 OS=Homo sapiens OX=9606 GN=MTREX PE=1 SV=3	0.46662	-1.0997	0.02793
Q14919	Dr1-associated corepressor OS=Homo sapiens OX=9606 GN=DRAP1 PE=1 SV=3	0.46618	-1.101	0.026348
P30876	DNA-directed RNA polymerase II subunit RPB2 OS=Homo sapiens OX=9606 GN=POLR2B PE=1 SV=1	0.46582	-1.1022	0.008051
P61513	60S ribosomal protein L37a OS=Homo sapiens OX=9606 GN=RPL37A PE=1 SV=2	0.46545	-1.1033	0.012261
Q9BXJ9	N-alpha-acetyltransferase 15, NatA auxiliary subunit OS=Homo sapiens OX=9606 GN=NAA15 PE=1 SV=1	0.46535	-1.1036	0.016267

Q16643	Drebrin OS=Homo sapiens OX=9606 GN=DBN1 PE=1 SV=4	0.46505	-1.1045	0.048195
Q13523	Serine/threonine-protein kinase PRP4 homolog OS=Homo sapiens OX=9606 GN=PRPF4B PE=1 SV=3	0.46455	-1.1061	0.049781
P05198	Eukaryotic translation initiation factor 2 subunit 1 OS=Homo sapiens OX=9606 GN=EIF2S1 PE=1 SV=3	0.4644	-1.1066	0.015232
Q8IVF2	Protein AHNAK2 OS=Homo sapiens OX=9606 GN=AHNAK2 PE=1 SV=2	0.46389	-1.1082	0.010808
P12004	Proliferating cell nuclear antigen OS=Homo sapiens OX=9606 GN=PCNA PE=1 SV=1	0.46366	-1.1088	0.04431
P11387	DNA topoisomerase 1 OS=Homo sapiens OX=9606 GN=TOP1 PE=1 SV=2	0.46322	-1.1102	0.048341
P20042	Eukaryotic translation initiation factor 2 subunit 2 OS=Homo sapiens OX=9606 GN=EIF2S2 PE=1 SV=2	0.46267	-1.1119	0.0187
P35749	Myosin-11 OS=Homo sapiens OX=9606 GN=MYH11 PE=1 SV=3	0.46261	-1.1121	0.018708
O14548	Cytochrome c oxidase subunit 7A-related protein, mitochondrial OS=Homo sapiens OX=9606 GN=COX7A2L PE=1 SV=2	0.46241	-1.1127	0.00468
P06396	Gelsolin OS=Homo sapiens OX=9606 GN=GSN PE=1 SV=1	0.4617	-1.115	0.025706
Q8IWB7	WD repeat and FYVE domain-containing protein 1 OS=Homo sapiens OX=9606 GN=WDFY1 PE=1 SV=1	0.4617	-1.115	0.02765
Q13868	Exosome complex component RRP4 OS=Homo sapiens OX=9606 GN=EXOSC2 PE=1 SV=2	0.46145	-1.1158	0.01911
Q9NUP9	Protein lin-7 homolog C OS=Homo sapiens OX=9606 GN=LIN7C PE=1 SV=1	0.46135	-1.1161	0.019837
P25786	Proteasome subunit alpha type-1 OS=Homo sapiens OX=9606 GN=PSMA1 PE=1 SV=1	0.46022	-1.1196	0.049891
O14929	Histone acetyltransferase type B catalytic subunit OS=Homo sapiens OX=9606 GN=HAT1 PE=1 SV=1	0.45965	-1.1214	0.048697
Q9P2E9	Ribosome-binding protein 1 OS=Homo sapiens OX=9606 GN=RRBP1 PE=1 SV=5	0.45849	-1.125	0.004243
P35080	Profilin-2 OS=Homo sapiens OX=9606 GN=PFN2 PE=1 SV=3	0.45846	-1.1251	0.034993
Q9HCU4	Cadherin EGF LAG seven-pass G-type receptor 2 OS=Homo sapiens OX=9606 GN=CELSR2 PE=1 SV=1	0.45769	-1.1275	0.008012
P30740	Leukocyte elastase inhibitor OS=Homo sapiens OX=9606 GN=SERPINB1 PE=1 SV=1	0.45684	-1.1302	0.00894
P11586	C-1-tetrahydrofolate synthase, cytoplasmic OS=Homo sapiens OX=9606 GN=MTHFD1 PE=1 SV=4	0.45644	-1.1315	0.003012
P0DP25	Calmodulin-3 OS=Homo sapiens OX=9606 GN=CALM3 PE=1 SV=1	0.45606	-1.1327	0.019519

Q9NX63	MICOS complex subunit MIC19 OS=Homo sapiens OX=9606 GN=CHCHD3 PE=1 SV=1	0.45558	-1.1342	0.047766
Q13907	Isopentenyl-diphosphate Delta-isomerase 1 OS=Homo sapiens OX=9606 GN=IDI1 PE=1 SV=2	0.45488	-1.1364	0.04285
Q13952	Nuclear transcription factor Y subunit gamma OS=Homo sapiens OX=9606 GN=NFYC PE=1 SV=3	0.45481	-1.1367	0.030431
P35221	Catenin alpha-1 OS=Homo sapiens OX=9606 GN=CTNNA1 PE=1 SV=1	0.45457	-1.1374	0.037519
O95831	Apoptosis-inducing factor 1, mitochondrial OS=Homo sapiens OX=9606 GN=AIFM1 PE=1 SV=1	0.45403	-1.1391	0.023724
P27816	Microtubule-associated protein 4 OS=Homo sapiens OX=9606 GN=MAP4 PE=1 SV=3	0.45372	-1.1401	0.003953
A6NDG6	Glycerol-3-phosphate phosphatase OS=Homo sapiens OX=9606 GN=PGP PE=1 SV=1	0.45365	-1.1403	0.01419
O14745	Na(+)/H(+) exchange regulatory cofactor NHE-RF1 OS=Homo sapiens OX=9606 GN=SLC9A3R1 PE=1 SV=4	0.45318	-1.1419	0.036338
P25789	Proteasome subunit alpha type-4 OS=Homo sapiens OX=9606 GN=PSMA4 PE=1 SV=1	0.45245	-1.1442	0.049132
P49023	Paxillin OS=Homo sapiens OX=9606 GN=PXN PE=1 SV=3	0.45206	-1.1454	0.001599
P61088	Ubiquitin-conjugating enzyme E2 N OS=Homo sapiens OX=9606 GN=UBE2N PE=1 SV=1	0.45188	-1.146	0.036258
Q07020	60S ribosomal protein L18 OS=Homo sapiens OX=9606 GN=RPL18 PE=1 SV=2	0.45094	-1.149	0.002739
P49189	4-trimethylaminobutyraldehyde dehydrogenase OS=Homo sapiens OX=9606 GN=ALDH9A1 PE=1 SV=3	0.45074	-1.1496	0.034849
Q9P2K8	eIF-2-alpha kinase GCN2 OS=Homo sapiens OX=9606 GN=EIF2AK4 PE=1 SV=3	0.44972	-1.1529	0.011327
P28066	Proteasome subunit alpha type-5 OS=Homo sapiens OX=9606 GN=PSMA5 PE=1 SV=3	0.44951	-1.1536	0.002056
P09543	2',3'-cyclic-nucleotide 3'-phosphodiesterase OS=Homo sapiens OX=9606 GN=CNP PE=1 SV=2	0.44922	-1.1545	0.008513
Q15819	Ubiquitin-conjugating enzyme E2 variant 2 OS=Homo sapiens OX=9606 GN=UBE2V2 PE=1 SV=4	0.44912	-1.1548	0.007626
P54577	Tyrosine--tRNA ligase, cytoplasmic OS=Homo sapiens OX=9606 GN=YARS1 PE=1 SV=4	0.44864	-1.1564	0.012562
Q14696	LRP chaperone MESD OS=Homo sapiens OX=9606 GN=MESD PE=1 SV=2	0.4483	-1.1575	0.030498
P23588	Eukaryotic translation initiation factor 4B OS=Homo sapiens OX=9606 GN=EIF4B PE=1 SV=2	0.44788	-1.1588	0.018624

Q15370	Elongin-B OS=Homo sapiens OX=9606 GN=ELOB PE=1 SV=1	0.44593	-1.1651	0.003054
Q14498	RNA-binding protein 39 OS=Homo sapiens OX=9606 GN=RBM39 PE=1 SV=2	0.44491	-1.1684	0.003645
P08754	Guanine nucleotide-binding protein G(i) subunit alpha-3 OS=Homo sapiens OX=9606 GN=GNAI3 PE=1 SV=3	0.44436	-1.1702	0.003057
Q9Y3A5	Ribosome maturation protein SBDS OS=Homo sapiens OX=9606 GN=SBDS PE=1 SV=4	0.44398	-1.1714	0.007652
Q15417	Calponin-3 OS=Homo sapiens OX=9606 GN=CNN3 PE=1 SV=1	0.44335	-1.1735	0.007698
O00244	Copper transport protein ATOX1 OS=Homo sapiens OX=9606 GN=ATOX1 PE=1 SV=1	0.44319	-1.174	0.004564
P55809	Succinyl-CoA:3-ketoacid coenzyme A transferase 1, mitochondrial OS=Homo sapiens OX=9606 GN=OXCT1 PE=1 SV=1	0.44295	-1.1748	0.019223
Q9NXA8	NAD-dependent protein deacylase sirtuin-5, mitochondrial OS=Homo sapiens OX=9606 GN=SIRT5 PE=1 SV=2	0.44103	-1.181	0.006549
Q8TCT9	Minor histocompatibility antigen H13 OS=Homo sapiens OX=9606 GN=HM13 PE=1 SV=1	0.44102	-1.1811	0.037728
Q9BVK6	Transmembrane emp24 domain-containing protein 9 OS=Homo sapiens OX=9606 GN=TMED9 PE=1 SV=2	0.44015	-1.1839	0.038296
Q13637	Ras-related protein Rab-32 OS=Homo sapiens OX=9606 GN=RAB32 PE=1 SV=3	0.43942	-1.1863	0.030245
O75381	Peroxisomal membrane protein PEX14 OS=Homo sapiens OX=9606 GN=PEX14 PE=1 SV=1	0.43918	-1.1871	0.003354
P09622	Dihydrolipoyl dehydrogenase, mitochondrial OS=Homo sapiens OX=9606 GN=DLD PE=1 SV=2	0.43841	-1.1896	0.017952
P46063	ATP-dependent DNA helicase Q1 OS=Homo sapiens OX=9606 GN=RECQL PE=1 SV=3	0.43818	-1.1904	0.012268
P09669	Cytochrome c oxidase subunit 6C OS=Homo sapiens OX=9606 GN=COX6C PE=1 SV=2	0.43798	-1.1911	0.020504
Q9Y3C8	Ubiquitin-fold modifier-conjugating enzyme 1 OS=Homo sapiens OX=9606 GN=UFC1 PE=1 SV=3	0.43797	-1.1911	0.035078
Q9UL25	Ras-related protein Rab-21 OS=Homo sapiens OX=9606 GN=RAB21 PE=1 SV=3	0.43713	-1.1939	0.036807
P48643	T-complex protein 1 subunit epsilon OS=Homo sapiens OX=9606 GN=CCT5 PE=1 SV=1	0.43637	-1.1964	0.009352
Q9UMY4	Sorting nexin-12 OS=Homo sapiens OX=9606 GN=SNX12 PE=1 SV=4	0.43504	-1.2008	0.01613
Q86UP2	Kinectin OS=Homo sapiens OX=9606 GN=KTN1 PE=1 SV=1	0.43499	-1.2009	0.025522

P62140	Serine/threonine-protein phosphatase PP1-beta catalytic subunit OS=Homo sapiens OX=9606 GN=PPP1CB PE=1 SV=3	0.43495	-1.2011	0.020487
O96000	NADH dehydrogenase [ubiquinone] 1 beta subcomplex subunit 10 OS=Homo sapiens OX=9606 GN=NDUFB10 PE=1 SV=3	0.43468	-1.202	0.020055
Q8NE71	ATP-binding cassette sub-family F member 1 OS=Homo sapiens OX=9606 GN=ABCF1 PE=1 SV=2	0.4329	-1.2079	0.042609
A6NHR9	Structural maintenance of chromosomes flexible hinge domain-containing protein 1 OS=Homo sapiens OX=9606 GN=SMCHD1 PE=1 SV=2	0.43284	-1.2081	0.00835
Q92609	TBC1 domain family member 5 OS=Homo sapiens OX=9606 GN=TBC1D5 PE=1 SV=1	0.43195	-1.2111	0.013889
P51452	Dual specificity protein phosphatase 3 OS=Homo sapiens OX=9606 GN=DUSP3 PE=1 SV=1	0.43179	-1.2116	0.031386
Q32MZ4	Leucine-rich repeat flightless-interacting protein 1 OS=Homo sapiens OX=9606 GN=LRRFIP1 PE=1 SV=2	0.43103	-1.2141	0.043416
Q9H2M9	Rab3 GTPase-activating protein non-catalytic subunit OS=Homo sapiens OX=9606 GN=RAB3GAP2 PE=1 SV=1	0.43051	-1.2159	0.016528
Q969V3	Nicalin OS=Homo sapiens OX=9606 GN=NCLN PE=1 SV=2	0.42975	-1.2184	0.047322
P18077	60S ribosomal protein L35a OS=Homo sapiens OX=9606 GN=RPL35A PE=1 SV=2	0.4283	-1.2233	0.006273
Q96CM8	Medium-chain acyl-CoA ligase ACSF2, mitochondrial OS=Homo sapiens OX=9606 GN=ACSF2 PE=1 SV=2	0.42721	-1.227	0.027906
Q08722	Leukocyte surface antigen CD47 OS=Homo sapiens OX=9606 GN=CD47 PE=1 SV=1	0.42713	-1.2272	0.024983
P78345	Ribonuclease P protein subunit p38 OS=Homo sapiens OX=9606 GN=RPP38 PE=1 SV=2	0.42696	-1.2278	0.011906
P21964	Catechol O-methyltransferase OS=Homo sapiens OX=9606 GN=COMT PE=1 SV=2	0.42695	-1.2279	0.013811
Q05682	Caldesmon OS=Homo sapiens OX=9606 GN=CALD1 PE=1 SV=3	0.42636	-1.2299	0.01808
Q96BJ3	Axin interactor, dorsalization-associated protein OS=Homo sapiens OX=9606 GN=AIDA PE=1 SV=1	0.42557	-1.2325	0.008224
Q9BTZ2	Dehydrogenase/reductase SDR family member 4 OS=Homo sapiens OX=9606 GN=DHRS4 PE=1 SV=3	0.42508	-1.2342	0.012372
O60841	Eukaryotic translation initiation factor 5B OS=Homo sapiens OX=9606 GN=EIF5B PE=1 SV=4	0.42456	-1.236	0.03158
P50402	Emerin OS=Homo sapiens OX=9606 GN=EMD PE=1 SV=1	0.42414	-1.2374	0.033708

P60900	Proteasome subunit alpha type-6 OS=Homo sapiens OX=9606 GN=PSMA6 PE=1 SV=1	0.42316	-1.2407	0.03325
Q13561	Dynactin subunit 2 OS=Homo sapiens OX=9606 GN=DCTN2 PE=1 SV=4	0.42315	-1.2407	0.037199
P25325	3-mercaptopyruvate sulfurtransferase OS=Homo sapiens OX=9606 GN=MPST PE=1 SV=3	0.42295	-1.2415	0.01028
Q92896	Golgi apparatus protein 1 OS=Homo sapiens OX=9606 GN=GLG1 PE=1 SV=2	0.42211	-1.2443	0.024946
P61247	40S ribosomal protein S3a OS=Homo sapiens OX=9606 GN=RPS3A PE=1 SV=2	0.42202	-1.2446	0.002039
P49773	Adenosine 5'-monophosphoramidase HINT1 OS=Homo sapiens OX=9606 GN=HINT1 PE=1 SV=2	0.4212	-1.2474	0.030456
P46060	Ran GTPase-activating protein 1 OS=Homo sapiens OX=9606 GN=RANGAP1 PE=1 SV=1	0.41974	-1.2524	0.048733
P35249	Replication factor C subunit 4 OS=Homo sapiens OX=9606 GN=RFC4 PE=1 SV=2	0.41937	-1.2537	0.006123
Q8WXX5	DnaJ homolog subfamily C member 9 OS=Homo sapiens OX=9606 GN=DNAJC9 PE=1 SV=1	0.41908	-1.2547	0.012901
Q04760	Lactoylglutathione lyase OS=Homo sapiens OX=9606 GN=GLO1 PE=1 SV=4	0.41782	-1.2591	0.043938
Q96IX5	ATP synthase membrane subunit K, mitochondrial OS=Homo sapiens OX=9606 GN=ATP5MK PE=1 SV=1	0.41775	-1.2593	0.009299
Q96GX9	Methylthioribulose-1-phosphate dehydratase OS=Homo sapiens OX=9606 GN=APIP PE=1 SV=1	0.41741	-1.2605	0.030536
O14744	Protein arginine N-methyltransferase 5 OS=Homo sapiens OX=9606 GN=PRMT5 PE=1 SV=4	0.41711	-1.2615	0.003246
P30626	Sorcin OS=Homo sapiens OX=9606 GN=SRI PE=1 SV=1	0.41668	-1.263	0.001154
Q8TD19	Serine/threonine-protein kinase Nek9 OS=Homo sapiens OX=9606 GN=NEK9 PE=1 SV=2	0.416	-1.2653	0.018308
P49321	Nuclear autoantigenic sperm protein OS=Homo sapiens OX=9606 GN=NASP PE=1 SV=2	0.41513	-1.2684	0.04504
P26373	60S ribosomal protein L13 OS=Homo sapiens OX=9606 GN=RPL13 PE=1 SV=4	0.41491	-1.2691	0.002297
P00441	Superoxide dismutase [Cu-Zn] OS=Homo sapiens OX=9606 GN=SOD1 PE=1 SV=2	0.41431	-1.2712	0.035985
P15170	Eukaryotic peptide chain release factor GTP-binding subunit ERF3A OS=Homo sapiens OX=9606 GN=GSPT1 PE=1 SV=1	0.41402	-1.2722	0.004778
P15880	40S ribosomal protein S2 OS=Homo sapiens OX=9606 GN=RPS2 PE=1 SV=2	0.4139	-1.2726	0.007407
Q9NTX5	Ethylmalonyl-CoA decarboxylase OS=Homo sapiens OX=9606 GN=ECHDC1 PE=1 SV=2	0.41299	-1.2758	0.024838

O14828	Secretory carrier-associated membrane protein 3 OS=Homo sapiens OX=9606 GN=SCAMP3 PE=1 SV=3	0.41247	-1.2776	0.026354
Q96HY6	DDRGK domain-containing protein 1 OS=Homo sapiens OX=9606 GN=DDRGK1 PE=1 SV=2	0.41203	-1.2792	0.020607
P50897	Palmitoyl-protein thioesterase 1 OS=Homo sapiens OX=9606 GN=PPT1 PE=1 SV=1	0.41099	-1.2828	0.001798
P14927	Cytochrome b-c1 complex subunit 7 OS=Homo sapiens OX=9606 GN=UQCRB PE=1 SV=2	0.41057	-1.2843	0.037519
P28838	Cytosol aminopeptidase OS=Homo sapiens OX=9606 GN=LAP3 PE=1 SV=3	0.41046	-1.2847	0.021146
P26640	Valine--tRNA ligase OS=Homo sapiens OX=9606 GN=VARS1 PE=1 SV=4	0.4103	-1.2852	0.012726
Q92973	Transportin-1 OS=Homo sapiens OX=9606 GN=TNPO1 PE=1 SV=2	0.41028	-1.2853	0.008096
P02533	Keratin, type I cytoskeletal 14 OS=Homo sapiens OX=9606 GN=KRT14 PE=1 SV=4	0.41008	-1.286	0.015616
Q9BYD6	39S ribosomal protein L1, mitochondrial OS=Homo sapiens OX=9606 GN=MRPL1 PE=1 SV=2	0.40995	-1.2865	0.018332
Q9H2G2	STE20-like serine/threonine-protein kinase OS=Homo sapiens OX=9606 GN=SLK PE=1 SV=1	0.40916	-1.2893	0.006432
P42677	40S ribosomal protein S27 OS=Homo sapiens OX=9606 GN=RPS27 PE=1 SV=3	0.40875	-1.2907	0.031081
O14818	Proteasome subunit alpha type-7 OS=Homo sapiens OX=9606 GN=PSMA7 PE=1 SV=1	0.40865	-1.291	0.026277
O14964	Hepatocyte growth factor-regulated tyrosine kinase substrate OS=Homo sapiens OX=9606 GN=HGS PE=1 SV=1	0.40806	-1.2932	0.007058
P09382	Galectin-1 OS=Homo sapiens OX=9606 GN=LGALS1 PE=1 SV=2	0.40785	-1.2939	0.004698
Q9Y3E7	Charged multivesicular body protein 3 OS=Homo sapiens OX=9606 GN=CHMP3 PE=1 SV=3	0.40747	-1.2952	0.045485
Q9Y281	Cofilin-2 OS=Homo sapiens OX=9606 GN=CFL2 PE=1 SV=1	0.40699	-1.2969	0.008562
Q92598	Heat shock protein 105 kDa OS=Homo sapiens OX=9606 GN=HSPH1 PE=1 SV=1	0.40695	-1.2971	0.017252
Q6IBS0	Twinfilin-2 OS=Homo sapiens OX=9606 GN=TWF2 PE=1 SV=2	0.40631	-1.2993	0.013295
P35222	Catenin beta-1 OS=Homo sapiens OX=9606 GN=CTNNB1 PE=1 SV=1	0.40618	-1.2998	0.020303
P78344	Eukaryotic translation initiation factor 4 gamma 2 OS=Homo sapiens OX=9606 GN=EIF4G2 PE=1 SV=1	0.40611	-1.3001	0.031075
Q02878	60S ribosomal protein L6 OS=Homo sapiens OX=9606 GN=RPL6 PE=1 SV=3	0.4056	-1.3019	0.003803

O43670	BUB3-interacting and GLEBS motif-containing protein ZNF207 OS=Homo sapiens OX=9606 GN=ZNF207 PE=1 SV=1	0.40548	-1.3023	0.018995
P50148	Guanine nucleotide-binding protein G(q) subunit alpha OS=Homo sapiens OX=9606 GN=GNAQ PE=1 SV=4	0.40535	-1.3028	0.043877
Q99426	Tubulin-folding cofactor B OS=Homo sapiens OX=9606 GN=TBCB PE=1 SV=2	0.40532	-1.3028	0.026297
O43175	D-3-phosphoglycerate dehydrogenase OS=Homo sapiens OX=9606 GN=PHGDH PE=1 SV=4	0.40528	-1.303	0.047443
P17174	Aspartate aminotransferase, cytoplasmic OS=Homo sapiens OX=9606 GN=GOT1 PE=1 SV=3	0.40339	-1.3098	0.037107
Q8IX01	SURP and G-patch domain-containing protein 2 OS=Homo sapiens OX=9606 GN=SUGP2 PE=1 SV=2	0.4027	-1.3122	0.006548
P62877	E3 ubiquitin-protein ligase RBX1 OS=Homo sapiens OX=9606 GN=RBX1 PE=1 SV=1	0.40258	-1.3127	0.000416
Q9UH99	SUN domain-containing protein 2 OS=Homo sapiens OX=9606 GN=SUN2 PE=1 SV=3	0.40193	-1.315	0.017095
Q13813	Spectrin alpha chain, non-erythrocytic 1 OS=Homo sapiens OX=9606 GN=SPTAN1 PE=1 SV=3	0.40162	-1.3161	0.01555
Q9H6S3	Epidermal growth factor receptor kinase substrate 8-like protein 2 OS=Homo sapiens OX=9606 GN=EPS8L2 PE=1 SV=2	0.40085	-1.3189	0.030585
Q9NW64	Pre-mRNA-splicing factor RBM22 OS=Homo sapiens OX=9606 GN=RBM22 PE=1 SV=1	0.40024	-1.321	0.010435
Q9BYC5	Alpha-(1,6)-fucosyltransferase OS=Homo sapiens OX=9606 GN=FUT8 PE=1 SV=2	0.40022	-1.3211	0.02897
O15533	Tapasin OS=Homo sapiens OX=9606 GN=TAPBP PE=1 SV=1	0.39932	-1.3244	0.010921
P28072	Proteasome subunit beta type-6 OS=Homo sapiens OX=9606 GN=PSMB6 PE=1 SV=4	0.39922	-1.3247	0.031619
P07686	Beta-hexosaminidase subunit beta OS=Homo sapiens OX=9606 GN=HEXB PE=1 SV=3	0.39908	-1.3253	0.025636
O00193	Small acidic protein OS=Homo sapiens OX=9606 GN=SMAP PE=1 SV=1	0.39897	-1.3257	0.006924
P34932	Heat shock 70 kDa protein 4 OS=Homo sapiens OX=9606 GN=HSPA4 PE=1 SV=4	0.39896	-1.3257	0.030318
Q8IYI6	Exocyst complex component 8 OS=Homo sapiens OX=9606 GN=EXOC8 PE=1 SV=2	0.39858	-1.3271	0.026246
P23193	Transcription elongation factor A protein 1 OS=Homo sapiens OX=9606 GN=TCEA1 PE=1 SV=2	0.39775	-1.3301	0.029321
Q7Z4H3	5'-deoxynucleotidase HDDC2 OS=Homo sapiens OX=9606 GN=HDDC2 PE=1 SV=1	0.39746	-1.3311	0.019616
Q86X76	Deaminated glutathione amidase OS=Homo sapiens OX=9606 GN=NIT1 PE=1 SV=2	0.39734	-1.3316	0.023597

Q03135	Caveolin-1 OS=Homo sapiens OX=9606 GN=CAV1 PE=1 SV=4	0.39682	-1.3334	0.012075
Q13404	Ubiquitin-conjugating enzyme E2 variant 1 OS=Homo sapiens OX=9606 GN=UBE2V1 PE=1 SV=2	0.39609	-1.3361	0.004775
Q9H446	RWD domain-containing protein 1 OS=Homo sapiens OX=9606 GN=RWDD1 PE=1 SV=1	0.3958	-1.3371	0.013253
P43487	Ran-specific GTPase-activating protein OS=Homo sapiens OX=9606 GN=RANBP1 PE=1 SV=1	0.39501	-1.34	0.002605
Q9Y512	Sorting and assembly machinery component 50 homolog OS=Homo sapiens OX=9606 GN=SAMM50 PE=1 SV=3	0.39475	-1.341	0.018318
O43684	Mitotic checkpoint protein BUB3 OS=Homo sapiens OX=9606 GN=BUB3 PE=1 SV=1	0.39445	-1.3421	0.015758
P46777	60S ribosomal protein L5 OS=Homo sapiens OX=9606 GN=RPL5 PE=1 SV=3	0.39281	-1.3481	0.037733
P12268	Inosine-5'-monophosphate dehydrogenase 2 OS=Homo sapiens OX=9606 GN=IMPDH2 PE=1 SV=2	0.39228	-1.35	0.027597
O43290	U4/U6.U5 tri-snRNP-associated protein 1 OS=Homo sapiens OX=9606 GN=SART1 PE=1 SV=1	0.39213	-1.3506	0.006144
P26440	Isovaleryl-CoA dehydrogenase, mitochondrial OS=Homo sapiens OX=9606 GN=IVD PE=1 SV=2	0.39207	-1.3508	0.043358
Q8WWM7	Ataxin-2-like protein OS=Homo sapiens OX=9606 GN=ATXN2L PE=1 SV=2	0.39097	-1.3549	0.042082
O94903	Pyridoxal phosphate homeostasis protein OS=Homo sapiens OX=9606 GN=PLPBP PE=1 SV=1	0.39092	-1.3551	0.004649
Q01082	Spectrin beta chain, non-erythrocytic 1 OS=Homo sapiens OX=9606 GN=SPTBN1 PE=1 SV=2	0.39077	-1.3556	0.005912
Q8TCE6	DENN domain-containing protein 10 OS=Homo sapiens OX=9606 GN=DENND10 PE=1 SV=1	0.39037	-1.3571	0.017841
Q8N1F7	Nuclear pore complex protein Nup93 OS=Homo sapiens OX=9606 GN=NUP93 PE=1 SV=2	0.38984	-1.3591	0.012255
O75400	Pre-mRNA-processing factor 40 homolog A OS=Homo sapiens OX=9606 GN=PRPF40A PE=1 SV=2	0.38972	-1.3595	0.009418
P49588	Alanine--tRNA ligase, cytoplasmic OS=Homo sapiens OX=9606 GN=AARS1 PE=1 SV=2	0.3892	-1.3614	0.007323
P21399	Cytoplasmic aconitate hydratase OS=Homo sapiens OX=9606 GN=ACO1 PE=1 SV=3	0.38878	-1.363	0.029486
O00203	AP-3 complex subunit beta-1 OS=Homo sapiens OX=9606 GN=AP3B1 PE=1 SV=3	0.38711	-1.3692	0.00363

Q8N0X7	Spartin OS=Homo sapiens OX=9606 GN=SPART PE=1 SV=1	0.38704	-1.3694	0.031052
Q9BQ39	ATP-dependent RNA helicase DDX50 OS=Homo sapiens OX=9606 GN=DDX50 PE=1 SV=1	0.3865	-1.3714	0.000615
P42167	Lamina-associated polypeptide 2, isoforms beta/gamma OS=Homo sapiens OX=9606 GN=TMPO PE=1 SV=2	0.38595	-1.3735	0.016201
P49721	Proteasome subunit beta type-2 OS=Homo sapiens OX=9606 GN=PSMB2 PE=1 SV=1	0.38567	-1.3745	0.025943
Q9Y3B7	39S ribosomal protein L11, mitochondrial OS=Homo sapiens OX=9606 GN=MRPL11 PE=1 SV=1	0.38566	-1.3746	0.030948
Q13136	Liprin-alpha-1 OS=Homo sapiens OX=9606 GN=PPFIA1 PE=1 SV=1	0.3849	-1.3774	0.014176
Q9UEY8	Gamma-adducin OS=Homo sapiens OX=9606 GN=ADD3 PE=1 SV=1	0.38448	-1.379	0.003597
P16403	Histone H1.2 OS=Homo sapiens OX=9606 GN=H1-2 PE=1 SV=2	0.38444	-1.3792	0.021174
Q92614	Unconventional myosin-XVIIIa OS=Homo sapiens OX=9606 GN=MYO18A PE=1 SV=3	0.38411	-1.3804	0.01525
Q08209	Serine/threonine-protein phosphatase 2B catalytic subunit alpha isoform OS=Homo sapiens OX=9606 GN=PPP3CA PE=1 SV=1	0.38327	-1.3836	0.000862
Q9BRQ6	MICOS complex subunit MIC25 OS=Homo sapiens OX=9606 GN=CHCHD6 PE=1 SV=1	0.38313	-1.3841	0.024989
Q9NYL9	Tropomodulin-3 OS=Homo sapiens OX=9606 GN=TMOD3 PE=1 SV=1	0.38256	-1.3862	0.006837
Q9Y3Z3	Deoxynucleoside triphosphate triphosphohydrolase SAMHD1 OS=Homo sapiens OX=9606 GN=SAMHD1 PE=1 SV=2	0.38221	-1.3876	0.020719
P21333	Filamin-A OS=Homo sapiens OX=9606 GN=FLNA PE=1 SV=4	0.38157	-1.39	0.01343
Q12905	Interleukin enhancer-binding factor 2 OS=Homo sapiens OX=9606 GN=ILF2 PE=1 SV=2	0.38134	-1.3909	0.029077
O14974	Protein phosphatase 1 regulatory subunit 12A OS=Homo sapiens OX=9606 GN=PPP1R12A PE=1 SV=1	0.38127	-1.3911	0.017607
Q9Y3A3	MOB-like protein phocein OS=Homo sapiens OX=9606 GN=MOB4 PE=1 SV=1	0.37937	-1.3983	0.023953
Q9UBT2	SUMO-activating enzyme subunit 2 OS=Homo sapiens OX=9606 GN=UBA2 PE=1 SV=2	0.37883	-1.4004	0.010495
Q15388	Mitochondrial import receptor subunit TOM20 homolog OS=Homo sapiens OX=9606 GN=TOMM20 PE=1 SV=1	0.3788	-1.4005	0.007849
Q9H3U1	Protein unc-45 homolog A OS=Homo sapiens OX=9606 GN=UNC45A PE=1 SV=1	0.37864	-1.4011	0.003894
O00712	Nuclear factor 1 B-type OS=Homo sapiens OX=9606 GN=NFIB PE=1 SV=2	0.37808	-1.4032	0.01597

P55010	Eukaryotic translation initiation factor 5 OS=Homo sapiens OX=9606 GN=EIF5 PE=1 SV=2	0.37791	-1.4039	0.028332
Q8IWS0	PHD finger protein 6 OS=Homo sapiens OX=9606 GN=PHF6 PE=1 SV=1	0.37733	-1.4061	0.015114
Q13308	Inactive tyrosine-protein kinase 7 OS=Homo sapiens OX=9606 GN=PTK7 PE=1 SV=2	0.37698	-1.4074	0.026824
O14936	Peripheral plasma membrane protein CASK OS=Homo sapiens OX=9606 GN=CASK PE=1 SV=3	0.37679	-1.4082	0.028134
Q8TCG1	Protein CIP2A OS=Homo sapiens OX=9606 GN=CIP2A PE=1 SV=2	0.37619	-1.4105	0.014207
Q12906	Interleukin enhancer-binding factor 3 OS=Homo sapiens OX=9606 GN=ILF3 PE=1 SV=3	0.37595	-1.4114	0.004824
Q9BZZ5	Apoptosis inhibitor 5 OS=Homo sapiens OX=9606 GN=API5 PE=1 SV=3	0.37562	-1.4126	0.003054
O14773	Tripeptidyl-peptidase 1 OS=Homo sapiens OX=9606 GN=TPP1 PE=1 SV=2	0.37517	-1.4144	0.014957
O95810	Caveolae-associated protein 2 OS=Homo sapiens OX=9606 GN=CAVIN2 PE=1 SV=3	0.37378	-1.4198	0.015935
P80303	Nucleobindin-2 OS=Homo sapiens OX=9606 GN=NUCB2 PE=1 SV=3	0.3713	-1.4293	0.036806
P28074	Proteasome subunit beta type-5 OS=Homo sapiens OX=9606 GN=PSMB5 PE=1 SV=3	0.37089	-1.431	0.042299
P49959	Double-strand break repair protein MRE11 OS=Homo sapiens OX=9606 GN=MRE11 PE=1 SV=3	0.36997	-1.4345	0.007407
O95817	BAG family molecular chaperone regulator 3 OS=Homo sapiens OX=9606 GN=BAG3 PE=1 SV=3	0.36994	-1.4347	0.000224
O00592	Podocalyxin OS=Homo sapiens OX=9606 GN=PODXL PE=1 SV=2	0.36979	-1.4352	0.001202
Q9UKG1	DCC-interacting protein 13-alpha OS=Homo sapiens OX=9606 GN=APPL1 PE=1 SV=1	0.36835	-1.4409	0.00273
P41227	N-alpha-acetyltransferase 10 OS=Homo sapiens OX=9606 GN=NAA10 PE=1 SV=1	0.36782	-1.4429	0.003275
P67809	Y-box-binding protein 1 OS=Homo sapiens OX=9606 GN=YBX1 PE=1 SV=3	0.36692	-1.4465	0.001176
Q96GK7	Fumarylacetoacetate hydrolase domain-containing protein 2A OS=Homo sapiens OX=9606 GN=FAHD2A PE=1 SV=1	0.36678	-1.447	0.037397
Q96RQ3	Methylcrotonoyl-CoA carboxylase subunit alpha, mitochondrial OS=Homo sapiens OX=9606 GN=MCCC1 PE=1 SV=3	0.36672	-1.4472	0.013854
P56199	Integrin alpha-1 OS=Homo sapiens OX=9606 GN=ITGA1 PE=1 SV=2	0.36582	-1.4508	0.012123
Q9BTT0	Acidic leucine-rich nuclear phosphoprotein 32 family member E OS=Homo sapiens OX=9606 GN=ANP32E PE=1 SV=1	0.36526	-1.453	0.022195

Q12972	Nuclear inhibitor of protein phosphatase 1 OS=Homo sapiens OX=9606 GN=PPP1R8 PE=1 SV=2	0.36496	-1.4542	0.000377
Q6NXE6	Armadillo repeat-containing protein 6 OS=Homo sapiens OX=9606 GN=ARMC6 PE=1 SV=2	0.36468	-1.4553	0.021772
P07951	Tropomyosin beta chain OS=Homo sapiens OX=9606 GN=TPM2 PE=1 SV=1	0.36389	-1.4584	0.008515
Q96AC1	Fermitin family homolog 2 OS=Homo sapiens OX=9606 GN=FERMT2 PE=1 SV=1	0.36361	-1.4595	0.027492
O43491	Band 4.1-like protein 2 OS=Homo sapiens OX=9606 GN=EPB41L2 PE=1 SV=1	0.36055	-1.4717	0.000332
Q96S66	Chloride channel CLIC-like protein 1 OS=Homo sapiens OX=9606 GN=CLCC1 PE=1 SV=1	0.3603	-1.4727	0.023939
P25205	DNA replication licensing factor MCM3 OS=Homo sapiens OX=9606 GN=MCM3 PE=1 SV=3	0.35977	-1.4748	0.005086
Q8N6H7	ADP-ribosylation factor GTPase-activating protein 2 OS=Homo sapiens OX=9606 GN=ARFGAP2 PE=1 SV=1	0.35961	-1.4755	0.014444
O43865	S-adenosylhomocysteine hydrolase-like protein 1 OS=Homo sapiens OX=9606 GN=AHCYL1 PE=1 SV=2	0.35935	-1.4765	0.031119
Q9BXB5	Oxysterol-binding protein-related protein 10 OS=Homo sapiens OX=9606 GN=OSBPL10 PE=1 SV=2	0.35933	-1.4766	0.03524
P49327	Fatty acid synthase OS=Homo sapiens OX=9606 GN=FASN PE=1 SV=3	0.35811	-1.4815	0.01515
Q9GZZ9	Ubiquitin-like modifier-activating enzyme 5 OS=Homo sapiens OX=9606 GN=UBA5 PE=1 SV=1	0.35764	-1.4834	0.001803
Q9Y6I9	Testis-expressed protein 264 OS=Homo sapiens OX=9606 GN=TEX264 PE=1 SV=1	0.35708	-1.4857	0.012695
P47813	Eukaryotic translation initiation factor 1A, X-chromosomal OS=Homo sapiens OX=9606 GN=EIF1AX PE=1 SV=2	0.3565	-1.488	0.018346
Q96IJ6	Mannose-1-phosphate guanyltransferase alpha OS=Homo sapiens OX=9606 GN=GMPPA PE=1 SV=1	0.35567	-1.4914	0.000571
Q9NPJ3	Acyl-coenzyme A thioesterase 13 OS=Homo sapiens OX=9606 GN=ACOT13 PE=1 SV=1	0.35565	-1.4915	0.004382
Q969X5	Endoplasmic reticulum-Golgi intermediate compartment protein 1 OS=Homo sapiens OX=9606 GN=ERGIC1 PE=1 SV=1	0.35479	-1.4949	0.03836
P56537	Eukaryotic translation initiation factor 6 OS=Homo sapiens OX=9606 GN=EIF6 PE=1 SV=1	0.35475	-1.4951	0.039345
Q8N766	ER membrane protein complex subunit 1 OS=Homo sapiens OX=9606 GN=EMC1 PE=1 SV=1	0.35436	-1.4967	0.015878

Q01581	Hydroxymethylglutaryl-CoA synthase, cytoplasmic OS=Homo sapiens OX=9606 GN=HMGCS1 PE=1 SV=2	0.35414	-1.4976	0.020857
Q9HD20	Endoplasmic reticulum transmembrane helix translocase OS=Homo sapiens OX=9606 GN=ATP13A1 PE=1 SV=2	0.35312	-1.5018	0.024012
Q92878	DNA repair protein RAD50 OS=Homo sapiens OX=9606 GN=RAD50 PE=1 SV=1	0.35276	-1.5032	0.004093
O75955	Flotillin-1 OS=Homo sapiens OX=9606 GN=FLOT1 PE=1 SV=3	0.35252	-1.5042	0.042831
Q9Y282	Endoplasmic reticulum-Golgi intermediate compartment protein 3 OS=Homo sapiens OX=9606 GN=ERGIC3 PE=1 SV=1	0.3522	-1.5055	0.000643
Q9UJU6	Drebrin-like protein OS=Homo sapiens OX=9606 GN=DBNL PE=1 SV=1	0.3513	-1.5092	0.040774
O00116	Alkyldihydroxyacetonephosphate synthase, peroxisomal OS=Homo sapiens OX=9606 GN=AGPS PE=1 SV=1	0.34689	-1.5274	0.049996
P54619	5'-AMP-activated protein kinase subunit gamma-1 OS=Homo sapiens OX=9606 GN=PRKAG1 PE=1 SV=1	0.34641	-1.5294	0.000908
Q9NZM1	Myoferlin OS=Homo sapiens OX=9606 GN=MYOF PE=1 SV=1	0.34527	-1.5342	0.005579
Q9NR77	Peroxisomal membrane protein 2 OS=Homo sapiens OX=9606 GN=PXMP2 PE=1 SV=3	0.34371	-1.5407	0.001001
P45954	Short/branched chain specific acyl-CoA dehydrogenase, mitochondrial OS=Homo sapiens OX=9606 GN=ACADSB PE=1 SV=1	0.34289	-1.5442	0.00796
Q14160	Protein scribble homolog OS=Homo sapiens OX=9606 GN=SCRIB PE=1 SV=4	0.34223	-1.547	0.034146
Q13243	Serine/arginine-rich splicing factor 5 OS=Homo sapiens OX=9606 GN=SRSF5 PE=1 SV=1	0.3421	-1.5475	0.009641
O15212	Prefoldin subunit 6 OS=Homo sapiens OX=9606 GN=PFDN6 PE=1 SV=1	0.34098	-1.5523	0.045276
Q9H3P7	Golgi resident protein GCP60 OS=Homo sapiens OX=9606 GN=ACBD3 PE=1 SV=4	0.33944	-1.5588	0.042676
Q27J81	Inverted formin-2 OS=Homo sapiens OX=9606 GN=INF2 PE=1 SV=2	0.3384	-1.5632	0.008105
O60909	Beta-1,4-galactosyltransferase 2 OS=Homo sapiens OX=9606 GN=B4GALT2 PE=1 SV=1	0.33503	-1.5776	0.027011
Q96L92	Sorting nexin-27 OS=Homo sapiens OX=9606 GN=SNX27 PE=1 SV=2	0.33238	-1.5891	0.001249
Q14573	Inositol 1,4,5-trisphosphate receptor type 3 OS=Homo sapiens OX=9606 GN=ITPR3 PE=1 SV=2	0.3299	-1.5999	0.018421
O60610	Protein diaphanous homolog 1 OS=Homo sapiens OX=9606 GN=DIAPH1 PE=1 SV=2	0.32938	-1.6022	0.003709
O15305	Phosphomannomutase 2 OS=Homo sapiens OX=9606 GN=PMM2 PE=1 SV=1	0.32912	-1.6033	0.004638

Q96KB5	Lymphokine-activated killer T-cell-originated protein kinase OS=Homo sapiens OX=9606 GN=PBK PE=1 SV=3	0.32712	-1.6121	0.037186
Q9UJW0	Dynactin subunit 4 OS=Homo sapiens OX=9606 GN=DCTN4 PE=1 SV=1	0.32655	-1.6146	0.028518
Q5VW32	BRO1 domain-containing protein BROX OS=Homo sapiens OX=9606 GN=BROX PE=1 SV=1	0.32644	-1.6151	0.039929
Q9NR56	Muscleblind-like protein 1 OS=Homo sapiens OX=9606 GN=MBNL1 PE=1 SV=2	0.32633	-1.6156	0.001999
Q15427	Splicing factor 3B subunit 4 OS=Homo sapiens OX=9606 GN=SF3B4 PE=1 SV=1	0.32593	-1.6174	0.046045
Q6P1N9	Putative deoxyribonuclease TATDN1 OS=Homo sapiens OX=9606 GN=TATDN1 PE=1 SV=2	0.32578	-1.618	0.007725
Q96PU8	Protein quaking OS=Homo sapiens OX=9606 GN=QKI PE=1 SV=1	0.32524	-1.6204	0.014067
P84098	60S ribosomal protein L19 OS=Homo sapiens OX=9606 GN=RPL19 PE=1 SV=1	0.32375	-1.627	0.037445
P06703	Protein S100-A6 OS=Homo sapiens OX=9606 GN=S100A6 PE=1 SV=1	0.32368	-1.6274	0.047781
O95347	Structural maintenance of chromosomes protein 2 OS=Homo sapiens OX=9606 GN=SMC2 PE=1 SV=2	0.32327	-1.6292	0.000506
Q92783	Signal transducing adapter molecule 1 OS=Homo sapiens OX=9606 GN=STAM PE=1 SV=3	0.31913	-1.6478	0.014999
Q9Y383	Putative RNA-binding protein Luc7-like 2 OS=Homo sapiens OX=9606 GN=LUC7L2 PE=1 SV=2	0.31851	-1.6506	0.011602
Q9UEU0	Vesicle transport through interaction with t-SNAREs homolog 1B OS=Homo sapiens OX=9606 GN=VTI1B PE=1 SV=3	0.3178	-1.6538	0.027721
O60725	Protein-S-isoprenylcysteine O-methyltransferase OS=Homo sapiens OX=9606 GN=ICMT PE=1 SV=1	0.31384	-1.6719	0.035972
Q5JTZ9	Alanine--tRNA ligase, mitochondrial OS=Homo sapiens OX=9606 GN=AARS2 PE=1 SV=1	0.31258	-1.6777	0.006268
Q86Y56	Dynein axonemal assembly factor 5 OS=Homo sapiens OX=9606 GN=DNAAF5 PE=1 SV=4	0.31236	-1.6787	0.039993
O75569	Interferon-inducible double-stranded RNA-dependent protein kinase activator A OS=Homo sapiens OX=9606 GN=PRKRA PE=1 SV=1	0.31093	-1.6853	0.04319
P09496	Clathrin light chain A OS=Homo sapiens OX=9606 GN=CLTA PE=1 SV=1	0.31031	-1.6882	0.00883
P10412	Histone H1.4 OS=Homo sapiens OX=9606 GN=H1-4 PE=1 SV=2	0.30916	-1.6936	0.001816

Q07866	Kinesin light chain 1 OS=Homo sapiens OX=9606 GN=KLC1 PE=1 SV=2	0.30912	-1.6938	0.011845
Q9Y6B6	GTP-binding protein SAR1b OS=Homo sapiens OX=9606 GN=SAR1B PE=1 SV=1	0.30869	-1.6958	0.033814
Q9Y678	Coatomer subunit gamma-1 OS=Homo sapiens OX=9606 GN=COPG1 PE=1 SV=1	0.30672	-1.705	0.00645
P33947	ER lumen protein-retaining receptor 2 OS=Homo sapiens OX=9606 GN=KDELR2 PE=1 SV=1	0.30541	-1.7112	0.039193
Q13618	Cullin-3 OS=Homo sapiens OX=9606 GN=CUL3 PE=1 SV=2	0.30474	-1.7144	0.029684
P61289	Proteasome activator complex subunit 3 OS=Homo sapiens OX=9606 GN=PSME3 PE=1 SV=1	0.304	-1.7179	0.001377
P07437	Tubulin beta chain OS=Homo sapiens OX=9606 GN=TUBB PE=1 SV=2	0.30277	-1.7237	0.035919
Q8WX93	Palladin OS=Homo sapiens OX=9606 GN=PALLD PE=1 SV=3	0.30154	-1.7296	0.003337
Q00796	Sorbitol dehydrogenase OS=Homo sapiens OX=9606 GN=SORD PE=1 SV=4	0.29982	-1.7378	0.048033
P29144	Tripeptidyl-peptidase 2 OS=Homo sapiens OX=9606 GN=TPP2 PE=1 SV=4	0.29976	-1.7381	0.047628
P46821	Microtubule-associated protein 1B OS=Homo sapiens OX=9606 GN=MAP1B PE=1 SV=2	0.29943	-1.7397	0.033156
Q96IZ0	PRKC apoptosis WT1 regulator protein OS=Homo sapiens OX=9606 GN=PAWR PE=1 SV=1	0.29858	-1.7438	0.00533
Q92499	ATP-dependent RNA helicase DDX1 OS=Homo sapiens OX=9606 GN=DDX1 PE=1 SV=2	0.29746	-1.7492	0.015697
Q13423	NAD(P) transhydrogenase, mitochondrial OS=Homo sapiens OX=9606 GN=NNT PE=1 SV=3	0.29444	-1.764	0.002644
O43674	NADH dehydrogenase [ubiquinone] 1 beta subcomplex subunit 5, mitochondrial OS=Homo sapiens OX=9606 GN=NDUFB5 PE=1 SV=1	0.29357	-1.7682	0.037889
P28070	Proteasome subunit beta type-4 OS=Homo sapiens OX=9606 GN=PSMB4 PE=1 SV=4	0.29356	-1.7683	0.018392
Q13557	Calcium/calmodulin-dependent protein kinase type II subunit delta OS=Homo sapiens OX=9606 GN=CAMK2D PE=1 SV=3	0.29268	-1.7726	0.044893
Q8NB25	Protein FAM184A OS=Homo sapiens OX=9606 GN=FAM184A PE=1 SV=3	0.29265	-1.7727	0.036044
O75475	PC4 and SFRS1-interacting protein OS=Homo sapiens OX=9606 GN=PSIP1 PE=1 SV=1	0.29088	-1.7815	0.000363
Q9H3N1	Thioredoxin-related transmembrane protein 1 OS=Homo sapiens OX=9606 GN=TMX1 PE=1 SV=1	0.28904	-1.7907	0.007332

P11717	Cation-independent mannose-6-phosphate receptor OS=Homo sapiens OX=9606 GN=IGF2R PE=1 SV=3	0.2868	-1.8019	0.001224
O00399	Dynactin subunit 6 OS=Homo sapiens OX=9606 GN=DCTN6 PE=1 SV=1	0.28254	-1.8235	0.035945
P07358	Complement component C8 beta chain OS=Homo sapiens OX=9606 GN=C8B PE=1 SV=3	0.28094	-1.8317	0.006928
P49585	Choline-phosphate cytidylyltransferase A OS=Homo sapiens OX=9606 GN=PCYT1A PE=1 SV=2	0.28045	-1.8342	0.034554
Q8NDY3	[Protein ADP-ribosylarginine] hydrolase-like protein 1 OS=Homo sapiens OX=9606 GN=ADPRHL1 PE=2 SV=1	0.2801	-1.836	0.023389
Q9Y446	Plakophilin-3 OS=Homo sapiens OX=9606 GN=PKP3 PE=1 SV=1	0.27985	-1.8373	0.002993
P56545	C-terminal-binding protein 2 OS=Homo sapiens OX=9606 GN=CTBP2 PE=1 SV=1	0.27741	-1.8499	0.003964
Q13268	Dehydrogenase/reductase SDR family member 2, mitochondrial OS=Homo sapiens OX=9606 GN=DHRS2 PE=1 SV=4	0.2769	-1.8526	0.010191
Q7L266	Isoaspartyl peptidase/L-asparaginase OS=Homo sapiens OX=9606 GN=ASRGL1 PE=1 SV=2	0.27598	-1.8573	0.036296
Q9Y617	Phosphoserine aminotransferase OS=Homo sapiens OX=9606 GN=PSAT1 PE=1 SV=2	0.27008	-1.8885	0.040932
Q9UFW8	CGG triplet repeat-binding protein 1 OS=Homo sapiens OX=9606 GN=CGGBP1 PE=1 SV=2	0.2695	-1.8916	0.039211
Q9UH65	Switch-associated protein 70 OS=Homo sapiens OX=9606 GN=SWAP70 PE=1 SV=1	0.26833	-1.8979	0.000248
Q93052	Lipoma-preferred partner OS=Homo sapiens OX=9606 GN=LPP PE=1 SV=1	0.26711	-1.9045	0.01006
Q9UIA9	Exportin-7 OS=Homo sapiens OX=9606 GN=XPO7 PE=1 SV=3	0.26542	-1.9137	0.015998
Q6XQN6	Nicotinate phosphoribosyltransferase OS=Homo sapiens OX=9606 GN=NAPRT PE=1 SV=2	0.26434	-1.9195	0.033921
Q9NZN9	Aryl-hydrocarbon-interacting protein-like 1 OS=Homo sapiens OX=9606 GN=AIPL1 PE=1 SV=2	0.26289	-1.9275	0.008286
Q8IWX8	Calcium homeostasis endoplasmic reticulum protein OS=Homo sapiens OX=9606 GN=CHERP PE=1 SV=3	0.26199	-1.9324	0.030717
O75608	Acyl-protein thioesterase 1 OS=Homo sapiens OX=9606 GN=LYPLA1 PE=1 SV=1	0.26159	-1.9346	0.000193
Q5VSL9	Striatin-interacting protein 1 OS=Homo sapiens OX=9606 GN=STRIP1 PE=1 SV=1	0.26115	-1.937	0.010702
Q13938	Calcyphosin OS=Homo sapiens OX=9606 GN=CAPS PE=1 SV=2	0.25907	-1.9486	0.000535

Q14318	Peptidyl-prolyl cis-trans isomerase FKBP8 OS=Homo sapiens OX=9606 GN=FKBP8 PE=1 SV=2	0.25846	-1.952	0.01216
Q16890	Tumor protein D53 OS=Homo sapiens OX=9606 GN=TPD52L1 PE=1 SV=1	0.25078	-1.9955	0.001093
Q7Z7H5	Transmembrane emp24 domain-containing protein 4 OS=Homo sapiens OX=9606 GN=TMED4 PE=1 SV=1	0.24997	-2.0002	0.004767
P20674	Cytochrome c oxidase subunit 5A, mitochondrial OS=Homo sapiens OX=9606 GN=COX5A PE=1 SV=2	0.24812	-2.0109	0.031627
P48449	Lanosterol synthase OS=Homo sapiens OX=9606 GN=LSS PE=1 SV=1	0.24582	-2.0243	0.007104
Q9HAB8	Phosphopantothenate--cysteine ligase OS=Homo sapiens OX=9606 GN=PPCS PE=1 SV=2	0.24297	-2.0411	0.004381
P17612	cAMP-dependent protein kinase catalytic subunit alpha OS=Homo sapiens OX=9606 GN=PRKACA PE=1 SV=2	0.23926	-2.0634	0.045529
P15151	Poliovirus receptor OS=Homo sapiens OX=9606 GN=PVR PE=1 SV=2	0.23735	-2.0749	0.010503
Q9BRJ2	39S ribosomal protein L45, mitochondrial OS=Homo sapiens OX=9606 GN=MRPL45 PE=1 SV=2	0.23408	-2.0949	0.008363
P46108	Adapter molecule crk OS=Homo sapiens OX=9606 GN=CRK PE=1 SV=2	0.22779	-2.1342	0.043586
P05107	Integrin beta-2 OS=Homo sapiens OX=9606 GN=ITGB2 PE=1 SV=2	0.22764	-2.1351	0.000398
Q02413	Desmoglein-1 OS=Homo sapiens OX=9606 GN=DSG1 PE=1 SV=2	0.22646	-2.1426	0.007603
Q4G0F5	Vacuolar protein sorting-associated protein 26B OS=Homo sapiens OX=9606 GN=VPS26B PE=1 SV=2	0.22504	-2.1517	0.001099
Q99700	Ataxin-2 OS=Homo sapiens OX=9606 GN=ATXN2 PE=1 SV=2	0.22485	-2.153	0.041085
P82909	28S ribosomal protein S36, mitochondrial OS=Homo sapiens OX=9606 GN=MRPS36 PE=1 SV=2	0.22438	-2.156	0.037268
Q96KG9	N-terminal kinase-like protein OS=Homo sapiens OX=9606 GN=SCYL1 PE=1 SV=1	0.21884	-2.1921	0.025682
A0FGR8	Extended synaptotagmin-2 OS=Homo sapiens OX=9606 GN=ESYT2 PE=1 SV=1	0.21851	-2.1942	0.001405
P19387	DNA-directed RNA polymerase II subunit RPB3 OS=Homo sapiens OX=9606 GN=POLR2C PE=1 SV=2	0.21773	-2.1994	0.008421
Q9H0U3	Magnesium transporter protein 1 OS=Homo sapiens OX=9606 GN=MAGT1 PE=1 SV=1	0.21638	-2.2083	0.045159
P49736	DNA replication licensing factor MCM2 OS=Homo sapiens OX=9606 GN=MCM2 PE=1 SV=4	0.21432	-2.2222	0.003499

O96005	Cleft lip and palate transmembrane protein 1 OS=Homo sapiens OX=9606 GN=CLPTM1 PE=1 SV=1	0.20951	-2.2549	0.019922
O14672	Disintegrin and metalloproteinase domain-containing protein 10 OS=Homo sapiens OX=9606 GN=ADAM10 PE=1 SV=1	0.20479	-2.2878	0.005573
Q9BWD1	Acetyl-CoA acetyltransferase, cytosolic OS=Homo sapiens OX=9606 GN=ACAT2 PE=1 SV=2	0.203	-2.3004	0.011556
Q01650	Large neutral amino acids transporter small subunit 1 OS=Homo sapiens OX=9606 GN=SLC7A5 PE=1 SV=2	0.1886	-2.4066	0.000103
Q9H6T3	RNA polymerase II-associated protein 3 OS=Homo sapiens OX=9606 GN=RPAP3 PE=1 SV=2	0.1872	-2.4173	0.021988
Q15021	Condensin complex subunit 1 OS=Homo sapiens OX=9606 GN=NCAPD2 PE=1 SV=3	0.18254	-2.4537	0.012592
P55327	Tumor protein D52 OS=Homo sapiens OX=9606 GN=TPD52 PE=1 SV=2	0.17816	-2.4887	0.044933
P23634	Plasma membrane calcium-transporting ATPase 4 OS=Homo sapiens OX=9606 GN=ATP2B4 PE=1 SV=2	0.17416	-2.5215	0.01175
Q12965	Unconventional myosin-Ie OS=Homo sapiens OX=9606 GN=MYO1E PE=1 SV=2	0.15167	-2.721	0.047315
P61803	Dolichyl-diphosphooligosaccharide--protein glycosyltransferase subunit DAD1 OS=Homo sapiens OX=9606 GN=DAD1 PE=1 SV=3	0.15084	-2.7289	0.035274
Q9NUQ6	SPATS2-like protein OS=Homo sapiens OX=9606 GN=SPATS2L PE=1 SV=2	0.1427	-2.809	0.000741
A0MZ66	Shootin-1 OS=Homo sapiens OX=9606 GN=SHTN1 PE=1 SV=4	0.13587	-2.8797	0.004937
O43852	Calumenin OS=Homo sapiens OX=9606 GN=CALU PE=1 SV=2	0.13294	-2.9112	0.045015
Q15758	Neutral amino acid transporter B(0) OS=Homo sapiens OX=9606 GN=SLC1A5 PE=1 SV=2	0.11727	-3.0921	0.001821
Q14192	Four and a half LIM domains protein 2 OS=Homo sapiens OX=9606 GN=FHL2 PE=1 SV=3	0.092226	-3.4387	0.005708
