# Supplementary material for: Proteomics and Bioinformatics Investigations Link Overexpression of FGF8 and Associated Hub Genes to the Progression of Ovarian Cancer and Poor Prognosis
Source: Biochem Res Int. 2024 Sep 13;2024:4288753. doi: 10.1155/2024/4288753 (PMC11415250; doi:10.1155/2024/4288753)
Supplement: Supplementary Materials — Supplementary Figure 1: venn diagram of proteins identified in FGF8-silenced and FGF8-expressing ovarian cancer cells (SKOV3). Supplementary File 1: proteins identified in ovarian cancer cells (SKOV3). Supplementary File 2: differentially expressed proteins identified by volcano plot analysis. Supplementary File 3: enrichment of GO terms and pathways among downregulated proteins in FGF8-silenced ovarian cancer cells (SKOV3). [file 4288753.f1.zip › Supplementary file 3.pdf]

**Supplementary file 3:** Enrichment of GO terms and pathways among downregulated proteins in FGF8-silenced ovarian cancer cells (SKOV3).

**Biological Processes**

| Term | Count | PValue | Genes | Fold Enrichment | FDR |
|---|---|---|---|---|---|
| GO:0002181~cytoplasmic translation | 17 | 4.22E-11 | Q07020, P26373, P18077, Q9H446, P18124, P42677, Q02878, P84098, P15880, P30050, P63220, P61247, P46777, P62851, P62753, P49207, P61513 | 9.138805 | 9.66E-08 |
| GO:0006412~translation | 22 | 7.89E-09 | Q07020, P26373, P18077, Q9BYD6, Q8NE71, P18124, P42677, Q9Y3B7, Q02878, P84098, P15880, P30050, P63220, P61247, P15170, Q9Y450, P46777, P62851, P62753, Q9P2E9, P49207, P61513 | 4.773103 | 8.69E-06 |
| GO:0010499~proteasomal ubiquitin-independent protein catabolic process | 9 | 1.14E-08 | P28066, O14818, P25789, P28074, P49721, P28072, P60900, P28070, P25786 | 18.93205 | 8.69E-06 |
| GO:0010498~proteasomal protein catabolic process | 9 | 3.45E-07 | P28066, O14818, P25789, P28074, P49721, P28072, P60900, P28070, P25786 | 12.80698 | 1.98E-04 |
| GO:0050821~protein stabilization | 17 | 1.40E-05 | Q9UL25, O75955, O43670, O75569, Q9H6T3, P50148, P17987, Q9BXJ9, Q02413, Q99497, O95817, O15212, P48643, P46777, P35080, Q969V3, P21333 | 3.738602 | 0.006432 |
| GO:0015031~protein transport | 24 | 4.52E-05 | Q9UL25, P49321, P33947, Q92609, O75674, Q8TEM1, Q8N1F7, Q03135, Q08752, P50897, Q8N6H7, Q86UP2, Q9UEU0, Q9Y3E7, Q8TCE6, Q8IYI6, O14828, Q9UMY4, Q9NUP9, Q92783, Q9UM54, O14964, Q9P2E9, Q9HD20 | 2.645025 | 0.01728 |
| GO:1990592~protein K69-linked ufmylation | 4 | 8.43E-05 | O94874, Q9Y3C8, Q96HY6, Q9GZZ9 | 38.70553 | 0.027615 |

| | | | | | |
|---|---|---|---|---|---|
| GO:0071569~protein ufmylation | 4 | 1.66E-04 | O94874, Q9Y3C8, Q96HY6, Q9GZZ9 | 32.25461 | 0.047586 |

**Cellular components**

| Term | Count | PValue | Genes | Fold Enrichment | FDR |
|---|---|---|---|---|---|
| GO:0005829~cytosol | 238 | 7.08E-41 | Q9Y266, Q15417, P67809, P49023, P54619, Q04760, Q01130, Q96GX9, Q9UEY8, Q9NTK5, Q92973, O94903, P09496, Q13243, P08729, Q92609, Q9GZZ9, O43684, O00154, Q8NE71, Q9NR77, O00399, O75381, Q6NXE6, P09382, P20042, Q16643, P46777, Q29RF7, Q9H2G2, Q13136, Q13813, P30626, Q8IVF2, P42167, P63220, P61289, Q9UJU6, P00441, P35749, O95347, P31948, P26373, Q16537, Q01581, P18077, Q9BXB5, Q8TD19, O75475, Q9UKG1, Q9Y617, Q8N6H7, P62140, P49588, P43487, Q9Y6B6, O15305, P80303, Q9H2M9, A0FGR8, P49189, P78344, P28482, Q4G0F5, Q9UEU0, Q9Y3E7, Q96RQ3, P21964, P35221, Q9Y6K9, Q02413, P23588, P15170, P61586, P48643, O14744, P62312, P46108, P07437, Q9UL25, Q9H6T3, Q32MZ4, Q9NTX5, Q08752, O00116, Q93052, P49721, Q9UM54, Q6XQN6, O43175, O75569, O95810, Q06203, Q13618, O00244, Q9H6S3, Q7L266, P11586, Q99700, Q02878, Q9UNH7, P49959, O95817, P24534, P55010, P61247, Q15365, P51452, Q13868, O75674, Q9NXA8, O43865, Q9NR56, Q9BWD1, Q9NY33, Q15370, P27816, Q99497, Q9NTJ3, P24666, P02533, P21399, P53004, O95831, P54577, Q96L92, Q13637, Q9Y6W5, Q01650, P18124, P50897, Q00796, Q9Y3A3, O60841, Q9Y3A5, Q96AC1, Q07866, P62877, Q9UH65, Q15021, P28838, | 2.164475 | 3.45E-38 |

| | | | | | |
|---|---|---|---|---|---|
| | | | Q96KG9, Q8IWB7, P46821, P62753, P25325, O94874, Q13642, Q9Y678, P29144, P49773, P06703, O15013, Q13404, Q9BRQ6, Q01082, P12268, Q05682, O60610, P84098, Q8WX93, Q9P2K8, O43290, P06396, P60900, P26640, P56537, Q9Y6I9, P25789, Q9Y450, O14964, P25786, Q8N1F7, P35222, P34932, P42677, O75083, P15880, Q8WXX5, Q8TCG1, Q9BXJ9, Q92783, O14974, P41227, O43598, P49585, P53680, Q8WWM7, Q13557, Q9BTZ2, Q9HAB8, P17987, Q9NUQ6, Q9UJW0, P30050, Q92499, Q13561, P07951, Q07020, P46060, Q5VSL9, P19387, Q07817, Q8IYI6, P49327, O43491, Q6IQ22, P49207, P61513, P21333, P28066, Q99426, Q9H3U1, P61088, P17174, Q92598, Q16851, P05198, P62851, Q9BVK6, P47813, O75608, Q14677, Q14318, P28074, P62191, P28072, P28070, Q13907, P50402, O75031, P17612, Q9NPJ3, O14936, O14818, Q08209, Q8N0X7, Q9NZN9 | | |
| GO:0070062~extracellular exosome | 132 | 1.02E-31 | P53004, Q16629, P67809, O14672, Q9Y6W5, P56199, Q15819, Q04760, Q08722, Q01650, P50897, Q9P2R7, P53007, Q9NYU2, Q00796, O14828, Q92973, Q9NTK5, P28838, P25325, P07358, P08729, P49773, P06703, Q13404, O00154, Q01082, P12268, P50148, Q96IJ6, P09382, P09543, P06396, P60900, P46777, Q9Y281, Q9H2G2, Q12965, Q13813, P30626, P56537, P30740, Q9Y3C8, P25789, Q9Y450, O14964, Q9UJU6, P35080, P25786, P00441, P11717, P35749, O95347, P18077, Q9UKG1, P35222, P34932, Q9Y617, P12004, O75083, P15880, P62140, P49588, P80303, O43598, P49189, Q9BYC5, Q9NX63, Q9Y3E7, P17987, P21964, O00592, P30050, Q92896, P61586, P48643, P29966, Q13561, P46108, O14745, P07437, O43760, | 2.964359 | 2.48E-29 |

| | | | | | |
|---|---|---|---|---|---|
| | | | Q9Y512, Q9UL25, Q5VSL9, Q5VW32, P49327, P49721, P07686, Q9UM54, Q6XQN6, O43491, P21333, P49207, P61513, O43175, P05107, P28066, Q15758, Q6IBS0, Q13618, Q9H6S3, P11586, P61088, P17174, Q9NZM1, P61247, Q92598, Q16851, P05198, P62851, Q9BVK6, Q15365, O75608, O75955, O75674, P28074, O43865, P28072, P28070, Q9BWD1, P17612, Q9NY33, O14818, Q14160, Q99497, P24666, O14773, P02533, P08754, Q9Y2W1, P21399 | | |
| GO:0005737~cytoplasm | 207 | 8.82E-24 | Q9BZZ5, O43670, Q16629, Q9Y266, O14672, P67809, P49023, P54619, Q04760, Q01130, A0MZ66, Q96GX9, Q9NTK5, Q92973, O94903, Q92614, Q14573, Q96HY6, P08729, Q9GZZ9, Q8NE71, Q86Y56, Q9NR77, O00399, P50148, Q9UBT2, Q96IJ6, P09382, Q9UIA9, P20042, Q16643, P46777, Q9Y281, Q9H2G2, Q13136, P35637, Q12965, P30626, Q96IZ0, Q13938, Q8IVF2, P63220, P61289, P35080, Q9UJU6, Q16890, P00441, Q12972, P35749, O95347, Q13268, Q8IWX8, P26373, Q16537, Q01581, P18077, Q9UKG1, Q9Y617, P62140, Q9NUP9, P49588, P43487, O15305, P49189, P78344, P28482, A6NDG6, P35221, O00592, Q9Y6K9, P15170, P48643, O14744, P62312, P49736, P46108, O14745, P07437, Q32MZ4, Q08752, Q86X76, P49721, Q9UM54, O75569, O95810, Q13618, Q9H6S3, Q7L266, Q99700, Q02878, Q9UNH7, P49959, P15151, Q96PK6, O95817, P24534, P55010, P61247, Q14157, Q15365, P51452, Q13868, P0DP25, O75674, O43865, Q9NR56, Q9BWD1, Q9NY33, P27816, Q99497, P24666, Q15131, P08754, P02533, P21399, O95831, P54577, Q15819, Q9H446, P18124, Q9Y3A3, O60841, Q9Y3A5, Q96AC1, Q07866, Q9UH65, P25205, Q15021, P28838, | 1.851116 | 1.43E-21 |

| | | | | | |
|---|---|---|---|---|---|
| | | | Q96KG9, P62753, O94874, Q13642, P29144, P49773, P06703, Q13404, Q01082, P12268, O60610, P84098, O43290, P09543, Q2TAL8, P06396, P60900, Q9Y446, P56537, P55327, P25789, O14964, P25786, P35222, P15880, Q8WXX5, Q8TCG1, Q9BXJ9, O14974, P41227, Q13557, Q96BJ3, Q9HAB8, Q12906, Q96PU8, Q9NUQ6, Q9UJW0, P30050, Q92499, P29966, Q13561, Q07020, P46060, P46063, Q07817, P49327, P49207, P61513, P21333, Q9HCU4, P28066, P49321, Q6IBS0, Q9NW64, Q99426, Q9H3U1, P61088, P17174, Q92598, Q16851, O15212, P05198, P62851, P47813, O75608, Q7Z4H3, P28074, P62191, P28072, P28070, Q13907, P50402, P17612, O14936, O14818, Q08209, Q8N0X7, Q9NZN9 | | |
| GO:0016020~membrane | 122 | 9.61E-21 | Q9BZZ5, O96005, Q96L92, Q13637, O14672, P56199, P54619, Q01650, P18124, P50897, O60725, Q96S66, Q00796, Q9H0U3, Q9UEY8, Q07866, Q9NTK5, P25205, Q15021, Q96KG9, P09496, Q92614, P62753, Q14573, P07358, P48449, O94874, P30876, Q8NE71, Q9NR77, P12268, P50148, O75381, P84098, P09543, P46777, Q9HD20, Q9Y282, Q13813, P30626, Q8TEM1, O75400, Q03135, O95573, O00203, P30740, P42167, P23368, O15533, Q9Y450, P61289, P11717, Q13423, Q8IWX8, P26373, Q9H3P7, P18077, Q13308, Q9BXB5, Q8N1F7, Q9UKG1, P35222, P15880, Q9BXJ9, P49588, P41227, Q9P2E9, A0FGR8, P78344, Q8WWM7, Q13557, Q9BQ39, Q96BJ3, Q9BYC5, Q12905, Q12906, P21964, P35221, P30050, Q92896, Q92499, Q969X5, P29966, Q13561, P46108, O14745, Q07020, P46063, O00116, Q5VW32, Q8IYI6, P49327, P49721, P23634, P07686, Q9UM54, P21333, P61803, | 2.385884 | 1.17E-18 |

| | | | | | |
|---|---|---|---|---|---|
| | | | P05107, O43852, O75569, Q15758, Q13618, P11586, Q99700, Q02878, O95817, P00403, P05198, Q92878, Q969V3, Q15365, Q14677, O75955, Q14318, O75674, P62191, P50402, Q86UP2, Q8TCT9, P17612, P08754 | | |
| GO:0005925~focal adhesion | 40 | 2.22E-15 | P05107, Q13136, P28482, O14672, Q15417, P49023, P56199, Q03135, P18124, Q02878, P35221, Q96AC1, Q9UJW0, Q14192, P30050, P15151, P61247, P28838, P61586, P29966, P11717, Q07020, Q9UL25, Q13642, O75955, Q13308, P35222, Q93052, P84098, P15880, O14936, Q8WX93, P62140, P06396, P46777, P29317, O14974, O43491, P21333, P61513 | 4.699077 | 2.16E-13 |
| GO:0022626~cytosolic ribosome | 17 | 2.24E-12 | Q07020, P26373, P18077, P18124, P42677, Q02878, P84098, P15880, P30050, P63220, Q9P2K8, P61247, P46777, P62851, P62753, P49207, P61513 | 10.99706 | 1.82E-10 |
| GO:0005839~proteasome core complex | 9 | 3.92E-09 | P28066, O14818, P25789, P28074, P49721, P28072, P60900, P28070, P25786 | 21.34724 | 2.73E-07 |
| GO:0005654~nucleoplasm | 127 | 1.28E-08 | Q8IWS0, O43670, Q96L92, Q16629, Q9Y266, P67809, O43674, Q15819, P54619, Q04760, Q01130, Q9Y3A5, Q96AC1, P62877, P25205, P28838, Q15021, Q13243, P62753, Q9Y3Z3, Q14573, O00712, P30876, P29144, P49773, Q13404, Q15427, O43684, O00154, Q8NE71, P35249, Q9UBT2, Q8TAE8, O43290, Q2TAL8, P60900, P46777, Q29RF7, Q9Y446, P27694, Q9ULX9, P35637, P30626, O75400, P56537, P11387, Q14192, P42285, P63220, P25789, P61289, P25786, P00441, Q12972, O95347, Q13268, Q8IWX8, O75475, P35222, P12004, P42677, P15880, Q8WXX5, P62140, O14974, O15305, Q13952, P23193, | 1.605965 | 7.77E-07 |

| | | | | | |
|---|---|---|---|---|---|
| | | | P28482, Q13557, P78345, Q8IX01, Q6P1N9, Q12905, Q12906, Q9NUQ6, Q9Y6K9, Q9UFW8, Q92499, Q14498, P62312, O14744, Q969X5, P49736, P46060, P46063, P19387, Q08752, P49721, Q9UM54, O43491, P26440, P28066, O75569, P49321, O60909, O95810, Q13618, Q9NW64, P49959, P61088, O14929, P17174, Q96PK6, P61247, Q92598, P62851, Q92878, Q15365, P51452, Q14677, Q13868, P28074, P62191, P28072, Q9NR56, P28070, P50402, P17612, Q15370, O14818, Q14160, Q08209, Q99497, Q9NTJ3, Q9Y2W1, Q9NZN9 | | |
| GO:0005634~nucleus | 170 | 5.76E-08 | Q9BZZ5, O95831, Q8IWS0, O43670, Q16629, P67809, O14672, Q15819, P54619, P18124, P50897, Q01130, P53007, Q9Y3A5, Q96AC1, Q92973, P62877, Q9UH65, P25205, P28838, Q15021, Q96KG9, Q8IWB7, Q92614, P62753, Q9Y3Z3, O00712, Q13523, O94874, Q13642, P30876, P08729, P49773, P06703, Q13404, Q9GZZ9, Q15427, Q01082, P12268, P35249, O75381, O60610, Q8WX93, P09382, Q9UIA9, O43290, Q2TAL8, P06396, P60900, P46777, Q29RF7, Q9Y446, P27694, Q9ULX9, P35637, Q96IZ0, Q9BTT0, P56537, P11387, Q9Y6I9, Q8IVF2, P42167, Q14192, P42285, P25789, P61289, P25786, P00441, Q12972, O95347, P56545, Q13268, P31948, P26373, Q16537, Q8TD19, O75475, Q9UKG1, P35222, P34932, Q14919, P12004, P42677, P15880, Q8WXX5, P62140, Q9BXJ9, P43487, P41227, Q13952, O43598, P23193, P49585, P28482, Q13557, Q8IX01, Q9BTZ2, Q9HAB8, Q12905, Q12906, Q9NX63, Q96PU8, Q9UJW0, Q9Y6K9, Q9UFW8, Q92499, P61586, Q14498, P62312, O14744, P49736, P46108, P07437, Q07020, P46060, P46063, Q96KB5, Q5VSL9, Q32MZ4, | 1.427949 | 3.12E-06 |

| | | | | | |
|---|---|---|---|---|---|
| | | | P19387, Q08752, Q86X76, Q93052, P49721, Q9UM54, P21333, P61513, P09622, P28066, O75569, P49321, Q13618, P10412, Q7L266, Q9NW64, Q02878, P49959, Q9UNH7, P61088, O14929, P17174, O95817, Q96PK6, P61247, Q92598, Q16851, P05198, P62851, Q92878, Q14157, Q15365, P51452, Q13868, P0DP25, P28074, P62191, Q9NXA8, P28072, Q9NR56, P28070, P16403, P17612, Q9NPJ3, O14818, Q99497, Q9NTJ3, Q15131, P02533, Q9Y2W1, Q9NZN9 | | |
| GO:0005840~ribosome | 18 | 9.15E-08 | Q07020, P26373, P18077, Q8NE71, P42677, Q9Y3B7, Q02878, P15880, Q8TAE8, P63220, P61247, Q9BRJ2, P46777, P62851, P62753, Q9P2E9, P49207, P61513 | 5.123337 | 4.46E-06 |
| GO:0000502~proteasome complex | 11 | 1.74E-07 | P28066, O14818, P25789, P62191, P28074, P49721, P61289, P28072, P60900, P28070, P25786 | 9.612498 | 7.68E-06 |
| GO:0005739~mitochondrion | 59 | 2.54E-07 | O95831, Q13637, P28482, O43674, Q9BTZ2, Q96IX5, Q5VTU8, Q9NX63, Q12906, P53007, Q9P2R7, O60841, Q96RQ3, O14949, Q92499, Q15388, P25325, O14548, Q9Y512, Q9BRQ6, O00154, Q5JTZ9, Q9NR77, O00116, Q86X76, Q07817, Q9Y3B7, Q8TAE8, Q8WX93, P09622, P26440, P09669, Q9ULX9, P30626, P82909, P11586, O00203, P55809, O14929, P23368, P00403, P45954, P00441, Q13423, Q13268, Q14318, Q9H3P7, Q9NXA8, Q9BYD6, P28070, P34932, Q9BWD1, P17612, Q4VC31, Q08209, Q99497, P49588, Q9BRJ2, P21399 | 2.045096 | 1.03E-05 |
| GO:0022625~cytosolic large ribosomal subunit | 11 | 2.87E-07 | Q07020, Q02878, P84098, P30050, P26373, P18077, Q9BYD6, P18124, P46777, P49207, P61513 | 9.131873 | 1.08E-05 |

| | | | | | |
|---|---|---|---|---|---|
| GO:0015629~actin cytoskeleton | 20 | 1.22E-06 | Q12965, Q96IZ0, Q15417, Q9Y6W5, Q05682, O75083, O14936, Q8WX93, P35221, Q9UH65, P06396, Q16643, Q9UM54, P29966, O14974, Q9Y281, P46108, P07951, P21333, O14745 | 3.861257 | 4.26E-05 |
| GO:0005844~polysome | 8 | 6.21E-06 | Q99700, P35637, Q9P2K8, Q9H446, P05198, P60900, P62753, P25786 | 11.06894 | 2.02E-04 |
| GO:0030027~lamellipodium | 16 | 8.00E-06 | O75955, Q9Y6W5, P49023, Q6IBS0, P35222, A0MZ66, Q8WX93, P35221, Q14160, O00592, Q9UH65, P61586, P06396, Q16643, P29317, Q9UJU6 | 4.150852 | 2.43E-04 |
| GO:0005938~cell cortex | 14 | 2.75E-05 | Q13813, P49023, Q03135, P35222, P52943, Q96AC1, Q9UEY8, Q9UJW0, P61586, Q9UM54, P29966, O43491, Q9UJU6, P21333 | 4.252092 | 7.87E-04 |
| GO:0019773~proteasome core complex, alpha-subunit complex | 5 | 4.68E-05 | P28066, O14818, P25789, P60900, P25786 | 22.64101 | 0.001266 |
| GO:0005856~cytoskeleton | 26 | 1.03E-04 | Q9NYL9, Q96L92, Q12965, Q13813, P28482, Q15417, Q9Y6W5, P49023, Q6IBS0, P35221, Q96AC1, Q9UEY8, Q92896, P61586, P35080, Q9UJU6, P07951, P07437, P49773, Q32MZ4, Q9BXB5, Q05682, Q16643, P02533, O14974, O43491 | 2.393837 | 0.002628 |
| GO:0005911~cell-cell junction | 14 | 1.38E-04 | Q9Y446, O75955, Q9Y6W5, P49023, Q13308, P35222, O75083, O14936, P35221, Q9UEY8, Q14160, Q02413, Q9NUP9, P21333 | 3.631995 | 0.00337 |
| GO:0048471~perinuclear region of cytoplasm | 32 | 1.68E-04 | Q9H2G2, O95831, P35637, O75569, Q6IBS0, Q03135, O95573, A0MZ66, P55327, Q99700, Q9UEU0, Q9Y3A3, Q9H3U1, P25205, P46821, P62753, Q16890, O14745, P11717, P46060, Q14677, P06703, Q8IWX8, P35222, | 2.089026 | 0.003895 |

| | | | | | |
|---|---|---|---|---|---|
| | | | P17612, Q8IYI6, Q99497, P09543, P06396, Q9UM54, Q27J81, P21333 | | |
| GO:0032991~macromolecular complex | 30 | 2.10E-04 | Q8N766, Q13136, Q9H2M9, P49321, P28482, Q9Y6W5, Q9H6S3, Q03135, Q9NYU2, Q9NUQ6, Q9Y6K9, P61088, O14929, Q92598, Q14498, Q969V3, P46108, P00441, P07437, O94874, Q14318, P31948, Q13404, P0DP25, P35222, Q9NR77, O75381, P23634, P06396, P46777 | 2.125614 | 0.00465 |
| GO:0005912~adherens junction | 13 | 2.21E-04 | Q9NYL9, Q9Y446, O75955, Q12965, O14672, Q15417, P78344, P35222, Q96AC1, P35221, Q14160, P15151, Q99497 | 3.700188 | 0.004682 |
| GO:0005743~mitochondrial inner membrane | 22 | 4.77E-04 | O95831, Q13423, P09669, P14927, O43674, Q9BRQ6, P82909, Q9BYD6, Q96IX5, Q5VTU8, O96000, Q9NX63, P53007, Q07817, Q9Y3B7, Q8TAE8, O14949, P00403, P09543, Q9BRJ2, O14548, P20674 | 2.361691 | 0.009671 |
| GO:0005635~nuclear envelope | 13 | 5.63E-04 | P46060, P80303, Q13268, P06703, P49585, Q8TEM1, Q8NE71, Q8N1F7, P50402, P42167, Q9NZM1, Q9UH99, P43487 | 3.337798 | 0.010817 |
| GO:0005794~Golgi apparatus | 40 | 5.91E-04 | P80303, O43852, Q9Y266, P28482, O14672, O60909, Q13618, Q9BYC5, Q03135, O95573, P50897, O00203, Q99700, Q9UEU0, Q9Y3A3, Q9H3U1, P17987, Q92896, Q96KG9, Q8IWB7, Q9BVK6, O14744, P11717, Q14677, Q9Y678, P31948, Q92609, Q9GZZ9, Q9H3P7, Q8N6H7, P50148, O43290, P49327, O14773, Q7Z7H5, P08754, Q9UM54, Q9Y6B6, Q6IQ22, P21399 | 1.775765 | 0.010817 |
| GO:0005884~actin filament | 9 | 6.00E-04 | O60610, Q96IZ0, Q8WX93, Q6IBS0, Q16643, Q9UM54, Q9UJU6, P07951, P21333 | 4.769064 | 0.010817 |

| | | | | | |
|---|---|---|---|---|---|
| GO:0005783~endoplasmic reticulum | 40 | 8.33E-04 | P80303, Q13637, O43852, P49585, P67809, Q03135, O95573, O60725, P55327, Q96S66, Q9NYU2, Q9H0U3, P24534, O15533, P61247, Q9BVK6, Q14696, Q969X5, P62753, Q14573, O75608, Q07020, O94874, Q96HY6, Q9Y678, Q14318, P33947, P26373, P50402, Q8TCT9, Q86UP2, Q07817, Q99497, Q7Z7H5, P46777, Q9Y6B6, Q9P2E9, P49207, Q9Y282, P21399 | 1.744666 | 0.01448 |
| GO:0001726~ruffle | 9 | 9.65E-04 | Q8WX93, P06703, Q9Y6W5, O00592, P06396, Q9UKG1, Q9UM54, Q9UJU6, O14745 | 4.438534 | 0.016201 |
| GO:0042470~melanosome | 9 | 0.001097 | P35749, O75955, Q13637, O43852, Q15758, O14773, P49327, P09543, P06396 | 4.352349 | 0.017447 |
| GO:0005793~endoplasmic reticulum-Golgi intermediate compartment | 8 | 0.001111 | Q9NYU2, P80303, Q9Y678, Q7Z7H5, Q96KG9, Q92614, Q9BVK6, Q969X5 | 4.981022 | 0.017447 |
| GO:0019774~proteasome core complex, beta-subunit complex | 4 | 0.001167 | P28074, P49721, P28072, P28070 | 18.11281 | 0.017766 |
| GO:0030864~cortical actin cytoskeleton | 7 | 0.00125 | O75083, O75955, Q13813, P06396, Q01082, Q16643, Q9UJU6 | 5.811192 | 0.018132 |
| GO:0005789~endoplasmic reticulum membrane | 37 | 0.001266 | Q8N766, P61803, Q9H2M9, O43852, P30626, P49585, A0FGR8, Q8TEM1, Q9BTZ2, Q03135, O95573, O60725, Q9Y6I9, Q96S66, Q9UEU0, Q9H0U3, O15533, P61586, Q9BVK6, Q969V3, Q14573, P48449, O94874, Q9UL25, Q96HY6, Q9Y678, P33947, Q9GZZ9, O43865, Q9H3N1, Q8TCT9, Q86UP2, Q7Z7H5, P08754, Q9Y6B6, Q9Y282, Q9HD20 | 1.75188 | 0.018132 |

| | | | | | |
|---|---|---|---|---|---|
| GO:0005769~early endosome | 16 | 0.001467 | P11717, Q9UL25, O75955, Q96L92, Q13637, Q9Y6W5, P28482, Q9UKG1, Q4G0F5, O00203, P30740, Q9Y3E7, Q9UMY4, Q8IWB7, O14964, Q9UJU6 | 2.587544 | 0.020184 |
| GO:0030018~Z disc | 10 | 0.001533 | P30626, Q8WX93, Q14192, Q08209, O95817, P23634, P35222, O14974, Q9Y281, P21333 | 3.717181 | 0.020184 |
| GO:0030904~retromer complex | 4 | 0.001533 | Q9UNH7, Q92609, Q9UMY4, Q4G0F5 | 16.60341 | 0.020184 |
| GO:0045121~membrane raft | 14 | 0.001729 | P07437, O75955, O95810, P56199, Q96C19, Q03135, P50897, O00592, Q99497, O14773, P23634, P08754, P46108, O14745 | 2.778259 | 0.022163 |
| GO:0022627~cytosolic small ribosomal subunit | 6 | 0.002194 | P42677, P15880, P63220, P61247, P62851, P62753 | 6.496985 | 0.027403 |
| GO:0000781~chromosome, telomeric region | 11 | 0.002624 | P27694, P30876, P49959, P62140, O14929, Q9UH99, P25205, Q92878, A6NHR9, P12004, P49736 | 3.167124 | 0.031952 |
| GO:0101031~chaperone complex | 5 | 0.002877 | Q8WXX5, P31948, Q9H6T3, O95817, O15212 | 8.301703 | 0.034173 |
| GO:0014069~postsynaptic density | 14 | 0.002964 | O14672, Q15417, P28482, Q01082, P18124, P42677, Q02878, Q9UEY8, Q14160, P30050, Q16643, P46821, P62851, Q9UJU6 | 2.611772 | 0.034363 |
| GO:0031965~nuclear membrane | 13 | 0.003537 | P46060, P67809, Q8TEM1, Q8N1F7, P50402, P50148, Q96S66, Q07817, P42167, Q9NZM1, Q9UH99, Q9UM54, O75608 | 2.686858 | 0.039656 |
| GO:0005813~centrosome | 22 | 0.003583 | O15013, P0DP25, Q13618, P28074, Q8N1F7, Q8TD19, P35222, O00399, P12004, Q07817, P17612, P17987, Q9UJW0, Q9NTK5, P25205, P48643, P43487, P08754, P29966, O14974, Q13561, P25786 | 2.003336 | 0.039656 |

| | | | | | |
|---|---|---|---|---|---|
| GO:0042788~polysomal ribosome | 5 | 0.004098 | Q07020, Q02878, P84098, P63220, Q8NE71 | 7.547003 | 0.04435 |

**Molecular Functions**

| Term | Count | PValue | Genes | Fold Enrichment | FDR |
|---|---|---|---|---|---|
| GO:0003723~RNA binding | 96 | 5.03E-23 | Q9BZZ5, P54577, Q8IWS0, O43670, Q16629, P67809, P78344, Q8WWM7, Q9BQ39, Q8IX01, P18124, Q12905, Q12906, Q01130, Q96PU8, P17987, O60841, Q9Y3A5, Q9NUQ6, P35221, P30050, Q92973, P23588, Q92499, P15170, P62312, Q13243, Q92614, Q14498, P62753, Q9Y3Z3, Q13523, Q07020, P46060, P30876, Q15427, Q8NE71, Q01082, P12268, O60610, Q9Y3B7, P84098, P09382, O43290, P49327, P09543, P20042, P60900, P46777, P21333, P49207, P61513, P35637, O75569, Q6IBS0, O75400, O43414, P10412, Q9NW64, P11387, Q99700, Q02878, P61088, P42285, P63220, P55010, Q96PK6, P61247, P05198, Q14157, P62851, P47813, Q15365, P25786, Q12972, Q13868, Q8IWX8, P31948, P26373, P18077, P62191, O43865, Q9BYD6, O75475, Q9NR56, P16403, Q86UP2, P42677, P15880, Q9BXJ9, P27816, Q9Y383, Q9BRJ2, Q9P2E9, Q9Y2W1, P21399 | 3.025797 | 3.44E-20 |
| GO:0005515~protein binding | 355 | 2.03E-22 | Q9BZZ5, O43670, Q16629, Q9Y266, O14672, O43674, P67809, P49023, P54619, Q04760, Q8NB25, Q9P2R7, Q01130, A0MZ66, Q96GX9, Q9NYU2, Q9NTK5, Q9UMY4, Q92973, P09496, Q14696, Q13243, Q92614, Q14573, Q9Y3Z3, O00712, Q96HY6, P30876, P08729, Q92609, Q9GZZ9, Q15427, O43684, O00154, Q8NE71, | 1.311751 | 6.92E-20 |

| | | | | | |
|---|---|---|---|---|---|
| | | | Q9NR77, P50148, Q9UBT2, O75381, Q96IJ6, P09382, Q9UIA9, P20042, Q16643, P46777, Q9Y281, Q29RF7, Q9Y282, Q9H2G2, P27694, Q9ULX9, Q13136, P35637, Q12965, Q13813, P30626, Q96IZ0, O95573, P30740, Q8IVF2, P42167, Q96CM8, P42285, P63220, O15533, P61289, P35080, Q9UJU6, Q16890, P00441, Q12972, P35749, O95347, Q13268, Q8IWX8, P31948, P26373, Q16537, P18077, Q9BXB5, Q9BYD6, Q8TD19, O75475, Q9UKG1, Q9Y617, Q14919, P62140, Q9NUP9, P43487, Q9Y6B6, O15305, Q13952, P80303, Q9H2M9, A0FGR8, P78344, P28482, P78345, Q4G0F5, Q9UEU0, Q96GK7, Q9Y3E7, Q96RQ3, P21964, P35221, O00592, Q9Y6K9, Q02413, P23588, Q9UFW8, P15170, P61586, P48643, O14744, Q14498, P62312, P49736, P46108, O14745, P07437, O43760, Q9UL25, Q96KB5, Q9H6T3, Q32MZ4, Q9NTX5, Q9H3N1, Q5JTZ9, Q08752, O00116, Q93052, P49721, P07686, Q9UM54, Q6XQN6, P09622, P05107, O43852, O75569, O95810, Q13618, O00244, Q9H6S3, P11586, Q99700, Q02878, Q9UNH7, P49959, P15151, Q96PK6, O95817, P24534, P55010, P61247, Q14157, Q92878, Q15365, P51452, Q13868, P0DP25, O75674, O43865, Q9NR56, Q8TCT9, Q86UP2, Q9BWD1, Q9NY33, Q15370, Q14160, P27816, Q99497, Q9NTJ3, P24666, O14773, Q9Y383, Q15131, Q9BRJ2, P08754, P02533, P29317, Q9Y2W1, A6NHR9, P21399, P53004, O95831, P54577, O96005, Q96L92, Q8IWS0, Q13637, Q9Y6W5, P56199, Q15819, Q08722, Q9H446, Q01650, P18124, P50897, O96000, O60725, Q96S66, Q9Y3A3, O60841, Q9Y3A5, Q96AC1, Q07866, P62877, Q9UH65, P25205, Q15021, P28838, Q8IWB7, P46821, P62753, | | |

| | | | | | |
|---|---|---|---|---|---|
| | | | Q15388, P25325, P20674, P48449, Q13523, O94874, Q13642, Q9Y678, P29144, P49773, P06703, O15013, Q13404, Q9BRQ6, Q01082, P12268, P35249, Q05682, O60610, Q9Y3B7, P84098, Q8TAE8, Q8WX93, O43290, P09543, Q2TAL8, P06396, P60900, Q9Y446, P26640, Q8TEM1, O75400, Q03135, Q9BTT0, P56537, O00203, P11387, P55327, Q9Y6I9, Q9Y3C8, Q14192, P25789, Q9Y450, O14964, P25786, P11717, P56545, Q9H3P7, Q13308, Q8N1F7, P35222, P34932, P12004, P42677, P15880, Q8WXX5, Q8TCG1, Q9BXJ9, Q9UH99, Q92783, O14974, P41227, O43598, P49585, P53680, P23193, Q8WWM7, Q13557, Q6P1N9, Q96BJ3, Q9BYC5, Q12905, Q12906, Q9NX63, Q96PU8, P17987, Q9UJW0, P30050, Q92499, Q969X5, Q13561, Q9Y512, Q07020, P46060, P46063, P14927, Q5VSL9, P19387, Q5VW32, Q07817, Q8IYI6, P49327, P23634, O43491, Q6IQ22, Q27J81, P49207, P61513, P21333, P26440, P09669, P28066, P49321, Q15758, Q6IBS0, P10412, Q9NW64, Q99426, Q9H3U1, P61088, O14929, Q9NZM1, Q92598, P00403, Q16851, O15212, P05198, P62851, Q9BVK6, Q969V3, P47813, O75608, Q14677, Q7Z4H3, O75955, Q14318, P28074, P62191, P28072, P28070, P52943, P16403, P50402, O75031, P17612, Q9NPJ3, O14936, O14818, Q08209, Q8N0X7, Q9NZN9 | | |
| GO:0045296~cadherin binding | 43 | 2.04E-21 | Q9H2G2, Q9Y446, Q13813, Q9Y266, A0FGR8, Q9Y6W5, P78344, Q8WWM7, Q96C19, Q6IBS0, Q9H6S3, A0MZ66, Q9H3U1, Q02878, P42167, P49959, P35221, Q9NTK5, O95817, P55010, Q9UH65, Q96KG9, Q9UJU6, Q15365, P46060, Q14677, Q32MZ4, P35222, | 6.304741 | 4.65E-19 |

| | | | | | |
|---|---|---|---|---|---|
| | | | Q01082, P28072, P50402, Q05682, Q86UP2, P15880, Q14160, Q8TCG1, Q99497, P49327, Q16643, P43487, P29317, P21333, P49207 | | |
| GO:0003735~structural constituent of ribosome | 18 | 7.56E-07 | Q07020, P26373, P18077, Q9BYD6, P18124, P42677, Q9Y3B7, Q02878, P84098, P15880, P30050, P63220, P61247, P46777, P62851, P62753, P49207, P61513 | 4.41262 | 1.29E-04 |
| GO:0003779~actin binding | 24 | 1.39E-06 | Q9NYL9, Q12965, Q96IZ0, Q13813, Q15417, Q9Y6W5, Q6IBS0, Q9H6S3, Q01082, P50402, Q05682, O60610, Q96AC1, Q8WX93, P06396, Q16643, P46821, Q9UM54, P35080, P29966, O43491, Q9UJU6, Q27J81, P07951 | 3.280178 | 1.90E-04 |
| GO:0051015~actin filament binding | 19 | 1.98E-06 | P35749, Q12965, Q13813, Q6IBS0, Q01082, A0MZ66, O75083, Q96AC1, P35221, Q9UEY8, P06396, Q16643, Q92614, Q9UM54, P29966, Q9UJU6, Q9Y281, P07951, P21333 | 3.912523 | 2.25E-04 |
| GO:0004175~endopeptidase activity | 12 | 2.60E-06 | P29144, P28066, O14818, O14672, O14773, P25789, P28074, P49721, P28072, P60900, P28070, P25786 | 6.390691 | 2.53E-04 |
| GO:0035091~phosphatidylinositol binding | 12 | 2.14E-05 | Q96L92, Q9UNH7, Q12965, Q8IYI6, A0FGR8, Q96BJ3, O75674, Q9UMY4, Q92783, Q9UKG1, O14964, Q14573 | 5.148057 | 0.001825 |
| GO:0005524~ATP binding | 57 | 7.93E-05 | P54577, P28482, Q9BQ39, Q13557, P54619, Q9HAB8, Q9P2R7, P17987, Q96RQ3, Q9NTK5, Q9UH65, P25205, Q92499, Q96KG9, P48643, Q92614, P49736, Q13523, P46063, Q96KB5, Q9GZZ9, Q8NE71, Q5JTZ9, P35249, Q9UBT2, Q9P2K8, P23634, Q9UM54, Q9HD20, Q9H2G2, P26640, Q12965, Q6IBS0, P10412, O95573, P11586, P11387, Q96CM8, P61088, P42285, Q92598, O14964, Q92878, P35749, O95347, P62191, Q13308, | 1.710462 | 0.006016 |

| | | | | | |
|---|---|---|---|---|---|
| | | | Q8TD19, P34932, P17612, O14936, Q9NTJ3, Q15131, P49588, P29317, A6NHR9, Q9Y2W1 | | |
| GO:0004298~threonine-type endopeptidase activity | 5 | 1.79E-04 | P25789, P28074, P49721, P28072, P28070 | 16.54733 | 0.012196 |
| GO:0042802~identical protein binding | 59 | 3.53E-04 | O43598, P49585, A0FGR8, P28482, Q13557, Q9BTZ2, Q9HAB8, P18124, Q96GX9, A0MZ66, Q00796, Q9Y3E7, P35221, Q9Y6K9, Q9UFW8, Q14696, O14744, P29966, P25325, Q13561, Q9Y3Z3, P07951, O14745, P29144, P12268, Q07817, O75381, P09382, P49327, P07686, Q9UM54, Q9H2G2, P26440, P35637, P30626, O75569, Q06203, Q13618, Q03135, P49959, Q14192, P55809, Q16851, P61289, Q92878, Q16890, P45954, P00441, P11717, P56545, Q14318, O43865, Q9UKG1, Q9Y617, Q14919, P12004, O14818, Q99497, Q9UH99 | 1.59674 | 0.021927 |
| GO:0003743~translation initiation factor activity | 8 | 4.40E-04 | O60841, P78344, P23588, P55010, P20042, P05198, P47813, P56537 | 5.791564 | 0.025028 |
| GO:0005516~calmodulin binding | 14 | 5.26E-04 | P35749, Q12965, Q13813, P49585, Q15417, Q13557, Q01082, Q05682, O14936, Q9UEY8, Q08209, P23634, Q9UM54, P29966 | 3.164172 | 0.027659 |
| GO:0043022~ribosome binding | 8 | 8.28E-04 | Q9Y3A5, Q9BXJ9, Q9NTK5, Q8NE71, P05198, P41227, Q969V3, P56537 | 5.220565 | 0.040384 |

**KEGG Pathways**

| Term | Count | PValue | Genes | Fold Enrichment | FDR |
|---|---|---|---|---|---|
| hsa05014:Amyotrophic lateral sclerosis | 35 | 1.22E-09 | P09669, P35637, Q16629, P28066, O43674, Q8WWM7, Q8TEM1, O96000, Q99700, O14949, Q9UJW0, Q07866, P25789, P00403, P05198, P35080, O14548, Q13561, Q14573, P20674, P25786, P00441, P07437, P14927, P62191, P28074, Q8N1F7, P28072, O00399, P28070, Q07817, O14818, Q08209, P49721, P60900 | 3.2813 | 3.53E-07 |
| hsa05012:Parkinson disease | 29 | 2.88E-09 | P09669, P28066, O43674, Q13557, O96000, O14949, Q07866, P25789, P00403, P05198, O14548, Q14573, P20674, P25786, P00441, P07437, P14927, P0DP25, P62191, P28074, P28072, P28070, Q07817, P17612, O14818, Q99497, P49721, P08754, P60900 | 3.720452 | 4.16E-07 |
| hsa05020:Prion disease | 29 | 5.19E-09 | P09669, P28066, P28482, O43674, Q03135, O96000, O14949, Q07866, P25789, P00403, P05198, O14548, P07358, Q14573, P20674, P25786, P00441, P07437, P14927, P31948, P62191, P28074, P28072, P28070, P17612, O14818, Q08209, P49721, P60900 | 3.625056 | 5.00E-07 |
| hsa05022:Pathways of neurodegeneration - multiple diseases | 38 | 3.26E-08 | P09669, P35637, P28066, P28482, O43674, Q8WWM7, Q13557, O96000, Q99700, O14949, Q9UJW0, Q07866, P25789, P00403, P05198, O14548, Q13561, Q14573, P20674, P25786, P00441, P07437, P14927, P0DP25, P62191, P28074, P35222, P28072, Q08752, O00399, P28070, P50148, Q07817, O14818, Q08209, Q99497, P49721, P60900 | 2.724306 | 2.36E-06 |
| hsa05016:Huntington disease | 29 | 6.33E-08 | P09669, P28066, P53680, O43674, O96000, O14949, Q9UJW0, Q07866, P25789, P00403, P09496, O14548, | 3.234118 | 3.66E-06 |

| | | | | | |
|---|---|---|---|---|---|
| | | | Q13561, P20674, P25786, P00441, P07437, P30876, P14927, P62191, P28074, P19387, P28072, O00399, P28070, P50148, O14818, P49721, P60900 | | |
| hsa03050:Proteasome | 11 | 6.00E-07 | P28066, O14818, P25789, P62191, P28074, P49721, P61289, P28072, P60900, P28070, P25786 | 8.160451 | 2.89E-05 |
| hsa05010:Alzheimer disease | 30 | 2.14E-06 | P09669, P28066, P28482, O14672, O43674, O96000, O14949, Q9Y6K9, Q07866, P25789, P00403, P05198, O14548, Q14573, P20674, P25786, P07437, P14927, P0DP25, P62191, P28074, P35222, P28072, Q08752, P28070, P50148, O14818, Q08209, P49721, P60900 | 2.666056 | 8.82E-05 |
| hsa03010:Ribosome | 18 | 3.20E-06 | Q07020, P26373, P18077, Q9BYD6, P18124, P42677, Q9Y3B7, Q02878, P84098, P15880, P30050, P63220, P61247, P46777, P62851, P62753, P49207, P61513 | 3.887718 | 1.16E-04 |
| hsa05171:Coronavirus disease - COVID-19 | 19 | 1.43E-04 | Q07020, P26373, P28482, P18077, P18124, P42677, Q02878, P84098, P15880, Q9Y6K9, P30050, P63220, P61247, P46777, P62851, P62753, P07358, P49207, P61513 | 2.794763 | 0.004583 |
| hsa05017:Spinocerebellar ataxia | 14 | 2.65E-04 | P28066, Q8WWM7, P62191, P28074, P28072, P28070, P50148, Q99700, O14818, P25789, P49721, P60900, Q14573, P25786 | 3.34096 | 0.007659 |
| hsa00280:Valine, leucine and isoleucine degradation | 8 | 4.37E-04 | P26440, Q9BWD1, Q96RQ3, P55809, P49189, Q01581, P45954, P09622 | 5.687587 | 0.011492 |
